# Acetyl Group Migration in Xylan and Glucan Model Compounds as Studied by Experimental and Computational Methods

**DOI:** 10.1021/acs.joc.2c01956

**Published:** 2022-10-17

**Authors:** Robert Lassfolk, Manuel Pedrón, Tomás Tejero, Pedro Merino, Johan Wärnå, Reko Leino

**Affiliations:** † Laboratory of Molecular Science and Engineering, Åbo Akademi University, 20500Turku, Finland; ‡ Institute of Biocomputation & Physics of Complex Systems (BIFI), University of Zaragoza, 50009Zaragoza, Spain; § Institute of Chemical Synthesis & Homogeneous Catalysis (ISQCH), University of Zaragoza, 50009Zaragoza, Spain; ∥ Laboratory of Industrial Chemistry and Reaction Engineering, Åbo Akademi University, 20500Turku, Finland

## Abstract

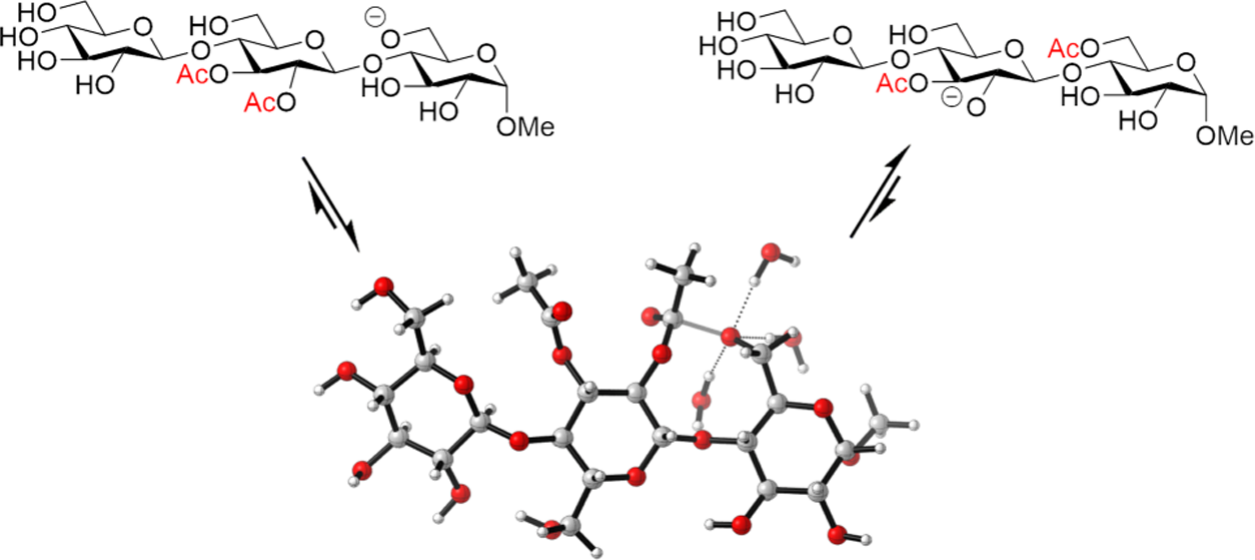

It was recently demonstrated by us that acetyl groups in oligosaccharides can migrate not only within one saccharide unit but also between two different saccharide units. Kinetics of this phenomenon were previously investigated in both mannan model compounds and a naturally occurring polysaccharide. In addition to mannans, there are also several other naturally acetylated polysaccharides, such as xyloglucans and xylans. Both xyloglucans and xylans are some of the most common acetylated polysaccharides in nature, displaying important roles in the plant cells. Considering the various biological roles of natural polysaccharides, it could be hypothesized that the intramolecular migration of acetyl groups might also be associated with regulation of the biological activity of polysaccharides in nature. Consequently, a better understanding of the overall migration phenomenon across the glycosidic bonds could help to understand the potential role of such migrations in the context of the biological activity of polysaccharides. Here, we present a detailed investigation on acetyl group migration in the synthesized xylan and glucan trisaccharide model compounds by a combination of experimental and computational methods, showing that the migration between the saccharide units proceeds from a secondary hydroxyl group of one saccharide unit toward a primary hydroxyl group of the other unit.

## Introduction

**Scheme 1:**
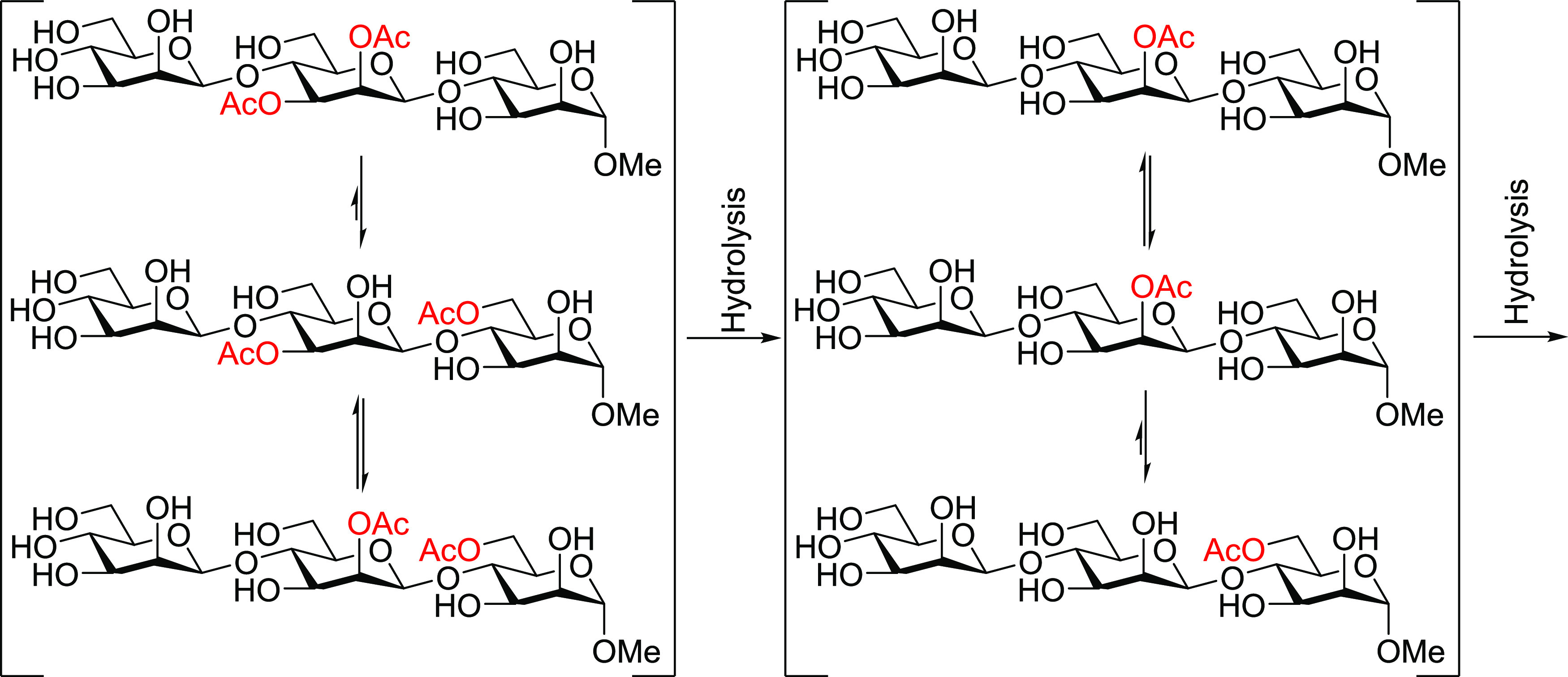
Migration Path in the Previously Disclosed Trimannoside Model Compound

Acyl groups in polyhydroxyl compounds are known to migrate, often complicating the isolation, purification, and synthesis of substances containing multiple hydroxyl groups. Such migration processes are particularly prominent in carbohydrates.^1−3^ The first description of the migration phenomenon was reported by Fischer,^4^ and since then several studies have been performed, mostly on monosaccharides. A typical acyl migration process takes place between two vicinal hydroxyl groups, as also confirmed by unsuccessful attempts to induce the migration of an acetyl group directly from O2 to O6 in a mannopyranoside.^5^ Recently, we have demonstrated that acetyl group migration may take place also across the glycosidic bond, between the O2 and O6 in two different saccharide units of an oligo- or polysaccharide, in a β-(1 → 4)-linked trimannoside model compound (Scheme 1), and even in the corresponding native galactoglucomannan polysaccharide.^6,7^ These findings could contribute to investigations on the regulation of the biological activity or other structural properties in natural polysaccharides.

Mechanism of the migration has been studied in several articles.^7−9^ By a thorough investigation of several different monosaccharides, we have recently demonstrated, through experimental and computational means, that the mechanism of acyl migration may involve two paths, either a neutral or an anionic path (commencing with deprotonation), of which the anionic is the more prominent one at a higher pH.^10^ Furthermore, the rate of the anionic migration path is highly dependent on the p*K*_a_ of the hydroxyl groups involved. Consequently, the concentration of the anion increases with increasing pH, resulting also in an increase in the rate of migration.

Hemicelluloses is the common name for the noncellulosic polysaccharides in plant cells. Such hemicelluloses comprise a diverse group of carbohydrates, possessing the key feature of β-(1 → 4)-linkages in their backbones. The group consists of three main types of polysaccharides: mannans,^11−13^ xylans,^14−16^ and glucans.^17−19^ Xylans are found in a wide variety of plants, from trees to grasses and herbs, making them one of the most common hemicelluloses in nature.^20,21^ A wide structural variety of xylans have been reported, involving different sidechain substitution patterns,^22−24^ often consisting of α-(1 → 2)-linked glucuronosyl and/or 4-*O*-methyl glucuronosyl residues. Such glucuronoxylans are the major hemicellulose type in the secondary cell wall of dicots.^22^ Glucuronoarabinoxylans, in turn, are the major hemicelluloses in the primary cell wall of commelinid monocots.^22^ Xylans are load-bearing components in the cell walls, especially in the secondary cell walls and the xylem, the water-conducting system in plants. Outside of the host plants, some xylans have been found anticarcinogenic and able to improve the beneficial bacterial population growth in the colon.^23,25^

A key similarity between most of the xylans in nature is the acetylation of the O2 and O3 of the xylose units.^20,22,23^ The degree of substitution typically ranges from 30 to 60% with the ratio between the acetyl groups at O2 and O3 varying depending on the source.^15,20^ The acetyl groups are crucial for both the structure and function of the cell walls.^26^ Generally, acetyl groups are evenly spread between the saccharide units in xylans.^27^ Considering the recently established possibility of acetyl migration even between the different saccharide units in oligo- and polysaccharides, across the glycosidic bond,^7^ it could be speculated that the even distribution of the acetyl groups along the xylan backbone could result from such migration processes, finally resulting in an equilibrium concentration. Earlier migration studies in xylans, by nuclear magnetic resonance (NMR) spectroscopy, have concluded that the acetyl group migration proceeds within a single saccharide unit in the chain.^14^ As demonstrated earlier, migration from the secondary to primary hydroxyl group, across the glycosidic linkage, in the model mannan trisaccharide, is slow compared to the rate of migration within the saccharide unit.^6^ Consequently, the potential migration rate between two secondary hydroxyl groups, across the glycosidic linkage, could in xylans be anticipated to be even slower.

Of the naturally occurring acetylated glucans, xyloglucans are the most common ones, found in almost every plant species.^28−30^ This type of glucans consists of β-(1 → 4)-linked glucose backbones containing xylose branches, α-(1 → 6)-linked to the backbone. One key feature of xyloglucans is that they consist of a repetitive pattern and the pattern itself can vary depending on the plant and the corresponding tissue.^22^ Many of the xyloglucans are acetylated at O6 of the unbranched glucose units of their backbones.^31,32^ In plants, xyloglucans function as the main load-bearing component of the primary cell wall,^33−35^ and the xyloglucans have been shown to display at least some regulatory activity during plant cell growth and elongation.^36,37^

Since the pH in the plant cells changes with their life cycle, in particular increasing during the cell growth and development,^38,39^ the rate of migration should likewise increase with increasing pH. Such an increase in the migration rate could potentially be related to the change of chemical and physical properties of the polysaccharides or possibly enhanced cell signaling via migration. Also the position of the acetyl groups and the overall degree of acetylation depend on the development stage of the plant during its life cycle,^40,41^ manifesting their importance in plant development, particularly considering that many biologically active compounds are regulated by acetyl groups.^42,43^ Investigation of the potential migration of acetyl groups across the glycosidic linkages, besides in mannans also in glucans and xylans, could provide insights into how the acetyl groups are positioned after biosynthesis. Hemicelluloses are synthesized in the Golgi lumen, where the acetyl groups are also added to the growing polysaccharide chain. The degree of acetylation and position of the acetyl groups may, however, change after disposition in the cell wall.^41^ Tentatively, this postmodification in the position of the acetyl groups could result from migration processes.

In the present work, we have investigated in detail the acetyl group migration in xylan and glucan model trisaccharides by NMR spectroscopy and kinetic modeling, providing insights besides into the migration process, such as possible migration between secondary hydroxyl groups over the glycosidic bond and the influence of the stereochemistry of C2, also into the calculated rates of migration and hydrolysis of the migrating groups. Furthermore, computational studies have been carried out to confirm the proposed mechanism of migration and for validating our previously disclosed mechanistic model,^10^ in particular when the acetyl group migration takes place between two different saccharide units. Attempts to approximate the p*K*_a_ values involved and the associated rate constants are also briefly discussed.

## Results and Discussion

### Migration Studies

**Figure 1:**
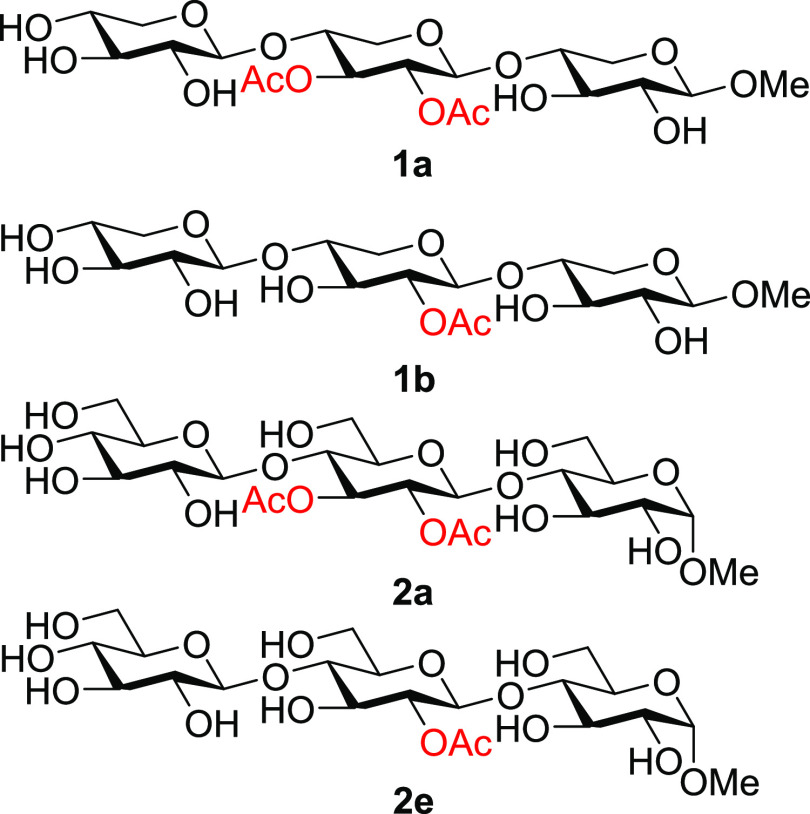
Xylan (**1a**, **1b**) and glucan (**2a**, **2e**) model trisaccharides investigated in this study.

For further investigating the migration between the saccharide units in β-(1 → 4)-linked polysaccharides, model trisaccharides of xylans and glucans were synthesized (for details, see the Supporting Information) and subjected to similar migration conditions as employed in our earlier study on mannan trisaccharides.^6^ The possible migration between the secondary hydroxyl groups in the different saccharide units in xylans was studied with the model compounds **1a** and **1b** (Figure 1). In addition, similar glucan model compounds **2a** and **2e** were used to investigate the possible migration process in glucans, which in combination with our earlier study on mannan model compounds,^6^ will help to shed light on the influence of the stereochemistry at O2 on acetyl migration.

The migrations were followed by NMR spectroscopy. To determine the ratios of the different products, the acetyl peaks were used. In line with our previous study, the potential change in pH of the solution during the migration process was monitored (for details, see Figure S1 in the Supporting Information), and addressed in a similar manner as described earlier for the corresponding mannan trisaccharide model compounds.^6^ Similar migrations and hydrolysis, e.g., hydrolysis from the secondary hydroxyl groups, were set to possess the same rate constant in cases where these could not be differentiated. It can generally be expected that similar types of reactions will have similar rate constants, thereby also simplifying the kinetic model. The errors reported are standard error = variance/(*N*^1/2^) (*N* number of samples), within 95% confidence interval. Single experiments were used for kinetic modeling of specific compounds. Here, it can be noted that by use of mathematical kinetic modeling, combined with experimental methods, it is possible to identify whether an experimental point, or one complete experimental series for a specific compound, would not fit well to the model. Consequently, it becomes possible to identify problematic experimental data points. Such experiments can then be repeated, or alternatively analysis of the specific data point rechecked.

**Scheme 2:**
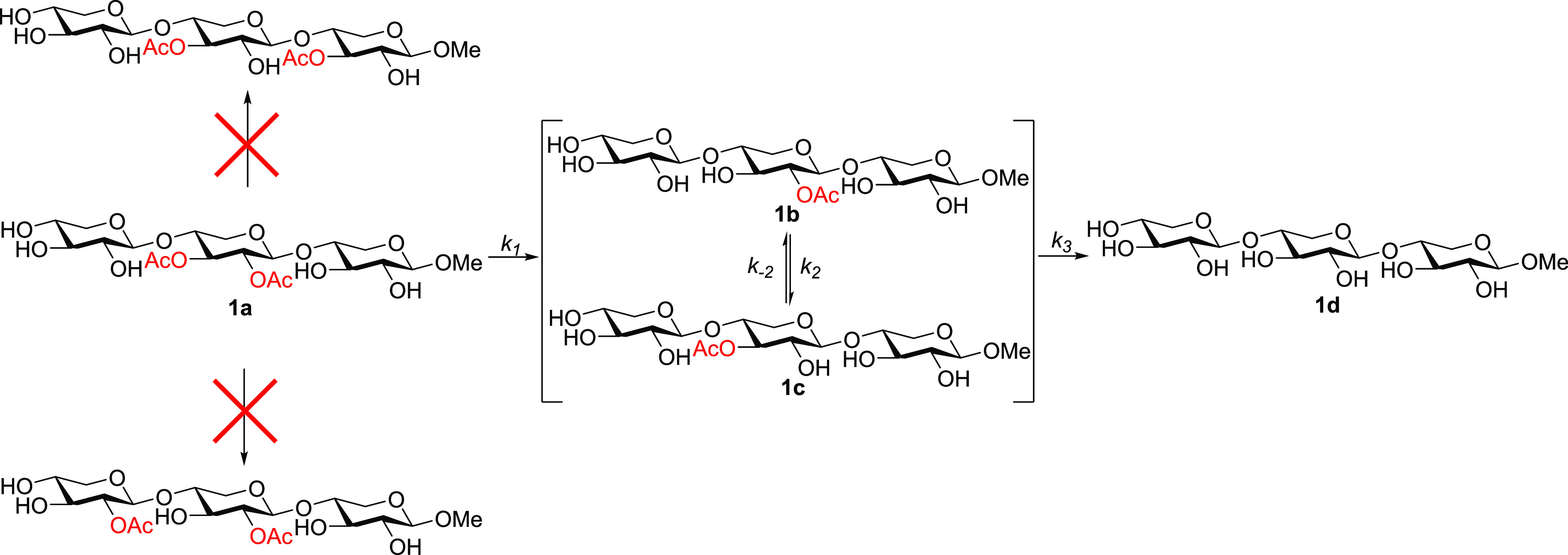
Migration and Hydrolysis Paths in the Xylan Model Trisaccharides

Migration between the different saccharide units in the xylan trisaccharides was anticipated to be slow, if present at all. From both starting points **1a** and **1b**, no migration between the saccharide units was observed, resulting in the migration and hydrolysis path displayed in Scheme 2. The prerequisite conformation of the oligosaccharide chain and the distance between the acetylated hydroxyl group, O2 and O3, and a free hydroxyl group in the neighboring saccharide unit needed for the migration to take place are most likely energetically unfavorable, preventing the migration between two nonadjacent hydroxyl groups.

**Figure 2:**
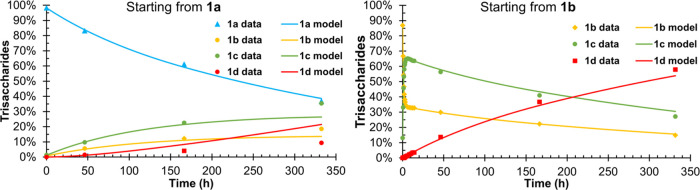
Experimental data and the kinetic model of the acetyl group migration starting from model compounds **1a** and **1b**. Conditions: 100 mM phosphate solution with 10% D_2_O, pH = 8, 25 °C. Degree of explanation: 99.31%.

For model compound **1b**, a migratory equilibrium between the O2 and O3 of the middle unit was established within 3 h, resulting in an acetylation ratio between O2 and O3, **1b** and **1c**, of approximately 1:2 (Figure 2). This migration between two adjacent hydroxyl groups is, as expected, fast, ca. 100–200 times faster than the acetyl group hydrolysis from the positions O2 and O3, meaning that no differentiation between the rates of hydrolysis of the acetyl group from O2 and O3 can be established (Table 1). The rate of hydrolysis of the acetyl groups from **1a** is 40% slower than the hydrolysis from **1b** and **1c**, most likely inferred by steric hindrance of the neighboring adjacent acetyl group.

**Table 1:** Rate Constants at pH = 8 for the Acetyl Group Migration Displayed in Scheme 2^a^

rate constants for the xylotrisaccharide (h^–1^)	
*k*_1_	1.82 × 10^–3^ ± 1.28 × 10^–4^
*k*_2_	6.33 × 10^–1^ ± 5.32 × 10^–2^
*k*_–2_	3.25 × 10^–1^ ± 3.58 × 10^–2^
*k*_3_	3.01 × 10^–3^ ± 1.94 × 10^–4^

a Conditions: 100 mM phosphate solution with 10% D_2_O at 25 °C, starting pH = 8.

It could be argued that the possibly formed migration products (for migration across the glycosidic bond) cannot be distinguished from the starting compounds. However, because of the two starting points, comparison of the acetyl peaks should indicate whether migration between the saccharide units takes place or not. The lack of migration between the saccharide units could imply that the acetyl groups are placed on the specific saccharide unit already during the biosynthesis.

**Figure 3:**
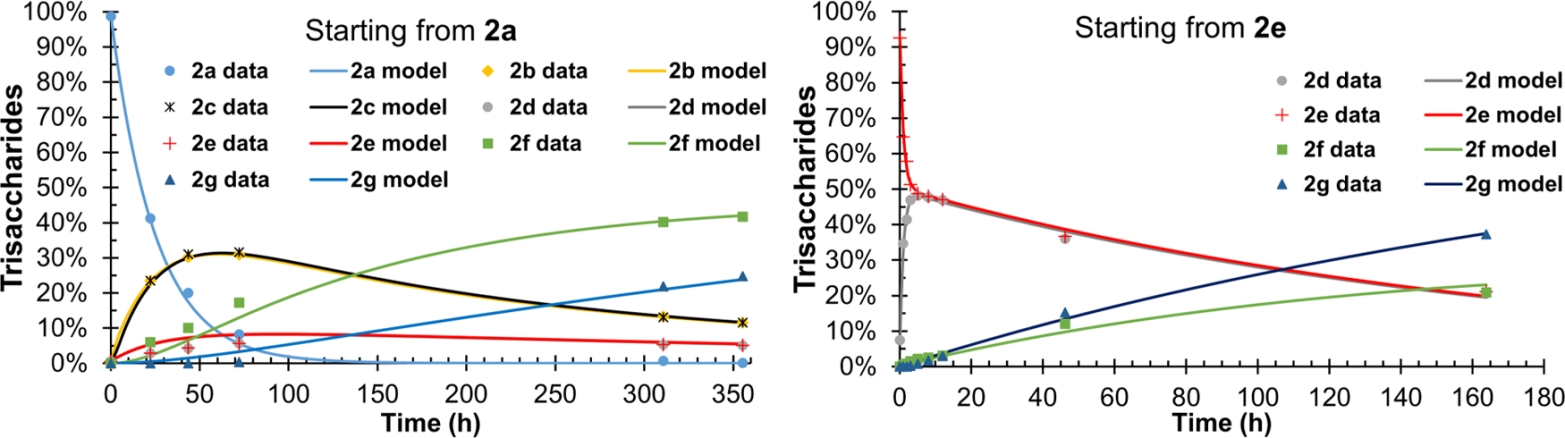
Experimental data and the kinetic model of the acetyl group migration starting from model compounds **2a** and **2e**. Conditions: 100 mM phosphate solution with 10% D_2_O, pH = 8, 25 °C. Degree of explanation: 99.45%.

**Scheme 3:**
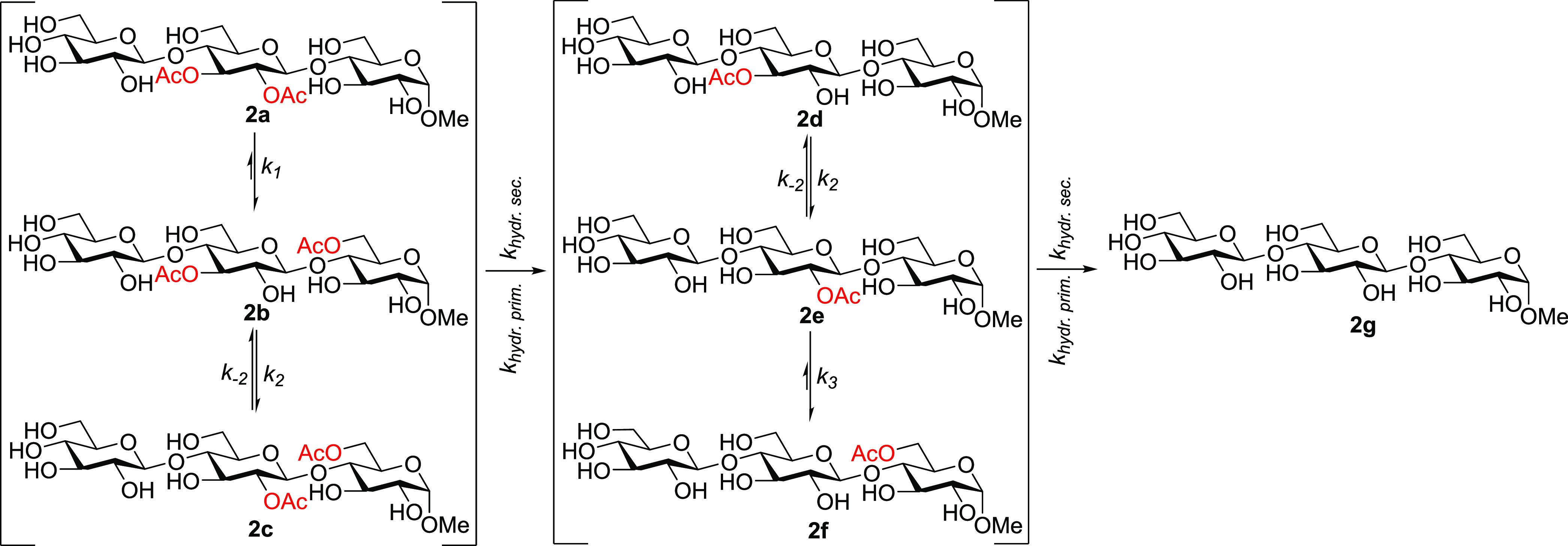
Migration and Hydrolysis Path for Glucan Trisaccharides

In glucose, the equatorial O2 will influence the rate of migration between the saccharide units compared to the previously studied mannan model trisaccharide.^6^ The acetyl group in the glucan trisaccharide will be closer in space to the O6 of the reducing end saccharide unit, when considering the way O6 moves toward the acetyl group in the mannan trisaccharide.^7^ The observed rate of migration in the glucan model trisaccharide **2a** is several times faster than the corresponding O2 → O6 migration rate in the mannan trisaccharide. It takes less than 24 h for the concentration of **2a** to decrease below 50% (Figure 3). The **2e** → **2f** migration rate is also significantly slower, ca. 7 times, compared to the **2a** → **2b** migration rate (Table 2). The observed **2e** → **2f** migration rate is, nevertheless, approximately 2 times faster in the glucan trisaccharide studied, compared to the O2 → O6 migration in the mannan trisaccharide. This could possibly be explained by the conformational dynamics of the trisaccharide and also the configuration of C2. A similar phenomenon was not observed with the mannan trisaccharide,^6^ possibly due to the axial configuration of O2 in mannose. Because of the large difference between the rates of **2a** → **2b** and **2e** → **2f** migrations, simplification to a general O2 → O6 migration could not be established, resulting in the migration and hydrolysis paths illustrated in Scheme 3.

**Table 2:** Rate Constants at pH = 8 for the Acetyl Group Migration in Scheme 3^a^

rate constants for the glucan trisaccharide (h^–1^)	
*k*_1_	3.33 × 10^–2^ ± 1.62 × 10^–3^
*k*_2_	4.92 × 10^–1^ ± 5.46 × 10^–2^
*k*_–2_	4.82 × 10^–1^ ± 4.58 × 10^–2^
*k*_3_	4.85 × 10^–3^ ± 5.08 × 10^–4^
*k*_hydr. prim._	1.35 × 10 ^–3^ ± 1.55 × 10^–4^
k_hydr. sec._	3.24 × 10^–3^ ± 1.50 × 10^–4^

a Conditions: 100 mM phosphate solution with 10% D_2_O at 25 °C, starting pH = 8.

The fast migration between the saccharide units in the glucan trisaccharides could be one of the explanations for why acetyl groups are not found in the secondary positions of the glucose units in natural glucans. When starting from **2a**, the major migration product after 2 weeks is **2f** (approx. 40%), with one acetyl group in the O6 position, followed by the fully hydrolyzed compound **2g** (approx. 20%). Starting from **2e**, the concentration of the fully hydrolyzed compound **2g** increases faster than the concentration of **2f**, due to hydrolysis from the secondary position being almost as fast as the **2e** → **2f** migration. The slower hydrolysis from the primary position compared to the secondary in oligosaccharides has not been addressed in earlier studies, possibly contributing to O6 being the major site of acetylation in natural glucans.

For the glucan trisaccharide, the ratio between O2 and O3 is 1:1 while for the xylan trisaccharide the ratio is approximately 1:2. It seems that steric hindrance induced by the primary position of the nonreducing end in the glucan trisaccharide, or possible hydrogen bonding, would hinder the acetyl group migration to O3, pushing the acetyl group toward the O2 position, therefore changing the ratio of acetylated O2 and O3. This interaction demonstrates how subtle changes in the conditions or chemical structures influence the migration phenomenon and the migration rates. Differences in the reported degree of acetylation between the O2 and O3 of isolated xylans from plants could potentially be due to hydrogen bonding between some of the hydroxyl groups, thereby influencing the preferred position of the acetyls.^15,20^

The degree of acetylation will undoubtedly influence the biological properties of a polysaccharide and the possible migration might change the way by which glucans interact with different compounds in their vicinity. Since xyloglucans have been demonstrated to be involved in cell elongation,^37^ it is conceivable that the migration could also change the physical properties to allow for the elongation to take place. For example, when an acetyl group is located at a secondary position, the primary position could still hydrogen bond to other polysaccharides, stiffening the cell wall. Upon migration, the hydrogen bond is broken allowing the elongation to proceed. Observations that possible interactions between the polysaccharide chains affect the rate of migration have been made in migration studies on native galactoglucomannan, where the migration from O2 to O6 was slower at higher polysaccharide concentration, where hydrogen bonding is more likely to occur.^6^ More detailed studies regarding the influence of acetyl group migration on the interactions between polysaccharide chains are, however, still needed for better understanding of such processes.

### Computational Studies

**Figure 4:**
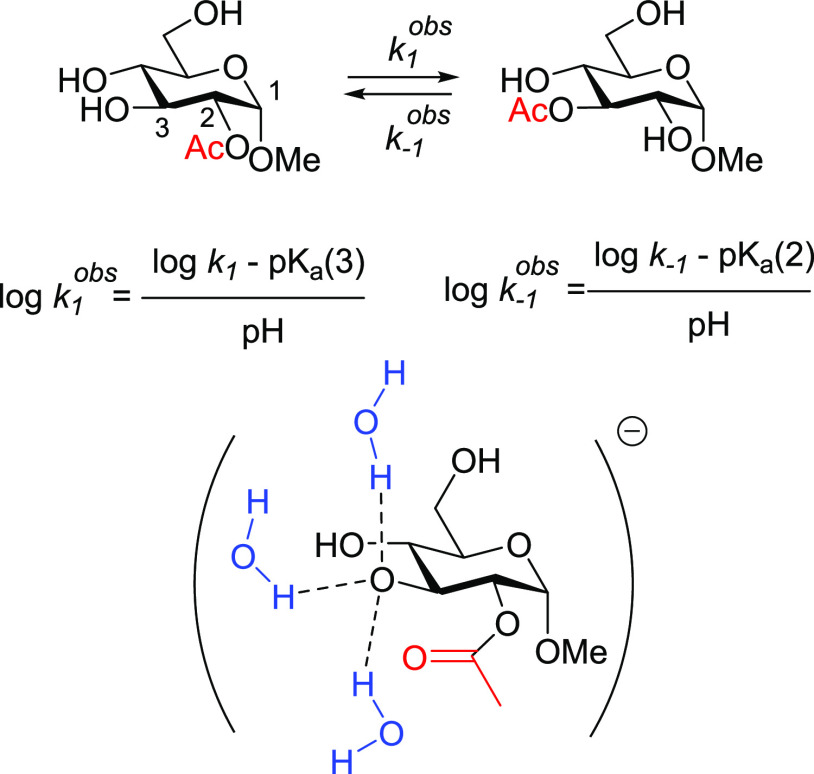
Top: Calculation of the observed kinetic constants for an acetyl group migration at pH > 6. Bottom: Anionic model with the three explicit molecules of water.

Next, computational studies of the acetyl migration in the gluco derivatives **2a**–**2f** were performed. For the purpose of comparison, calculations of the previously reported acetyl group migration in mannan trisaccharides were also performed (see the Supporting Information). We have previously demonstrated^10^ that two mechanisms (neutral and anionic) can operate and that at pH > 6 contribution of the neutral mechanism is essentially negligible, the anionic mechanism being the only operating one. In our previous paper, we also demonstrated the validity and efficacy of considering an anionic model with three explicit molecules of water and the requirement of calculating the corresponding p*K*_a_ values for each deprotonation equilibrium, since the formation of the anion depends on the p*K*_a_ of the corresponding hydroxyl group. For the migration of an acyl group in a monosaccharide at basic pH, the observed kinetic constants can be approached by the expression given in Figure 4, where *k*_1_ and *k*_*–*1_ are, respectively, the direct and reverse formal (as these should be calculated from the corresponding individual barriers of the two steps of the migration) kinetic constants of the acetyl group migration following an anionic mechanism,^10^ and p*K*_a_(*x*) is the p*K*_a_ of the hydroxyl group at *x*-position.

**Scheme 4:**
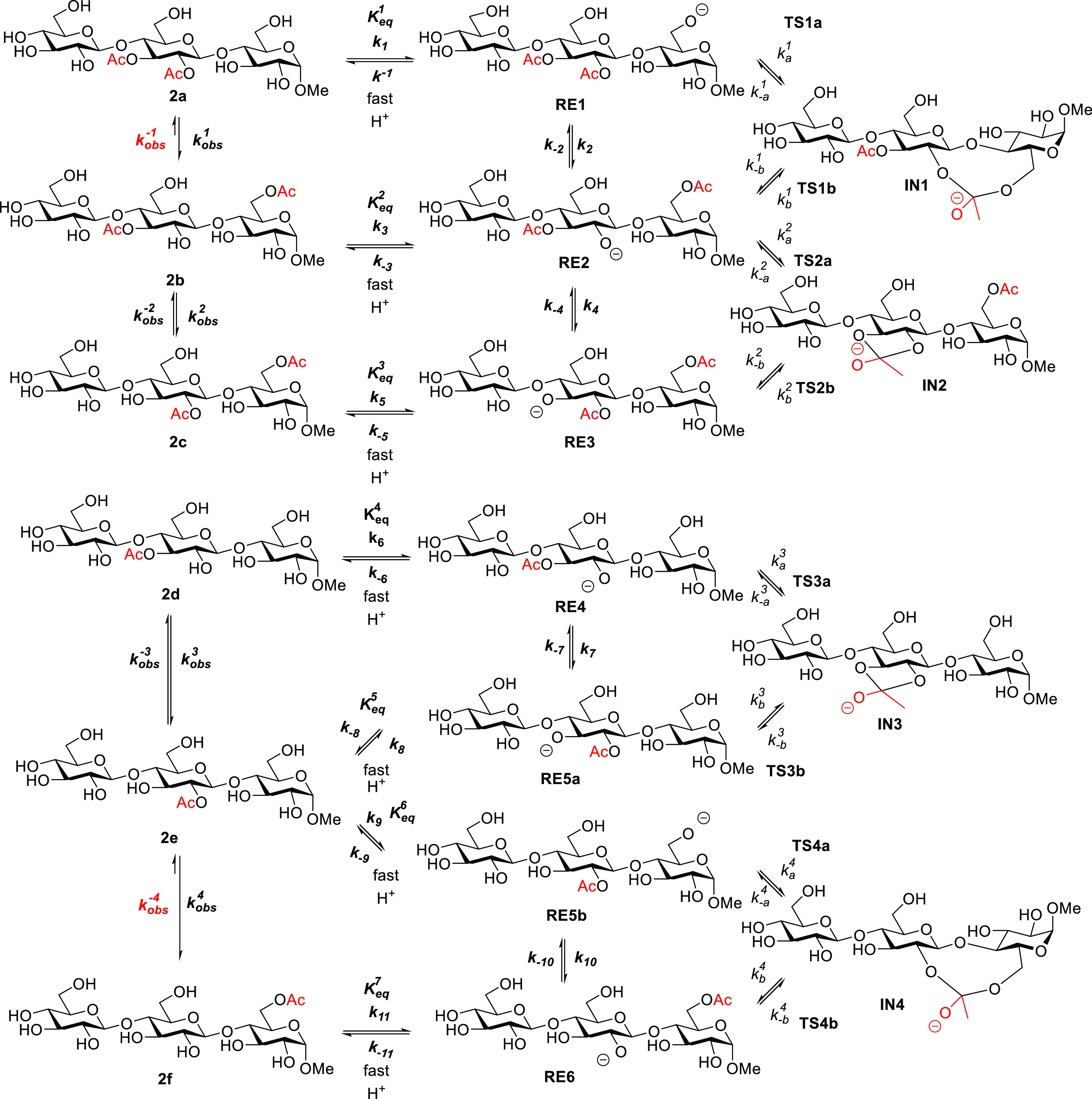
Acetyl Group Migration in Glucan Trisaccharides. For Clarity, the Three Explicit Molecules of Water Added for the Calculations Are Not Displayed

Hence, we applied this model to the acetyl group migration in the glucan trisaccharides **2a**–**2f** and calculated both the p*K*_a_ values and the anionic mechanisms, considering in all cases the presence of three explicit molecules of water (Scheme 4).

**Figure 5:**
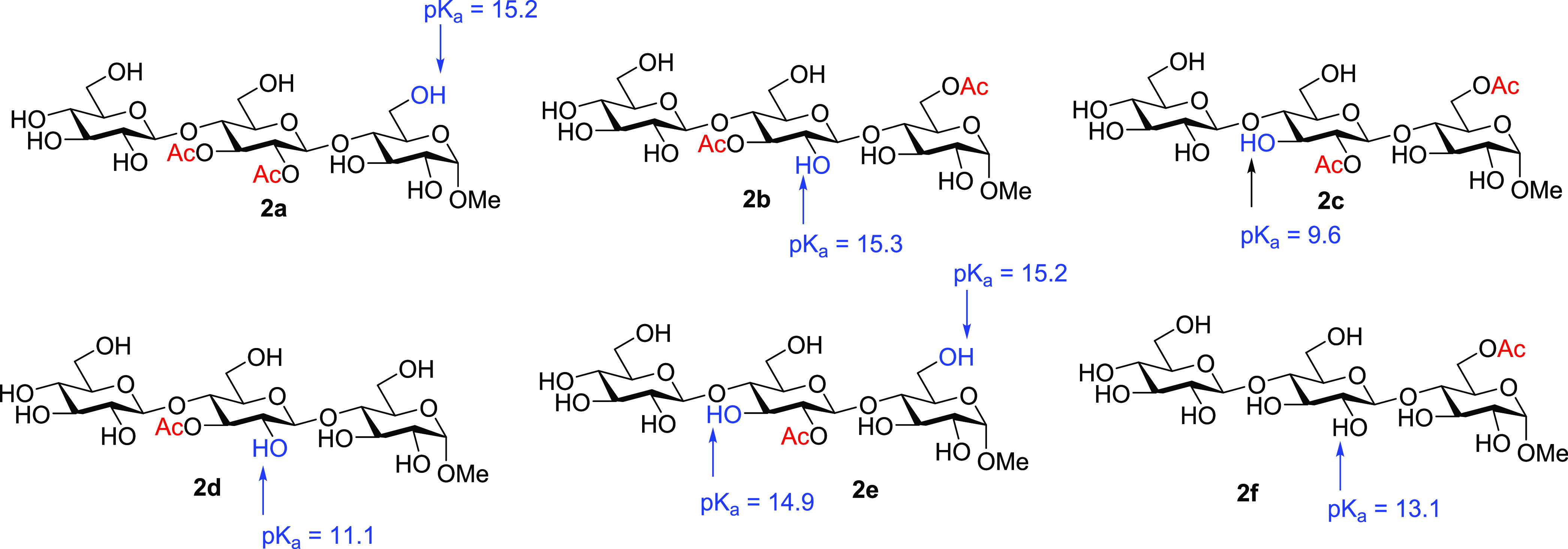
Calculated (wb97xd/def2tzvp/SMD = water) p*K*_a_ values for hydroxyl groups involved in acetyl group migration processes.

**Figure 6:**
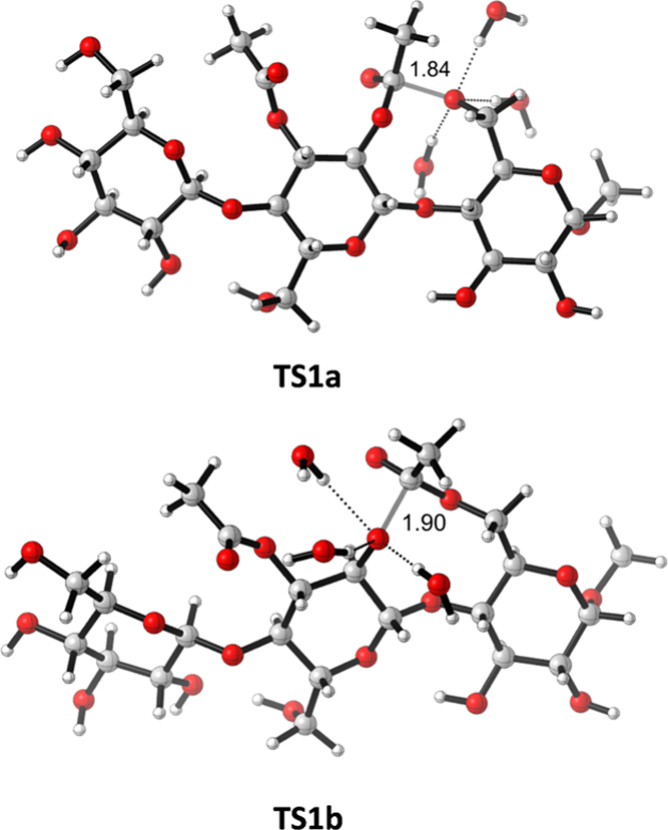
Optimized (wb97xd/6-31+G(d,p)/SMD = water) geometries of the transition structures **TS1a** and **TS1b**, corresponding to the transformation of **RE1** into **RE2**.

The high number of conformations within a narrow range of energy makes it very difficult to accurately determine the geometry of the trisaccharides. The possibility of forming different hydrogen bonds causes such conformations to be in the range of energy up to 5 kcal/mol or more, distorting any calculation on a process involving transition structures and intermediates. To minimize this issue, we first carried out a conformational study of the trisaccharides to determine the preferred conformations and interactions in the different saccharide units and between them, with the aim of selecting stationary points of minimum energy. For that purpose, we performed in parallel molecular dynamics (MD) studies and conformational searches with Macromodel.^44^ Both analyses were coincident, and the structures obtained in a window of 2.0 kcal/mol were optimized at the density functional theory (DFT) level to select those of minimum energy in each case (see the Supporting Information for details). A preliminary benchmark among the previously well-established levels of theory for this type of studies^45^ confirmed wb97xd/6-311++G(d,p)/SMD = water as that better approaching the experimental results when applied to geometries optimized at wb97xd/6-31+G(d,p)/SMD = water level of theory. We then located and characterized all of the minima corresponding to protonated **2a**–**f** and deprotonated **RE1**–**6** substrates. The obtained energy values for these compounds were then used for calculating the p*K*_a_ values of the different hydroxyl groups involved in an acetyl group migration. We used the same protocol that was implemented for monosaccharides in our earlier study.^10^ The p*K*_a_ values were determined by calculating the difference between the solvated states following eq 1, where the aqueous phase proton free energy is −265.9 kcal/mol according to the literature^9,45,46^ and the free energy change due to changing the standard state from 1 atm to 1 M is 1.89 kcal/mol (eq 2).
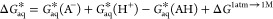
1
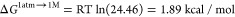
2Further application of eq 3 provided the p*K*_a_ values (Figure 5). Admittedly, there is some variability in the observed p*K*_a_ values, but it should be taken into consideration that according to the logarithmic dependence of free energy and the DFT error,^47^ those values have a confidence interval of, at least, two units. Calculated p*K*_a_’s are subjected to the DFT error, estimated in 1–2 kcal/mol when using ultrafine grid,^47^ resulting in a range (according to eq 3) of ±1.4 units of p*K*_a_. In the case of compounds **2c** and **2d**, the observed low p*K*_a_ values are due to an overstabilization of the deprotonated form caused by more stable conformations due to the presence of extra hydrogen bonds.
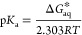
3Next, transition structures **TS1**-**4a,b** were also located and characterized at the same level of theory. In all cases, we employed the above-mentioned model including three explicit molecules of water. The geometries of the saccharides involved in **TS2a,b** and **TS3a,b** and the intermediate anions **IN2** and **IN3** were similar to those obtained in our previous study with monosaccharides, showing the typical five-membered orthoester structure for a stepwise process. On the other hand, **TS1a,b** and **TS4a,b**, as well as the intermediates **IN1** and **IN4**, are substantially different since they come from a migration between the saccharide units and form a nine-membered ring orthoester intermediate with substantial conformational flexibility. It has been a considerable computational effort to consider the conformational variability of both the transition structures and the intermediates. As an example, the optimized structures for **TS1a** and **TS1b** are illustrated in Figure 6. The preferred conformation determined through the corresponding conformational study in each case is conserved during the whole acetyl group migration to avoid distortion in the energy values. Nevertheless, it should be mentioned that, considering the conformational variability in a range of 2 kcal/mol and the DFT error of 1–2 kcal/mol, our approach cannot discriminate between values of less than 2 kcal/mol. Consequently, any difference within this range should be considered under such uncertainty.

The analysis of the corresponding barriers for the individual steps of the acetyl group migration (i.e., *k*_a_^n^, *k*_–a_^n^, *k*_b_^n^, and *k*_–b_^n^; *n* = 1, 2, 3, 4) according to the kinetics of the process (see the Supporting Information) provided the formal rate constants and, by extension, the direct and inverse barriers for acetyl group migrations under an anionic mechanism (Table 3).

**Table 3:** Calculated (wb97xd/6-311++G(d,p)/SMD = Water//wb97xd/6-31+G(d,p)/SMD = water) Formal Energy Barriers^a^ and Rate Constants^b^ for the Acetyl Group Migration in the Glucan Trisaccharides under an Anionic Mechanism Considering Three Explicit Molecules of Water^c^

	rate constant (s^–1^)	Δ*G*(kcal/mol)
*k*_2_	2.68	16.9
*k*_–2_	5.23 × 10^–5^	23.3
*k*_4_	3.75 × 10^2^	13.9
*k*_–4_	1.10 × 10^–3^	21.5
*k*_7_	1.07 × 10^–1^	18.8
*k*_–7_	5.28	16.5
*k*_10_	1.33 × 10^–1^	18.6
*k*_–10_	7.57 × 10^–9^	28.5

a Obtained from the individual barriers of the stepwise mechanism.

b Obtained by applying previously reported kinetic equations (see the Supporting Information).

c All of the calculated values are subjected to the DFT error estimated in 1–2 kcal/mol.^47^

Application of the equations given in Figure 4, including the calculated p*K*_a_ values and considering pH = 8, provides the values and the corresponding formal energy barriers for the acetyl group migration process experimentally observed. The predicted values have been listed in Table 4 with the experimentally observed values included for the purpose of comparison.

**Table 4:** Calculated (wb97xd/6-311++G(d,p)/SMD = Water//wb97xd/6-31+G(d,p)/SMD = Water) Formal Energy Barriers^a^ and Rate Constants^b^ for the Acetyl Group Migration in the Glucan Trisaccharides at pH = 8^c^

	experimental rate constants (s^–1^)	Δ*G*(kcal/mol)	predicted rate constants (s^–1^)	Δ*G*(kcal/mol)	ΔΔ*G*^d^(kcal/mol)
*k*_1_^obs^	9.25 × 10^–6^	24.3	1.56 × 10^–07^	26.7	2.4
*k*_–1_^obs^^e^	<1.00 × 10^–14^	>36.0	2.80 × 10^–12^	33.2	f
*k*_2_^obs^	1.37 × 10^–4^	22.7	2.01 × 10^–05^	23.9	1.2
*k*_–2_^obs^	1.34 × 10^–4^	22.7	2.52 × 10^–05^	23.7	1.0
*k*_3_^obs^	1.37 × 10^–4^	22.7	8.97 × 10^–05^	23.0	0.3
*k*_–3_^obs^	1.34 × 10^–4^	22.7	6.79 × 10^–07^	25.9	3.2
*k*_4_^obs^	1.35 × 10^–6^	25.5	8.25 × 10^–09^	28.5	3.0
*k*_–4_^obs^^e^	<1.00 × 10^–14^	>36.0	6.73 × 10^–14^	35.4	f

a Obtained from the individual barriers of the stepwise mechanism.

b Obtained by applying previously reported kinetic equations (see the Supporting Information).

c All of the calculated values are subjected to the DFT error estimated in 1–2 kcal/mol.^47^

d Difference between experimental and calculated values.

e Since this constant has not been observed experimentally, it is considered very low and an arbitrary valor has been employed.

f This error cannot be considered because the experimental value has not been measured, just confirming that this process is very slow.

The logarithmic dependence of the rate constants on the activation energy makes small variations of the latter to result in substantial variations of the former. Because of that, to evaluate the accuracy of the predictions, it is more suitable to consider the energy barriers for which it is possible to assume an error of 1.2 kcal/mol.^47^ Under these considerations, and despite some clear deviations, the predicted values listed in Table 4 can be considered to be in acceptable agreement with the experimental results. The calculations correctly predicted the nonobserved barriers as the highest ones (corresponding to *K*_–1_^obs^ and *K*_–4_^obs^) and with high values (more than 30 kcal/mol) confirming the difficulty of migrating from a primary position to a secondary one. Similarly, although with some deviation, the highest observed barriers could be identified (corresponding to *K*_1_^obs^ and *K*_4_^obs^). Concerning the remaining barriers, they are predicted correctly with the only exception of *K*_*–*3_^obs^. Admittedly, as already mentioned above, it is not possible to differentiate processes that are in a range of 2 kcal/mol. These predictions have also been applied to acetyl group migration in previously reported^7^ mannan trisaccharides and even better results have been obtained (see the Supporting Information), demonstrating the applicability of the method developed for monosaccharides^10^ for more complex structures. In fact, in the case of acetyl group migrations within the same carbohydrate unit, the situation can be considered identical to that of isolated monosaccharides. Interestingly, although a completely different structural approach must be considered in acetyl group migrations between different saccharide units, in which a nine-membered ring is formed as the intermediate, the defined protocol continues to be valid. The same mechanisms operate between carbohydrate units and the reaction shows the same pH dependence as that observed in monosaccharides. The high barrier observed for the migration from a primary alcohol to a secondary one can be attributed not to a particular transition structure but to the high stability of the substrate acetylated in the primary alcohol. Consequently, the ultimate reason for the observed absence of acetyl group migration from primary to secondary alcohols is not kinetic but thermodynamic.

## Conclusions

It has been demonstrated here that acetyl group migration between the saccharide units in an oligosaccharide, evidently applying to the corresponding polysaccharides as well, can only take place when the acetyl group can migrate from a secondary position to a primary (it can be noted that the preferential acetylation of primary hydroxyl groups over secondary groups has been recently reported^48^). Such migration between the saccharide units is much faster in the glucan trisaccharide studied compared to the earlier described mannan trisaccharide model compound and is completely lacking in xylan oligosaccharides. The increased rate of migration in the glucan trisaccharide is most likely due to the equatorial O2 being closer in space to the O6 of the reducing end unit of the compound. Computational studies agree with the experimental values when considering a model based on an anionic mechanism with the participation of three explicit molecules of water. The model also considers the dependence of the pH and the p*K*_a_ of the hydroxyl group involved in the migration and can be applied to migrations between different saccharide units (as in oligosaccharides). However, in that case, it is strictly necessary that a comprehensive conformational study of all of the stationary points involved in the process is carried out to determine the preferred conformations at each point. Considering that the pH increases during cell growth and that acetyl groups have an important role in the regulation of biological activity, the role of xyloglucans during plant cell growth and elongation could possibly be regulated by acetyl group migration. Further studies regarding this must, nevertheless, be performed to elucidate the potential role of the migration in nature and for verifying such hypotheses.

## Experimental Section

Synthesis of the model compounds is described in detail in the Supporting Information. Structural assignments were made with additional information from gCOSY, gHSQC, and gHMBC experiments.

### Preparation of the Migration Samples

For monitoring the acetyl group migration by NMR spectroscopy, a phosphate solution was used. First, a 100 mM phosphate solution with 10% D_2_O and pH = 8 was prepared. A concentration of 2 mg/ml was used for the migration studies.

### Migration Studies

For following the migration process, a Bruker Avance-III spectrometer operating at 500.20 MHz (^1^H) and 125.78 MHz (^13^C) equipped with a Smartprobe: BB/1H was used. The migration was followed with water-suppressed ^1^H. The acetyl peaks were used to follow the migration. The ratios of the migration and hydrolysis products were obtained using the NMR simulation software Chemadder/Spinadder.^49^

### Migration Kinetics and Kinetic Modeling

The reaction kinetics for the acetyl group migration for the trisaccharides **1a** and **1b** was described with a reversible (O2 ⇌ O3 migration) and irreversible (hydrolysis) first-order reaction scheme as follows
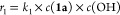

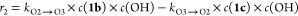

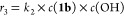

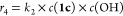
where
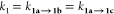

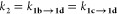
and
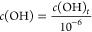
where *c*(OH)*_t_* is the concentration of [OH^–^] at a certain time *t* based on pH and 10^–6^ is the concentration of [OH^–^] at pH = 8. The mass balances become
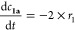

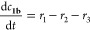

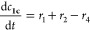

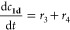
The fit of model to experimental data was good, and the fit is displayed in more detail in the Supporting Information.

The reaction kinetics for the acetyl group migration for the trisaccharides **2a** and **2e** was described with a reversible (O2 ⇌ O3 migration) and irreversible (hydrolysis and O2 → O6 migration) first-order reaction scheme as follows
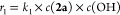

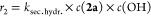

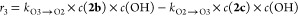

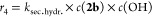

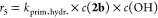

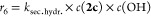

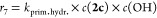

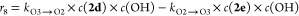

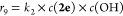

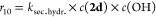

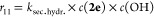

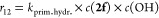
where
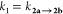

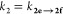

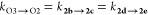

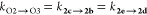

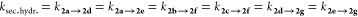

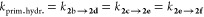
and
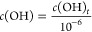
where *c*(OH)*_t_* is the concentration of [OH^–^] at the time *t* based on pH and 10^–6^ is the concentration of [OH^–^] at pH = 8. The mass balances become
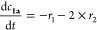

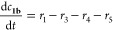

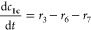

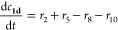

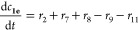

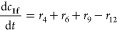

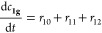
The differential equations are solved with the backward difference method as a subtask to the optimizing methods (simplex and/or Levenberg–Marquardt) with the software Modest.^50^ As objective function, the sum of square function was used
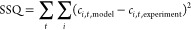


## Notes

The data underlying this study are available in the published article and its online supplementary material.

## Supporting Information Available

Synthetic procedures, NMR spectra, migration data, and computational modeling (PDF)
