# Supplementary material for: Acetyl Group Migration in Xylan and Glucan Model Compounds as Studied by Experimental and Computational Methods
Source: J Org Chem. 2022 Oct 17;87(21):14544–54. doi: 10.1021/acs.joc.2c01956 (PMC9639004; doi:10.1021/acs.joc.2c01956)
Supplement: Supplementary file 1 — jo2c01956_si_001.pdf [file jo2c01956_si_001.pdf]

Supporting Information

Acetyl group migration in xylan and glucan model compounds as studied by experimental and computational methods

Robert Lassfolk[a], Manuel Pedrón[b], Tomás Tejero[c], Pedro Merino[b,*], Johan Wärnå[d] and Reko Leino[a,*]

[a] *Laboratory of Molecular Science and Engineering, Åbo Akademi University, 20500 Turku, Finland.*

[b] *Institute of Biocomputation & Physics of Complex Systems (BIFI), University of Zaragoza, 50009 Zaragoza, Spain.*

[c] *Institute of Chemical Synthesis & Homogeneous Catalysis (ISQCH), University of Zaragoza, 50009 Zaragoza, Spain.*

[d] *Laboratory of Industrial Chemistry and Reaction Engineering, Åbo Akademi University, 20500 Turku, Finland.*

**E-mail: reko.leino@abo.fi*

**E-mail:* pmerino@unizar.es

CONTENTS

Synthesis S3

Experimental procedures S6

NMR spectra S20

Migration data S58

Changes in pH S58

Experimental data points S58

Xylan trisaccharides S58

Glucan trisaccharides S59

Computational Studies S60

DFT calculations. General methods. S60

Kinetics S61

Conformational studies S66

Molecular Dynamics S66

Conformational search S67

Determination of pKa values S73

Energies S74

Predicted Values S76

Transition Structures S77

Cartesian Coordinates S79

References S181

Synthesis

The synthesis of both xylan trisaccharides **1a** and **1b** commenced with the well-established glycosylation method using acetimidate donor **3**[1] and acceptor **4**[2] to give compound **5** (Scheme S1). This was followed by the standard procedure for removing benzoyl groups under Zemplén conditions,[3] followed by the addition of benzyl groups to provide **6** in a fair yield. Next, the propylidene group was removed under acidic conditions to give the disaccharide intermediate **7** in a fair yield. The syntheses of **1a** and **1b** differ from this point onwards. For preparation of **1a**, both of the free hydroxyl groups in **7** were fully acetylated to give **8** in excellent yield. For **1b** instead, selective acetylation of O2 in compound **7** was achieved by using 1.3 equiv. of Ac_2O at 0 °C in pyridine to provide **9** in good yield. Next, both disaccharide donors were glycosylated with the acceptor **10**[4] using the NIS/TMSOTf method. Both compounds **11** and **12** were isolated in good yields. Finally, hydrogenolysis of **11** and **12**, respectively, gave the desired products **1a** and **1b** in excellent yields.

The glucan trisaccharides were synthesized in a similar manner as the xylan trisaccharides, although the first glycosylation posed a problem in the beginning. The first attempts to prepare a glucan disaccharide were carried out using a PMB protecting group instead of Nap in compound **14**. It appeared that the PMB group was not stable under the acidic conditions employed and, consequently, Nap was preferred instead (Scheme S2). The first acceptor is synthesized from the well-known compound **13**[5] by adding the Nap groups, followed by selective ring opening to provide compound **14** in 37% yield. The Nap group is also much more stable than the PMB group, allowing for the use of selective ring opening. Next, using the well-known donor **15**[6], disaccharide **16** was prepared in good yield, using the standard method for glycosylation with trichloroacetimidate donors. This was followed by two protecting group manipulations: First, the benzoyl groups were exchanged for benzyl groups. This was achieved using standard procedures, providing compound **17** in good yield. Next, the Nap groups were removed using DDQ in dichloromethane, water and MeOH, giving **18** also in good yield. Here, the synthesis of compounds **2a** and **2e** differ. For preparation of **2a**, a full acetylation of **18** gave compound **19** in fair yield, while for **2e** a selective acetylation of the O2 position was achieved by using Ac_2O in pyridine at 0 °C, providing compound **20** in 65% yield. Subsequently, both compounds were subjected to glycosylation reactions using compound **21**[7] as the acceptor, resulting in good yields for both compounds (**22**: 62%, **23**: 64%). Finally, hydrogenolysis provided the desired unprotected glucans in excellent yields.

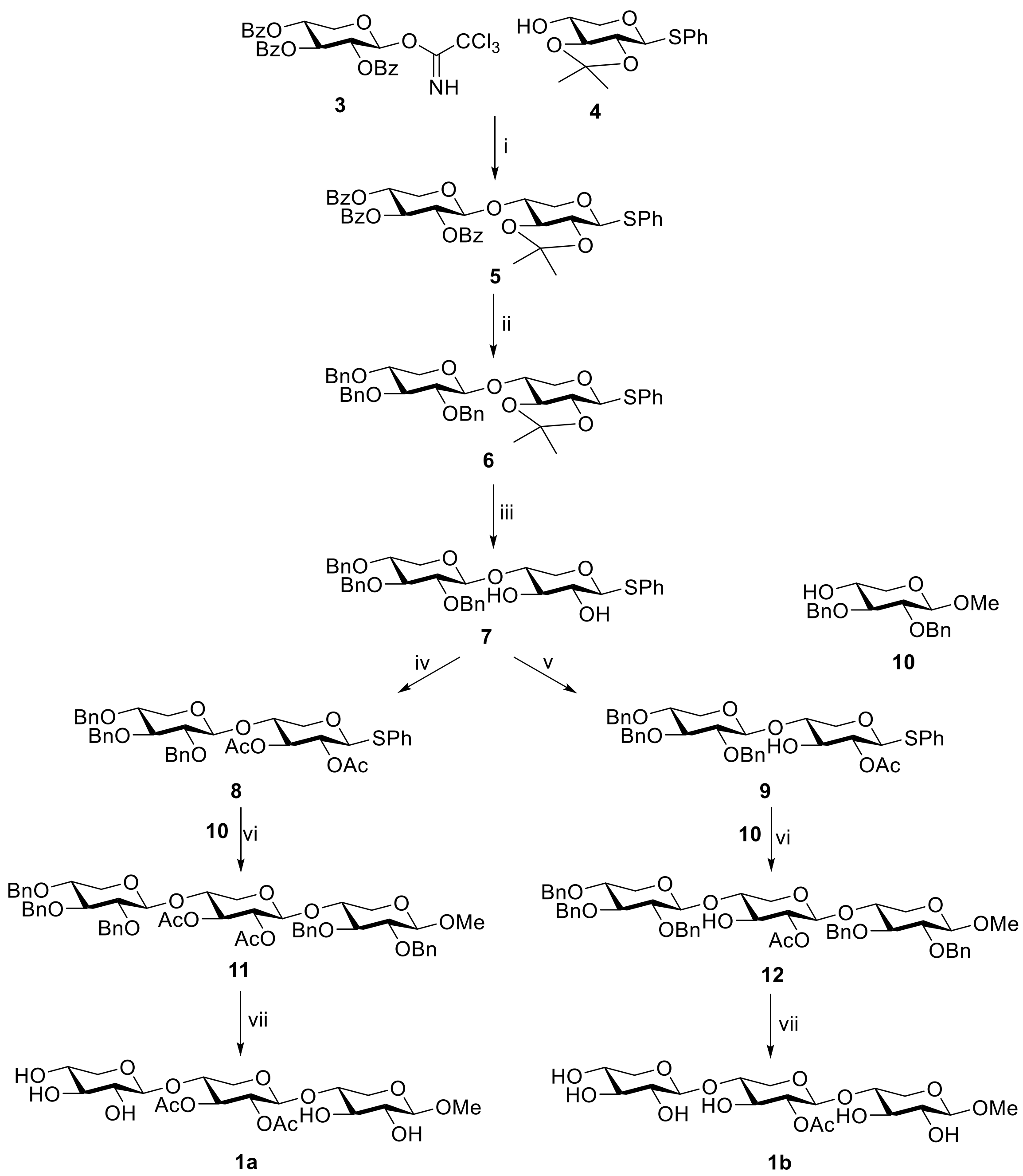

Scheme S1. Preparation of the xylan trisaccharides. Reagents and conditions: (i) TMSOTf, CH_2Cl_2, -40 °C, 10 min, (69%); (ii) 1) NaOMe, MeOH, r.t., 1.5 h, 2) NaH, BnBr, DMF, 0 °C, 2 h, (51%); (iii) TFA, CH_2Cl_2:MeOH 1:3, r.t., 20 h, (62%); (iv) Ac_2O, pyridine, 19 h, r.t. (90%); (v) Ac_2O, pyridine, 19 h, r.t. (73%); (vi) **10**, NIS, TMSOTf, CH_2Cl_2, -40 °C, 30 min (**11**: 57%, **12**: 71%); (vii) Pd/C, MeOH/AcOH, 4 h (**1a**: 87%, **1b**: 82%).

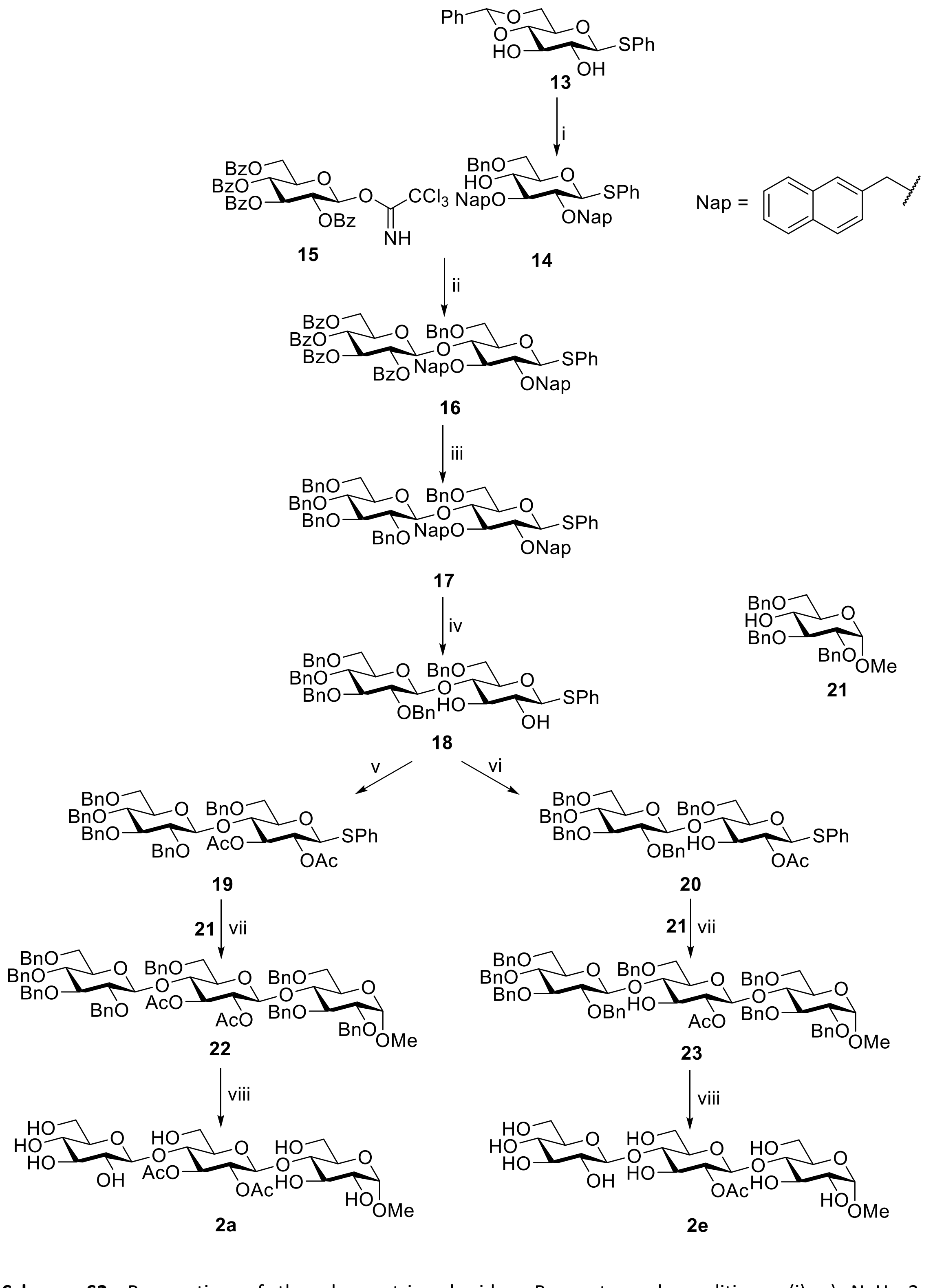

Scheme S2. Preparation of the glucan trisaccharides. Reagents and conditions: (i) a) NaH, 2-(bromomethyl) naphthalene, DMF 0°C → r.t. b) Et_3SiH, BF_3 OEt_2, CH_2Cl_2, 0 °C, (37%); (ii) TMSOTf, CH_2Cl_2, -40 °C, 10 min, (69%); (iii) 1) NaOMe, MeOH, r.t., 17 h, 2) NaH, BnBr, DMF, 0 °C → r.t., 24 h, (81%); (iv) DDQ, CH_2Cl_2:MeOH:H_2O 90:4:4, 0 °C → r.t., 1 h, (68%); (v) Ac_2O, pyridine, 19 h, r.t. (50%); (vi) Ac_2O, pyridine, 19 h, 0 °C → r.t. (65%); (vii) **21**, NIS, TMSOTf, CH_2Cl_2, -40 °C, 30 min (**22**: 62%, **23**: 64%); (viii) Pd/C, MeOH/AcOH, 4 h (**2a**: 95%, **2e**: 90%).

Experimental procedures

General. For following the migration process, a Bruker Avance-III spectrometer operating at 500.20 MHz (^{1}H) and 125.78 MHz (^{13}C) equipped with a Smartprobe: BB/1H was used, while for identification and characterization of the new compounds, a Bruker Avance-III spectrometer operating at 500.20 MHz (^{1}H) and 125.78 MHz (^{13}C) equipped with a Prodigy BBO CryoProbe was employed. The characterizations were performed using a standard set of 1D and 2D NMR spectroscopic techniques: ^{1}H, ^{13}C{^{1}H}, 1D-TOCSY, DQF-COSY, Multiplicity edited HSQC (CH and CH_3 positive, CH_2 negative, both coupled and decoupled), and HMBC. The reported signals are referenced to an internal standard (TMS δ_H = 0.0 ppm, δ_C = 0.0 ppm) or solvent signal (MeOH δ_H = 3.31 ppm, δ_C = 49.00 ppm, $CDCl_3$ δ_H = 7.26 ppm, δ_C = 77.16 ppm). Chemical shifts are reported with two decimals for ^{1}H and one decimal for ^{13}C. Where this is not sufficient for distinguishing two signals, an additional decimal is given. Coupling constants are reported in Hz with one decimal and mentioned only the first time they are encountered. Accurate coupling constants and shifts were extracted from the ^{1}H NMR spectra using the NMR simulation software ChemAdder/SpinAdder.[8] HRMS were recorded on a Bruker daltonics micro-ToF with ESI in positive mode as the ionization source. TLC analysis was performed on Merck silica gel 60 F254 plates and the spots were visualized with UV light and charring with H_2SO_4/MeOH (1:4) and heating. All reactions were monitored by TLC. Column chromatography was carried out using silica gel 60 (0.040 – 0.060 mm) as the stationary phase and as hexane:EtOAc or toluene:EtOAc as the eluent. All chemicals were purchased from Sigma-Aldrich and used as received. Anhydrous dichloromethane was obtained by distillation from a suspension of CaH_2 under argon. Anhydrous DMF was purchased and used as received. Reactions sensitive towards moisture and manipulations of air sensitive reagents were carried out under argon atmosphere.

Standard reaction procedure for glycosylation using trichloroacetimidate donor. To a solution of the donor (1 equivalent) and acceptor (0.75 equivalent) in anhydrous CH_2Cl_2 (1 ml/70 mg donor) were added 4 Å molecular sieves under stirring. After 15-30 min, the reaction mixture was cooled to –40 °C and TMSOTf (0.15 equivalent) was added. After 10 min, the reaction was quenched with Et_3N and warmed to room temperature and the solvent evaporated. The product was purified using column chromatography.

Standard reaction procedure for glycosylation using thiophenyl donor. To a solution of the donor (1 equivalent) and acceptor (1.2 equivalents) in anhydrous CH_2Cl_2 (1 ml/30 mg donor) were added 4 Å molecular sieves. After 15-30 min, the reaction mixture was cooled to –50 °C

and NIS (1.2 equivalents) was added. Next, the reaction mixture was warmed to –40 °C and TMSOTf (0.3 equivalent) was added. After 1 h, the reaction was quenched with Et_3N and warmed to room temperature. The reaction mixture was diluted with CH_2Cl_2 and washed with 20% Na_2SO_4 solution, saturated $NaHCO_3$ solution and saturated NaCl solution. The organic phase was dried over Na_2SO_4, filtered and evaporated. The product was purified using column chromatography.

Standard reaction procedures for hydrogenolysis of benzyl- and benzylidene protecting groups. To a solution of the substrate in MeOH with 30% AcOH (1 ml/20 mg substrate) was added Pd/C 10% w/w (2 weight equivalents). The reaction mixture was stirred under 4 bar H_2-gas in an autoclave reactor for 4 h, after which the mixture was filtered and the solvent evaporated.

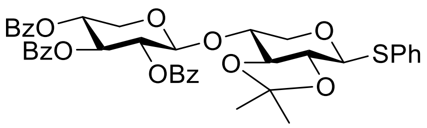

Thiophenyl *O*-(2,3,4-tri-*O*-benzoyl-β-D-xylopyranosyl)-(1→4)-2,3-*O*-propylidene-β-D-xylopyranoside (5): Prepared from donor **3** (1510 mg) and acceptor **4** (530 mg) according to the standard reaction procedure for glycosylation using trichloroacetimidate donor. The crude product was purified by column chromatography (hexane:EtOAc 3:1) to provide **5** as a white foam. Yield: 940 mg (69%), R_f = 0.26. ^{1}H NMR (500.20 MHz, $CDCl_3$, 25 °C): δ = 8.04 – 7.27 (m, 20 H, aromatic H), 5.71 (dd, 1 H, $J_{H-2',H-3'}$ = 6.4 Hz, , $J_{H-3',H-4'}$ = 6.6 Hz, H-3′), 5.30 (dd, 1 H, $J_{H-1',H-'2}$ = 4.8 Hz, H-2′), 5.27 (ddd, 1 H, $J_{H-4',H-5'a}$ = 4.0 Hz, $J_{H-4',H-5'b}$ = 6.2 Hz, H-4′), 4.95 (d, 1 H, H-1′), 4.76 (d, 1 H, $J_{H-1,H-2}$ = 9.5 Hz, H-1), 4.55 (dd, 1 H, $J_{H-5'a,H-5'b}$ = –12.5 Hz, H-5′a), 4.12 (dd, 1 H, $J_{H-5a,H-5b}$ = –11.9 Hz, H-5a), 4.05 (ddd, 1 H, $J_{H-3,H-4}$ = 9.4 Hz, $J_{H-4,H-5a}$ = 5.1 Hz, $J_{H-4,H-5b}$ = 8.9 Hz, H-4), 3.73 (dd, 1 H, H-5′b), 3.68 (dd, 1 H, $J_{H-2,H-3}$ = 9.0 Hz, H-3), 3.24 (dd, 1 H, H-2), 3.22 (dd, 1 H, H-5b), 1.50 (s, 3 H, 2,3-OC(CH_3)$_2$), 1.45 (s, 3 H, 2,3-OC(CH_3)$_2$) ppm. ^{13}C{^{1}H} NMR (125.8 MHz, $CDCl_3$, 25 °C): δ = 165.7 (4′-*C*OPh), 165.4 (3′-*C*OPh), 165.2 (2′-*C*OPh), 133.5 – 128.4 (aromatic C), 111.4 (2,3-O*C*(CH_3)$_2$), 98.1 (C-1′), 85.3 (C-1), 80.7 (C-3), 75.4 (C-2), 74.3 (C-4), 69.7 (C-2′, C-3′), 68.8 (C-4′), 67.7 (C-5), 61.0 (C-5′), 26.9 (2,3-OC(*C*H_3)$_2$), 26.8 (2,3-OC(*C*H_3)$_2$) ppm. HRMS (ESI) m/z: $[M + K]^+$ calculated for $C_{40}H_{38}O_{11}SK$ 765.1772, found 765.1780.

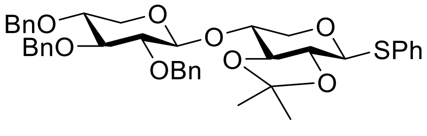

Thiophenyl *O*-(2,3,4-tri-*O*-benzyl-β-D-xylopyranosyl)-(1→4)-2,3-*O*-propylidene-β-D-xylopyranoside (6): To a solution of **5** (480 mg, 1 equivalent) in MeOH (10 ml) at room temperature was added 5.4 M NaOMe in MeOH (37 µl, 0.3 equivalent) . After 1.5 h, DOWEX 50 WX8 H^+ form was added and after 15 min the mixture was filtered and the solvent evaporated. The crude mixture was dissolved in DMF (5 ml), cooled to 0 °C, whereafter 60 % NaH dispersed in mineral oil (160 mg, 6 equivalents) and BnBr (480 µl, 6 equivalents) were added.

After 1.5 h, the reaction was quenched by the addition of MeOH, followed by evaporation of the solvent after an additional 15 min. The crude product was purified by column chromatography (hexane:EtOAc 4:1) to provide **6** as a clear oil. Yield: 230 mg (51%), R_f = 0.29. ^{1}H NMR (500.20 MHz, $CDCl_3$, 25 °C): δ = 7.58 – 7.27 (m, 20 H, aromatic H), 4.84 (d, 1 H, *J* = -10.9 Hz, 3′-OC*H₂*Ph), 4.82 (d, 1 H, 3′-OC*H₂*Ph), 4.790 (d, 1 H, $J_{H\text{-}1,H\text{-}2}$ = 9.5 Hz, H-1), 4.786 (d, 1 H, *J* = -11.0 Hz, 2′-OC*H₂*Ph), 4.71 (d, 1 H, 2′-OC*H₂*Ph), 4.70 (d, 1 H, *J* = -11.6 Hz, 4′-OC*H₂*Ph), 4.60 (d, 1 H, 4′-OC*H₂*Ph), 4.37 (d, 1 H, $J_{H\text{-}1',H\text{-}'2}$ = 7.7 Hz, H-1′), 4.16 (dd, 1 H, $J_{H\text{-}4,H\text{-}5a}$ = 5.0 Hz, $J_{H\text{-}5a,H\text{-}5b}$ = –12.0 Hz, H-5a), 4.00 (ddd, 1 H, $J_{H\text{-}3,H\text{-}4}$ = 9.3 Hz, $J_{H\text{-}4,H\text{-}5b}$ = 8.7 Hz, H-4), 3.92 (dd, 1 H, $J_{H\text{-}4',H\text{-}5'a}$ = 5.3 Hz, $J_{H\text{-}5'a,H\text{-}5'b}$ = –11.7 Hz, H-5′a), 3.64 (dd, 1 H, $J_{H\text{-}2,H\text{-}3}$ = 9.0 Hz, H-3), 3.61 (ddd, 1 H, $J_{H\text{-}3',H\text{-}4'}$ = 8.9 Hz, $J_{H\text{-}4',H\text{-}5'b}$ = 10.3 Hz, H-4′), 3.54 (dd, 1 H, $J_{H\text{-}2',H\text{-}3'}$ = 9.2 Hz, H-3′), 3.37 (dd, 1 H, H-2′), 3.25 (dd, 1 H, H-5b), 3.21 (dd, 1 H, H-2), 3.16 (dd, 1 H, H-5′b), 1.48 (s, 3 H, 2,3-OC(C*H₃*)₂), 1.43 (s, 3 H, 2,3-OC(C*H₃*)₂) ppm. ^{13}C{^{1}H} NMR (125.8 MHz, $CDCl_3$, 25 °C): δ = 138.7 – 127.8 (aromatic C), 111.5 (2,3-O*C*(CH₃)₂), 102.4 (C-1′), 85.3 (C-1), 84.1 (C-3′), 81.7 (C-2′), 80.7 (C-3), 77.7 (C-4′), 75.8 (3′-O*C*H₂Ph), 75.4 (2′-O*C*H₂Ph), 75.3 (C-2), 75.0 (C-4), 73.5 (4′-O*C*H₂Ph), 67.8 (C-5′), 64.2 (C-5), 26.9 (2,3-OC(*C*H₃)₂), 26.6 (2,3-OC(*C*H₃)₂) ppm. HRMS (ESI) m/z: [M + K]$^+$ calculated for $C_{40}H_{44}O_8SK$ 723.2394; Found 723.2397.

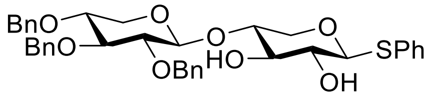

Thiophenyl *O*-(2,3,4-tri-*O*-benzyl-β-D-xylopyranosyl)-(1→4)-β-D-xyloopyranoside (7): To a solution of **6** (285 mg, 1 equivalent) in MeOH:CH_2Cl_2 3:1 (8 ml) was added TFA (32 µl, 1 equivalent). After 20 h, the reaction was quenched with Et_3N and the solvent evaporated. The crude product was purified by column chromatography (hexane:EtOAc 1:1) to provide **7** as a white solid. Yield: 165 mg (62%), R_f= 0.28. ^{1}H NMR (500.20 MHz, $CDCl_3$, 25 °C): δ = 7.56 – 7.25 (m, 20 H, aromatic H), 4.86 (d, 1 H, *J* = -11.1 Hz, 3′-OC*H₂*Ph), 4.84 (d, 1 H, 3′-OC*H₂*Ph), 4.74 (d, 1 H, *J* = -10.9 Hz, 2′-OC*H₂*Ph), 4.72 (d, 1 H, *J* = -11.6 Hz, 4′-OC*H₂*Ph), 4.70 (d, 1 H, 2′-OC*H₂*Ph), 4.61 (d, 1 H, 4′-OC*H₂*Ph), 4.51 (d, 1 H, $J_{H\text{-}1,H\text{-}2}$ = 9.5 Hz, H-1), 4.35 (d, 1 H, $J_{H\text{-}1',H\text{-}'2}$ = 7.7 Hz, H-1′), 4.07 (dd, 1 H, $J_{H\text{-}4,H\text{-}5a}$ = 5.2 Hz, $J_{H\text{-}5a,H\text{-}5b}$ = –11.6 Hz, H-5a), 4.03 (d, 1H, $J_{H\text{-}3,3\text{-}OH}$ = 0.9 Hz, 3-OH), 3.95 (dd, 1 H, $J_{H\text{-}4',H\text{-}5'a}$ = 5.4 Hz, $J_{H\text{-}5'a,H\text{-}5'b}$ = –11.7 Hz, H-5′a), 3.62 (ddd, 1 H, $J_{H\text{-}3',H\text{-}4'}$ = 8.9 Hz, $J_{H\text{-}4',H\text{-}5'b}$ = 10.4 Hz, H-4′), 3.57 (ddd, 1 H, $J_{H\text{-}3,H\text{-}4}$ = 8.7 Hz, $J_{H\text{-}4,H\text{-}5b}$ = 10.2 Hz, H-4), 3.560 (ddd, 1 H, $J_{H\text{-}2,H\text{-}3}$ = 8.8 Hz, H-3), 3.558 (dd, 1 H, $J_{H\text{-}2',H\text{-}3'}$ = 9.2 Hz, H-3′), 3.37 (ddd, 1 H, $J_{H\text{-}2,2\text{-}OH}$ = 2.1 Hz, H-2), 3.35 (dd, 1 H, H-5b), 3.34 (dd, 1 H, H-2′), 3.24 (dd, 1 H, H-5′b), 2.65 (d, 1H, 2-OH) ppm. ^{13}C{^{1}H} NMR (125.8 MHz, $CDCl_3$, 25 °C): δ = 138.6 – 127.8 (aromatic C), 104.4 (C-1′), 88.4 (C-1), 83.8 (C-3′), 81.6 (C-2′), 80.1 (C-4), 77.6 (C-4′), 76.3 (C-3), 75.9 (3′-O*C*H₂Ph), 75.4 (2′-O*C*H₂Ph), 73.7 (4′-O*C*H₂Ph), 71.9 (C-2), 67.5 (C-5), 64.2 (C-5′) ppm. HRMS (ESI) m/z: [M + K]$^+$ calculated for $C_{37}H_{40}O_8SK$ 683.2081; Found 683.2087.

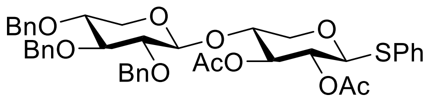

Thiophenyl *O*-(2,3,4-tri-*O*-benzyl-β-D-xylopyranosyl)-(1→4)-2,3-di-*O*-acetyl-β-D-xylopyranoside (8): To a solution of **7** (80 mg, 1 equivalent) in pyridine (2 ml) was added Ac_2O (70 µl, 6 equivalents) under stirring. After 66 h, MeOH (200 µl) was added and the mixture was diluted with CH_2Cl_2 (40 ml) and washed with saturated $NaHCO_3$ solution (20 ml), 1 M HCl solution (20 ml) and saturated NaCl solution (20 ml). The organic phase was dried over Na_2SO_4, filtered and evaporated. The crude product was purified by column chromatography (hexane:EtOAc 2:1) to provide **8** as a white foam. Yield: 81 mg (90%), R_f = 0.41. ^{1}H NMR (500.20 MHz, $CDCl_3$, 25 °C): δ = 7.47 – 7.25 (m, 20 H, aromatic H), 5.15 (dd, 1 H, $J_{H-1,H-2}$ = 8.7Hz, $J_{H-2,H-3}$ = 8.3 Hz, H-2), 4.89 (dd, 1 H, $J_{H-3,H-4}$ = 8.7 Hz, H-3), 4.83 (d, 1 H, *J* = -12.7 Hz, 3′-OCH_2Ph), 4.82 (d, 1 H, 3′-OCH_2Ph), 4.76 (d, 1 H, *J* = -11.0 Hz, 2′-OCH_2Ph), 4.74 (d, 1 H, H-1), 4.70 (d, 1 H, *J* = -11.6 Hz, 4′-OCH_2Ph), 4.69 (d, 1 H, 2′-OCH_2Ph), 4.60 (d, 1 H, 4′-OCH_2Ph), 4.31 (d, 1 H, $J_{H-1′,H-′2}$ = 7.5 Hz, H-1′), 4.18 (dd, 1 H, $J_{H-4,H-5a}$ = 4.8 Hz, $J_{H-5a,H-5b}$ = –11.9 Hz, H-5a), 3.86 (dd, 1 H, $J_{H-4′,H-5′a}$ = 5.3 Hz, $J_{H-5′a,H-5′b}$ = –11.7 Hz, H-5′a), 3.82 (ddd, 1 H, $J_{H-4,H-5b}$ = 9.3 Hz, H-4), 3.56 (ddd, 1 H, $J_{H-3′,H-4′}$ = 8.9 Hz, $J_{H-4′,H-5′b}$ = 9.9 Hz, H-4′), 3.53 (dd, 1 H, $J_{H-2′,H-3′}$ = 9.1 Hz, H-3′), 3.26 (dd, 1 H, H-5b), 3.21 (dd, 1 H, H-2′), 3.17 (dd, 1 H, H-5′b), 2.07 (s, 3 H, 3-COCH_3), 2.02 (s, 3 H, 2-COCH_3) ppm. ^{13}C{^{1}H} NMR (125.8 MHz, $CDCl_3$, 25 °C): δ = 170.1 (2-*C*OCH$_3$), 169.6 (3-*C*OCH$_3$), 138.7 – 127.8 (aromatic C), 103.7 (C-1′), 86.5 (C-1), 83.8 (C-3′), 81.8 (C-2′), 77.9 (C-4′), 75.6 (3′-O*C*H$_2$Ph), 75.3 (2′-O*C*H$_2$Ph), 75.1 (C-4), 73.5 (4′-O*C*H$_2$Ph), 73.4 (C-2), 70.1 (C-3), 66.5 (C-5), 64.1 (C-5′), 21.03 (3-CO*C*H$_3$), 20.99 (2-CO*C*H$_3$) ppm. HRMS (ESI) m/z: $[M + Na]^+$ calculated for $C_{41}H_{44}O_{10}SNa$ 751.2553; Found 751.2575.

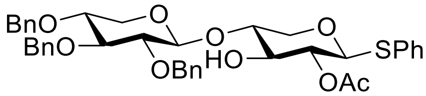

Thiophenyl *O*-(2,3,4-tri-*O*-benzyl-β-D-xyloopyranosyl)-(1→4)-2-*O*-acetyl-β-D-xylopyranoside (9): To a solution of **7** (20 mg, 1 equivalent) in pyridine (0.5 ml) was added Ac_2O (3.2 µl, 1.1 equivalents) under stirring. After 20 h the mixture was diluted with CH_2Cl_2 (15 ml) and washed with saturated $NaHCO_3$ solution (10 ml), 1 M HCl solution (10 ml) and saturated NaCl solution (10 ml). The organic phase was dried over Na_2SO_4, filtered and evaporated. The crude product was purified by column chromatography (hexane:EtOAc 2:1) to provide **9** as a clear oil Yield: 16 mg (73%), R_f = 0.25. ^{1}H NMR (500.20 MHz, $CDCl_3$, 25 °C): δ = 7.49 – 7.26 (m, 20 H, aromatic H), 4.87 (dd, 1 H, $J_{H-1,H-2}$ = 9.9 Hz, $J_{H-2,H-3}$ = 9.5 Hz, H-2), 4.86 (d, 1 H, *J* = -10.9 Hz, 3′-OCH_2Ph), 4.84 (d, 1 H, 3′-OCH_2Ph), 4.74 (d, 1 H, *J* = -10.9 Hz, 2′-OCH_2Ph), 4.72 (d, 1 H, *J* = -11.6 Hz, 4′-OCH_2Ph), 4.69 (d, 1 H, 2′-OCH_2Ph), 4.61 (d, 1 H, H-1), 4.59 (d, 1 H, 4′-OCH_2Ph), 4.35 (d, 1 H, $J_{H-1′,H-′2}$ = 7.8 Hz, H-1′), 4.17 (d, 1 H, $J_{H-3,3-OH}$ = 0.9 Hz, 3-OH), 4.08 (dd, 1 H, $J_{H-4,H-5a}$ = 5.3 Hz, $J_{H-5a,H-5b}$ = –11.6 Hz, H-5a), 3.87 (dd, 1 H, $J_{H-4′,H-5′a}$ = 5.3 Hz, $J_{H-5′a,H-5′b}$ = –11.7 Hz, H-5′a), 3.64 (ddd, 1 H, $J_{H-3,H-4}$ = 9.6 Hz, H-3), 3.63 (ddd, 1 H, $J_{H-4,H-5b}$ = 10.1 Hz, H-4), 3.59 (ddd, 1 H, $J_{H-3′,H-4′}$ = 8.9 Hz, $J_{H-4′,H-5′b}$ = 10.4 Hz, H-4′), 3.55 (dd, 1 H,

$J_{H\text{-}2',H\text{-}3'}$ = 9.1 Hz, H-3′), 3.35 (dd, 1 H, H-5b), 3.33 (dd, 1 H, H-2′), 3.31 (dd, 1 H, H-5′b), 2.16 (s, 3 H, 2-COC*H₃*) ppm. $^{13}C\{^1H\}$ NMR (125.8 MHz, $CDCl_3$, 25 °C): δ = 169.9 (2-*C*OCH₃), 138.5 – 127.9 (aromatic C), 104.6 (C-1′), 86.7 (C-1), 83.8 (C-3′), 81.6 (C-2′), 80.9 (C-3), 77.5 (C-4′), 75.9 (3′-O*C*H₂Ph), 75.4 (2′-O*C*H₂Ph), 75.0 (C-4), 73.7 (4′-O*C*H₂Ph), 71.6 (C-2), 67.5 (C-5), 64.2 (C-5′), 21.2 (2-COCH₃) ppm. HRMS (ESI) m/z: $[M + K]^+$ calculated for $C_{39}H_{42}O_9SK$ 725.2187; Found 725.2191.

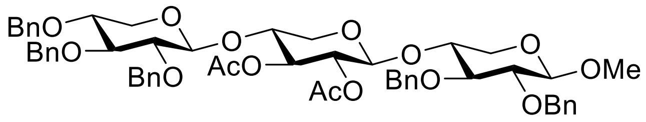

Methyl *O*-(2,3,4-tri-*O*-benzyl-β-D-xylopyranosyl)-(1→4)-*O*-(2,3-di-*O*-acetyl-β-D-xylopyranosyl)-(1→4)-2,3-di-*O*-benzyl-β-D-xylopyranoside (11): Prepared from donor **8** (32 mg) and acceptor **10** (18 mg) according to the standard reaction procedure for glycosylation using thiophenyl donor. The crude product was purified by column chromatography (toluene:EtOAc 7:1) to provide **11** as a clear oil. Yield: 17 mg (57%), R_f = 0.26. ^1H NMR (500.20 MHz, $CDCl_3$, 25 °C): δ = 7.27 – 7.06 (m, 25 H, aromatic H), 5.04 (dd, 1 H, $J_{H\text{-}2',H\text{-}3'}$ = 8.8 Hz, $J_{H\text{-}3',H\text{-}4'}$ = 8.3 Hz, H-3′), 4.80 (d, 1 H, *J* = –10.8 Hz, 3-OC*H₂*Ph), 4.763 (d, 1 H, *J* = –12.0 Hz, 3″-OC*H₂*Ph), 4.762 (dd, 1 H, $J_{H\text{-}1',H\text{-}2'}$ = 7.0 Hz, H-2′), 4.757 (d, 1 H, *J* = –11.1 Hz, 2″-OC*H₂*Ph), 4.756 (d, 1 H, 3″-OC*H₂*Ph), 4.68 (d, 1 H, 3-OC*H₂*Ph), 4.67 (d, 1 H, *J* = –11.0 Hz, 2-OC*H₂*Ph), 4.63 (d, 1 H, *J* = –11.6 Hz, 4″-OC*H₂*Ph), 4.62 (d, 1 H, 2″-OC*H₂*Ph), 4.60 (d, 1 H, 2-OC*H₂*Ph), 4.53 (d, 1 H, 4″-OC*H₂*Ph), 4.46 (d, 1 H, H-1′), 4.17 (d, 1 H, $J_{H\text{-}1'',H\text{-}2''}$ = 7.5 Hz, H-1″), 4.16 (d, 1 H, $J_{H\text{-}1,H\text{-}2}$ = 7.6 Hz, H-1), 3.06 (dd, 1 H, $J_{H\text{-}4',H\text{-}5'a}$ = 4.8 Hz, $J_{H\text{-}5'a,H\text{-}5'b}$ = –12.1 Hz, H-5′a), 3.84 (dd, 1 H, $J_{H\text{-}4,H\text{-}5a}$ = 5.4 Hz, $J_{H\text{-}5a,H\text{-}5b}$ = –11.7 Hz, H-5a), 3.79 (dd, 1 H, $J_{H\text{-}4'',H\text{-}5''a}$ = 5.2 Hz, $J_{H\text{-}5''a,H\text{-}5''b}$ = –11.7 Hz, H-5″a), 3.761 (ddd, 1 H, $J_{H\text{-}3,H\text{-}4}$ = 8.8 Hz, $J_{H\text{-}4,H\text{-}5b}$ = 10.1 Hz, H-4), 3.760 (ddd, 1 H, $J_{H\text{-}4',H\text{-}5'b}$ = 9.6 Hz, H-4′), 3.50 (ddd, 1 H, $J_{H\text{-}3'',H\text{-}4''}$ = 9.1 Hz, $J_{H\text{-}4'',H\text{-}5''b}$ = 9.8 Hz, H-4″), 3.67 (dd, 1 H, $J_{H\text{-}2'',H\text{-}3''}$ = 9.1 Hz, H-3″), 3.46 (s, 3 H, 1-OC*H₃*), 3.43 (dd, 1 H, $J_{H\text{-}2,H\text{-}3}$ = 9.2 Hz, H-3), 3.25 (dd, 1 H, H-2), 3.21 (dd, 1 H, H-2″), 3.19 (dd, 1 H, H-5′b), 3.11 (dd, 1 H, H-5″b), 3.07 (dd, 1 H, H-5b), 1.95 (s, 3 H, 2′-OCOC*H₃*), 1.93 (s, 3 H, 3′-OCOC*H₃*) ppm. $^{13}C\{^1H\}$ NMR (125.8 MHz, $CDCl_3$, 25 °C): δ = 170.2 (3′-O*C*OCH₃), 169.6 (2′-O*C*OCH₃), 138.7 – 127.7 (aromatic C), 105.4 (C-1), 103.8 (C-1″), 100.4 (C-1′), 83.8 (C-3″), 82.2 (C-3), 81.8 (C-2″), 81.7 (C-2), 77.9 (C-4″), 77.5 (C-4), 75.5 (3″-O*C*H₂Ph), 75.5 (3-O*C*H₂Ph), 75.4 (C-4′), 75.3 (2-O*C*H₂Ph), 75.0 (2″-O*C*H₂Ph), 73.5 (4″-O*C*H₂Ph), 72.6 (C-3′), 71.5 (C-2′), 64.1 (C-5″), 63.3 (C-5), 63.2 (C-5′), 57.2 (1-O*C*H₃), 21.0 (3′-OCO*C*H₃), 20.9 (2′-OCO*C*H₃) ppm. HRMS (ESI) m/z: $[M + Na]^+$ calculated for $C_{55}H_{62}O_{15}Na$ 985.3986; Found 985.3994.

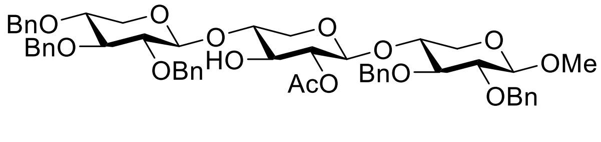

Methyl *O*-(2,3,4-tri-*O*-benzyl-β-D-xylopyranosyl)-(1→4)-*O*-(2,3-di-*O*-acetyl-β-D-xylopyranosyl)-(1→4)-2,3-di-*O*-benzyl-β-D-xylopyranoside (12): Prepared from donor **10** (24 mg) and acceptor **9** (18 mg)

according to the standard reaction procedure for glycosylation using thiophenyl donor. The crude product was purified by column chromatography (hexane:EtOAc 3:2 and toluene:EtOAc 4:1) to provide **12** as a clear oil. Yield: 23 mg (71%), R_f = 0.19 (hexane:EtOAc 3:2). ^{1}H NMR (500.20 MHz, $CDCl_3$, 25 °C): δ = 7.36 – 7.21 (m, 25 H, aromatic H), 4.873 (d, 1 H, *J* = –10.7Hz, 3-OCH_2Ph), 4.866 (d, 1 H, *J* = –10.4 Hz, 3′′-OCH_2Ph), 4.86 (d, 1 H, 3′′-OCH_2Ph), 4.834 (d, 1 H, *J* = –11.2 Hz, 2′′-OCH_2Ph), 4.827 (dd, 1 H, $J_{H-1',H-2'}$ = 7.6 Hz, $J_{H-2',H-3'}$ = 9.4 Hz, H-2′), 4.73 (d, 1 H, *J* = –10.7 Hz, 2-OCH_2Ph), 4.75(d, 1 H, 3-OCH_2Ph), 4.72 (d, 1 H, *J* = –11.7 Hz, 4′′-OCH_2Ph), 4.70 (d, 1 H, 2′′-OCH_2Ph), 4.69 (d, 1 H, 2-OCH_2Ph), 4.60 (d, 1 H, 4′′-OCH_2Ph), 4.46 (d, 1 H, H-1′), 4.35 (d, 1 H, $J_{H-1'',H-2''}$ = 7.8 Hz, H-1′′), 4.24 (d, 1 H, $J_{H-1,H-2}$ = 7.6 Hz, H-1), 4.12 (d, 1 H, $J_{H-3',3'-OH}$ = 1.3 Hz, 3′-OH), 3.97 (dd, 1 H, $J_{H-4',H-5'a}$ = 5.5 Hz, $J_{H-5'a,H-5'b}$ = –11.9 Hz, H-5′a), 3.91 (dd, 1 H, $J_{H-4,H-5a}$ = 5.4 Hz, $J_{H-5a,H-5b}$ = –11.7 Hz, H-5a), 3.88 (dd, 1 H, $J_{H-4'',H-5''a}$ = 5.4 Hz, $J_{H-5''a,H-5''b}$ = –11.7 Hz, H-5′′a), 3.81 (ddd, 1 H, $J_{H-3,H-4}$ = 8.8 Hz, $J_{H-4,H-5b}$ = 10.2 Hz, H-4), 3.65 (ddd, 1 H, $J_{H-3',H-4'}$ = 9.5 Hz, $J_{H-4',H-5'b}$ = 9.9 Hz, H-4′), 3.60 (ddd, 1 H, H-3′), 3.59 (ddd, 1 H, $J_{H-3'',H-4''}$ = 8.9 Hz, $J_{H-4'',H-5''b}$ = 10.4 Hz, H-4′′), 3.56 (dd, 1 H, $J_{H-2'',H-3''}$ = 9.1 Hz, H-3′′), 3.53 (s, 3 H, 1-OCH_3), 3.51 (dd, 1 H, $J_{H-2,H-3}$ = 9.2 Hz, H-3), 3.33 (dd, 1 H, H-2′′), 3.32 (dd, 1 H, H-2), 3.25 (dd, 1 H, H-5′b), 3.22 (dd, 1 H, H-5′′b), 3.16 (dd, 1 H, H-5b), 2.11 (s, 3 H, 2′-OCOCH_3) ppm. ^{13}C{^{1}H} NMR (125.8 MHz, $CDCl_3$, 25 °C): δ = 169.8 (2′-O*C*OPh), 138.7 – 127.6 (aromatic C), 105.3 (C-1), 104.7 (C-1′′), 101.1 (C-1′), 83.8 (C-3′′), 82.2 (C-3), 81.7 (C-2), 81.6 (C-2′′), 81.0 (C-4′), 77.9 (C-4), 77.5 (C-4′′), 75.9 (3′′-O*C*H_2Ph), 75.6 (2-O*C*H_2Ph), 75.5 (3-O*C*H_2Ph), 75.0 (2′′-O*C*H_2Ph), 73.7 (4′′-O*C*H_2Ph), 73.3 (C-3′), 73.1 (C-2′), 64.2 (C-5′′), 63.7 (C-5′), 63.4 (C-5), 57.3 (1-O*C*H_3), 21.1 (2′-OCO*C*H_3) ppm. HRMS (ESI) m/z: $[M + Na]^+$ calculated for $C_{53}H_{60}O_{14}Na$ 943.3881; Found 943.3893.

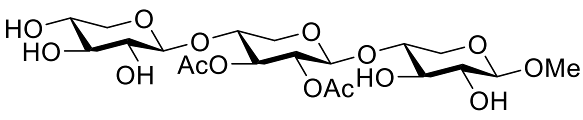

Methyl *O*-(β-D-xylopyranosyl)-(1→4)-*O*-(2,3-di-*O*-acetyl-β-D-xylopyranosyl)-(1→4)-β-D-xylopyranoside (1a): Compound **1a** (30 mg) was prepared according to the standard reaction procedures for hydrogenolysis of benzyl- and benzylidene protecting groups from **11**. Yield: 14 mg (87%). ^{1}H NMR (500.20 MHz, MeOD, 25 °C): δ = 5.11 (dd, 1 H, $J_{H-2',H-3'}$ = 9.5 Hz, $J_{H-3',H-4'}$ = 9.0 Hz, H-3′), 4.80 (dd, 1 H, $J_{H-1',H-2'}$ = 7.6 Hz, H-2′), 4.67 (d, 1 H, H-1′), 4.24 (d, 1 H, $J_{H-1'',H-2''}$ = 7.6 Hz, H-1′′), 4.14 (dd, 1 H, $J_{H-4',H-5'a}$ = 5.7 Hz, $J_{H-5'a,H-5'b}$ = –12.0 Hz, H-5′a), 4.12 (d, 1 H, $J_{H-1,H-2}$ = 7.3 Hz, H-1), 3.96 (dd, 1 H, $J_{H-4,H-5a}$ = 5.2 Hz, $J_{H-5a,H-5b}$ = –11.6 Hz, H-5a), 3.89 (ddd, 1 H, $J_{H-4',H-5'b}$ = 10.1 Hz, H-4′), 3.82 (dd, 1 H, $J_{H-4'',H-5''a}$ = 5.2 Hz, $J_{H-5''a,H-5''b}$ = –11.6 Hz, H-5′′a), 3.62 (ddd, 1 H, $J_{H-3,H-4}$ = 8.5 Hz, $J_{H-4,H-5b}$ = 10.1 Hz, H-4), 3.47 (s, 3 H, 1-OCH_3), 3.445 (dd, 1 H, H-5′b), 3.442 (ddd, 1 H, $J_{H-3'',H-4''}$ = 8.7 Hz, $J_{H-4'',H-5''b}$ = 10.1 Hz, H-4′′), 3.40 (dd, 1 H, $J_{H-2,H-3}$ = 8.9 Hz, H-3), 3.27 (dd, 1 H, $J_{H-2'',H-3''}$ = 9.2 Hz, H-3′′), 3.189 (dd, 1 H, H-5′′b), 3.188 (dd, 1 H, H-5b), 3.17 (dd, 1 H, H-2), 3.07 (dd, 1 H, H-2′′), 2.02 (s, 3 H, 2′-OCOCH_3), 2.00 (s, 3 H, 3′-OCOCH_3) ppm. ^{13}C{^{1}H} NMR (125.8 MHz, MeOD, 25 °C): δ = 172.1 (3′-O*C*OCH$_3$), 171.4 (2′-O*C*OCH$_3$), 105.9

(C-1), 104.8 (C-1″), 101.3 (C-1′), 78.8 (C-4), 77.8 (C-3″), 76.6 (C-4′), 75.5 (C-3), 74.7 (C-2″), 74.6 (C-2), 74.5 (C-3′), 73.0 (C-2′), 71.1 (C-4″), 67.1 (C-5″), 64.6 (C-5′), 64.1 (C-5), 57.2 (1-O*C*H_3), 21.0 (3′-OCO*C*H_3), 20.7 (2′-OCO*C*H_3) ppm. HRMS (ESI) m/z: $[M + K]^+$ calculated for $C_{20}H_{32}O_{15}K$ 551.1378; Found 551.1356.

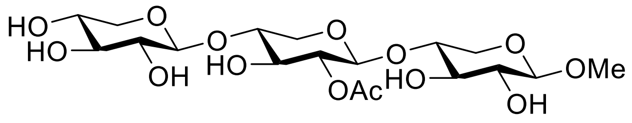

Methyl *O*-(β-D-xylopyranosyl)-(1→4)-*O*-(2-*O*-acetyl-β-D-xylopyranosyl)-(1→4)-β-D-xylopyranoside (1b): Compound **1b** (19 mg) was prepared according to the standard reaction procedures for hydrogenolysis of benzyl- and benzylidene protecting groups from **12**. Yield: 8.0 mg (82%). ^1H NMR (500.20 MHz, MeOD, 25 °C): δ = 4.71 (dd, 1 H, $J_{H\text{-}1',H\text{-}2'}$ = 7.6 Hz, $J_{H\text{-}2',H\text{-}3'}$ = 9.2 Hz, H-2′), 4.54 (d, 1 H, H-1′), 4.33 (d, 1 H, $J_{H\text{-}1'',H\text{-}2''}$ = 7.6 Hz, H-1″), 4.13 (d, 1 H, $J_{H\text{-}1,H\text{-}2}$ = 7.3 Hz, H-1), 4.09 (dd, 1 H, $J_{H\text{-}4',H\text{-}5'a}$ = 5.3 Hz, $J_{H\text{-}5'a,H\text{-}5'b}$ = –11.8 Hz, H-5′a), 3.94 (dd, 1 H, $J_{H\text{-}4,H\text{-}5a}$ = 5.1 Hz, $J_{H\text{-}5a,H\text{-}5b}$ = –11.7 Hz, H-5a), 3.88 (dd, 1 H, $J_{H\text{-}4'',H\text{-}5''a}$ = 5.3 Hz, $J_{H\text{-}5''a,H\text{-}5''b}$ = –11.5 Hz, H-5″a), 3.74 (ddd, 1 H, $J_{H\text{-}4',H\text{-}5'b}$ = 9.9 Hz, H-4′), 3.63 (dd, 1 H, $J_{H\text{-}3',H\text{-}4'}$ = 8.7 Hz, H-3′), 3.59 (ddd, 1 H, $J_{H\text{-}3,H\text{-}4}$ = 8.5 Hz, $J_{H\text{-}4,H\text{-}5b}$ = 9.8 Hz, H-4), 3.49 (ddd, 1 H, $J_{H\text{-}3'',H\text{-}4''}$ = 8.9 Hz, $J_{H\text{-}4'',H\text{-}5''b}$ = 10.2 Hz, H-4″), 3.47 (s, 3 H, 1-OC*H_3*), 3.40 (dd, 1 H, $J_{H\text{-}2,H\text{-}3}$ = 8.9 Hz, H-3), 3.37 (dd, 1 H, H-5′b), 3.31 (dd, 1 H, $J_{H\text{-}2'',H\text{-}3''}$ = 9.1 Hz, H-3″), 3.23 (dd, 1 H, H-5″b), 3.20 (dd, 1 H, H-2″), 3.19 (dd, 1 H, H-5b), 3.17 (dd, 1 H, H-2), 2.08 (s, 3 H, 2′-OCOC*H_3*) ppm. $^{13}C\{^1H\}$ NMR (125.8 MHz, MeOD, 25 °C): δ = 171.8 (2′-O*C*OCH_3), 105.8 (C-1), 104.1 (C-1″), 101.6(C-1′), 78.9 (C-4), 77.9 (C-4′), 77.7 (C-3″), 75.5 (C-3), 74.8 (C-2′), 74.5 (C-2), 74.4 (C-2″), 73.8 (C-3′), 71.1 (C-4″), 67.1 (C-5″), 64.6 (C-5′), 64.0 (C-5), 57.2 (1-O*C*H_3), 21.0 (2′-OCO*C*H_3) ppm. HRMS (ESI) m/z: $[M + K]^+$ calculated for $C_{18}H_{30}O_{14}K$ 509.1273; Found 509.1238.

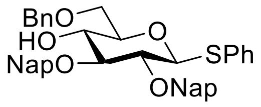

Thiophenyl 6-*O*-benzyl-2,3-di-*O*-2-naphthylmethyl-β-D-glucopyranoside (14): To a solution of **13** (340 mg, 1 equivalent) in DMF (1.5 ml), cooled to 0 °C, were added 60 % NaH dispersed in mineral oil (150 mg, 4 equivalents) and NapBr (620 mg, 3 equivalents). The reaction was slowly brought to room temperature over 17 h, after which the reaction mixture was quenched by the addition of MeOH. After an additional 15 min, the reaction mixture was diluted with CH_2Cl_2 (100 ml) and washed with saturated $NaHCO_3$ solution (80 ml) and saturated NaCl solution (80 ml). The organic phase was dried over Na_2SO_4, filtered and evaporated. The crude product was dissolved in CH_2Cl_2 (5 ml) and cooled to 0 °C. Next, Et_3SiH (1.5 ml, equivalents) and BF_3 OEt_2 (190 µl, equivalents) were added. After 3 h, the mixture was diluted with CH_2Cl_2 (100 ml) and washed with saturated $NaHCO_3$ solution (80 ml) and saturated NaCl solution (80 ml). The organic phase was dried over Na_2SO_4, filtered and evaporated. The crude product was purified by column chromatography

(hexane:EtOAc 3:1) to provide **14** as a white powder. Yield: 220 mg (37%), R_f = 0.20. ^{1}H NMR (500.20 MHz, $CDCl_3$, 25 °C): δ = 7.85 – 7.25 (m, 24 H, aromatic H), 5.10 (d, 1 H, *J* = -10.6 Hz, 2-OC*H*$_2$Ar), 5.08 (d, 1 H, *J* = -11.7 Hz, 3-OC*H*$_2$Ar), 4.99 (d, 1 H, 3-OC*H*$_2$Ar), 4.92 (d, 1 H, 2-OC*H*$_2$Ar), 4.76 (d, 1 H, $J_{\text{H-1,H-2}}$ = 9.7 Hz, H-1), 4.61 (d, 1 H, *J* = -11.9 Hz, 6-OC*H*$_2$Ph), 4.58 (d, 1 H, 6-OC*H*$_2$Ph), 3.81 (dd, 1 H, $J_{\text{H-5,H-6a}}$ = 4.2 Hz, $J_{\text{H-6a,H-6b}}$ = -10.5 Hz, H-6a), 3.79 (dd, 1 H, $J_{\text{H-5,H-6b}}$ = 5.27 Hz,H-6b), 3.74 (ddd, 1 H, $J_{\text{H-3,H-4}}$ = 9.0 Hz, $J_{\text{H-4,H-5}}$ = 9.6 Hz, $J_{\text{H-4,4-OH}}$ = 2.4 Hz, H-4), 3.64 (dd, 1 H, $J_{\text{H-2,H-3}}$ = 8.8 Hz, H-3), 3.59 (dd, 1 H, H-2), 3.52 (dd, 1 H, H-5), 2.64 (d, 1 H, 4-OH) ppm. ^{13}C{^{1}H} NMR (125.78 MHz, $CDCl_3$, 25 °C): δ = 138.0 – 125.9 (aromatic C), 87.9 (C-1), 86.3 (C-3), 80.7 (C-2), 78.2 (C-5), 75.7 (3-*C*H$_2$Ar), 75.6 (2-*C*H$_2$Ar), 73.8 (6-*C*H$_2$Ph), 72.1 (C-4), 70.6 (C-6) ppm. HRMS (ESI) m/z: [M + Na]$^+$ calculated for $C_{41}H_{38}O_5SNa$ 665.2338; Found 665.2308.

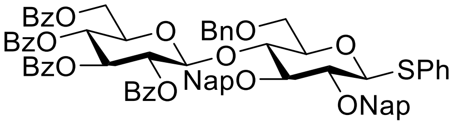

Thiophenyl *O*-(2,3,4,6-tetra-*O*-benzoyl-β-D-glucopyranosyl)-(1→4)-6-*O*-benzyl-2,3-*O*-2-naphthylmethyl-β-D-glucopyranoside (16): Prepared from donor **15** (720 mg) and acceptor **14** (470 mg) according to the standard reaction procedure for glycosylation using trichloroacetimidate donor. The crude product was purified by column chromatography (toluene:EtOAc 16:1) to provide **16** as a white foam. Yield: 590 mg (67%), R_f = 0.32. ^{1}H NMR (500.20 MHz, $CDCl_3$, 25 °C): δ = 7.93 – 7.13 (m, 44 H, aromatic H), 5.73 (dd, 1 H, $J_{\text{H-2',H-3'}}$ = 9.8 Hz, $J_{\text{H-3',H-4'}}$ = 9.5 Hz, H-3'), 5.59 (dd, 1 H, $J_{\text{H-4',H-5'}}$ = 9.9 Hz, H-4'), 5.52 (dd, 1 H, $J_{\text{H-1',H-'2}}$ = 8.1 Hz, H-2'), 5.32 (d, 1 H, *J* = -11.6 Hz, 3-OC*H*$_2$Ar), 5.05 (d, 1 H, H-1'), 4.90 (d, 1 H, 3-OC*H*$_2$Ar), 4.87 (d, 1 H, *J* = -10.5 Hz, 2-OC*H*$_2$Ar), 4.78 (d, 1 H, 2-OC*H*$_2$Ar), 4.72 (d, 1 H, *J* = -11.9 Hz, 6-OC*H*$_2$Ph), 4.59 (d, 1 H, $J_{\text{H-1,H-2}}$ = 9.8 Hz, H-1), 4.461 (dd, 1 H, $J_{\text{H-6'a,H-5'}}$ = 3.3 Hz, $J_{\text{H-6'a,H-6'b}}$ = -12.1 Hz, H-6'a), 4.455 (d, 1 H, 6-OC*H*$_2$Ph), 4.32 (dd, 1 H, $J_{\text{H-6'b,H-5'}}$ = 4.8 Hz, H-6'b), 4.14 (dd, 1 H, $J_{\text{H-3,H-4}}$ = 9.0 Hz, $J_{\text{H-4,H-5}}$ = 9.8 Hz, H-4), 3.79 (ddd, 1 H, H-5'), 3.74 (dd, 1 H, $J_{\text{H-2,H-3}}$ = 8.7 Hz, H-3), 3.71 (dd, 1 H, $J_{\text{H-6a,H-5}}$ = 3.5 Hz, $J_{\text{H-6a,H-6b}}$ = -11.0 Hz, H-6a), 3.63 (dd, 1 H, $J_{\text{H-6b,H-5}}$ = 1.6 Hz, H-6b), 3.48 (dd, 1 H, H-2), 3.28 (ddd, 1 H, H-5) ppm. ^{13}C{^{1}H} NMR (125.8 MHz, $CDCl_3$, 25 °C): δ = 166.1 (6'-*C*OPh), 165.9 (3'-*C*OPh), 165.2 (4'-*C*OPh), 165.0 (2'-*C*OPh), 138.3 – 125.4 (aromatic C), 100.6 (C-1'), 87.7 (C-1), 84.9 (C-3), 80.4 (C-2), 78.7 (C-5), 77.4 (C-4), 75.8 (3-*C*H$_2$Ar), 75.6 (2-*C*H$_2$Ar), 73.7 (6-*C*H$_2$Ph), 73.3 (C-3'), 72.4 (C-2'), 72.2 (C-5'), 69.8 (C-4'), 68.1 (C-6), 63.0 (C-6') ppm. HRMS (ESI) m/z: [M + NH_4]$^+$ calculated for $C_{75}H_{68}O_{14}SN$ 1238.4355; Found 1238.4328.

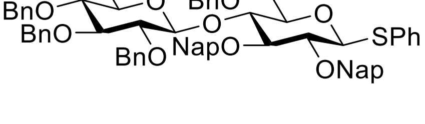

Thiophenyl *O*-(2,3,4,6-tetra-*O*-benzyl-β-D-glucopyranosyl)-(1→4)-6-*O*-benzyl-2,3-*O*-2-naphthylmethyl-β-D-glucopyranoside (17): To a solution of **16** (580 mg, 1 equivalent) in MeOH (10 ml) at room temperature was added 5.4 M NaOMe in

MeOH (35 µl, 0.4 equivalent). After 17 h, DOWEX 50 WX8 H^+ form was added and after 15 min the mixture was filtered and the solvent evaporated. The crude mixture was dissolved in DMF (8 ml), cooled to 0 °C, followed by the addition of 60 % NaH dispersed in mineral oil (115 mg, 6 equivalents) and BnBr (340 µl, 6 equivalents). The reaction mixture was slowly brought to room temperature over 17 h. The reaction was quench by the addition of MeOH and after an additional 15 min the mixture was diluted with CH_2Cl_2 (100 ml) and washed with saturated $NaHCO_3$ solution (80 ml) and saturated NaCl solution (80 ml). The organic phase was dried over Na_2SO_4, filtered and evaporated. The crude product was purified by column chromatography (toluene:EtOAc 16:1) to provide **17** as a white solid. Yield: 450 mg (81%), R_f = 0.41. ^{1}H NMR (500.20 MHz, $CDCl_3$, 25 °C): δ = 7.80 – 7.12 (m, 44 H, aromatic H), 5.30 (d, 1 H, *J* = -11.7 Hz, 3-OCH_2Ar), 4.93 (d, 1 H, *J* = -10.5 Hz, 2-OCH_2Ar), 4.889 (d, 1 H, 3-OCH_2Ar), 4.887 (d, 1 H, *J* = -11.0 Hz, 3′-OCH_2Ph), 4.86 (d, 1 H, 2-OCH_2Ar), 4.80 (d, 1 H, 3′-OCH_2Ph), 4.789 (d, 1 H, *J* = -11.3 Hz, 2′-OCH_2Ph), 4.787 (d, 1 H, *J* = -10.8 Hz, 4′-OCH_2Ph), 4.72 (d, 1 H, 2′-OCH_2Ph), 4.68 (d, 1 H, $J_{H\text{-}1,H\text{-}2}$ = 9.8 Hz, H-1), 4.57 (d, 1 H, *J* = -11.9 Hz, 6-OCH_2Ph), 4.55 (d, 1 H, $J_{H\text{-}1',H\text{-}'2}$ = 8.0 Hz, H-1′), 4.53 (d, 1 H, 4′-OCH_2Ph), 4.48 (d, 1 H, 6-OCH_2Ph), 4.315 (d, 1 H, *J* = -12.0 Hz, 6′-OCH_2Ph), 4.313 (d, 1 H, 6′-OCH_2Ph), 4.09 (dd, 1 H, $J_{H\text{-}3,H\text{-}4}$ = 8.9 Hz, $J_{H\text{-}4,H\text{-}5}$ = 9.8 Hz, H-4), 3.86 (dd, 1 H, $J_{H\text{-}6a,H\text{-}5}$ = 4.1 Hz, $J_{H\text{-}6a,H\text{-}6b}$ = -10.9 Hz, H-6a), 3.77 (dd, 1 H, $J_{H\text{-}6b,H\text{-}5}$ = 1.9 Hz, H-6b), 3.73 (dd, 1 H, $J_{H\text{-}2,H\text{-}3}$ = 8.8 Hz, H-3), 3.67 (dd, 1 H, $J_{H\text{-}6'a,H\text{-}5'}$ = 1.9 Hz, $J_{H\text{-}6'a,H\text{-}6'b}$ = -11.1 Hz, H-6′a), 3.63 (dd, 1 H, $J_{H\text{-}3',H\text{-}4'}$ = 9.4 Hz, $J_{H\text{-}4',H\text{-}5'}$ = 9.4 Hz, H-4′), 3.57 (dd, 1 H, $J_{H\text{-}2',H\text{-}3'}$ = 9.1 Hz, H-3′), 3.545 (dd, 1 H, H-2), 3.544 (dd, 1 H, $J_{H\text{-}6'b,H\text{-}5'}$ = 4.3 Hz, H-6′b), 3.41 (ddd, 1 H, H-5), 3.39 (dd, 1 H, H-2′), 3.34 (ddd, 1 H, H-5′) ppm. $^{13}C\{^1H\}$ NMR (125.8 MHz, $CDCl_3$, 25 °C): δ = 138.8 – 125.7 (aromatic C), 102.8 (C-1′), 87.6 (C-1), 85.2 (C-3), 85.1 (C-3′), 82.9(C-2′), 80.4 (C-2), 79.5 (C-5), 78.2 (C-4′), 76.7 (C-4), 75.8 (3′-*C*H$_2$Ph), 75.7 (2-*C*H$_2$Ar), 75.5 (3-*C*H$_2$Ar), 75.2 (C-5′), 75.1 (2′-*C*H$_2$Ph), 75.0 (4′-*C*H$_2$Ph), 73.4 (6′-*C*H$_2$Ph, 6-*C*H$_2$Ph), 69.0 (C-6′), 68.4 (C-6) ppm. HRMS (ESI) m/z: $[M + Na]^+$ calculated for $C_{75}H_{72}O_{10}SNa$ 1187.4744; Found 1187.4640.

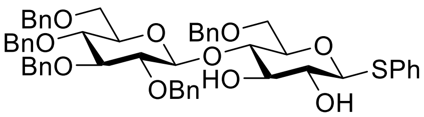

Thiophenyl *O*-(2,3,4,6-tetra-*O*-benzyl-β-D-glucopyranosyl)-(1→4)-6-*O*-benzyl-β-D-glucopyranoside (18): To a solution of **17** (400 mg, 1 equivalent) in CH_2Cl_2 (5 ml), MeOH (0.4 ml) and H_2O (0.4 ml), cooled to 0 °C, was slowly added DDQ dissolved in CH_2Cl_2 (4 ml). The reaction mixture was brought to room temperature over 1 h. After additional 1 h, the reaction mixture was diluted with CH_2Cl_2 (100 ml) and washed with saturated $NaHCO_3$ solution (80 ml), H_2O (80 ml) and saturated NaCl solution (80 ml). The organic phase was dried over Na_2SO_4, filtered and evaporated. The crude product was purified by column chromatography (hexane:EtOAc 3:2) to provide **20** as a white solid. Yield: 205 mg (68%), R_f = 0.29. ^{1}H NMR (500.20 MHz, $CDCl_3$, 25 °C): δ = 7.60 – 7.12 (m, 30 H, aromatic H), 4.89 (d, 1 H, *J* = -11.0

Hz, 3′-OCH_2Ph), 4.82 (d, 1 H, 3′-OCH_2Ph), 4.792 (d, 1 H, *J* = -11.6 Hz, 2′-OCH_2Ph), 4.785 (d, 1 H, *J* = -11.0 Hz, 4′-OCH_2Ph), 4.78 (d, 1 H, 2′-OCH_2Ph), 4.58 (d, 1 H, $J_{H\text{-}1,H\text{-}2}$ = 9.4 Hz, H-1), 4.55 (d, 1 H, *J* = -11.9 Hz, 6′-OCH_2Ph), 4.50 (d, 1 H, 4′-OCH_2Ph), 4.48 (d, 1 H, 6′-OCH_2Ph), 4.37 (d, 1 H, $J_{H\text{-}1',H\text{-}'2}$ = 8.0 Hz, H-1′), 4.33 (d, 1 H, *J* = -12.0 Hz, 6-OCH_2Ph), 4.31 (d, 1 H, 6-OCH_2Ph), 3.73 (dd, 1 H, $J_{H\text{-}6a,H\text{-}5}$ = 1.5 Hz, $J_{H\text{-}6a,H\text{-}6b}$ = -11.0 Hz, H-6a), 3.67 (dd, 1 H, $J_{H\text{-}6'a,H\text{-}5'}$ = 2.0 Hz, $J_{H\text{-}6'a,H\text{-}6'b}$ = -10.7 Hz, H-6′a), 3.64 (dd, 1 H, $J_{H\text{-}6b,H\text{-}5}$ = 5.2 Hz, H-6b), 3.63 (ddd, 1 H, $J_{H\text{-}2,H\text{-}3}$ = 8.8 Hz, $J_{H\text{-}3,H\text{-}4}$ = 8.7 Hz, $J_{H\text{-}3,3\text{-}OH}$ = 0.7 Hz, H-3), 3.62 (dd, 1 H, $J_{H\text{-}2',H\text{-}3'}$ = 9.3 Hz, $J_{H\text{-}3',H\text{-}4'}$ = 9.0 Hz, H-3′), 3.59 (ddd, 1 H, $J_{H\text{-}4,H\text{-}5}$ = 9.4 Hz, H-5), 3.58 (dd, 1 H, $J_{H\text{-}6'b,H\text{-}5'}$ = 5.7 Hz, H-6′b), 3.56 (dd, 1 H, $J_{H\text{-}4',H\text{-}5'}$ = 9.9 Hz, H-4′), 3.54 (dd, 1 H, H-4), 3.50 (ddd, 1 H, H-5′), 3.43 (ddd, 1 H, $J_{H\text{-}2,2\text{-}OH}$ = 1.7 Hz, H-2), 3.42 (dd, 1 H, H-2′), 2.60 (d, 1 H, 2-O*H*), 1.56 (d, 1 H, 3-O*H*) ppm. $^{13}C\{^1H\}$ NMR (125.8 MHz, $CDCl_3$, 25 °C): δ = 138.5 – 127.6 (aromatic C), 103.4 (C-1′), 87.2 (C-1), 84.8 (C-3′), 81.9(C-2′), 80.7 (C-4), 78.6 (C-5), 77.8 (C-4′), 76.5 (C-3), 75.9 (3′-*C*H_2Ph), 75.3 (2′-*C*H_2Ph), 75.2 (4′-*C*H_2Ph), 74.6 (C-5′), 73.8 (6′-*C*H_2Ph), 73.2 (6-*C*H_2Ph), 72.1 (C-2), 68.93 (C-6), 68.86 (C-6′) ppm. HRMS (ESI) m/z: $[M + Na]^+$ calculated for $C_{53}H_{56}O_{10}SNa$ 907.3492 Found 907.3445.

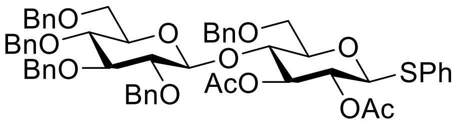

Thiophenyl *O*-(2,3,4,6-tetra-*O*-benzyl-β-D-glucopyranosyl)-(1→4)-2,3-di-*O*-acetyl-6-*O*-benzyl-β-D-glucopyranoside (19): To a solution of **18** (67mg, 1 equivalent) in pyridine (1.5 ml) was added Ac_2O (43 µl, 6 equivalents). After 24 h, the reaction was quenched with MeOH and diluted with CH_2Cl_2 (20 ml) and washed with saturated $NaHCO_3$ solution (15 ml), 1 M HCl solution (15 ml) and saturated NaCl solution (15 ml). The organic phase was dried over Na_2SO_4, filtered and evaporated. The crude product was purified by column chromatography (hexane:EtOAc 3:1) to provide **19** as a white solid. Yield: 37 mg (50%), R_f = 0.29. ^1H NMR (500.20 MHz, $CDCl_3$, 25 °C): δ = 7.52 – 7.12 (m, 30 H, aromatic H), 5.18 (dd, 1 H, $J_{H\text{-}2,H\text{-}3}$ = 9.5 Hz, $J_{H\text{-}3,H\text{-}4}$ = 9.4 Hz, H-3), 4.97 (dd, 1 H, $J_{H\text{-}1,H\text{-}2}$ = 10.0 Hz, H-2), 4.87 (d, 1 H, *J* = -11.1 Hz, 3′-OCH_2Ph), 4.78 (d, 1 H, *J* = -11.0 Hz, 4′-OCH_2Ph), 4.77 (d, 1 H, 3′-OCH_2Ph), 4.76 (d, 1 H, *J* = -11.2 Hz, 2′-OCH_2Ph), 4.69 (d, 1 H, 2′-OCH_2Ph), 4.67 (d, 1 H, H-1), 4.514 (d, 1 H, 4′-OCH_2Ph), 4.510 (d, 1 H, *J* = -12.0 Hz, 6-OCH_2Ph), 4.48 (d, 1 H, *J* = -11.9 Hz, 6′-OCH_2Ph), 4.43 (d, 1 H, 6′-OCH_2Ph), 4.42 (d, 1 H, 6-OCH_2Ph), 4.32 (d, 1 H, $J_{H\text{-}1',H\text{-}'2}$ = 8.0 Hz, H-1′), 3.91 (dd, 1 H, $J_{H\text{-}4,H\text{-}5}$ = 9.9 Hz, H-4), 3.78 (dd, 1 H, $J_{H\text{-}6a,H\text{-}5}$ = 4.1 Hz, $J_{H\text{-}6a,H\text{-}6b}$ = -11.2 Hz, H-6a), 3.707 (dd, 1 H, $J_{H\text{-}6'a,H\text{-}5'}$ = 1.8 Hz, $J_{H\text{-}6'a,H\text{-}6'b}$ = -10.5 Hz, H-6′a), 3.706 (dd, 1 H, $J_{H\text{-}6b,H\text{-}5}$ = 1.6 Hz, H-6b), 3.68 (dd, 1 H, $J_{H\text{-}6'b,H\text{-}5'}$ = 3.9 Hz, H-6′b), 3.63 (dd, 1 H, $J_{H\text{-}3',H\text{-}4'}$ = 9.0 Hz, $J_{H\text{-}4',H\text{-}5'}$ = 9.8 Hz, H-4′), 3.51 (dd, 1 H, $J_{H\text{-}2',H\text{-}3'}$ = 9.2 Hz, H-3′), 3.48 (ddd, 1 H, H-5), 3.30 (dd, 1 H, H-2′), 3.26 (ddd, 1 H, H-5′), 2.08 (s, 3 H, 2-COCH_3), 1.96 (s, 3 H, 3-COCH_3) ppm. $^{13}C\{^1H\}$ NMR (125.8 MHz, $CDCl_3$, 25 °C): δ = 170.9 (3-*C*OCH_3), 169.6 (2-*C*OCH_3), 138.8 – 127.7 (aromatic C), 102.9 (C-1′), 85.9 (C-1), 84.9 (C-3′), 82.5 (C-2′), 79.5 (C-5), 77.7 (C-4′), 75.6 (3′-

CH2Ph), 75.2 (2′-CH2Ph), 75.0 (4′-CH2Ph), 74.6 (C-4), 74.5 (C-5′), 74.1 (C-3), 73.4 (6-CH2Ph),73.3 (6′-CH2Ph), 70.3 (C-2), 68.9 (C-6′), 67.9 (C-6), 21.0 (2-COCH3), 20.8 (3-COCH3) ppm. HRMS (ESI) m/z: $[M + Na]^+$ calculated for $C_{57}H_{60}O_{12}SNa$ 991.3703; Found 991.3637.

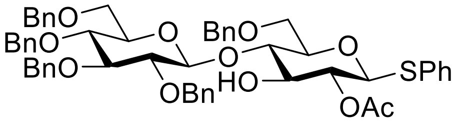

Thiophenyl *O*-(2,3,4,6-tetra-*O*-benzyl-β-D-glucopyranosyl)-(1→4)-*O*-2-*O*-acetyl-6-*O*-benzyl-β-D-glucopyranoside (20): To a solution of **18** (50 mg, 1 equivalent) in dry pyridine (1 ml) was added Ac_2O (8.3 µl, 1,5 equivalents). After 41 h, the reaction was quenched with MeOH and diluted with CH_2Cl_2 (20 ml) and washed with saturated $NaHCO_3$ solution (15 ml), 1 M HCl solution (15 ml) and saturated NaCl solution (15 ml). The organic phase was dried over Na_2SO_4, filtered and evaporated. The crude product was purified by column chromatography (hexane:EtOAc 2:1) to provide **22** as a clear solid. Yield: 35.5 mg (65%), R_f = 0.31. ^1H NMR (500.20 MHz, $CDCl_3$, 25 °C): δ = 7.53 – 7.13 (m, 30 H, aromatic H), 4.96 (dd, 1 H, $J_{H\text{-}1,H\text{-}2}$ = 10.1 Hz, $J_{H\text{-}2,H\text{-}3}$ = 9.1 Hz, H-2), 4.88 (d, 1 H, *J* = -11.0 Hz, 3′-OC*H2*Ph), 4.79 (d, 1 H, *J* = -11.1 Hz, 4′-OC*H2*Ph), 4.81 (d, 1 H, 3′-OC*H2*Ph), 4.79 (d, 1 H, *J* = -11.5 Hz, 2′-OC*H2*Ph), 4.78 (d, 1 H, 2′-OC*H2*Ph), 4.67 (d, 1 H, H-1), 4.52 (d, 1 H, *J* = -11.9 Hz, 6′-OC*H2*Ph), 4.51 (d, 1 H, 4′-OC*H2*Ph), 4.46 (d, 1 H, 6′-OC*H2*Ph), 4.43 (d, 1 H, $J_{H\text{-}3,3\text{-}OH}$ = 1.2 Hz, 3-OH), 4.37 (d, 1 H, $J_{H\text{-}1',H\text{-}'2}$ = 7.9 Hz, H-1′), 4.31 (d, 1 H, *J* = -12.4 Hz, 6-OC*H2*Ph), 4.30 (d, 1 H, 6-OC*H2*Ph), 3.76 (ddd, 1 H, $J_{H\text{-}3,H\text{-}4}$ = 8.4 Hz, H-3), 3.75 (dd, 1 H, $J_{H\text{-}6a,H\text{-}5}$ = 1.2 Hz, $J_{H\text{-}6a,H\text{-}6b}$ = -11.0 Hz, H-6a), 3.67 (dd, 1 H, $J_{H\text{-}6'a,H\text{-}5'}$ = 1.9 Hz, $J_{H\text{-}6'a,H\text{-}6'b}$ = -10.5 Hz, H-6′a), 3.64 (dd, 1 H, $J_{H\text{-}6b,H\text{-}5}$ = 5.3 Hz, H-6b), 3.613 (dd, 1 H, $J_{H\text{-}2',H\text{-}3'}$ = 9.6 Hz, $J_{H\text{-}3',H\text{-}4'}$ = 9.0 Hz, H-3′), 3.606 (dd, 1 H, $J_{H\text{-}4,H\text{-}5}$ = 9.2 Hz, H-4), 3.59 (ddd, 1 H, H-5), 3.58 (dd, 1 H, $J_{H\text{-}6'b,H\text{-}5'}$ = 5.5 Hz, H-6′b), 3.57 (dd, 1 H, $J_{H\text{-}4',H\text{-}5'}$ = 9.8 Hz, H-4′), 3.49 (ddd, 1 H, H-5′), 3.41 (dd, 1 H, H-2′), 2.15 (s, 3 H, 2-COC*H3*) ppm. $^{13}C\{^1H\}$ NMR (125.8 MHz, $CDCl_3$, 25 °C): δ = 169.8 (2-*C*OCH3), 138.5 – 127.6 (aromatic C), 103.4 (C-1′), 85.9 (C-1), 84.8 (C-3′), 82.0 (C-2′), 81.0 (C-4), 78.6 (C-5), 77.8 (C-4′), 75.9 (3′-*C*H2Ph), 75.3 (2′-*C*H2Ph), 75.2 (4′-*C*H2Ph), 74.8 (C-3), 74.7 (C-5′), 73.7 (6′-*C*H2Ph), 73.3 (6-*C*H2Ph), 72.0 (C-2), 68.9 (C-6), 68.7 (C-6′), 21.2 (2-CO*CH3*) ppm. HRMS (ESI) m/z: $[M + Na]^+$ calculated for $C_{55}H_{58}O_{11}SNa$ 949.3598; Found 949.3563.

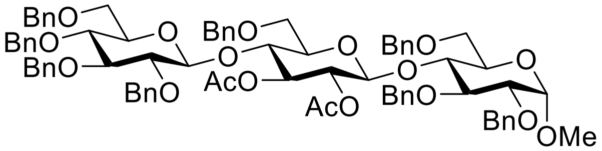

Methyl *O*-(2,3,4,6-tetra-*O*-benzyl-β-D-glucopyranosyl)-(1→4)-*O*-(2,3-di-*O*-acetyl-6-*O*-benzyl-β-D-glucopyranosyl)-(1→4)-2,3,6-tri-*O*-benzyl-α-D-glucopyranoside (22): Prepared from donor **19** (33 mg) and acceptor **21** (19 mg) according to the standard reaction procedure for glycosylation using thiophenyl donor. The crude product was purified by column chromatography (hexane:EtOAc 2:1) to provide **22** as a clear oil. Yield: 28 mg (62%), R_f = 0.33. ^1H NMR (500.20 MHz, $CDCl_3$, 25 °C):

δ = 7.35 – 7.09 (m, 40 H, aromatic H), 5.02 (d, 1 H, J = -11.7 Hz, 3-OCH_2Ph), 4.98 (dd, 1 H, $J_{H\text{-}2,H\text{-}3}$ = 9.8 Hz, $J_{H\text{-}3',H\text{-}4'}$ = 9.4 Hz, H-3′), 4.90 (dd, 1 H, $J_{H\text{-}1',H\text{-}2'}$ = 8.1 Hz, H-2′), 4.83 (d, 1 H, J = -11.0 Hz, 3″-OCH_2Ph), 4.77 (d, 1 H, J = -10.9 Hz, 4″-OCH_2Ph), 4.75 (d, 1 H, 3-OCH_2Ph), 4.735 (d, 1 H, 3″-OCH_2Ph), 4.730 (d, 1 H, J = -11.2 Hz, 2″-OCH_2Ph), 4.72 (d, 1 H, J = -12.2 Hz, 2-OCH_2Ph), 4.70 (d, 1 H, J = -12.1 Hz, 6-OCH_2Ph), 4.63 (d, 1 H, 2″-OCH_2Ph), 4.57 (d, 1 H, 2-OCH_2Ph), 4.56 (d, 1 H, $J_{H\text{-}1,H\text{-}2}$ = 3.5 Hz, H-1), 4.52 (d, 1 H, H-1′), 4.50 (d, 1 H, 4″-OCH_2Ph), 4.48 (d, 1 H, J = -12.0 Hz, 6″-OCH_2Ph), 4.45 (d, 1 H, 6-OCH_2Ph), 4.43 (d, 1 H, 6″-OCH_2Ph), 4.34 (d, 1 H, J = -12.1 Hz, 6′-OCH_2Ph), 4.26 (d, 1 H, $J_{H\text{-}1'',H\text{-}2''}$ = 7.9 Hz, H-1″), 4.25 (d, 1 H, 6′-OCH_2Ph), 3.93 (dd, 1 H, $J_{H\text{-}4',H\text{-}5'}$ = 9.8 Hz, H-4′), 3.86 (dd, 1 H, $J_{H\text{-}3,H\text{-}4}$ = 8.8 Hz, $J_{H\text{-}4,H\text{-}5}$ = 9.5 Hz, H-4), 3.85 (dd, 1 H, $J_{H\text{-}2,H\text{-}3}$ = 9.5 Hz, H-3), 3.77 (dd, 1 H, $J_{H\text{-}6a,H\text{-}5}$ = 1.9 Hz, $J_{H\text{-}6a,H\text{-}6b}$ = -10.8 Hz, H-6a), 3.69 (dd, 1 H, $J_{H\text{-}6''a,H\text{-}5''}$ = 1.5 Hz, $J_{H\text{-}6''a,H\text{-}6''b}$ = -10.5 Hz, H-6″a), 3.66 (dd, 1 H, $J_{H\text{-}6''b,H\text{-}5''}$ = 3.8 Hz, H-6″b), 3.64 (ddd, 1 H, $J_{H\text{-}6b,H\text{-}5}$ = 3.4 Hz, H-5), 3.611 (dd, 1 H, H-6b), 3.609 (dd, 1 H, $J_{H\text{-}3'',H\text{-}4''}$ = 8.9 Hz, $J_{H\text{-}4'',H\text{-}5''}$ = 9.8 Hz, H-4″), 3.58 (dd, 1 H, $J_{H\text{-}6'a,H\text{-}5'}$ = 3.5 Hz, $J_{H\text{-}6'a,H\text{-}6'b}$ = -11.1 Hz, H-6′a), 3.55 (dd, 1 H, $J_{H\text{-}6'b,H\text{-}5'}$ = 1.5 Hz, H-6′b), 3.46 (dd, 1 H, H-2), 3.43 (dd, 1 H, $J_{H\text{-}2'',H\text{-}3''}$ = 9.1 Hz, H-3″), 3.35 (s, 3 H, 1-OCH_3), 3.27 (dd, 1 H, H-2″), 3.18 (ddd, 1 H, H-5″), 3.14 (ddd, 1 H, H-5′), 1.96 (s, 3 H, 2′-COCH_3), 1.95 (s, 3 H, 3′-COCH_3) ppm. ^{13}C{^{1}H} NMR (125.8 MHz, CDCl$_3$, 25 °C): δ = 170.9 (3′-COCH$_3$), 169.5 (2′-COCH$_3$), 139.7 – 127.1 (aromatic C), 102.9 (C-1″), 100.4 (C-1′), 98.5 (C-1), 84.8 (C-3″), 82.5 (C-2″), 80.4 (C-3), 79.2 (C-2), 77.7 (C-4″), 77.4 (C-4), 75.6 (3″-CH$_2$Ph), 75.3 (3-CH$_2$Ph), 75.2 (C-5′), 75.1 (2″-CH$_2$Ph), 74.9 (4″-CH$_2$Ph), 74.6 (C-4′), 74.5 (C-5″), 73.8 (6-CH$_2$Ph), 73.7 (2-CH$_2$Ph), 73.31 (6″-CH$_2$Ph), 73.28 (C-3′), 73.17 (6′-CH$_2$Ph), 72.4 (C-2′), 70.0 (C-5), 68.9 (C-6″), 68.0 (C-6), 67.6 (C-6′), 55.5 (1-OCH$_3$), 21.0 (2′-COCH$_3$), 20.9 (3′-COCH$_3$) ppm. HRMS (ESI) m/z: [M + Na]$^+$ calculated for $C_{79}H_{86}O_{18}Na$ 1345.5712; Found 1345.5618.

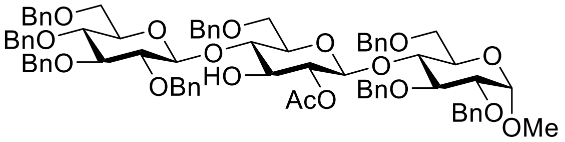

Methyl *O*-(2,3,4,6-tetra-*O*-benzyl-β-D-glucopyranosyl)-(1→4)-*O*-(2-*O*-acetyl-6-*O*-benzyl-β-D-glucopyranosyl)-(1→4)-2,3,6-tri-*O*-benzyl-α-D-glucopyranoside (23): Prepared from donor **20** (34 mg) and acceptor **21** (21 mg) according to the standard reaction procedure for glycosylation using thiophenyl donor. The crude product was purified by column chromatography (hexane:EtOAc 3:2) to provide **23** as a white foam. Yield: 30 mg (64%), R_f = 0.25. ^{1}H NMR (500.20 MHz, CDCl$_3$, 25 °C): δ = 7.36 – 7.12 (m, 40 H, aromatic H), 5.02 (d, 1 H, J = -11.6 Hz, 3-OCH_2Ph), 4.89 (dd, 1 H, $J_{H\text{-}1',H\text{-}2'}$ = 8.2 Hz, $J_{H\text{-}2,H\text{-}3}$ = 9.5 Hz,H-2′), 4.88 (d, 1 H, J = -10.9 Hz, 3″-OCH_2Ph), 4.80 (d, 1 H, 3″-OCH_2Ph), 4.78 (d, 1 H, J = -10.8 Hz, 4″-OCH_2Ph), 4.76 (d, 1 H, 3-OCH_2Ph), 4.75 (d, 1 H, J = -12.1 Hz, 2-OCH_2Ph), 4.725 (d, 1 H, J = -12.0 Hz, 2″-OCH_2Ph), 4.719 (d, 1 H, 2″-OCH_2Ph), 4.69 (d, 1 H, J = -12.1 Hz, 6-OCH_2Ph), 4.59 (d, 1 H, 2-OCH_2Ph), 4.56 (d, 1 H, $J_{H\text{-}1,H\text{-}2}$ = 3.6 Hz, H-1), 4.54 (d, 1 H, H-1′), 4.52 (d, 1 H, J = -11.8 Hz, 6″-OCH_2Ph), 4.50 (d, 1 H, 4″-OCH_2Ph), 4.48 (d, 1 H, 6-OCH_2Ph), 4.46 (d, 1 H, 6″-OCH_2Ph), 4.29 (d,

1 H, $J_{H-1'',H-2''}$ = 7.8 Hz, H-1''), 4.25 (d, 1H, $J_{H-3',3'-OH}$ = 0.9 Hz, 3'-OH), 4.22 (d, 1 H, J = -11.9 Hz, 6'-OCH_2Ph), 4.17 (d, 1 H, 6'-OCH_2Ph), 3.88 (dd, 1 H, $J_{H-3,H-4}$ = 8.8 Hz, $J_{H-4,H-5}$ = 9.5 Hz, H-4), 3.87 (dd, 1 H, $J_{H-2,H-3}$ = 9.6 Hz, H-3), 3.79 (dd, 1 H, $J_{H-6a,H-5}$ = 3.5 Hz, $J_{H-6a,H-6b}$ = -10.8 Hz, H-6a), 3.67 (dd, 1 H, $J_{H-6''a,H-5''}$ = 2.0 Hz, $J_{H-6''a,H-6''b}$ = -10.4 Hz, H-6''a), 3.66 (ddd, 1 H, $J_{H-6b,H-5}$ 1.9 Hz, H-5), 3.64 (dd, 1 H, H-6b), 3.61 (dd, 1 H, $J_{H-3',H-4'}$ = 8.7 Hz, $J_{H-4',H-5'}$ = 9.8 Hz, H-4'), 3.60 (dd, 1 H, $J_{H-6'a,H-5'}$ = 1.2 Hz, $J_{H-6'a,H-6'b}$ = -10.9 Hz, H-6'a), 3.584 (dd, 1 H, $J_{H-6''b,H-5''}$ = 5.0 Hz, H-6''b), 3.577 (dd, 1 H, $J_{H-3'',H-4''}$ = 9.3 Hz, $J_{H-4'',H-5''}$ = 9.8 Hz, H-4''), 3.54 (dd, 1 H, $J_{H-2'',H-3''}$ = 9.4 Hz, H-3''), 3.52 (ddd, 1 H, H-3'), 3.45 (dd, 1 H, H-2), 3.42 (ddd, 1 H, H-5''), 3.42 (dd, 1 H, $J_{H-6'b,H-5'}$ = 4.6 Hz, H-6'b), 3.37 (dd, 1 H, H-2''), 3.36 (s, 3 H, 1-OCH_3), 3.24 (ddd, 1 H, H-5'), 2.04 (s, 3 H, 2'-COCH_3) ppm. ^{13}C{^{1}H} NMR (125.8 MHz, $CDCl_3$, 25 °C): δ = 169.5 (2'-*C*OCH$_3$), 139.7 – 127.1 (aromatic C), 103.4 (C-1''), 100.2 (C-1), 98.6 (C-1'), 84.7 (C-3''), 81.9 (C-2''), 80.8 (C-4'), 80.4 (C-3), 79.2 (C-2), 77.9 (C-4''), 77.7 (C-4), 75.9 (3''-*C*H$_2$Ph), 75.40 (2''-*C*H$_2$Ph), 75.36 (3-*C*H$_2$Ph), 75.1 (4''-*C*H$_2$Ph), 74.9 (C-5'), 74.6 (C-5''), 73.9 (C-2'), 73.74 (2-*C*H$_2$Ph), 73.70 (6-*C*H$_2$Ph), 73.67 (6''-*C*H$_2$Ph), 73.6 (C-3'), 73.2 (6'-*C*H$_2$Ph), 70.0 (C-5), 68.7 (C-6''), 68.5 (C-6'), 68.2 (C-6), 55.4 (1-O*C*H$_3$), 21.2 (2'-CO*C*H$_3$) ppm. HRMS (ESI) m/z: $[M + Na]^+$ calculated for $C_{77}H_{84}O_{17}Na$ 1303.5606; Found 1303.5541.

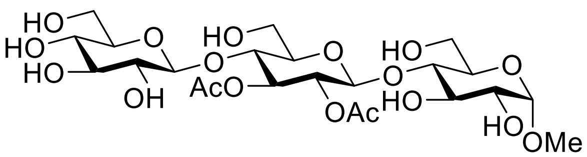

Methyl *O*-(β-D-glucopyranosyl)-(1→4)-*O*-(2,3-di-*O*-acetyl-β-D-glucopyranosyl)-(1→4)-α-D-glucopyranoside (2a): Compound **2a** (25,4 mg) was prepared according to the standard reaction procedures for hydrogenolysis of benzyl- and benzylidene protecting groups from **22**. Yield: 11mg (95%). ^{1}H NMR (500.20 MHz, MeOD, 25 °C): δ = 5.16 (dd, 1 H, $J_{H-2,H-3}$ = 9.8 Hz, $J_{H-3',H-4'}$ = 9.3 Hz, H-3'), 4.89 (dd, 1 H, $J_{H-1',H-2'}$ = 8.1 Hz, H-2'), 4.73 (d, 1 H, H-1'), 4.66 (d, 1 H, $J_{H-1,H-2}$ = 3.4 Hz, H-1), 4.34 (d, 1 H, $J_{H-1'',H-2''}$ = 7.8 Hz, H-1''), 3.949 (dd, 1 H, $J_{H-6'a,H-5'}$ = 2.2 Hz, $J_{H-6'a,H-6'b}$ = -12.2 Hz, H-6'a), 3.946 (dd, 1 H, $J_{H-4',H-5'}$ = 9.9 Hz, H-4'), 3.90 (dd, 1 H, $J_{H-6'b,H-5'}$ = 4.1 Hz, H-6'b), 3.86 (dd, 1 H, $J_{H-6''a,H-5''}$ = 2.0 Hz, $J_{H-6''a,H-6''b}$ = -11.8 Hz, H-6''a), 3.76 (dd, 1 H, $J_{H-6a,H-5}$ = 1.1 Hz, $J_{H-6a,H-6b}$ = -11.8 Hz, H-6a), 3.71 (dd, 1 H, $J_{H-2,H-3}$ = 9.6 Hz, $J_{H-3,H-4}$ = 8.5 Hz, H-3), 3.68 (dd, 1 H, $J_{H-6b,H-5}$ = 3.9 Hz, H-6b), 3.66 (dd, 1 H, $J_{H-6''b,H-5''}$ = 5.2 Hz, H-6''b), 3.57 (ddd, 1 H, H-5'), 3.53 (ddd, 1 H, $J_{H-4,H-5}$ = 9.9 Hz, H-5), 3.52 (dd, 1 H, H-4), 3.44 (dd, 1 H, H-2), 3.39 (s, 3 H, 1-OCH_3), 3.33 (dd, 1 H, $J_{H-2'',H-3''}$ = 9.2 Hz, $J_{H-3'',H-4''}$ = 8.9 Hz, H-3''), 3.26 (ddd, 1 H, H-5''), 3.24 (dd, 1 H, $J_{H-4'',H-5''}$ = 9.9 Hz, H-4''), 3.09 (dd, 1 H, H-2''), 2.06 (s, 3 H, 3'-COCH_3), 2.05 (s, 3 H, 2'-COCH_3) ppm. ^{13}C{^{1}H} NMR (125.8 MHz, MeOD, 25 °C): δ = 172.4 (3'-*C*OCH$_3$), 171.2 (2'-*C*OCH$_3$), 104.3 (C-1'), 101.9 (C-1), 101.0 (C-1''), 80.9 (C-4), 78.0 (C-5''), 77.9 (C-3''), 77.1 (C-5'), 76.2 (C-4'), 75.2 (C-2''), 74.7 (C-3'), 73.3 (C-2', C-3), 73.2 (C-2), 71.9 (C-5), 71.6 (C-4''), 62.8 (C-6''), 61.3 (C-6), 61.0 (C-6'), 55.6 (1-O*C*H$_3$), 21.0 (3'-CO*C*H$_3$), 20.7 (2'-CO*C*H$_3$) ppm. HRMS (ESI) m/z: $[M + Na]^+$ calculated for $C_{23}H_{38}O_{18}Na$ 625.1956; Found 625.1903.

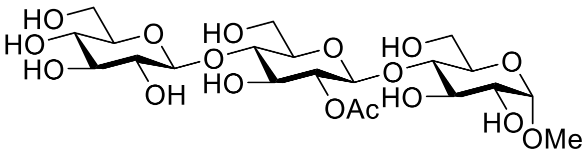

Methyl *O*-(β-D-glucopyranosyl)-(1→4)-*O*-(2-*O*-acetyl-β-D-glucopyranosyl)-(1→4)-α-D-glucopyranoside (2e): Compound **2e** (23 mg) was prepared according to the standard reaction procedures for hydrogenolysis of benzyl- and benzylidene protecting groups from **23**. Yield: 9 mg (90%). ^{1}H NMR (500.20 MHz, MeOD, 25 °C): δ = 4.81 (dd, 1 H, $J_{H\text{-}1',H\text{-}2'}$ = 8.1 Hz, $J_{H\text{-}2,H\text{-}3}$ = 9.8 Hz,H-2′), 4.66 (d, 1 H, $J_{H\text{-}1,H\text{-}2}$ = 3.7 Hz, H-1), 4.61 (d, 1 H, H-1′), 4.40 (d, 1 H, $J_{H\text{-}1'',H\text{-}2''}$ = 7.9 Hz, H-1″), 3.94 (dd, 1 H, $J_{H\text{-}6'a,H\text{-}5'}$ = 2.2 Hz, $J_{H\text{-}6'a,H\text{-}6'b}$ = -12.1 Hz, H-6′a), 3.88 (dd, 1 H, $J_{H\text{-}6''a,H\text{-}5''}$ = 2.2 Hz, $J_{H\text{-}6''a,H\text{-}6''b}$ = -11.8 Hz, H-6″a), 3.86 (dd, 1 H, $J_{H\text{-}6'b,H\text{-}5'}$ = 4.5 Hz, H-6′b), 3.76 (dd, 1 H, $J_{H\text{-}6a,H\text{-}5}$ = 1.7 Hz, $J_{H\text{-}6a,H\text{-}6b}$ = -11.9 Hz, H-6a), 3.71 (dd, 1 H, $J_{H\text{-}2,H\text{-}3}$ = 9.7 Hz, $J_{H\text{-}3,H\text{-}4}$ = 8.5 Hz, H-3), 3.681 (dd, 1 H, $J_{H\text{-}3',H\text{-}4'}$ = 9.4 Hz, $J_{H\text{-}4',H\text{-}5'}$ = 10.0 Hz, H-4′), 3.677 (dd, 1 H, H-3′), 3.67 (dd, 1 H, $J_{H\text{-}6b,H\text{-}5}$ = 4.1 Hz, H-6b), 3.62 (dd, 1 H, $J_{H\text{-}6''b,H\text{-}5''}$ = 6.2 Hz, H-6″b), 3.52 (ddd, 1 H, $J_{H\text{-}4,H\text{-}5}$ = 9.9 Hz, H-5), 3.50 (ddd, 1 H, H-5′), 3.49 (dd, 1 H, H-4), 3.43 (dd, 1 H, H-2), 3.39 (s, 3 H, 1-OC*H₃*), 3.36 (dd, 1 H, $J_{H\text{-}2'',H\text{-}3''}$ = 9.3 Hz, $J_{H\text{-}3'',H\text{-}4''}$ = 9.0 Hz, H-3″), 3.33 (ddd, 1 H, H-5″), 3.26 (dd, 1 H, $J_{H\text{-}4'',H\text{-}5''}$ = 9.8 Hz, H-4″), 3.21 (dd, 1 H, H-2″), 2.11 (s, 3 H, 2′-COCH_3) ppm. ^{13}C{^{1}H} NMR (125.8 MHz, MeOD, 25 °C): δ = 171.6 (2′-*C*OCH$_3$), 104.5 (C-1″), 102.1 (C-1′), 101.0 (C-1), 80.9 (C-4), 79.8 (C-4′), 78.2 (C-5″), 77.9 (C-3″), 76.9 (C-5′), 75.0 (C-2″), 74.8 (C-2′), 74.5 (C-3′), 73.4 (C-3), 73.2 (C-2), 71.9 (C-5), 71.4 (C-4″), 62.5 (C-6″), 61.40 (C-6′), 61.38 (C-6), 55.6 (1-O*C*H$_3$), 21.0 (2′-CO*C*H$_3$) ppm. HRMS (ESI) m/z: [M + Na]$^+$ calculated for $C_{21}H_{36}O_{17}Na$ 583.1850; Found 583.1808.

NMR spectra

Thiophenyl *O*-(2,3,4-tri-*O*-benzoyl-β-D-xylopyranosyl)-(1→4)-2,3-*O*-propylidene-β-D-xylopyranoside (5):

^{1}H NMR (500.20 MHz, $CDCl_3$, 25°C):

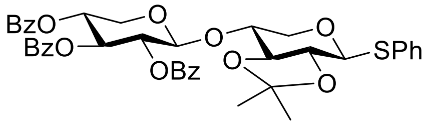

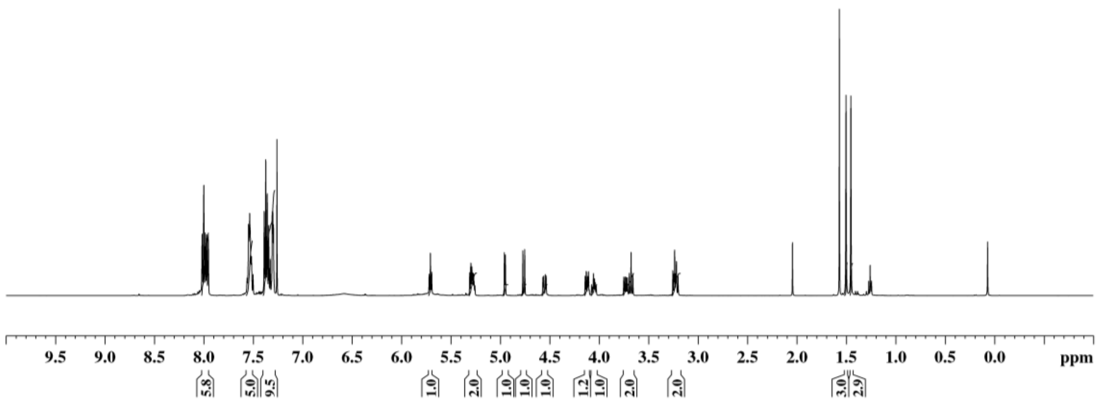

^{13}C{^{1}H} NMR (125.8 MHz, $CDCl_3$, 25°C):

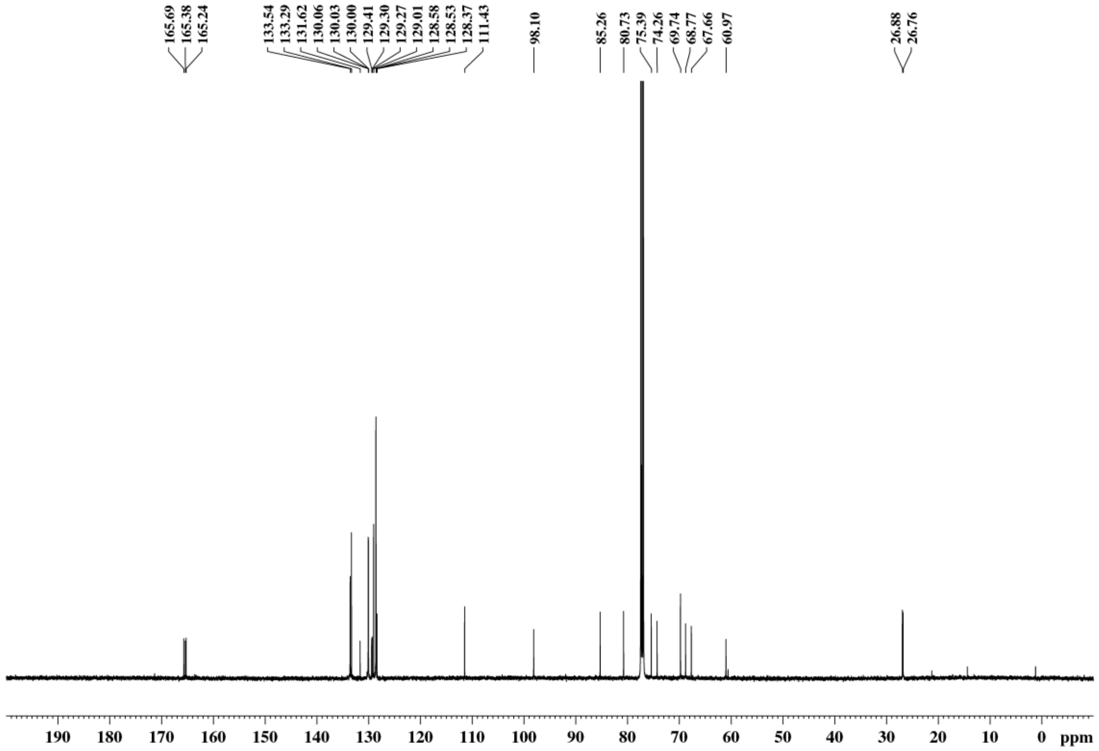

DQF-COSY:

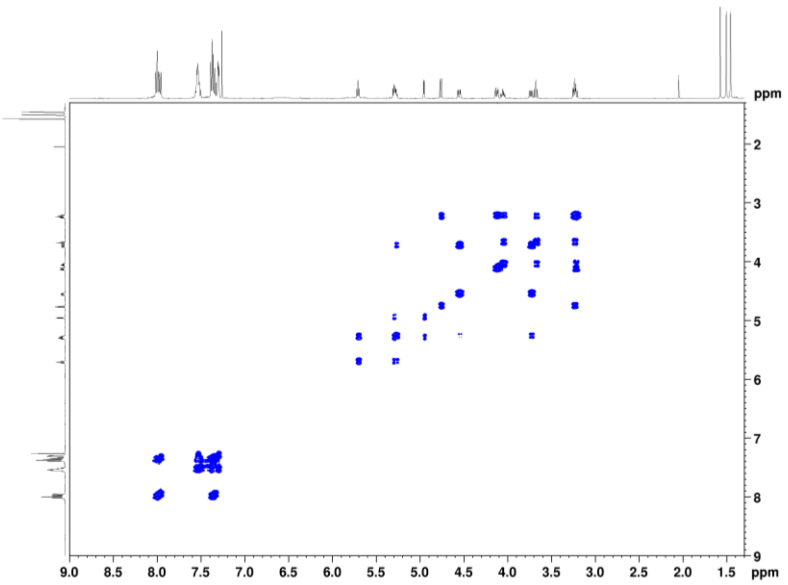

Multiplicity edited HSQC (CH and CH_3 positive, CH_2 negative):

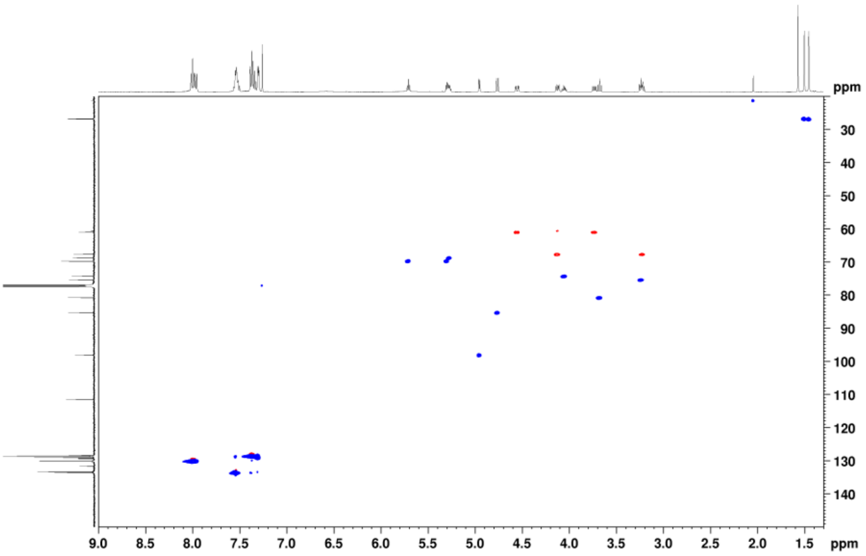

HMBC**:**

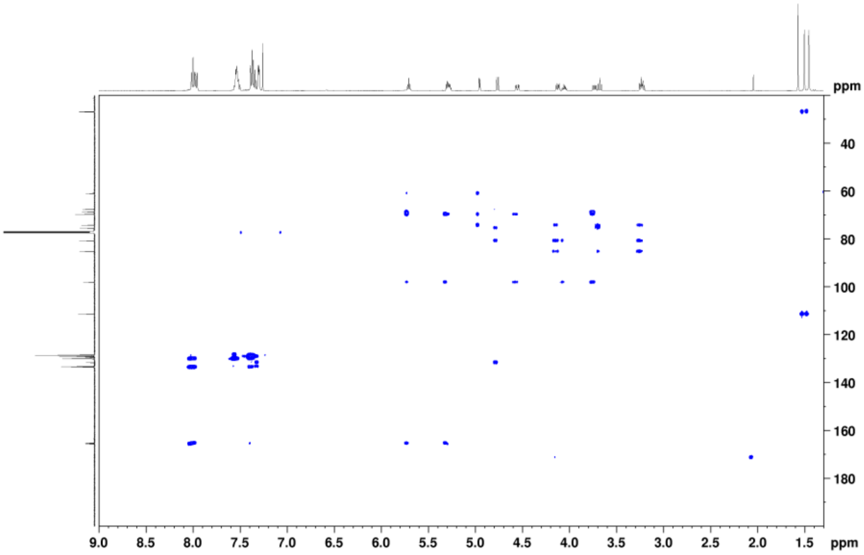

Thiophenyl *O*-(2,3,4-tri-*O*-benzyl-β-D-xylopyranosyl)-(1→4)-2,3-*O*-propylidene-β-D-xylopyranoside (6):

^{1}H NMR (500.20 MHz, $CDCl_3$, 25°C):

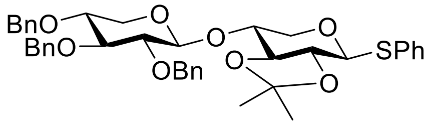

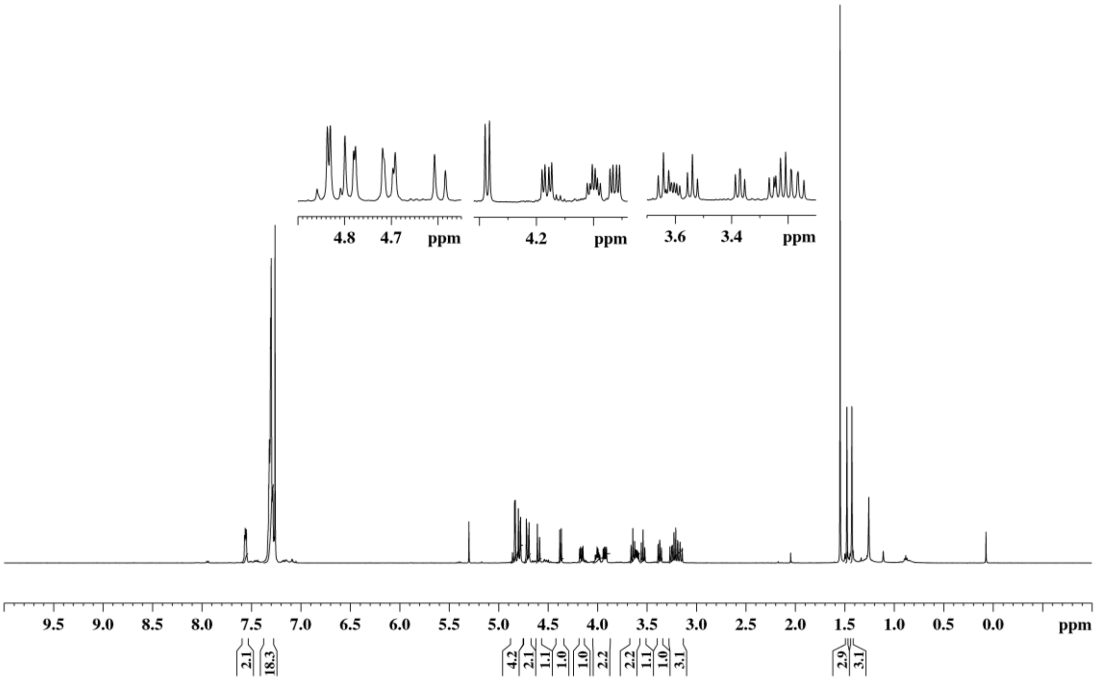

$^{13}C\{^{1}H\}$ NMR (125.8 MHz, $CDCl_3$, 25°C):

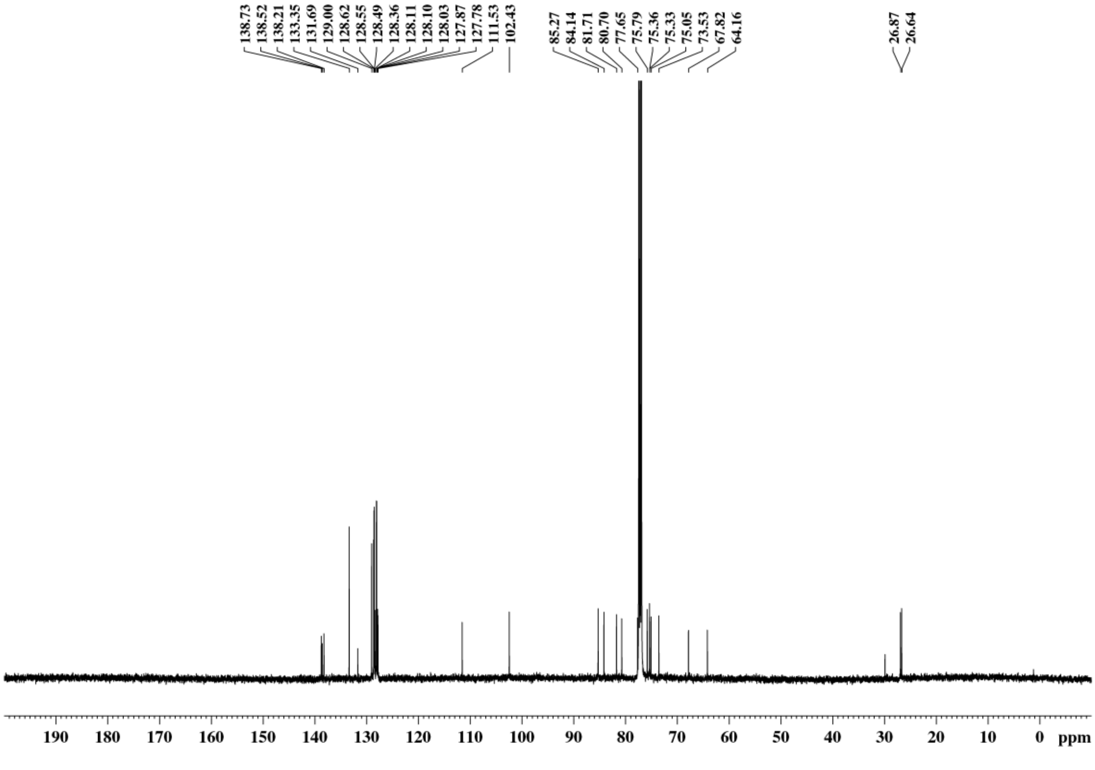

DQF-COSY:

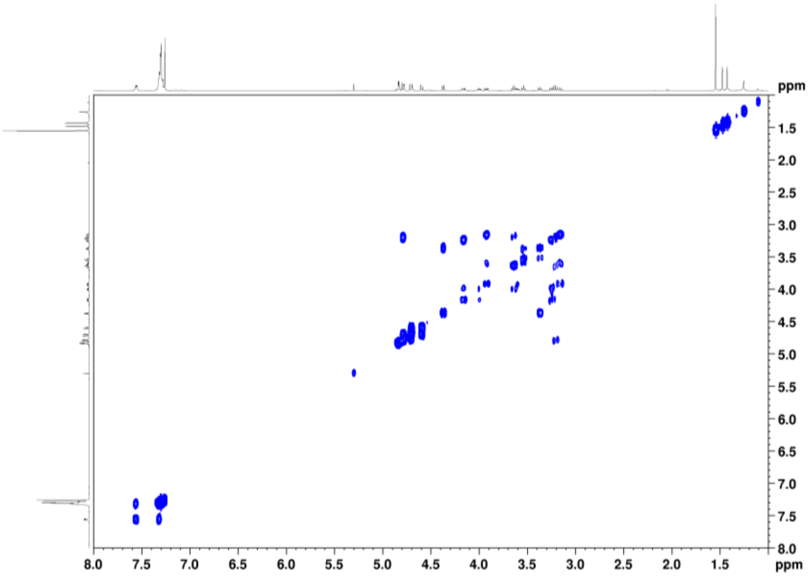

Multiplicity edited HSQC (CH and CH_3 positive, CH_2 negative):

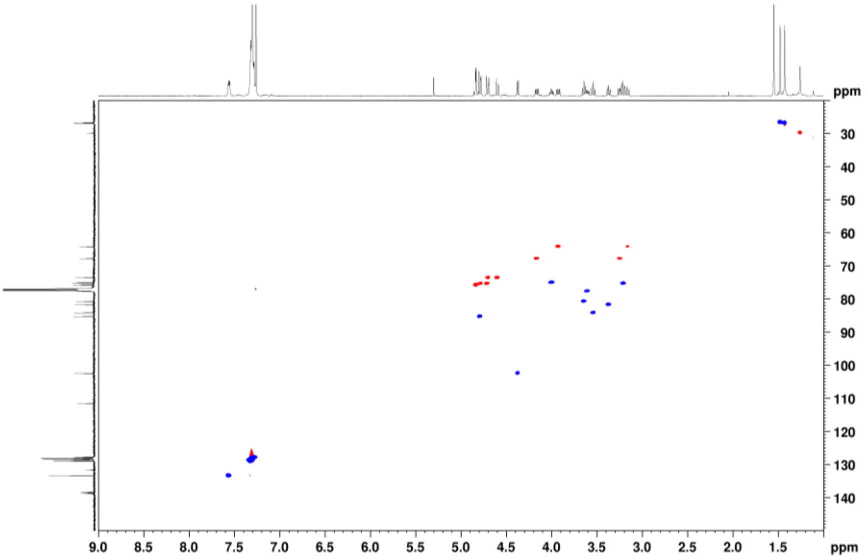

HMBC**:**

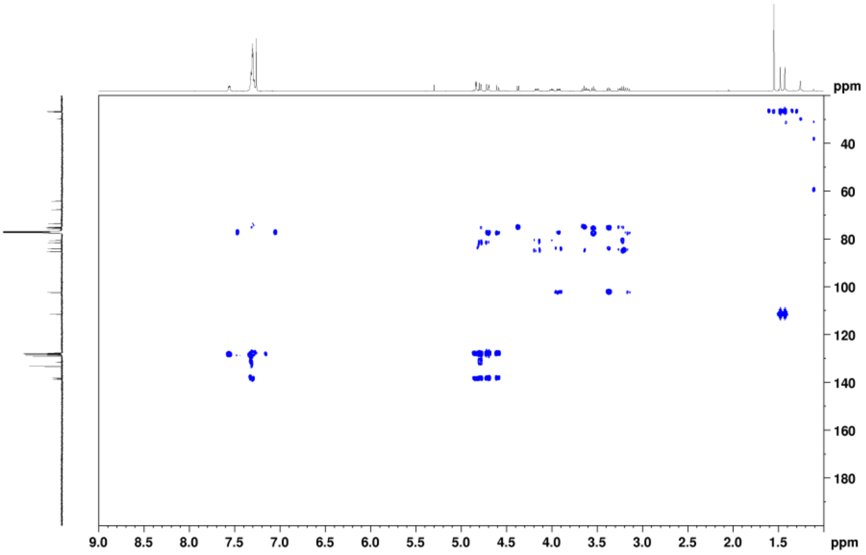

Thiophenyl *O*-(2,3,4-tri-*O*-benzyl-β-D-xylopyranosyl)-(1→4)-β-D-xyloopyranoside (7):

^{1}H NMR (500.20 MHz, $CDCl_3$, 25°C):

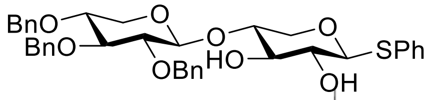

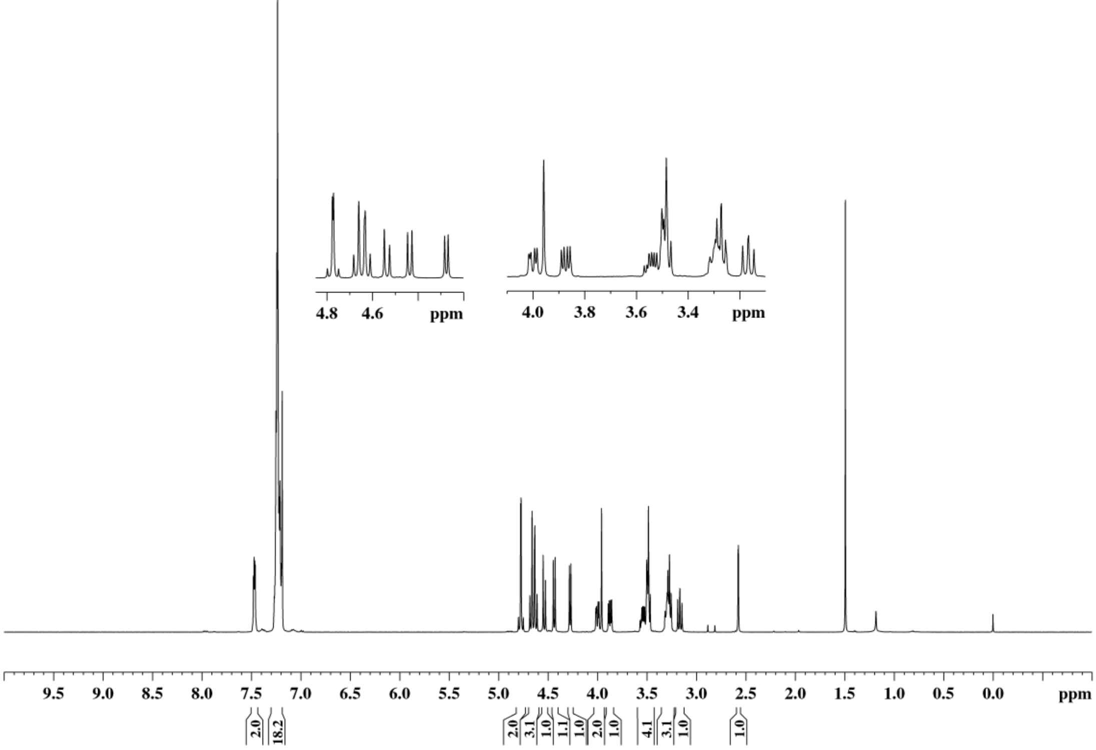

^{13}C{^{1}H} NMR (125.8 MHz, $CDCl_3$, 25°C):

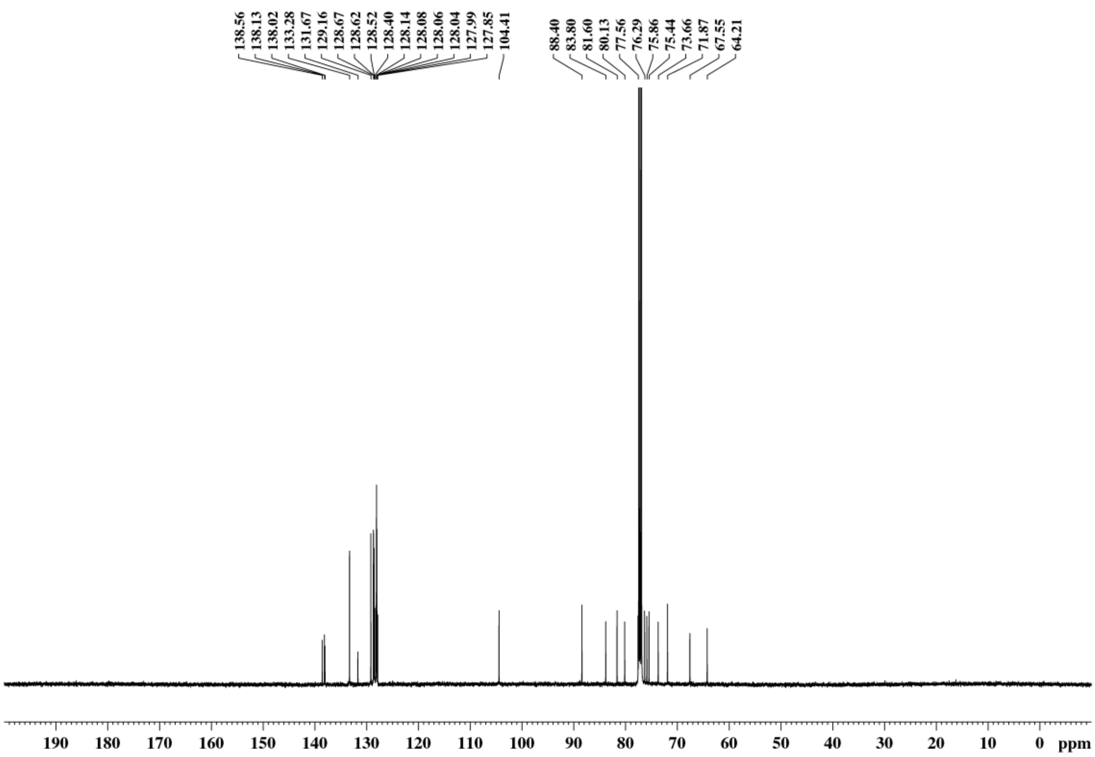

DQF-COSY:

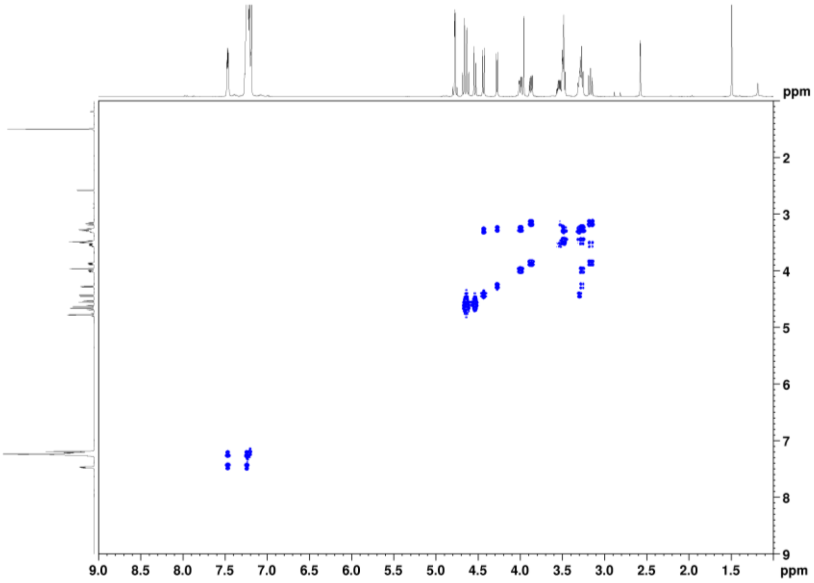

Multiplicity edited HSQC (CH and CH_3 positive, CH_2 negative):

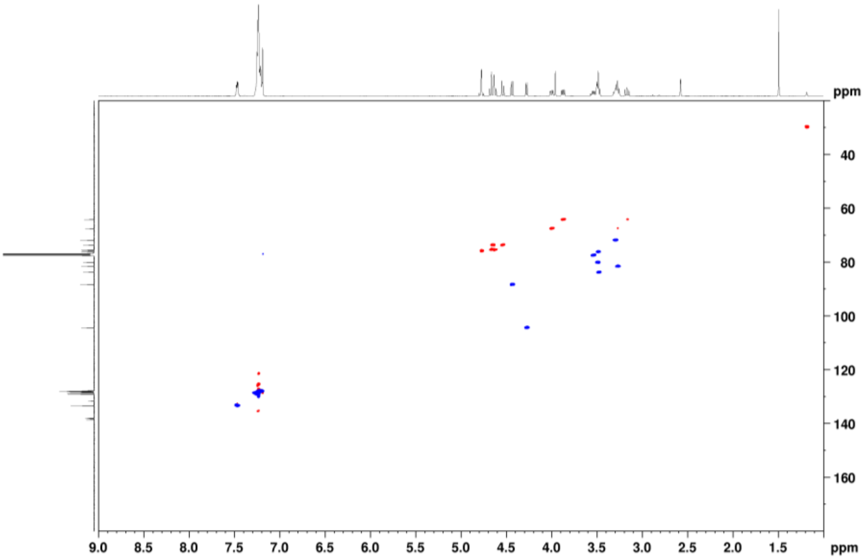

HMBC:

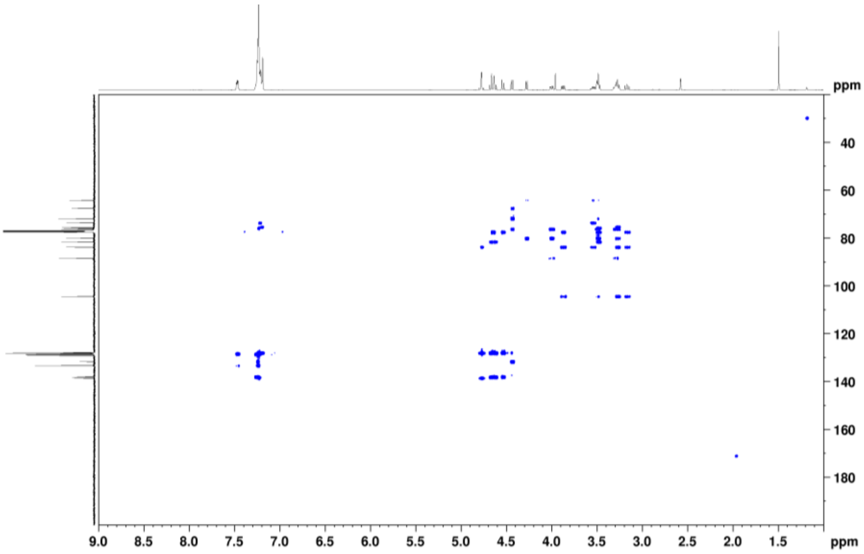

Thiophenyl *O*-(2,3,4-tri-*O*-benzyl-β-D-xylopyranosyl)-(1→4)-2,3-di-*O*-acetyl-β-D-xylopyranoside (8):

^{1}H NMR (500.20 MHz, $CDCl_3$, 25°C):

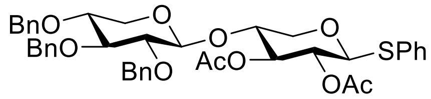

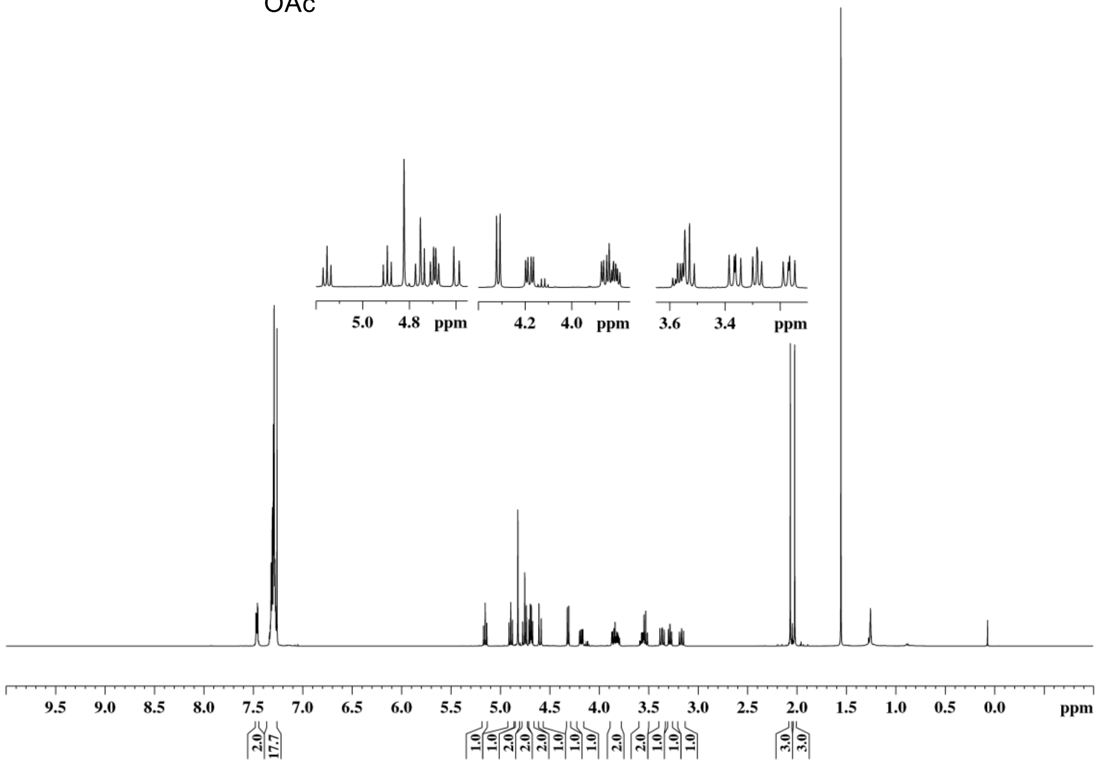

$^{13}C\{^1H\}$ NMR (125.8 MHz, $CDCl_3$, 25°C):

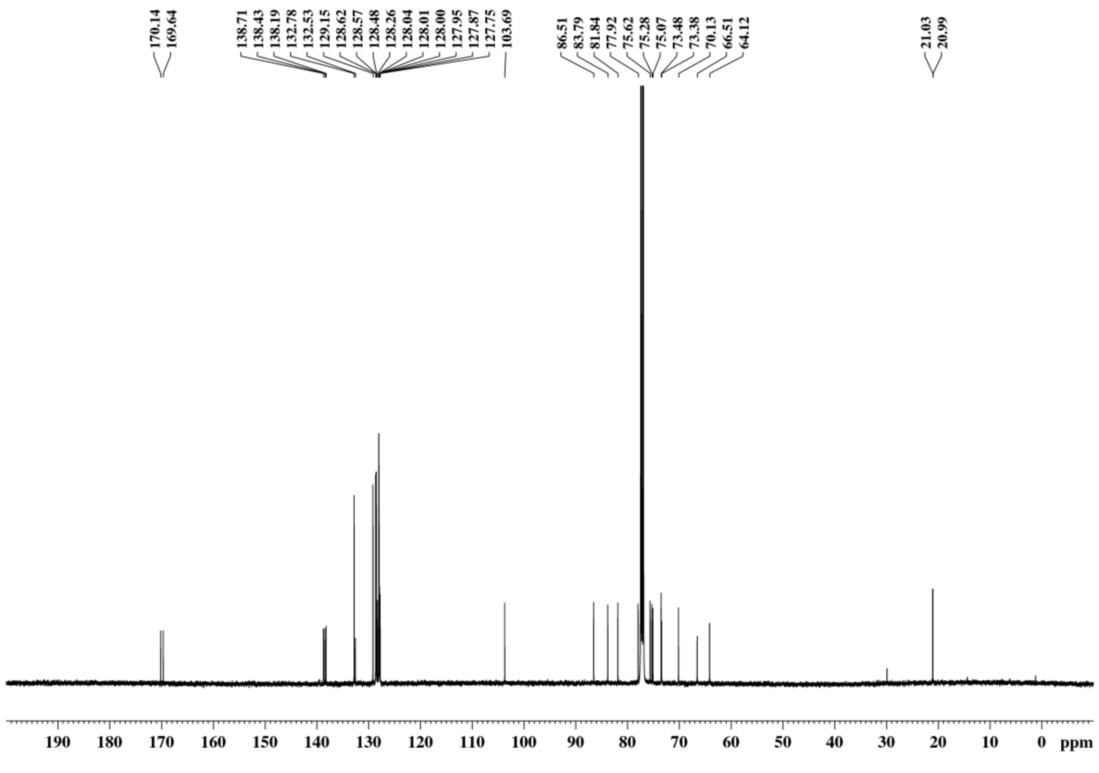

DQF-COSY:

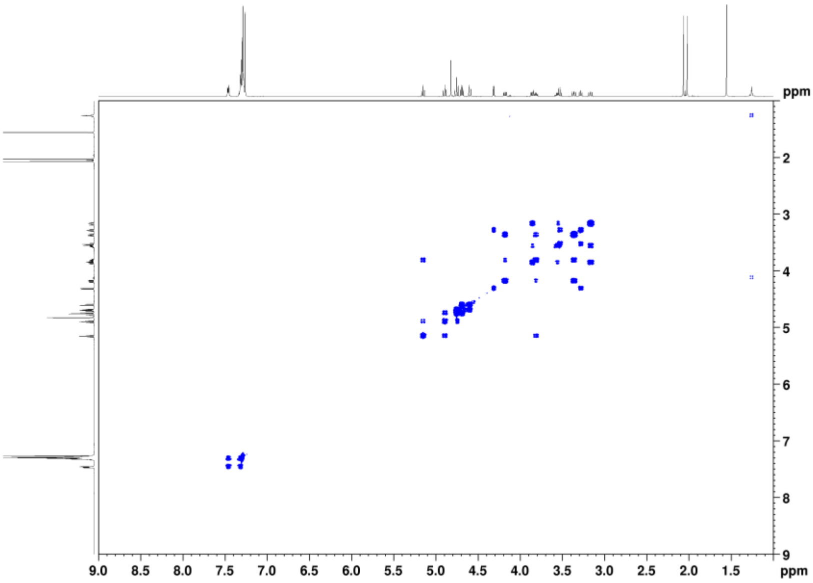

Multiplicity edited HSQC (CH and CH_3 positive, CH_2 negative):

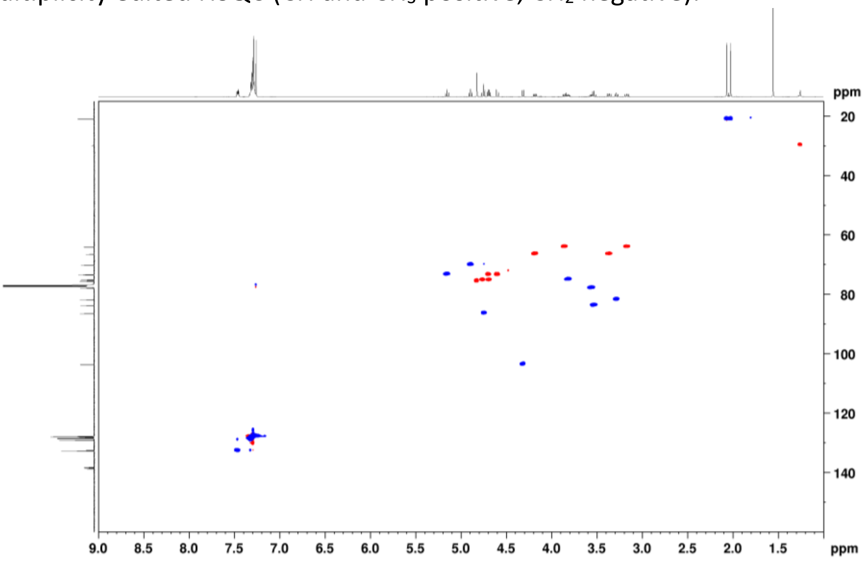

HMBC:

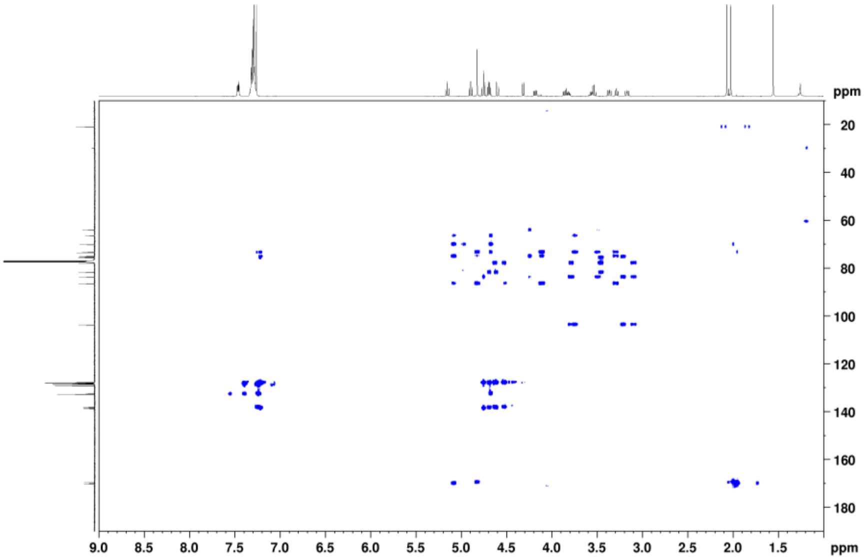

Thiophenyl *O*-(2,3,4-tri-*O*-benzyl-β-D-xyloopyranosyl)-(1→4)-2-*O*-acetyl-β-D-xylopyranoside (9):

^{1}H NMR (500.20 MHz, CDCl$_3$, 25°C):

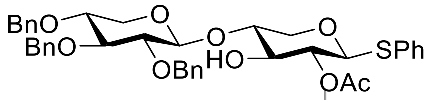

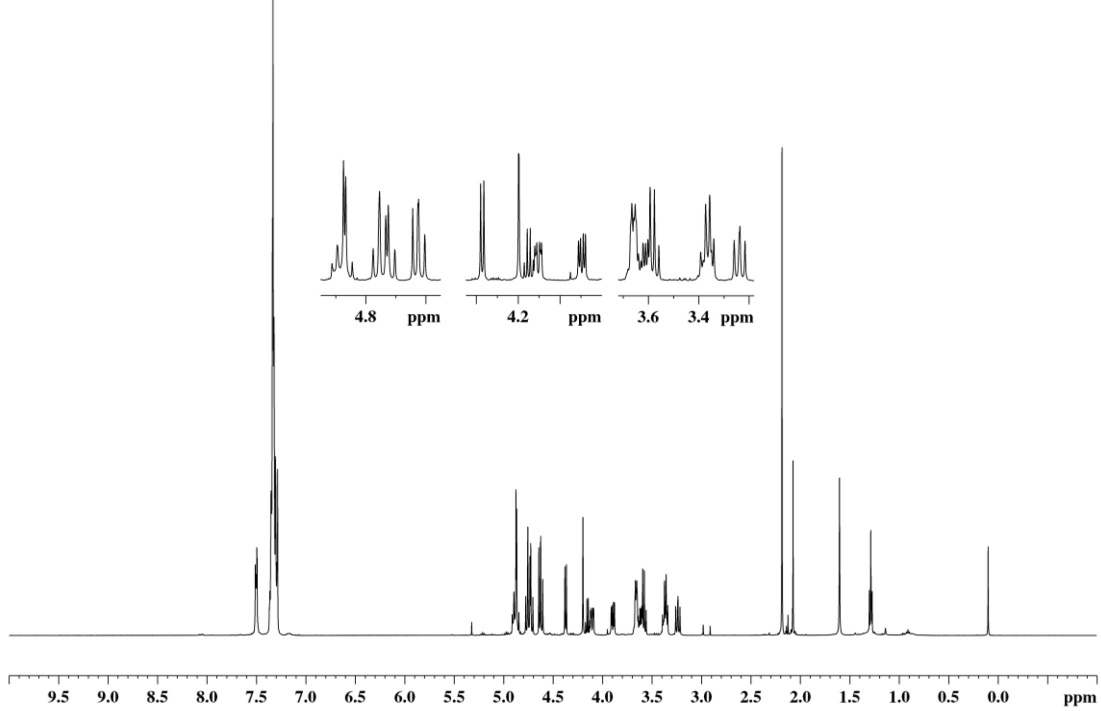

^{13}C{^{1}H} NMR (125.8 MHz, CDCl$_3$, 25°C):

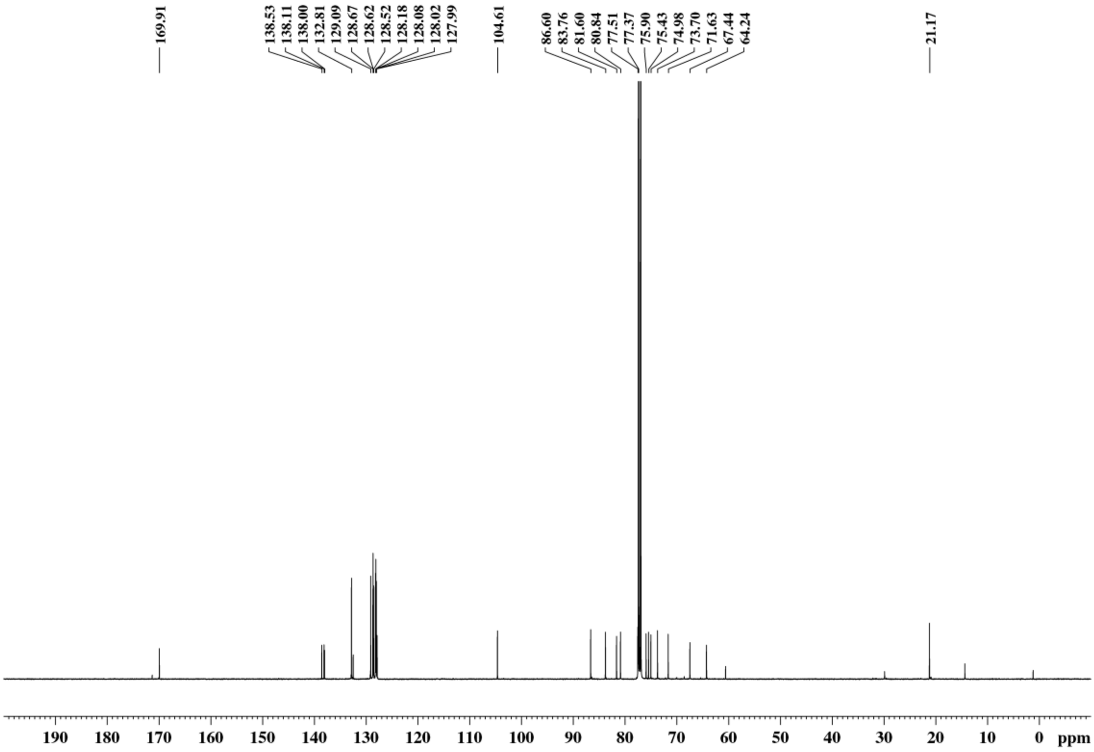

DQF-COSY:

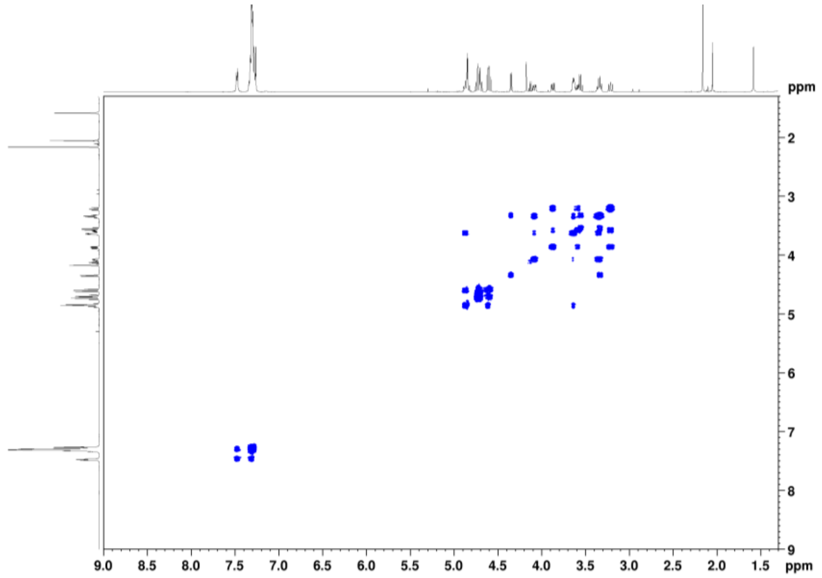

Multiplicity edited HSQC (CH and CH_3 positive, CH_2 negative):

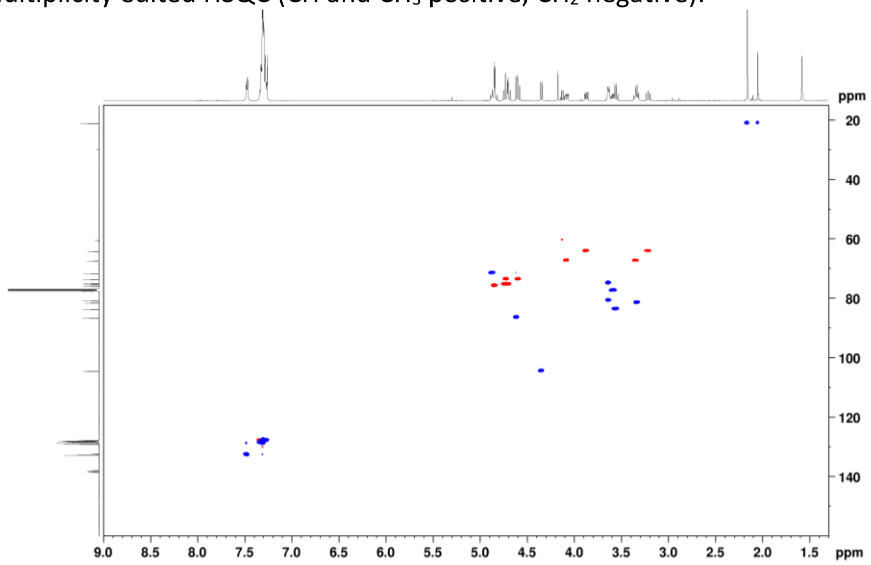

HMBC**:**

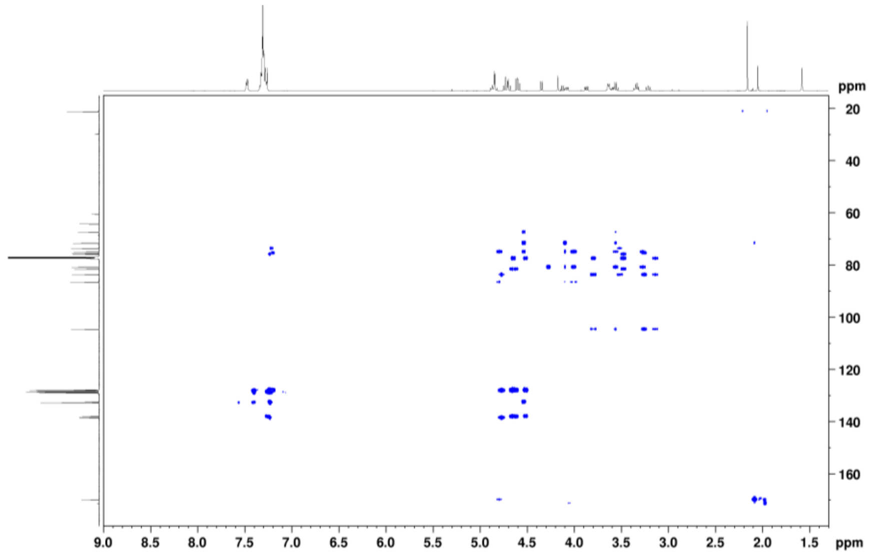

Methyl *O*-(2,3,4-tri-*O*-benzyl-β-D-xylopyranosyl)-(1→4)-*O*-(2,3-di-*O*-acetyl-β-D-xylopyranosyl)-(1→4)-2,3-di-*O*-benzyl-β-D-xylopyranoside (11):

^{1}H NMR (500.20 MHz, $CDCl_3$, 25°C):

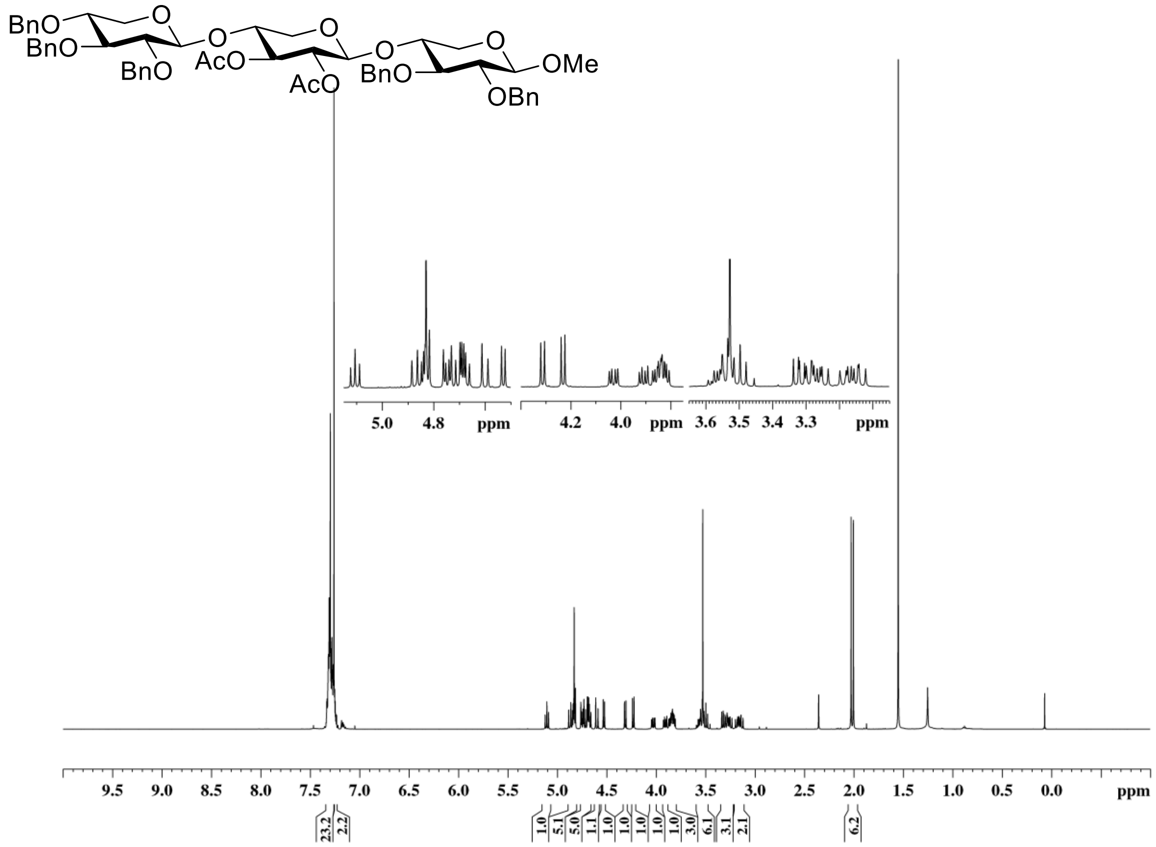

$^{13}C\{^1H\}$ NMR (125.8 MHz, $CDCl_3$, 25°C):

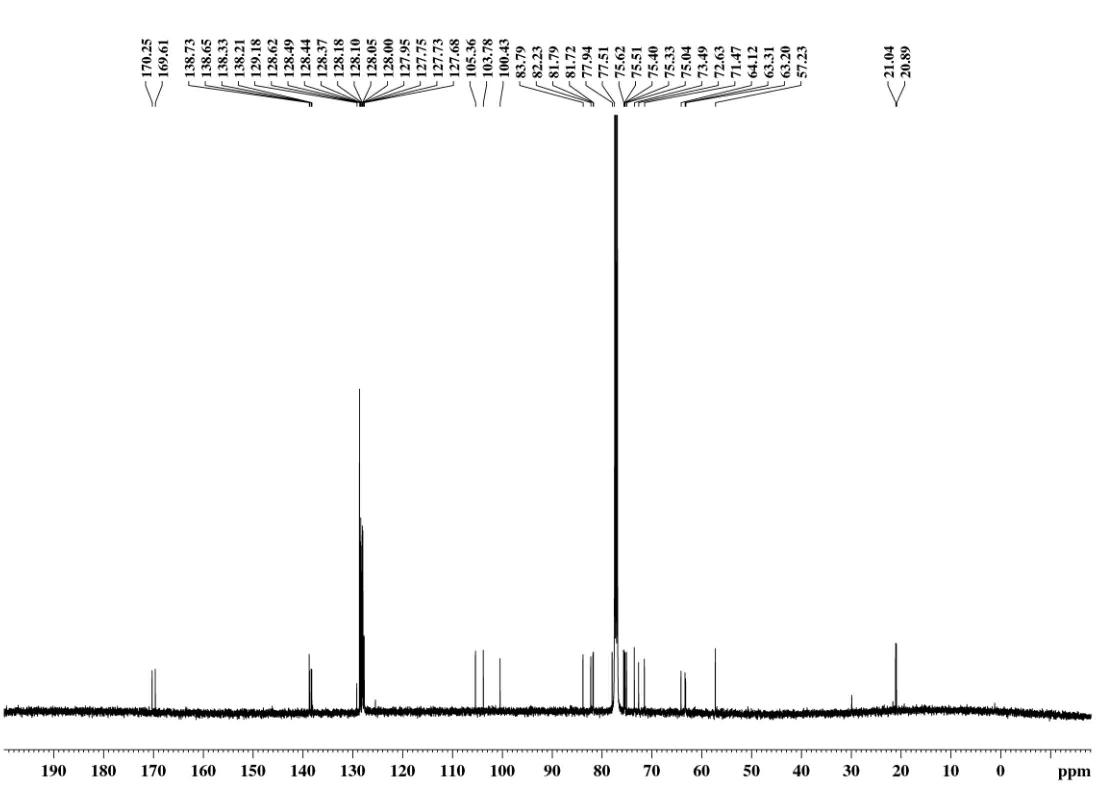

DQF-COSY:

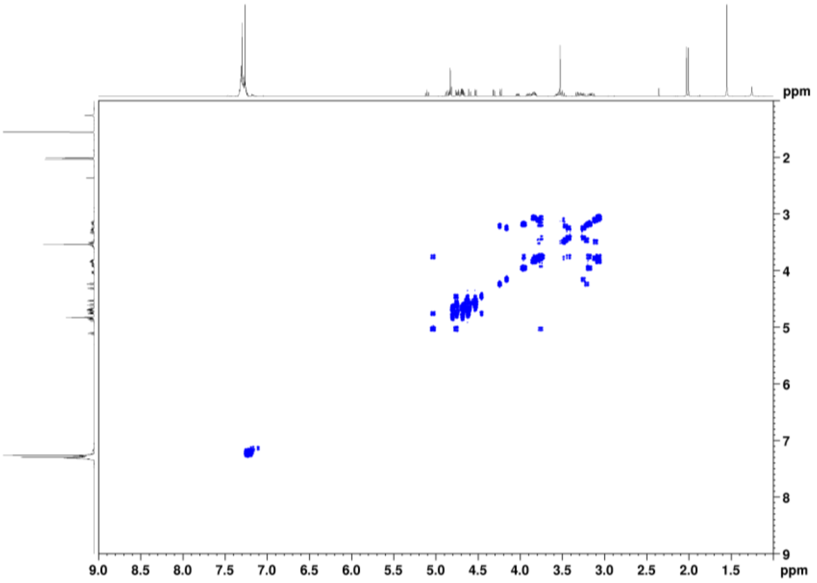

Multiplicity edited HSQC (CH and CH_3 positive, CH_2 negative):

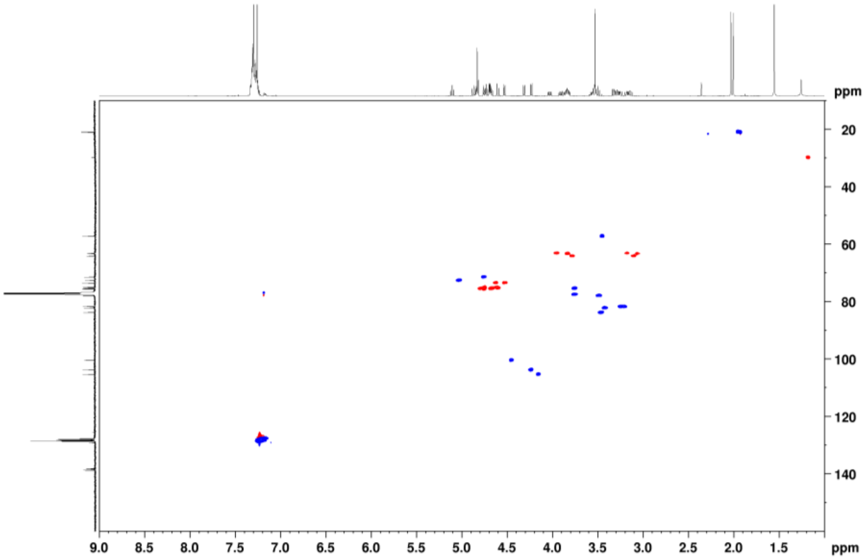

HMBC**:**

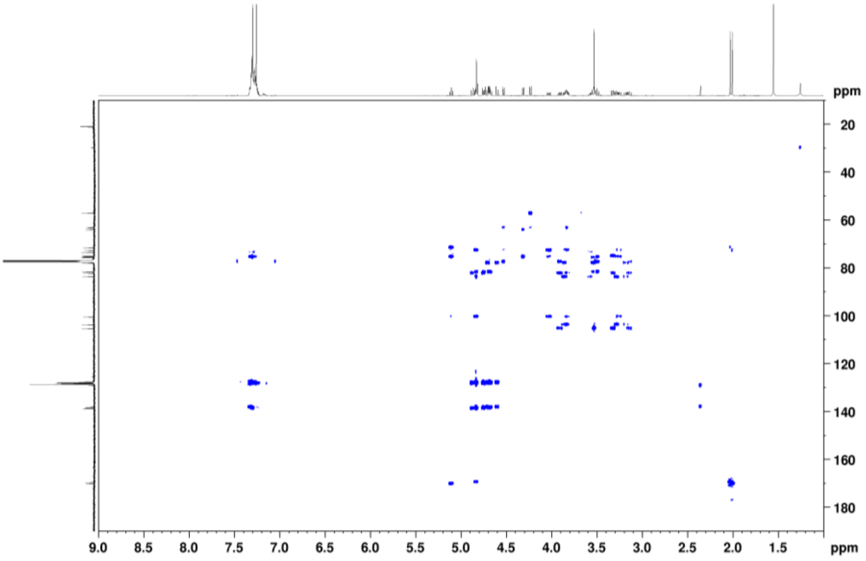

Methyl *O*-(2,3,4-tri-*O*-benzyl-β-D-xylopyranosyl)-(1→4)-*O*-(2,3-di-*O*-acetyl-β-D-xylopyranosyl)-(1→4)-2,3-di-*O*-benzyl-β-D-xylopyranoside (12):

^{1}H NMR (500.20 MHz, $CDCl_3$, 25°C):

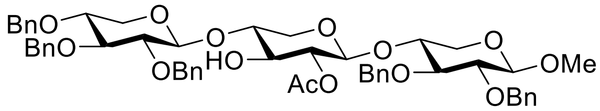

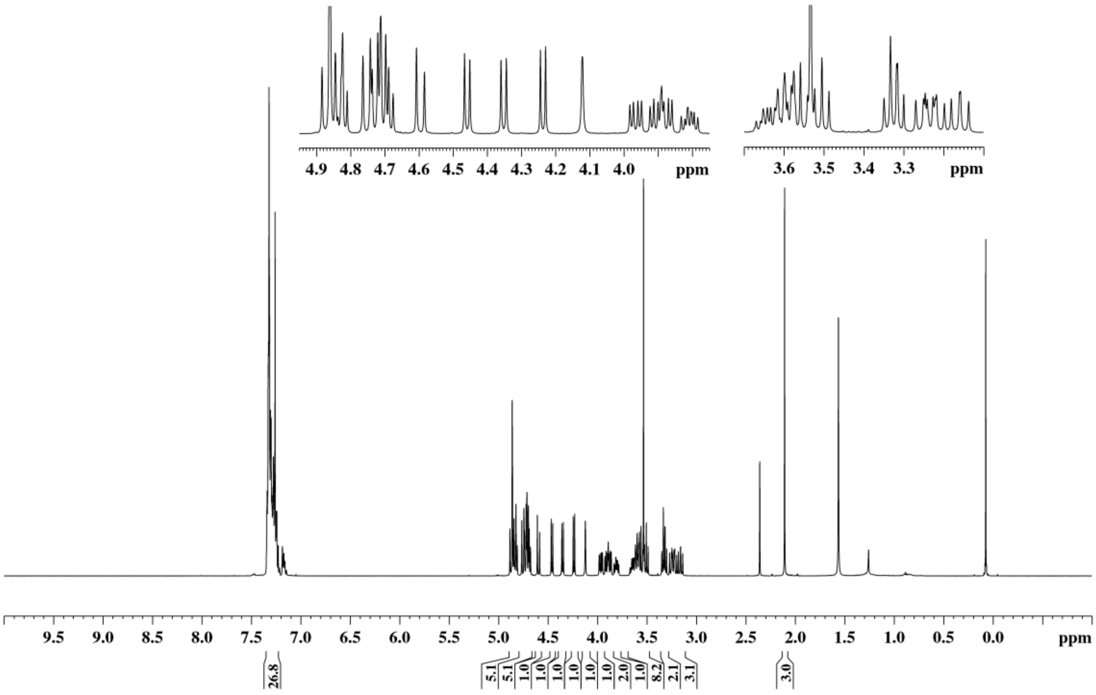

$^{13}C\{^{1}H\}$ NMR (125.8 MHz, $CDCl_3$, 25°C):

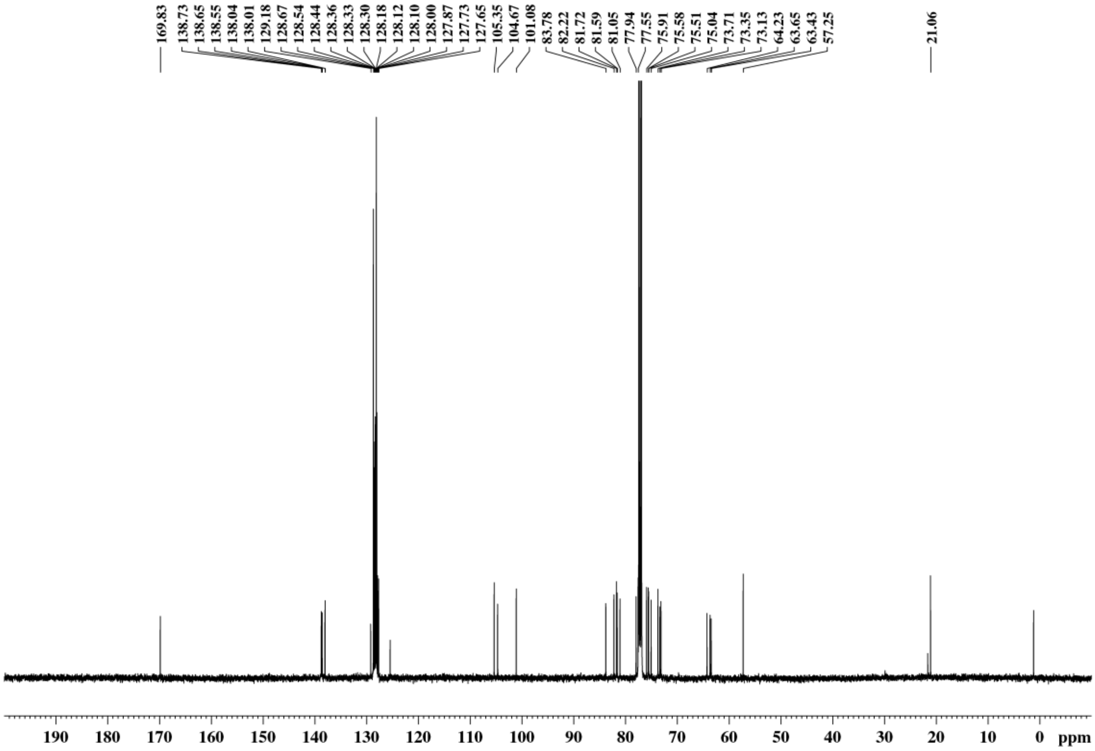

DQF-COSY:

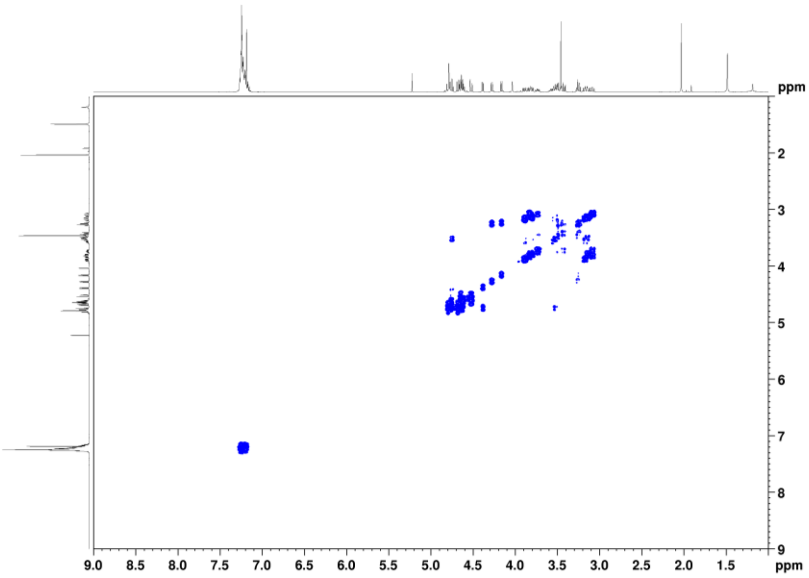

Multiplicity edited HSQC (CH and CH_3 positive, CH_2 negative):

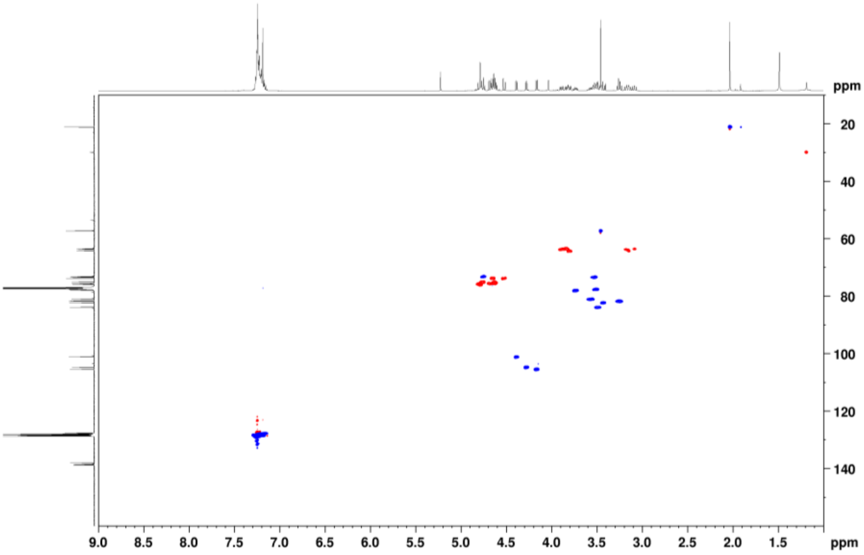

HMBC:

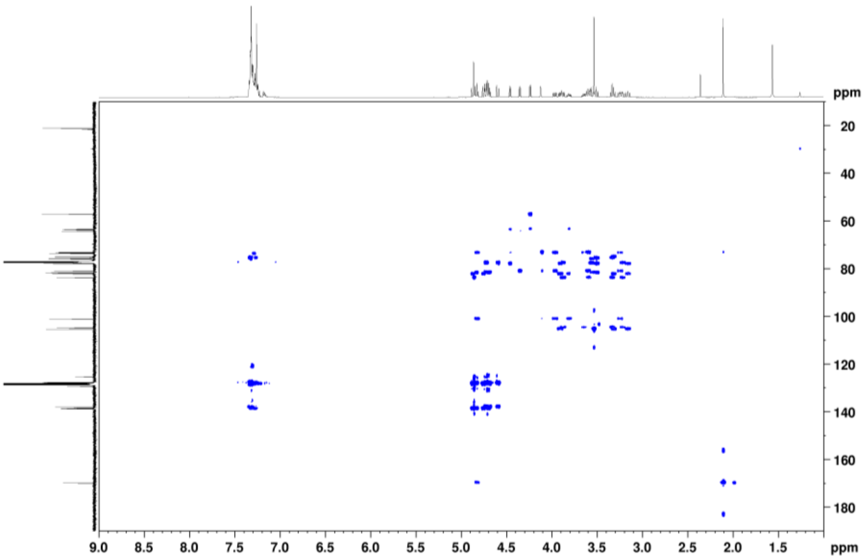

Methyl *O*-(β-D-xylopyranosyl)-(1→4)-*O*-(2,3-di-*O*-acetyl-β-D-xylopyranosyl)-(1→4)-β-D-xylopyranoside (1a):

^{1}H NMR (500.20 MHz, MeOD, 25°C):

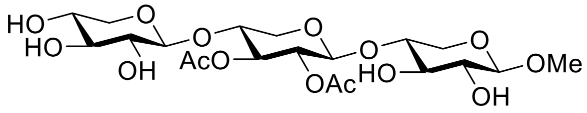

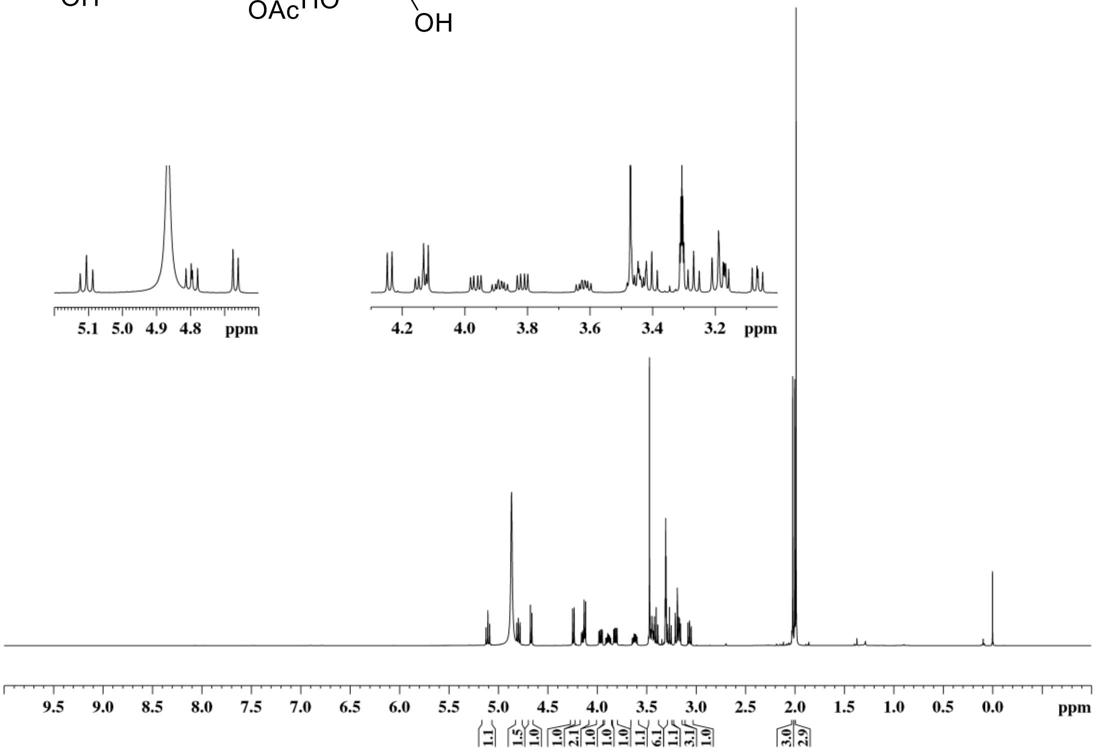

^{13}C{^{1}H} NMR (125.8 MHz, MeOD, 25°C):

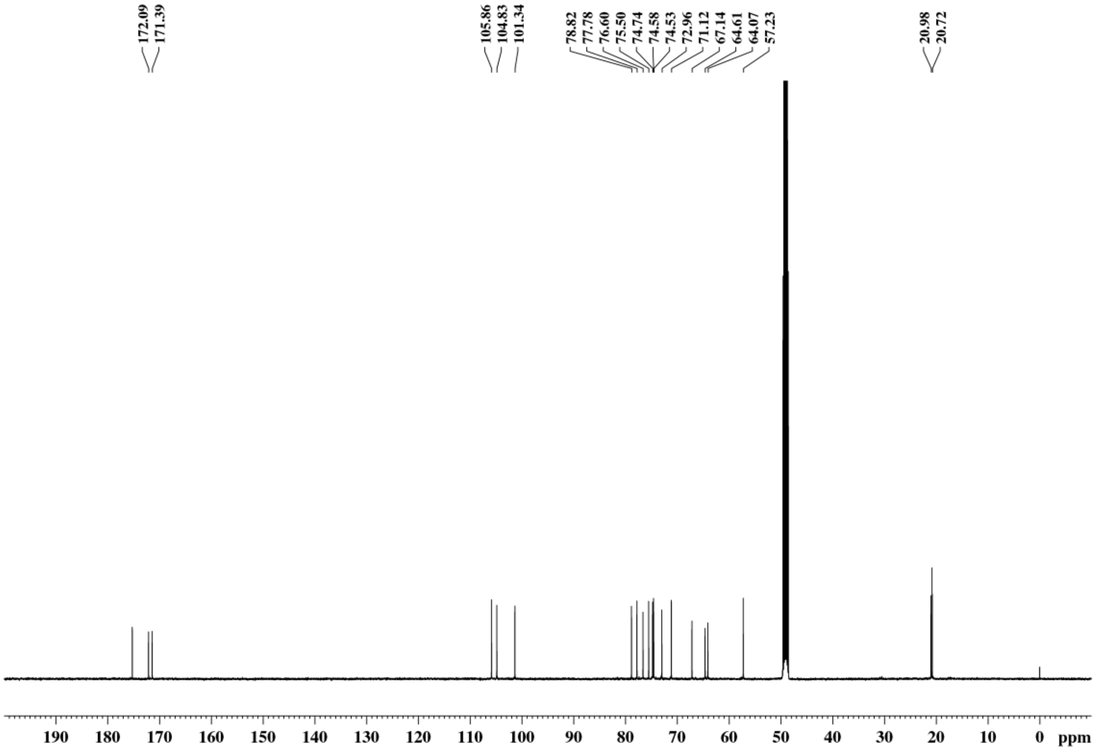

DQF-COSY:

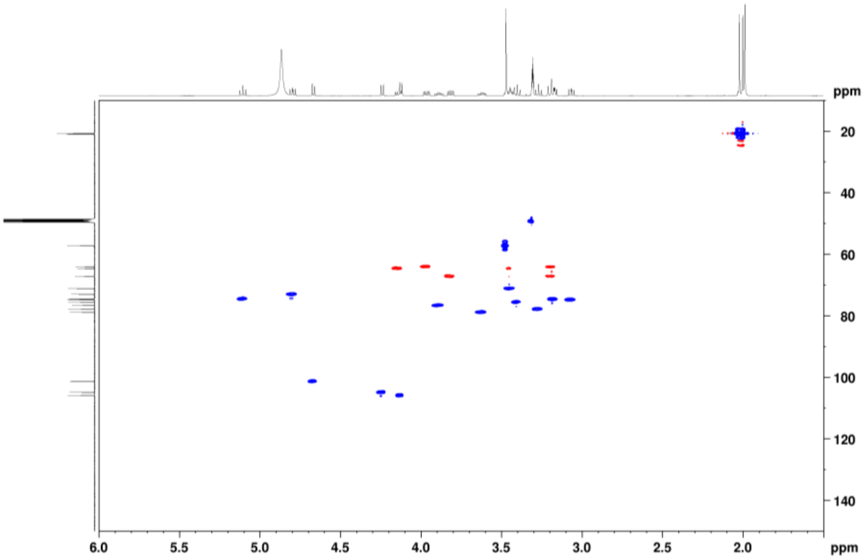

Multiplicity edited HSQC (CH and CH_3 positive, CH_2 negative):

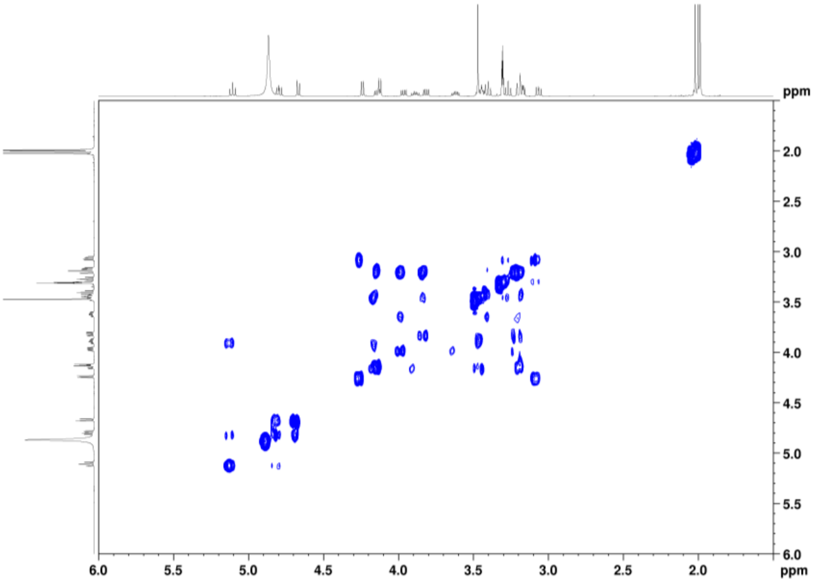

HMBC:

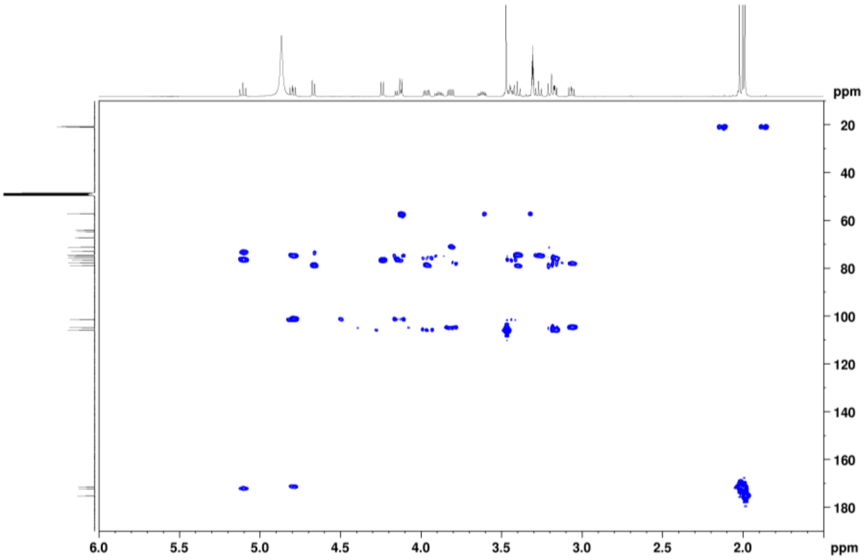

Methyl *O*-(β-D-xylopyranosyl)-(1→4)-*O*-(2-*O*-acetyl-β-D-xylopyranosyl)-(1→4)-β-D-xylopyranoside (1b):

^{1}H NMR (500.20 MHz, MeOD, 25°C):

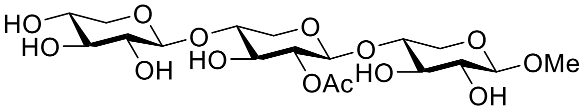

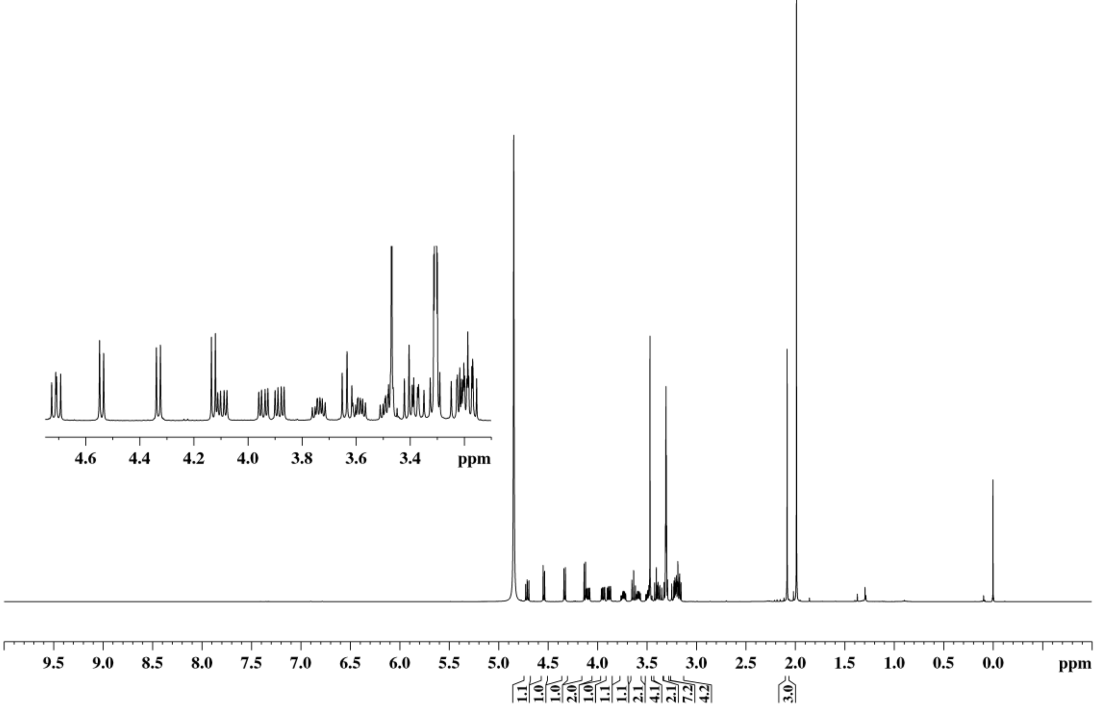

^{13}C{^{1}H} NMR (125.8 MHz, MeOD, 25°C):

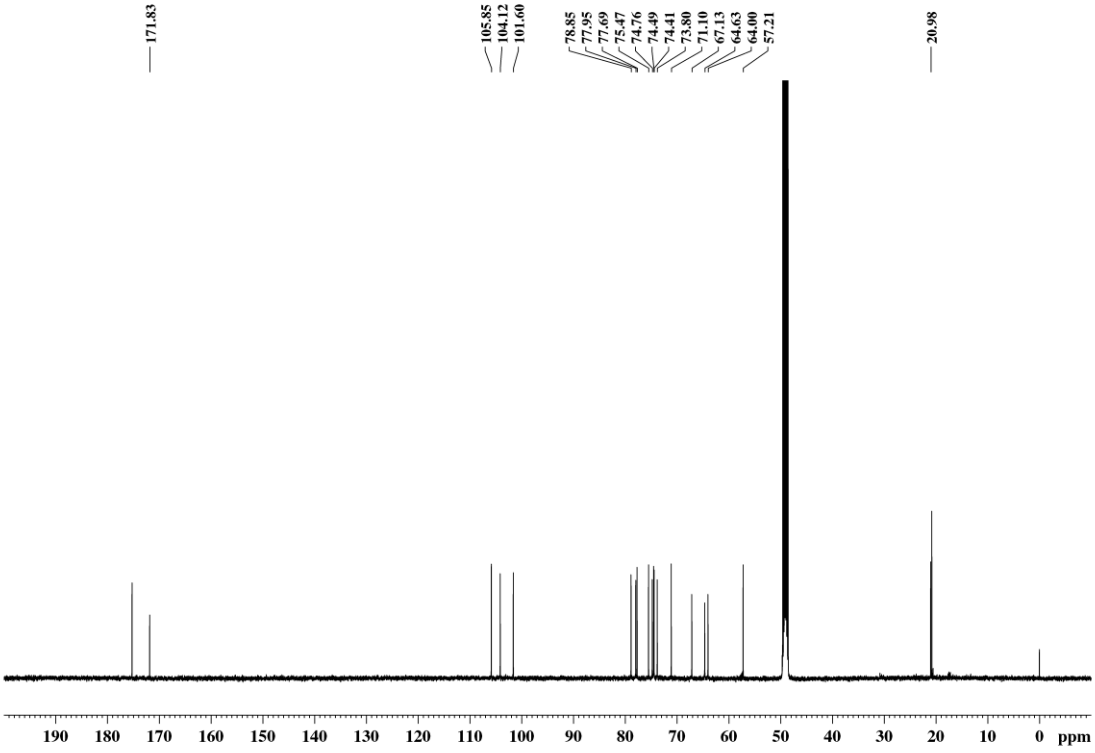

DQF-COSY:

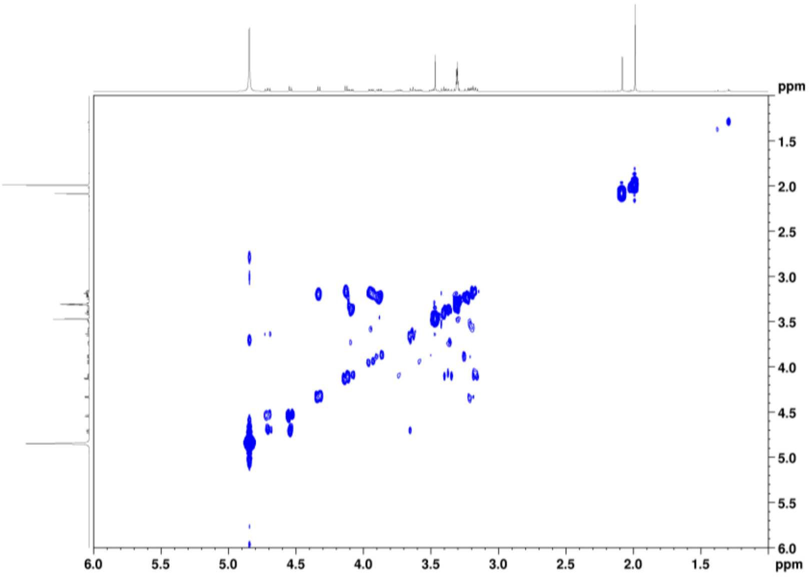

Multiplicity edited HSQC (CH and CH_3 positive, CH_2 negative):

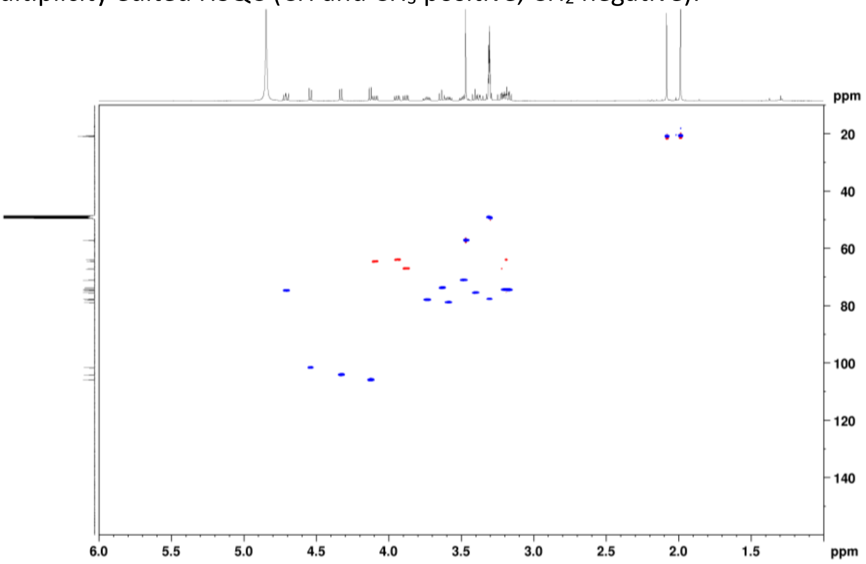

HMBC**:**

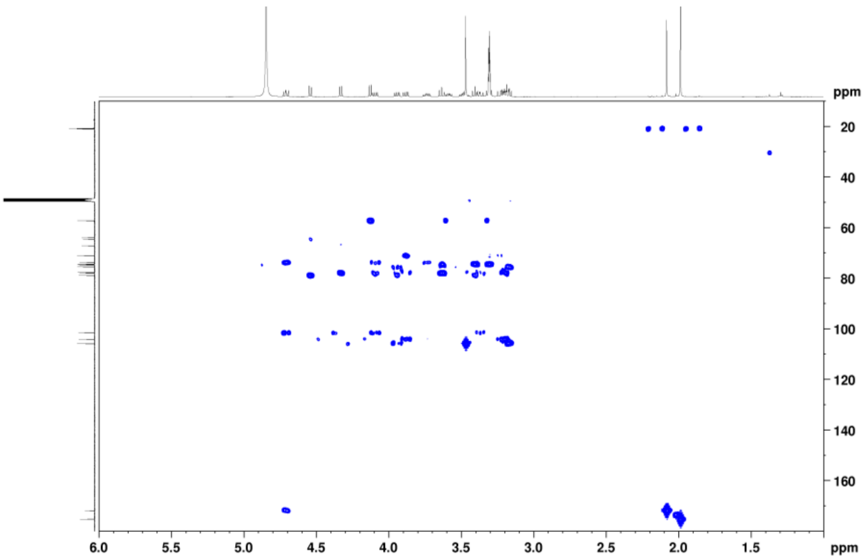

Thiophenyl 6-*O*-benzyl-2,3-di-*O*-2-naphthylmethyl-β-D-glucopyranoside (14):

^{1}H NMR (500.20 MHz, $CDCl_3$, 25°C):

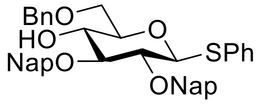

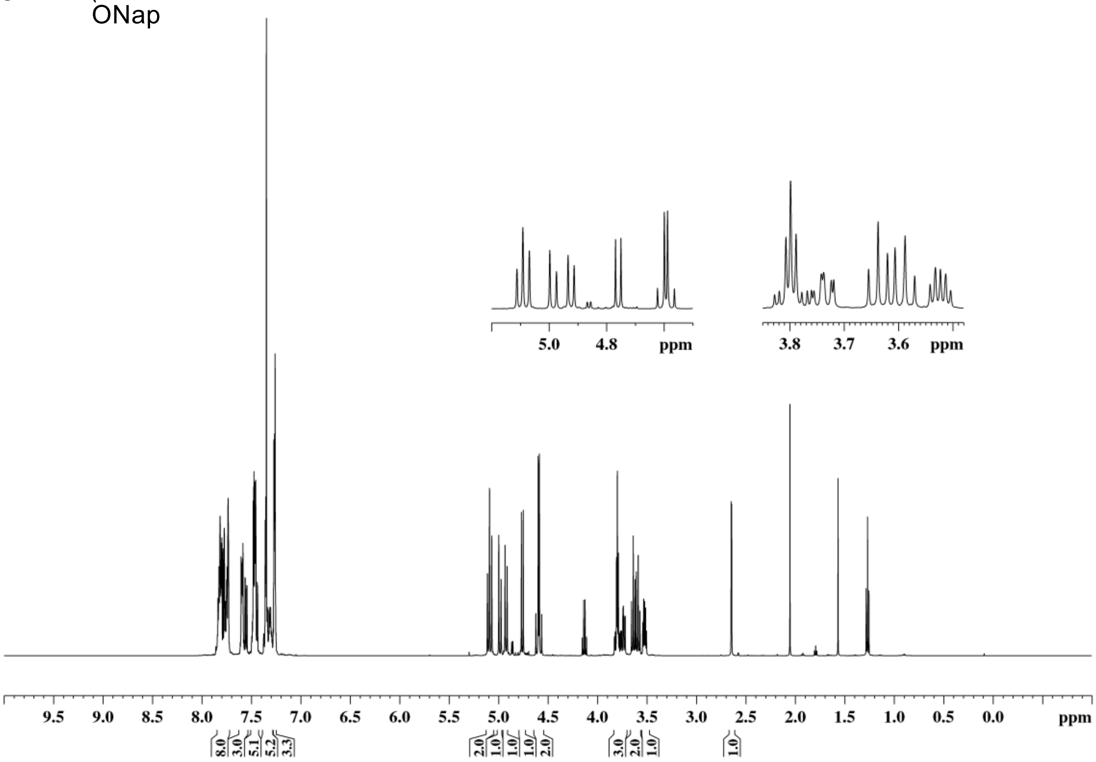

$^{13}C\{^{1}H\}$ NMR (125.8 MHz, $CDCl_3$, 25°C):

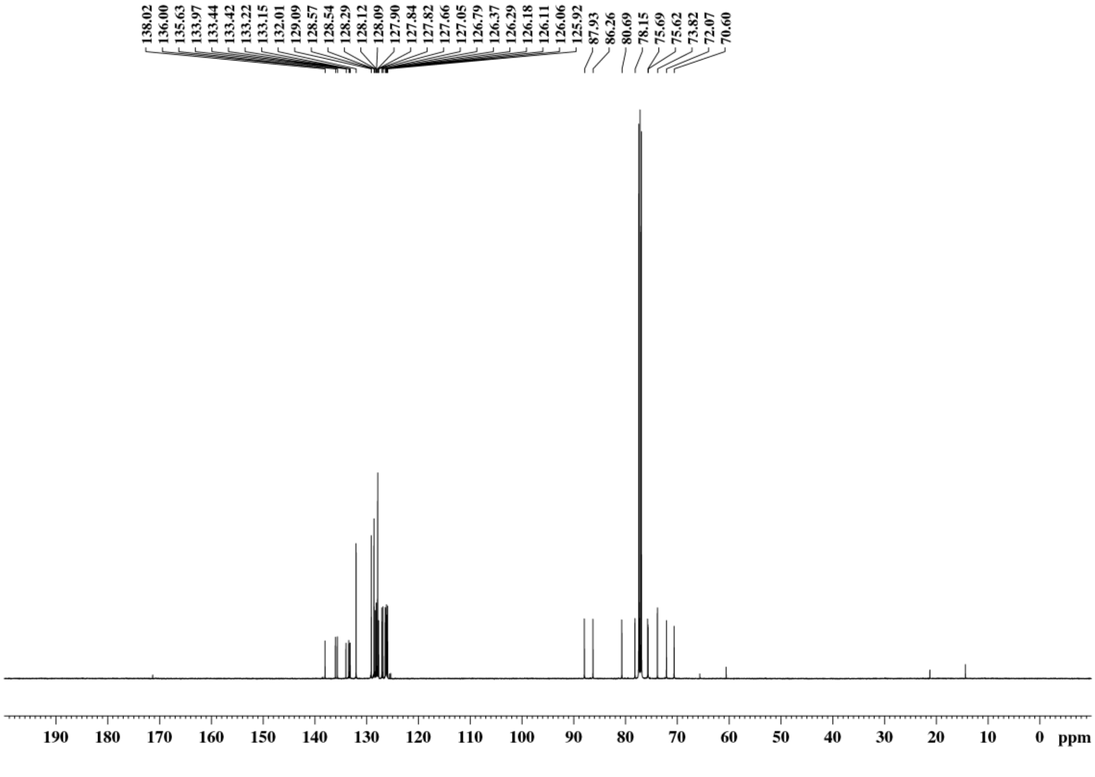

DQF-COSY:

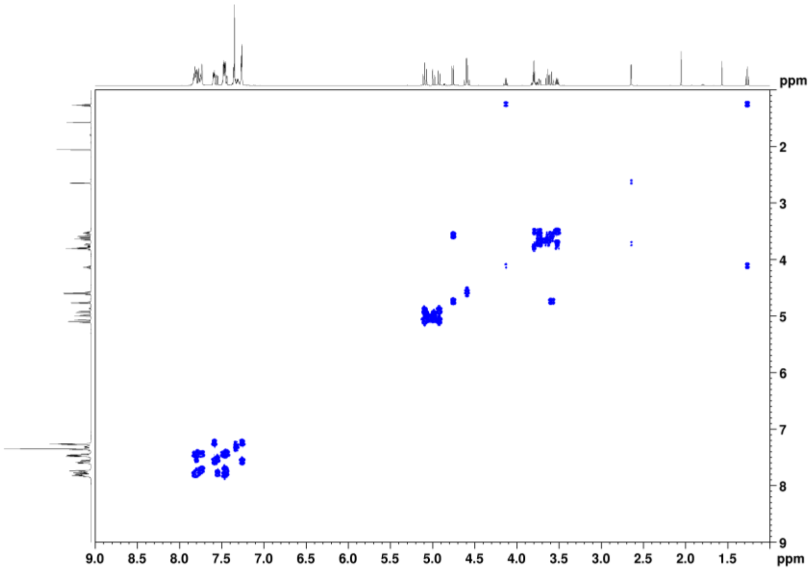

Multiplicity edited HSQC (CH and CH_3 positive, CH_2 negative):

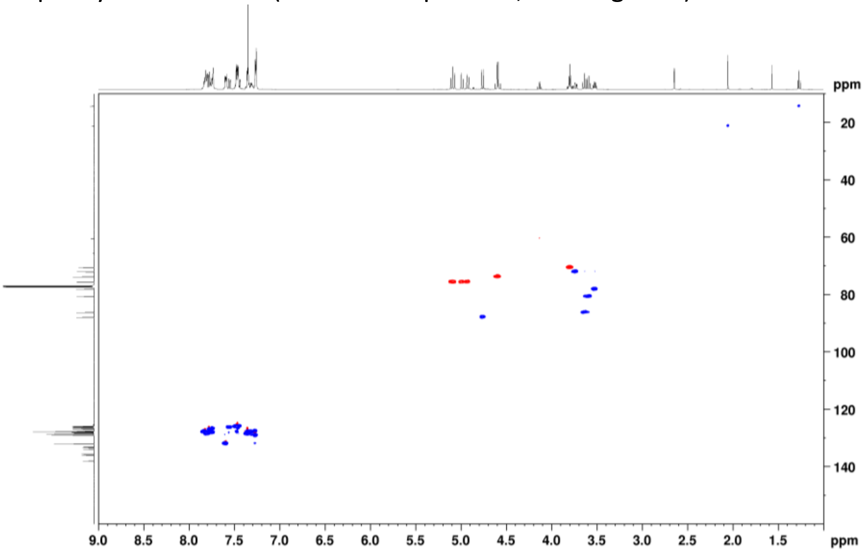

HMBC**:**

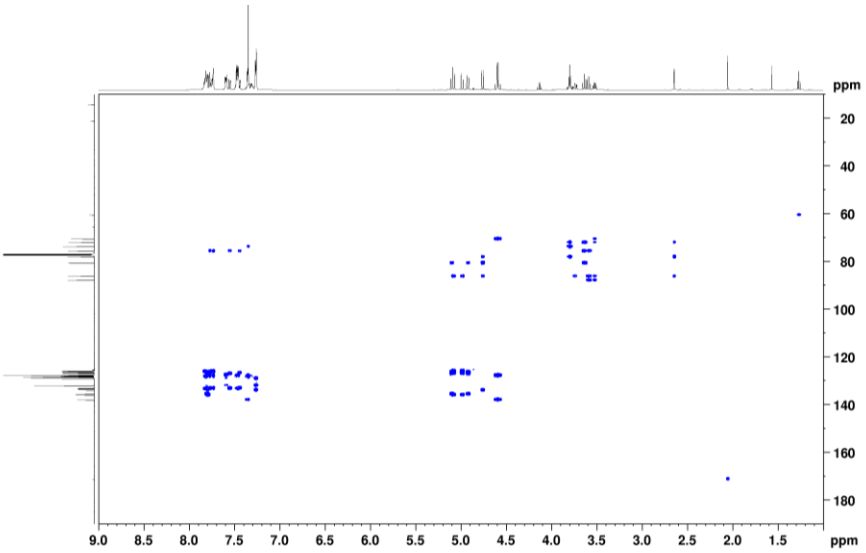

Thiophenyl *O*-(2,3,4,6-tetra-*O*-benzoyl-β-D-glucopyranosyl)-(1→4)-6-*O*-benzyl-2,3-*O*-2-naphthylmethyl-β-D-glucopyranoside (16):

^{1}H NMR (500.20 MHz, $CDCl_3$, 25°C):

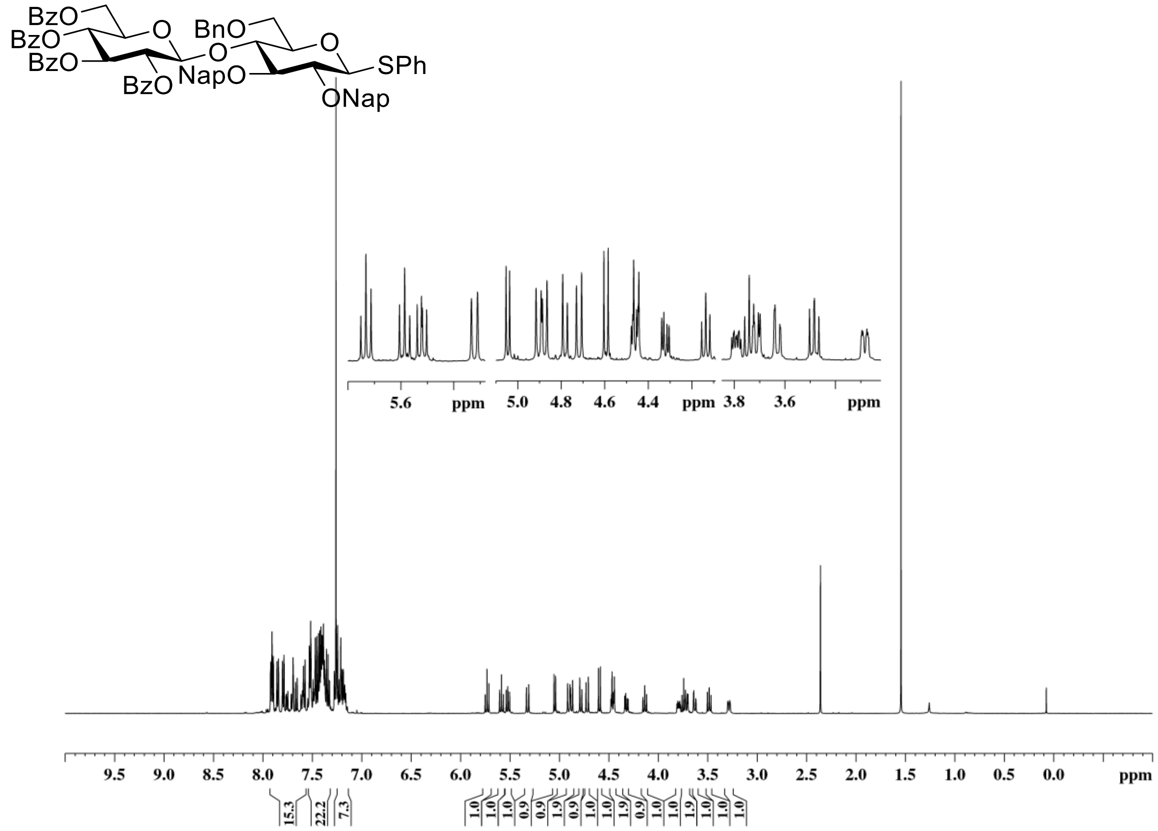

^{13}C{^{1}H} NMR (125.8 MHz, $CDCl_3$, 25°C):

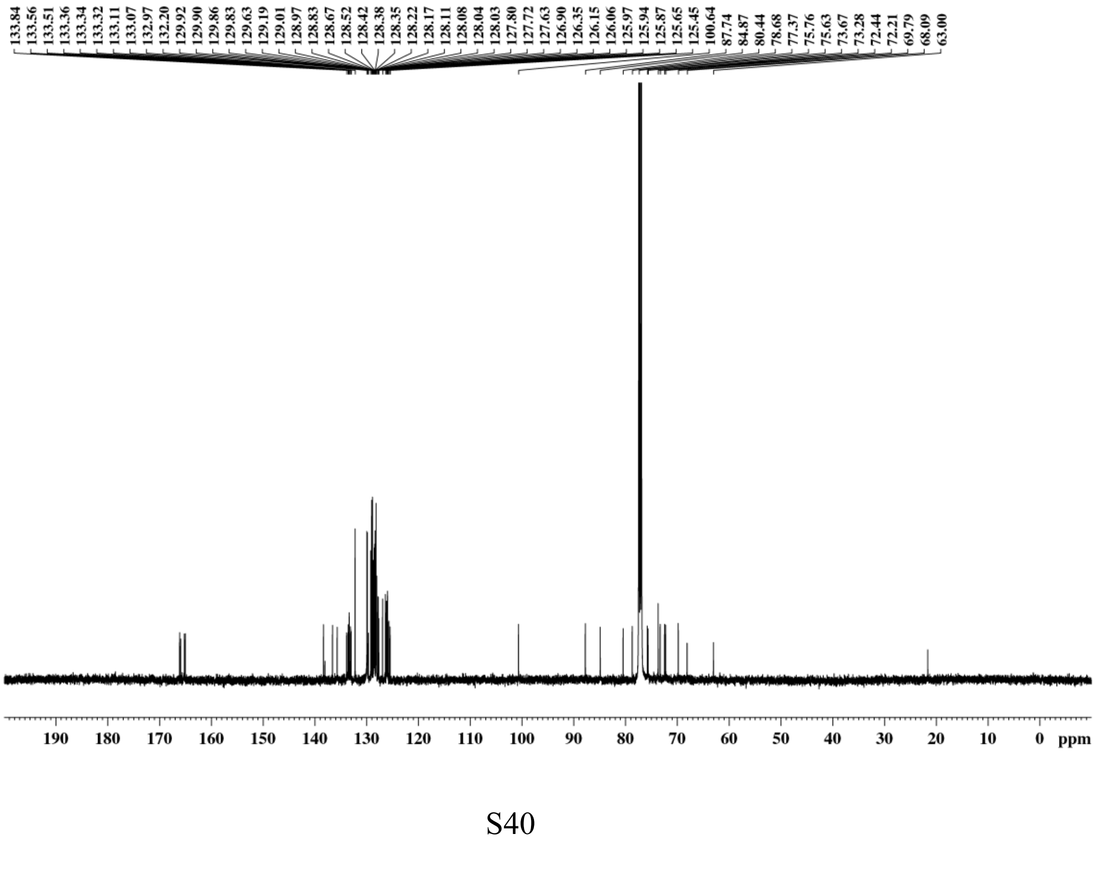

DQF-COSY:

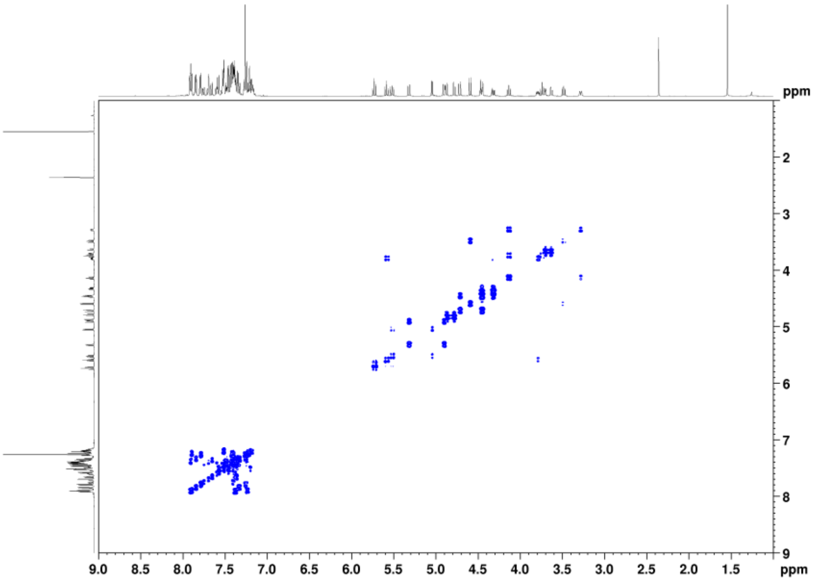

Multiplicity edited HSQC (CH and CH_3 positive, CH_2 negative):

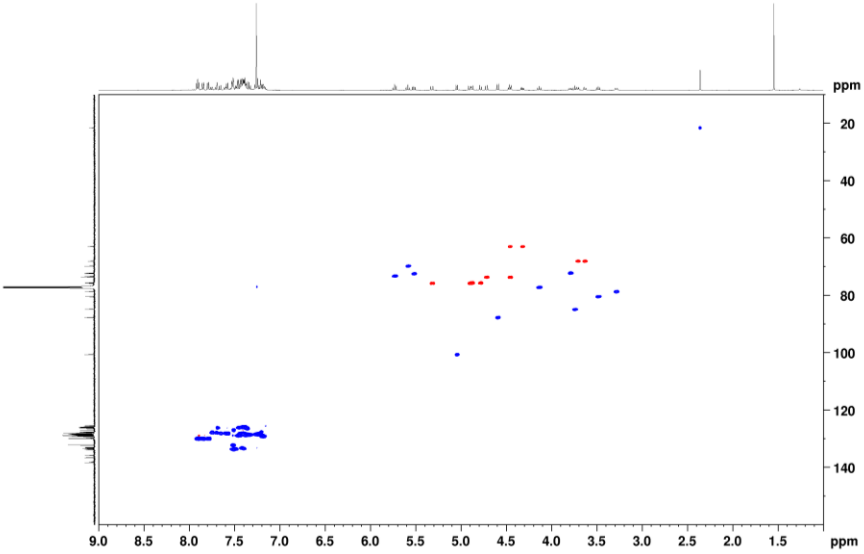

HMBC**:**

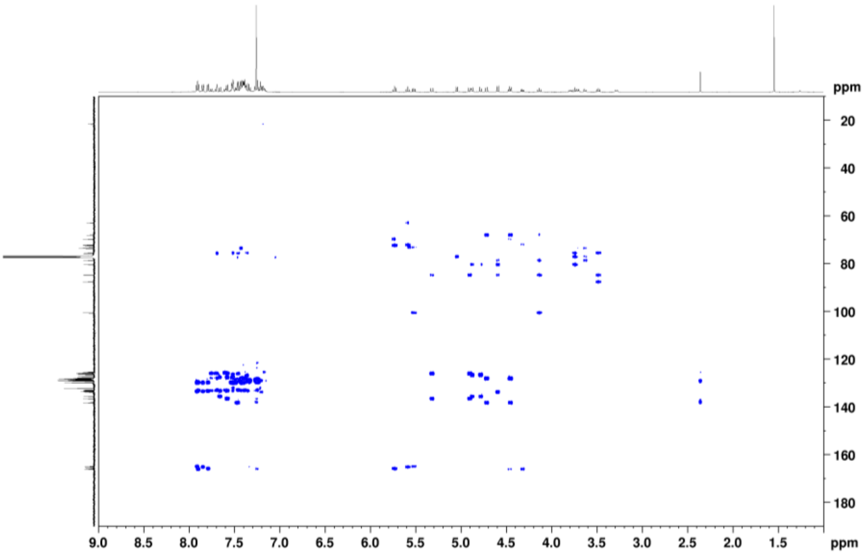

Thiophenyl *O*-(2,3,4,6-tetra-*O*-benzyl-β-D-glucopyranosyl)-(1→4)-6-*O*-benzyl-2,3-*O*-2-naphthylmethyl-β-D-glucopyranoside (17):

^{1}H NMR (500.20 MHz, $CDCl_3$, 25°C):

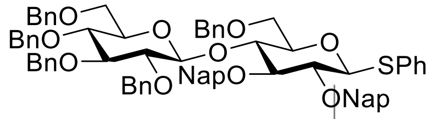

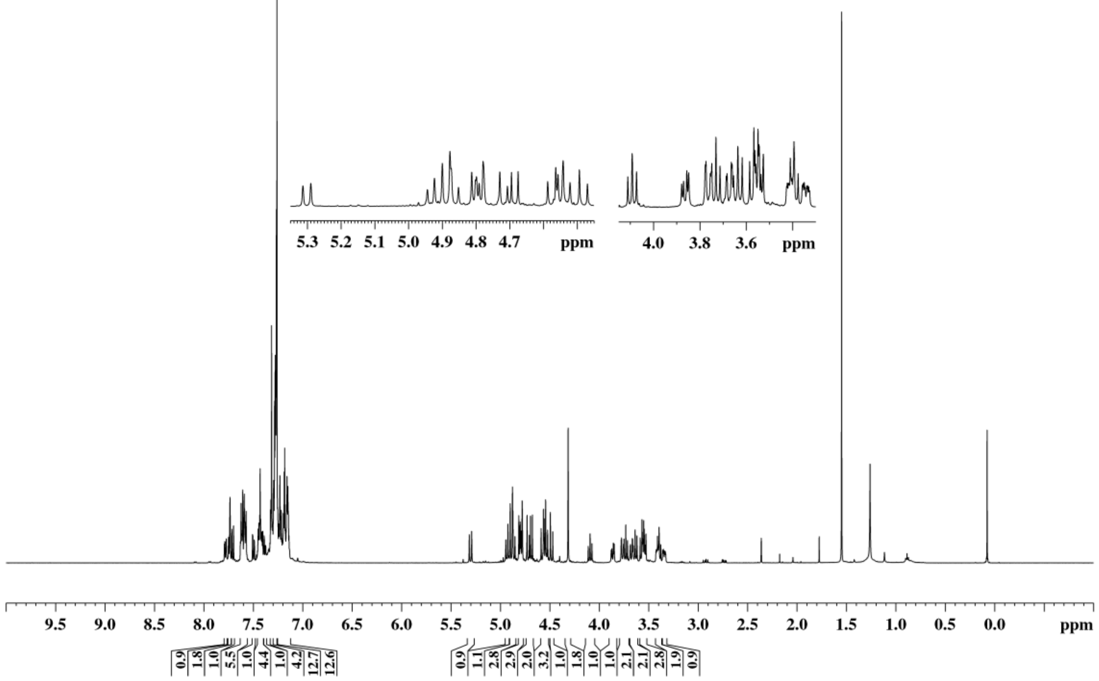

^{13}C{^{1}H} NMR (125.8 MHz, $CDCl_3$, 25°C):

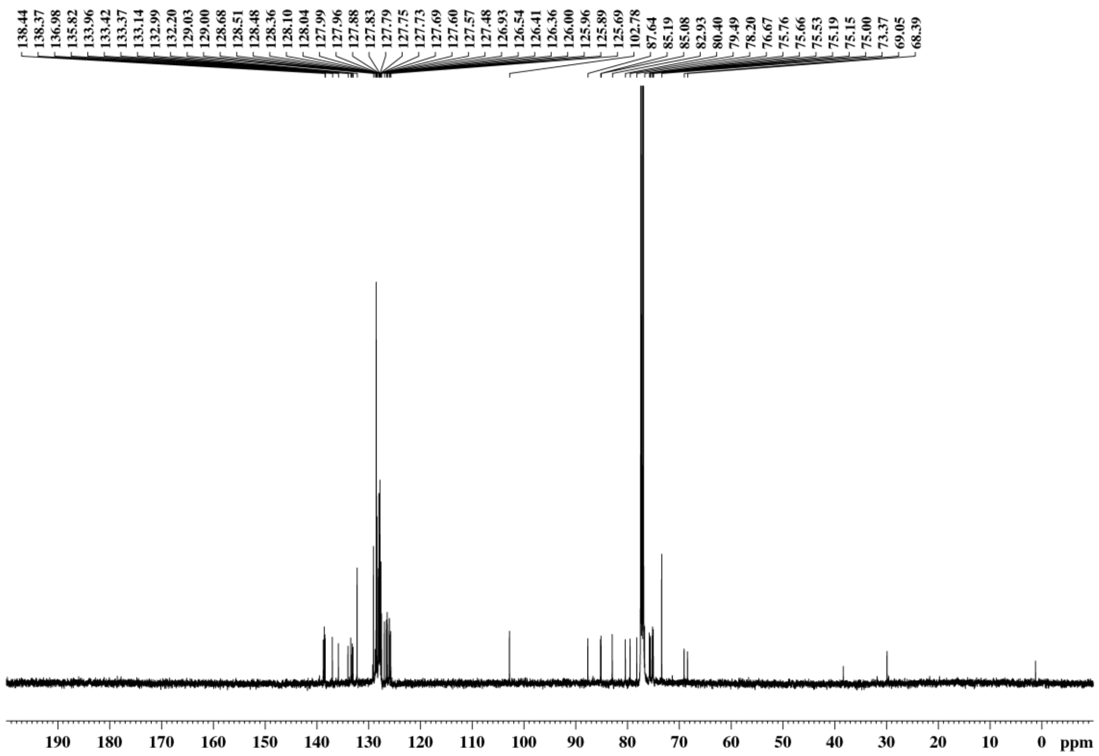

DQF-COSY:

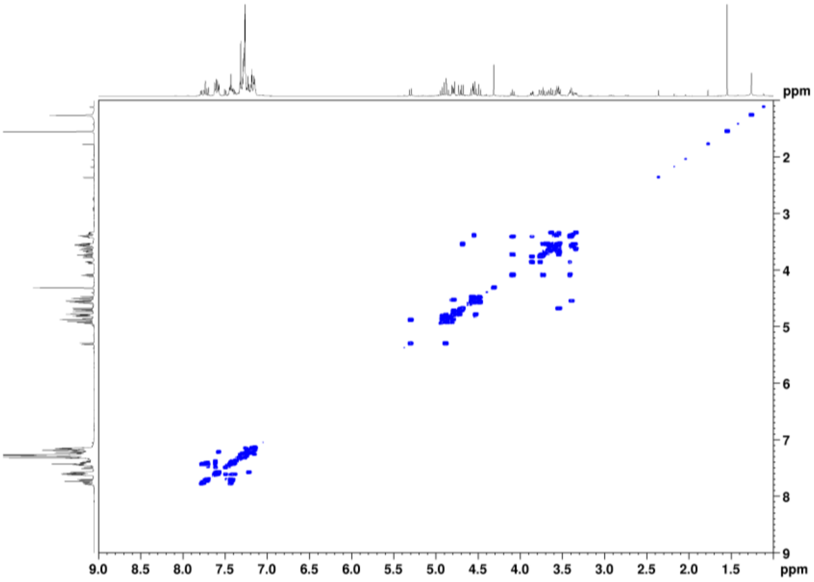

Multiplicity edited HSQC (CH and CH_3 positive, CH_2 negative):

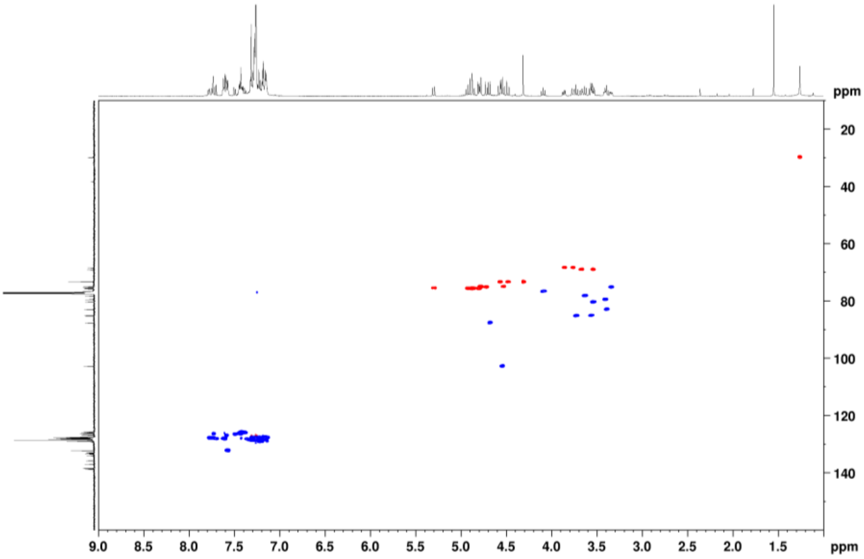

HMBC:

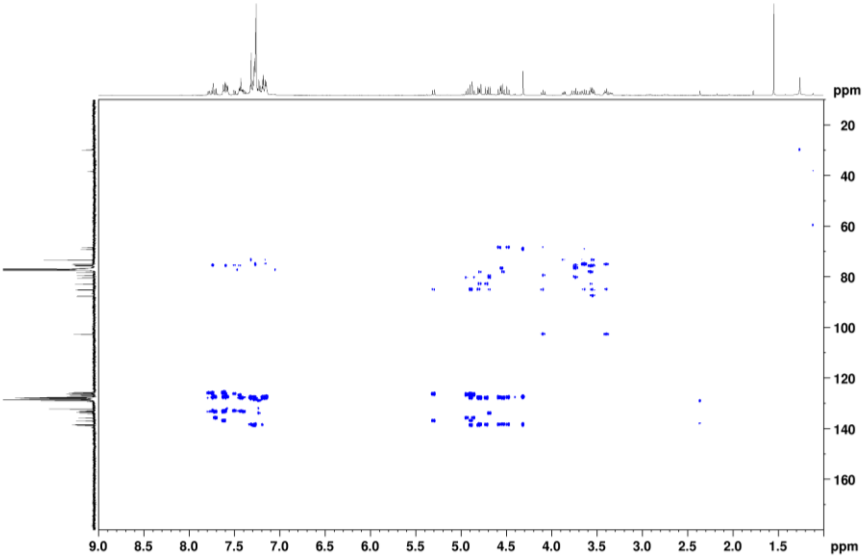

Thiophenyl *O*-(2,3,4,6-tetra-*O*-benzyl-β-D-glucopyranosyl)-(1→4)-6-*O*-benzyl-β-D-glucopyranoside (18):

^{1}H NMR (500.20 MHz, $CDCl_3$, 25°C):

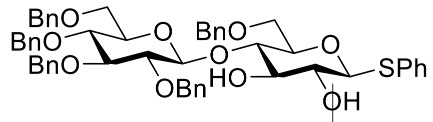

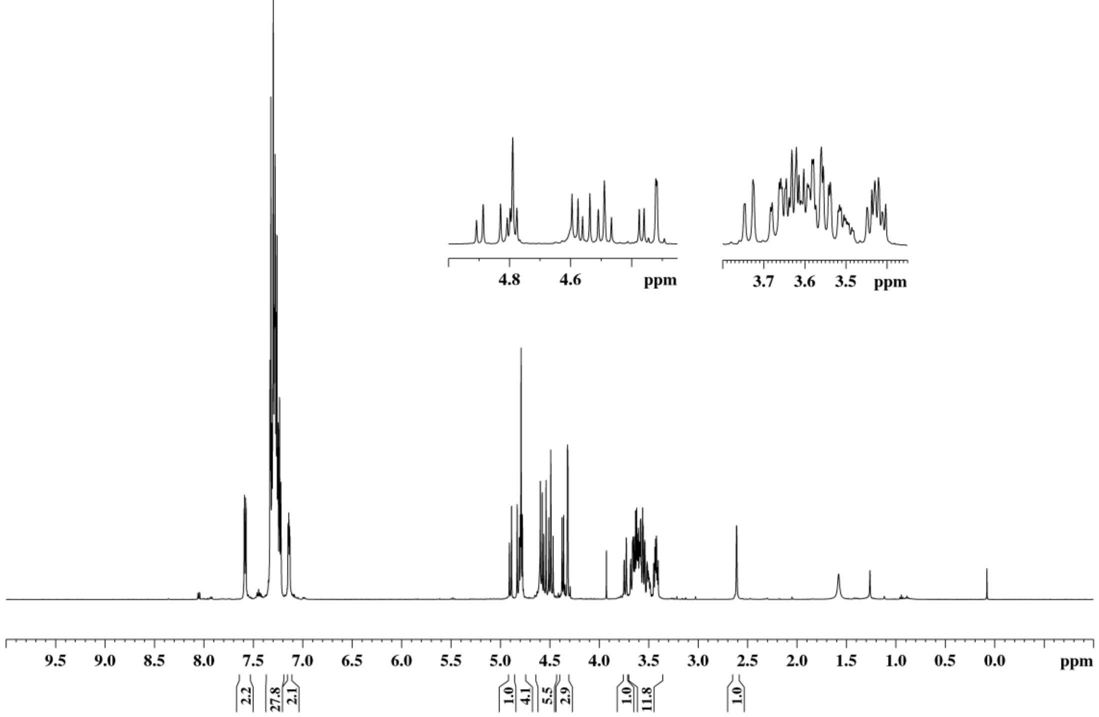

^{13}C{^{1}H} NMR (125.8 MHz, $CDCl_3$, 25°C):

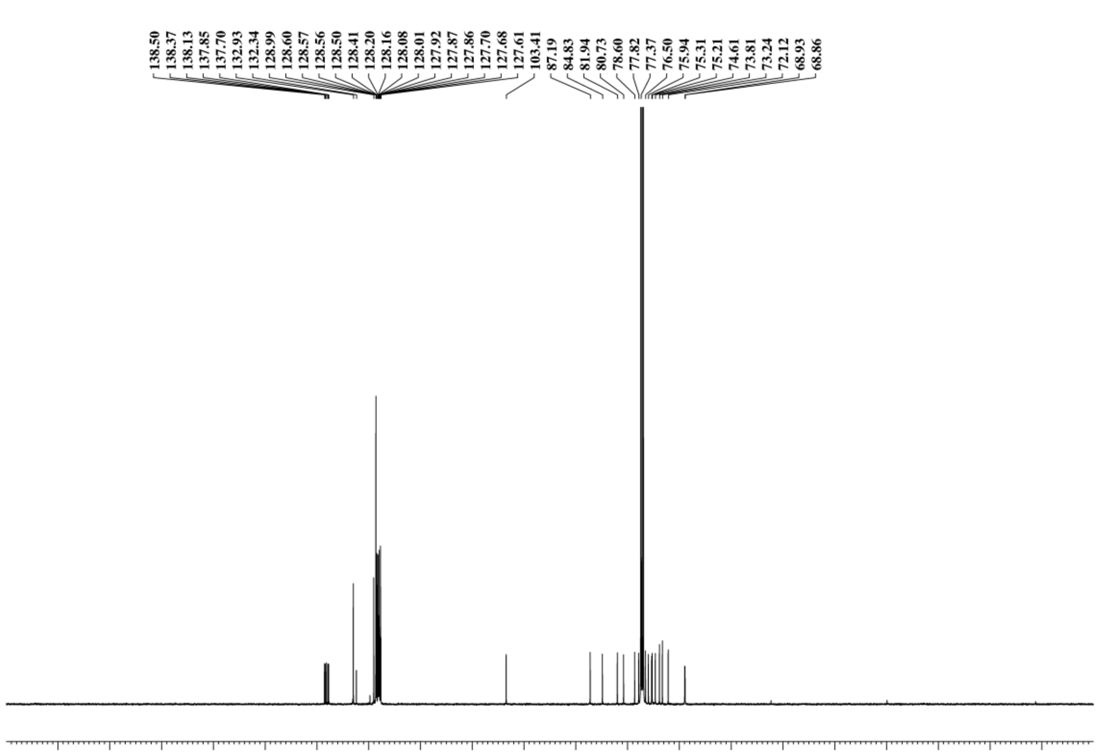

DQF-COSY:

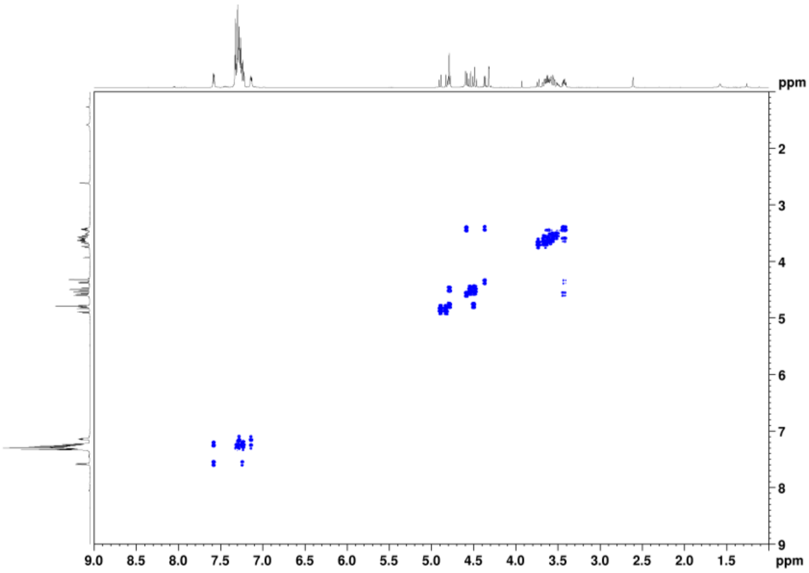

Multiplicity edited HSQC (CH and CH_3 positive, CH_2 negative):

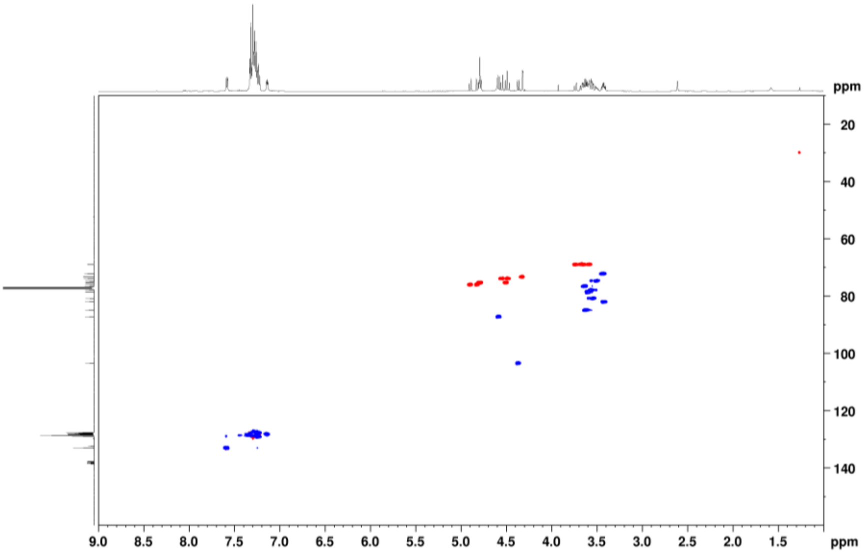

HMBC**:**

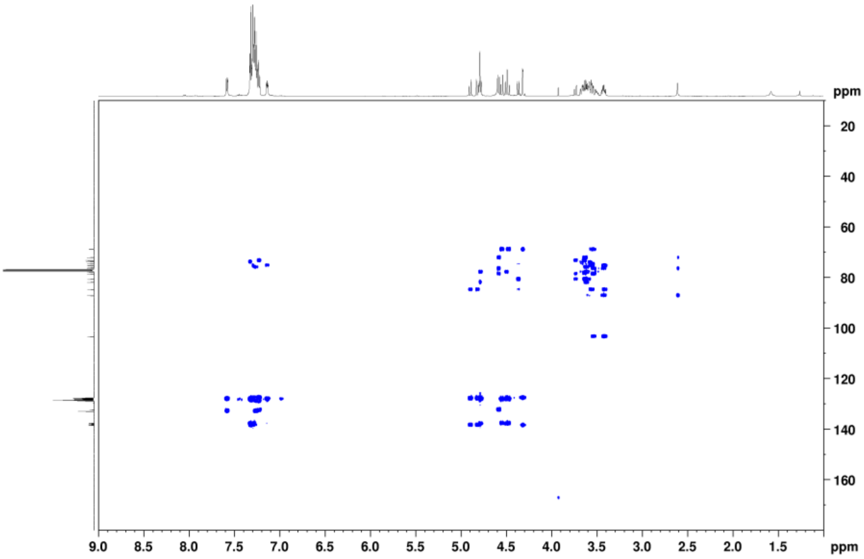

Thiophenyl *O*-(2,3,4,6-tetra-*O*-benzyl-β-D-glucopyranosyl)-(1→4)-2,3-di-*O*-acetyl-6-*O*-benzyl-β-D-glucopyranoside (19):

^{1}H NMR (500.20 MHz, $CDCl_3$, 25°C):

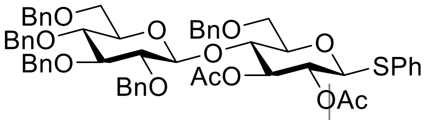

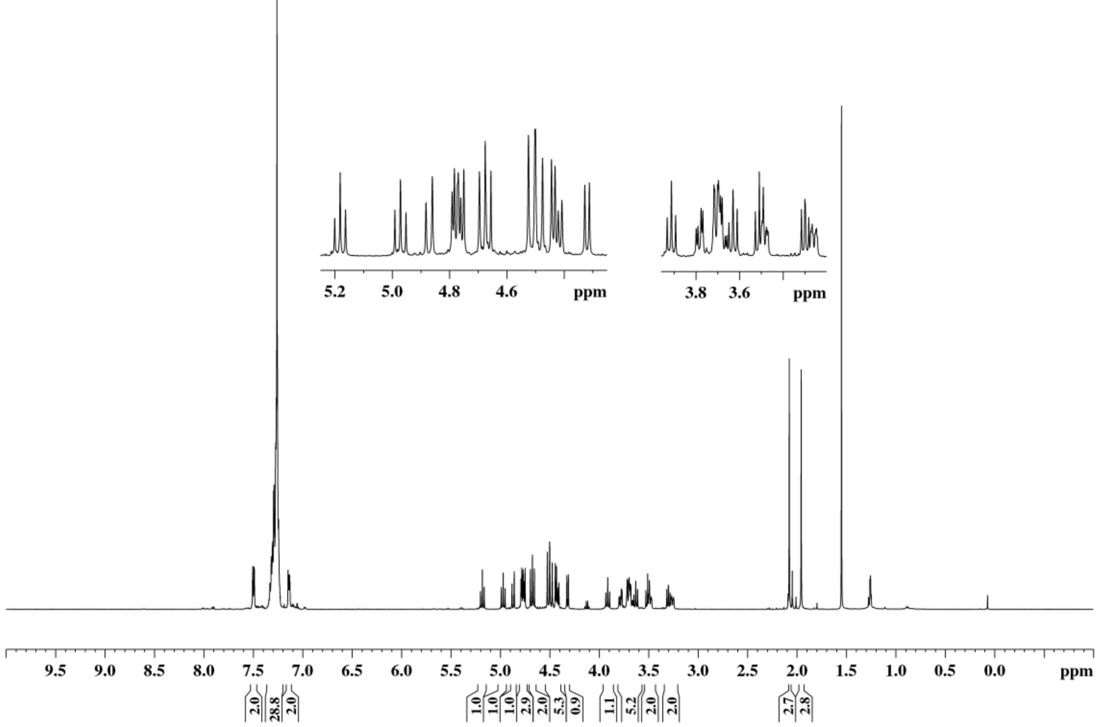

^{13}C{^{1}H} NMR (125.8 MHz, $CDCl_3$, 25°C):

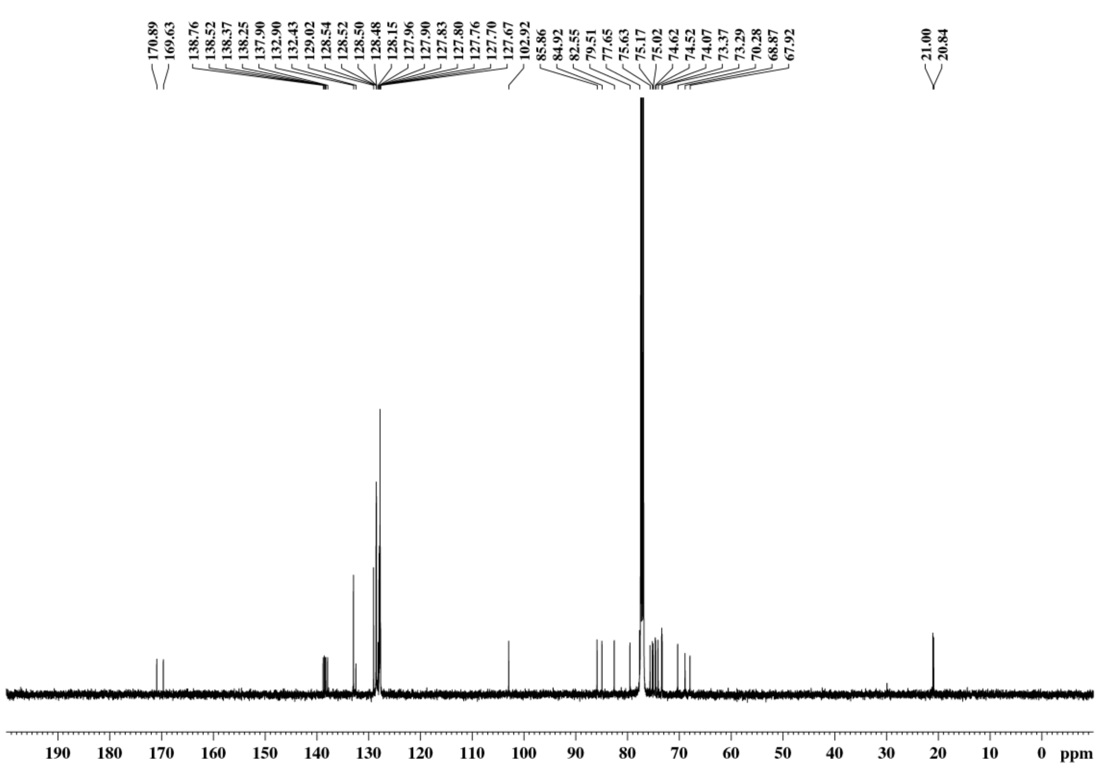

DQF-COSY:

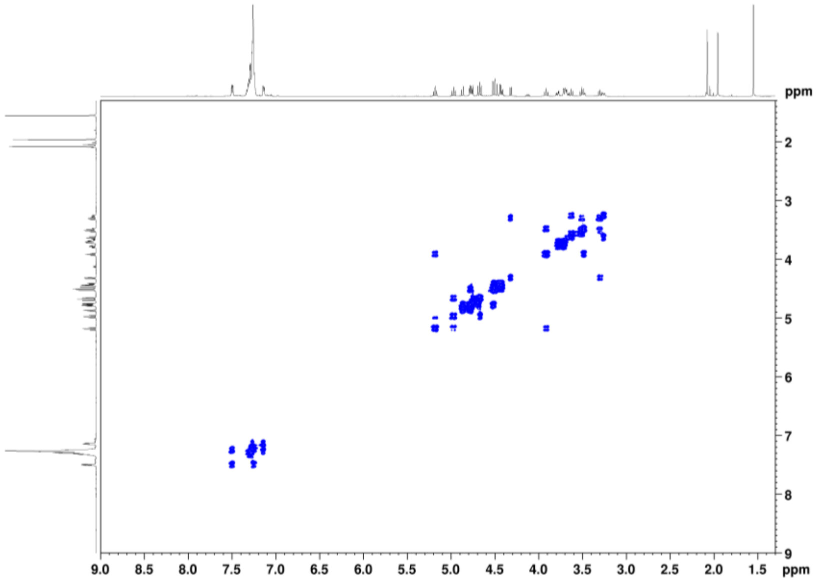

Multiplicity edited HSQC (CH and CH_3 positive, CH_2 negative):

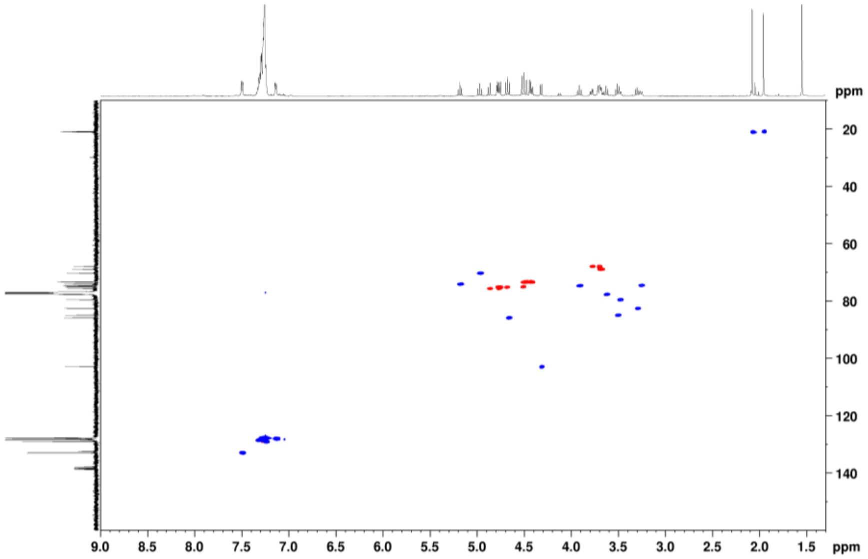

HMBC**:**

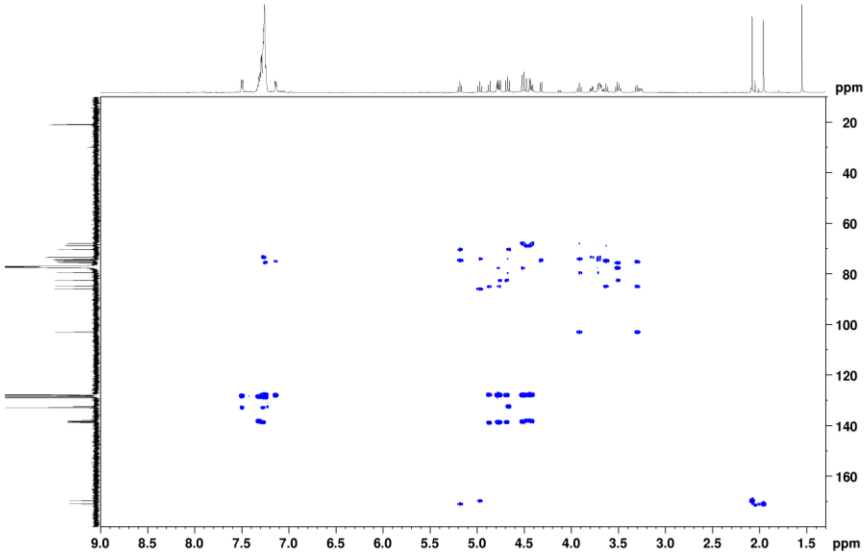

Thiophenyl *O*-(2,3,4,6-tetra-*O*-benzyl-β-D-glucopyranosyl)-(1→4)-*O*-2-*O*-acetyl-6-*O*-benzyl-β-D-glucopyranoside (20):

^{1}H NMR (500.20 MHz, $CDCl_3$, 25°C):

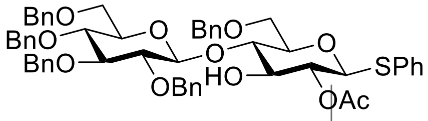

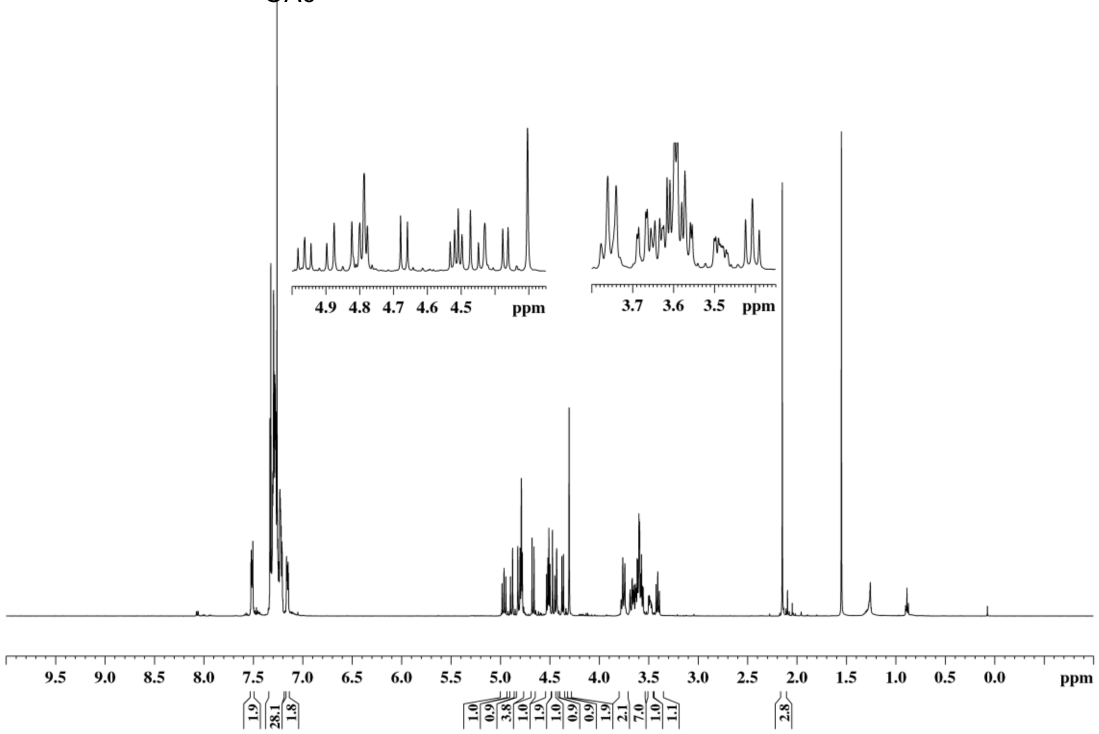

^{13}C{^{1}H} NMR (125.8 MHz, $CDCl_3$, 25°C):

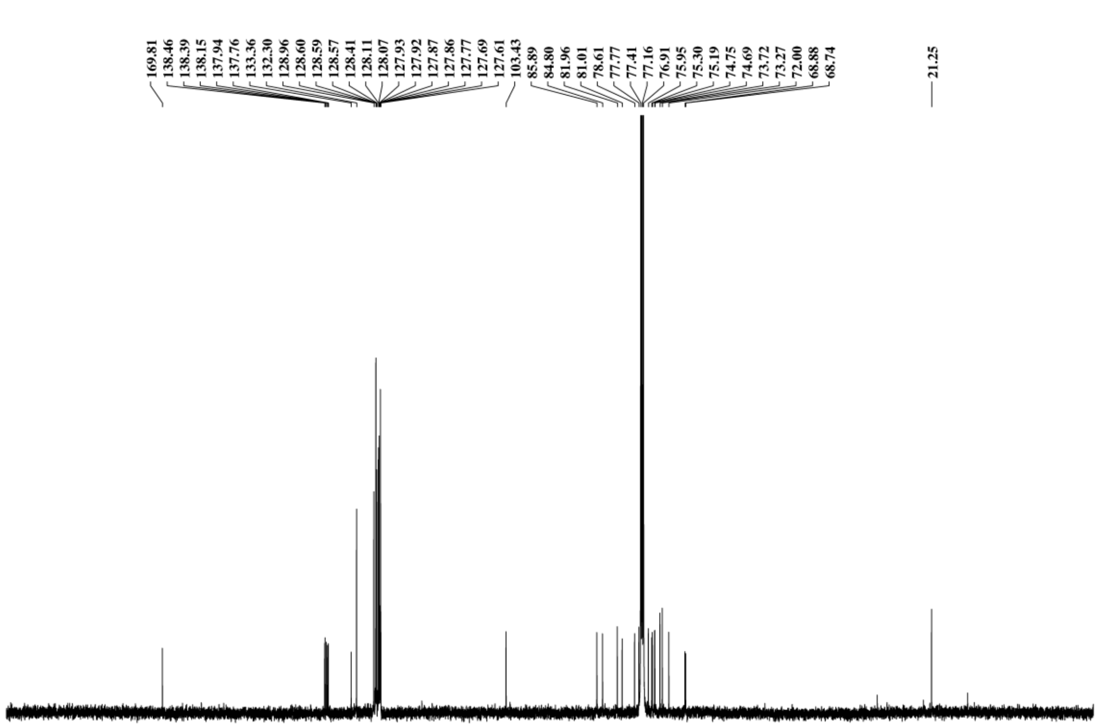

DQF-COSY:

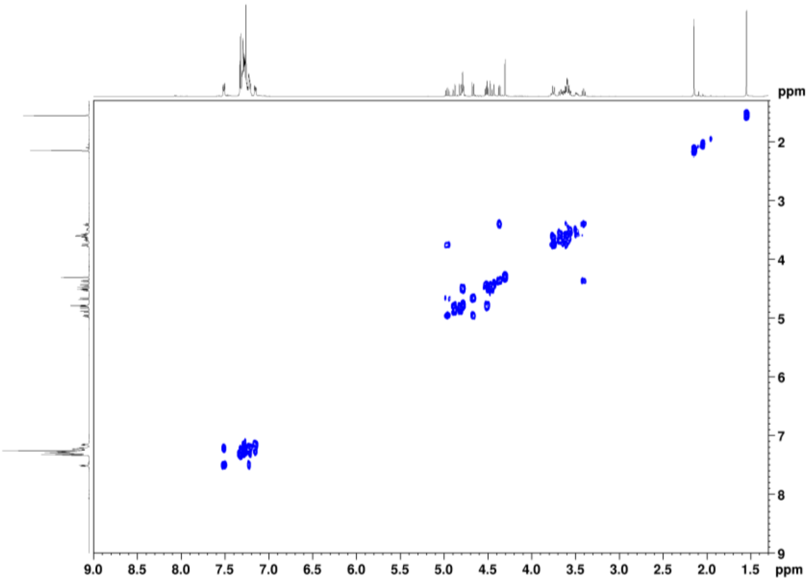

Multiplicity edited HSQC (CH and CH_3 positive, CH_2 negative):

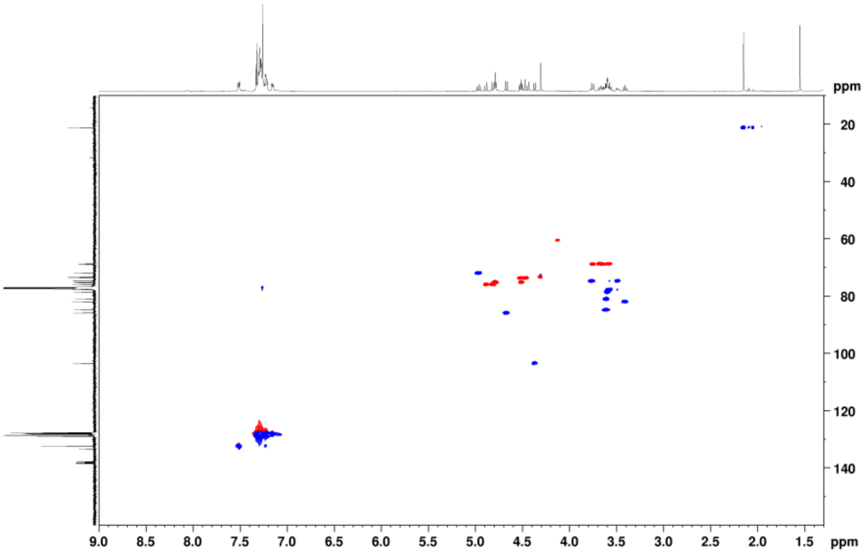

HMBC:

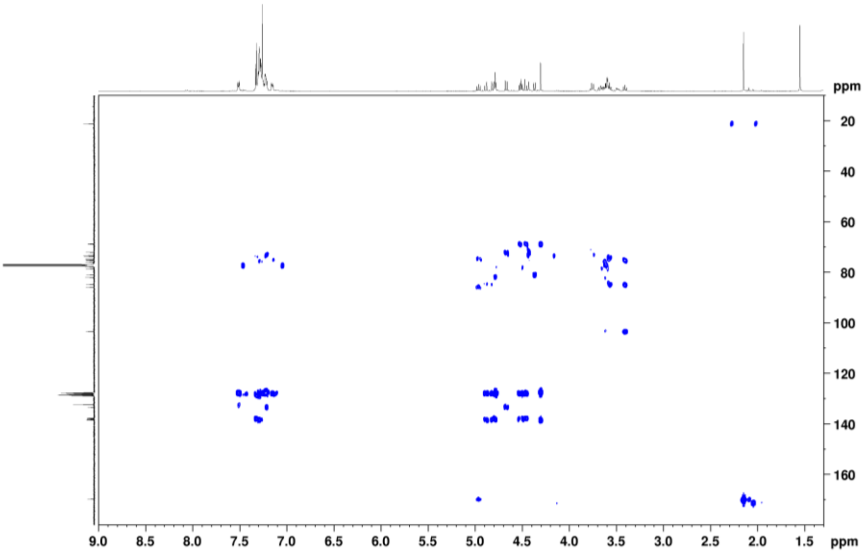

Methyl *O*-(2,3,4,6-tetra-*O*-benzyl-β-D-glucopyranosyl)-(1→4)-*O*-(2,3-di-*O*-acetyl-6-*O*-benzyl-β-D-glucopyranosyl)-(1→4)-2,3,6-tri-*O*-benzyl-α-D-glucopyranoside (22):

^{1}H NMR (500.20 MHz, $CDCl_3$, 25°C):

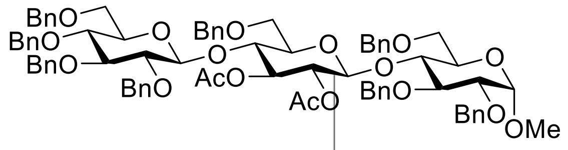

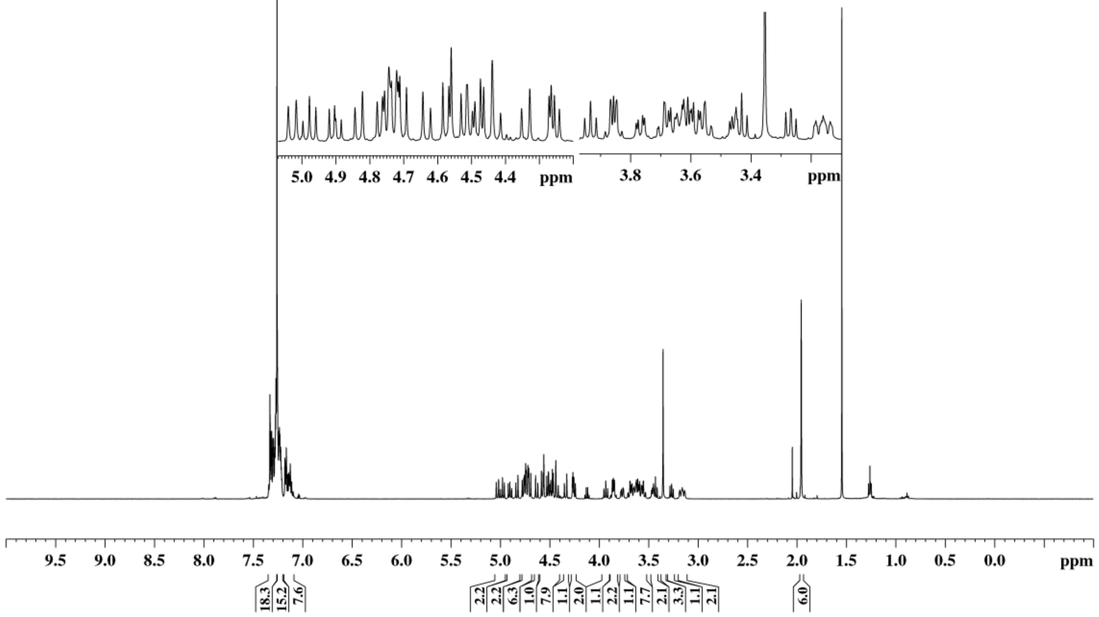

^{13}C{^{1}H} NMR (125.8 MHz, $CDCl_3$, 25°C):

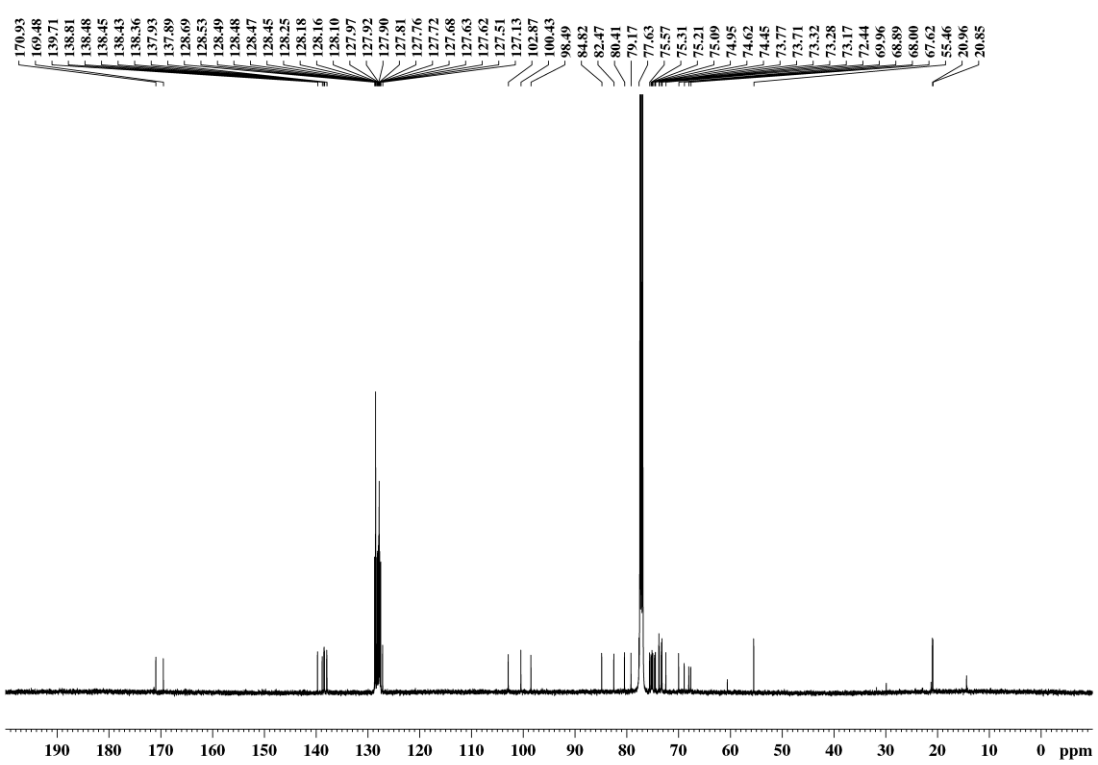

DQF-COSY:

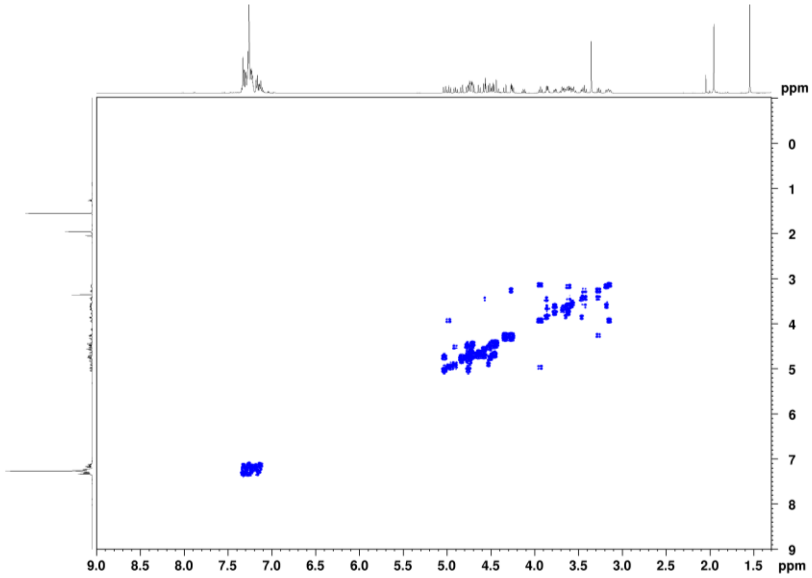

Multiplicity edited HSQC (CH and CH_3 positive, CH_2 negative):

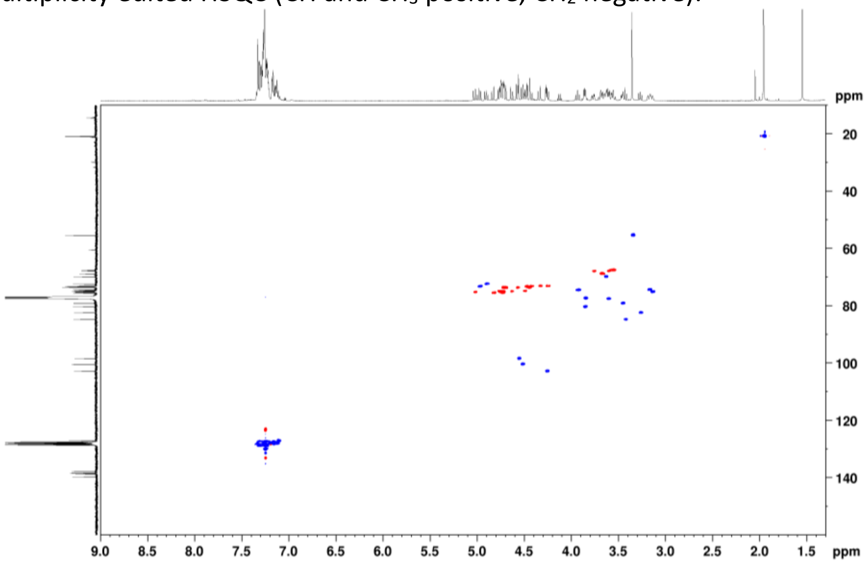

HMBC**:**

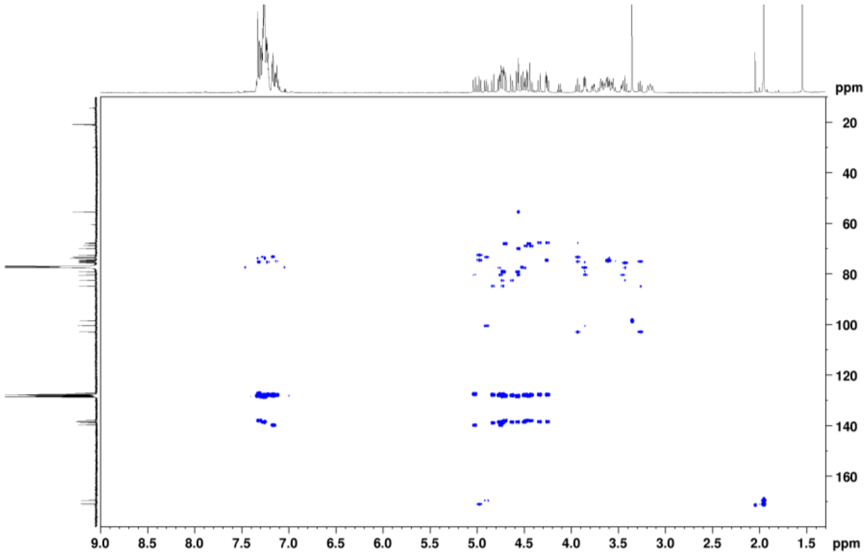

Methyl *O*-(2,3,4,6-tetra-*O*-benzyl-β-D-glucopyranosyl)-(1→4)-*O*-(2-*O*-acetyl-6-*O*-benzyl-β-D-glucopyranosyl)-(1→4)-2,3,6-tri-*O*-benzyl-α-D-glucopyranoside (23):

^{1}H NMR (500.20 MHz, $CDCl_3$, 25°C):

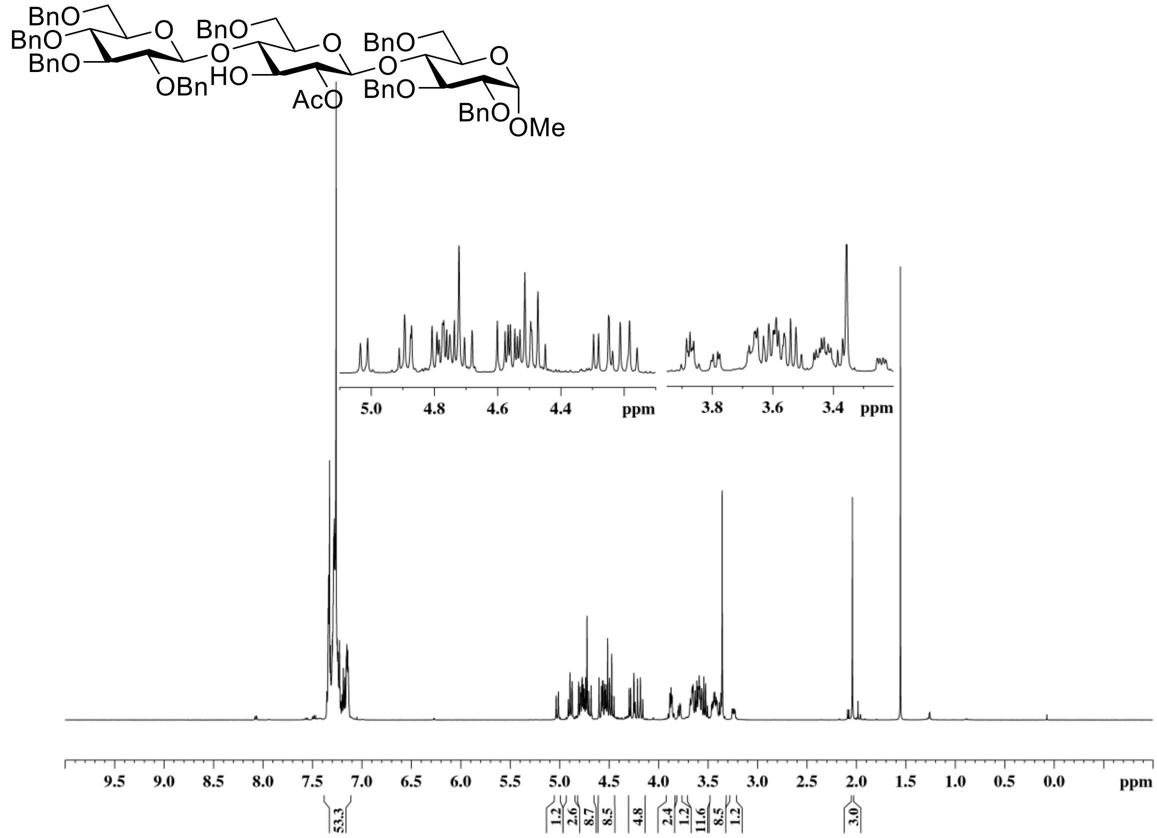

^{13}C{^{1}H} NMR (125.8 MHz, $CDCl_3$, 25°C):

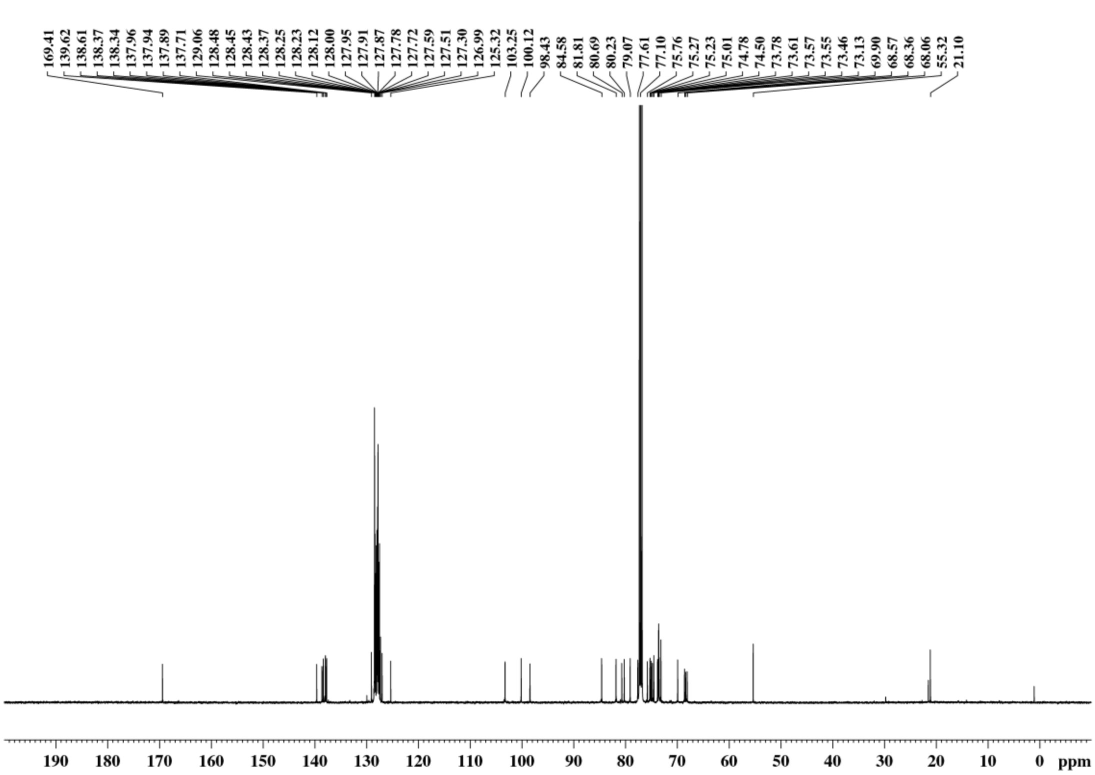

DQF-COSY:

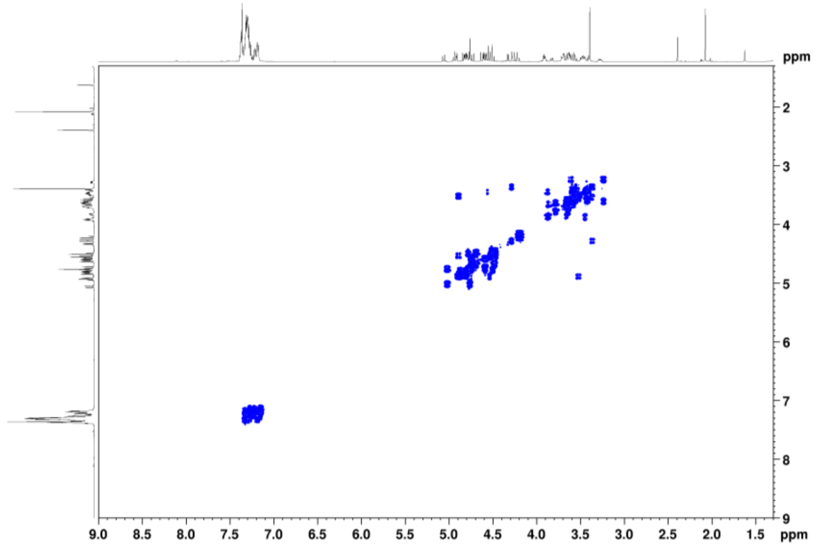

Multiplicity edited HSQC (CH and CH_3 positive, CH_2 negative):

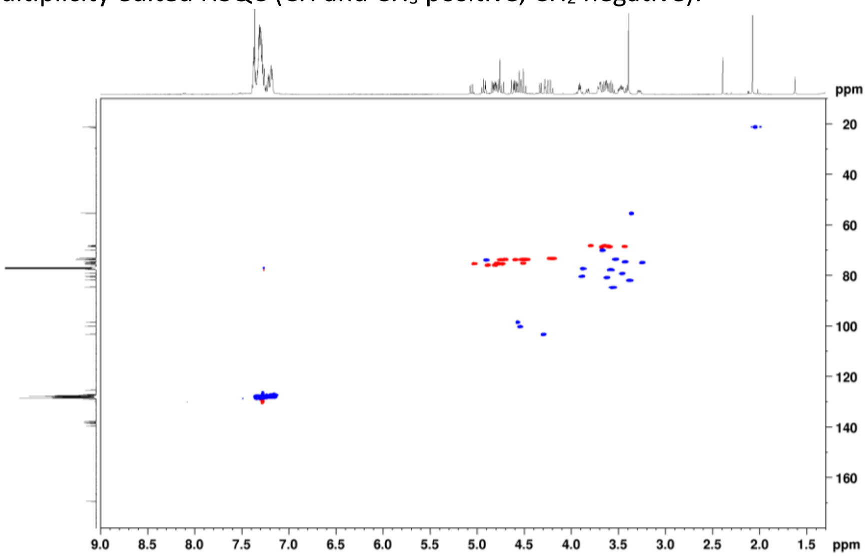

HMBC**:**

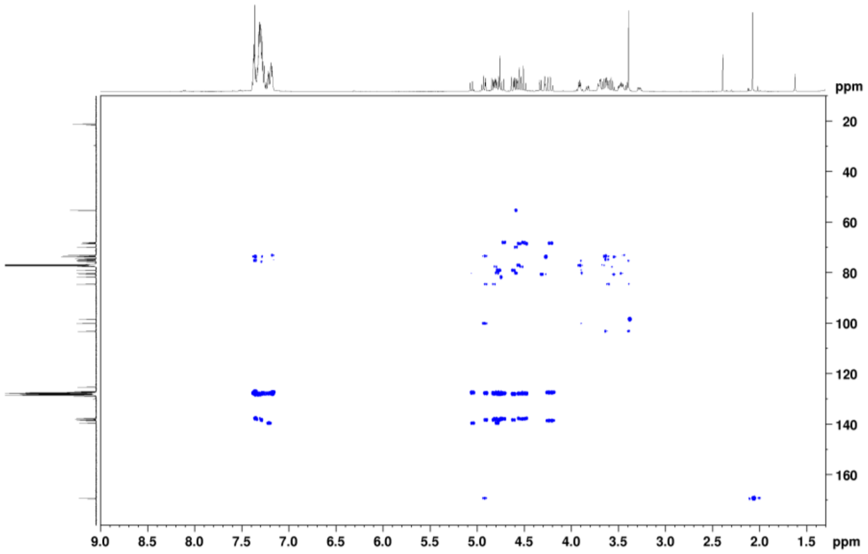

Methyl *O*-(β-D-glucopyranosyl)-(1→4)-*O*-(2,3-di-*O*-acetyl-β-D-glucopyranosyl)-(1→4)-α-D-glucopyranoside (2a):

^{1}H NMR (500.20 MHz, MeOD, 25°C):

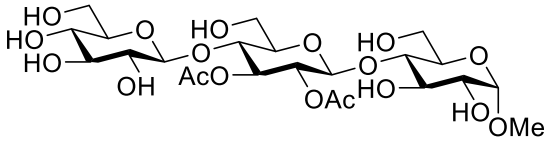

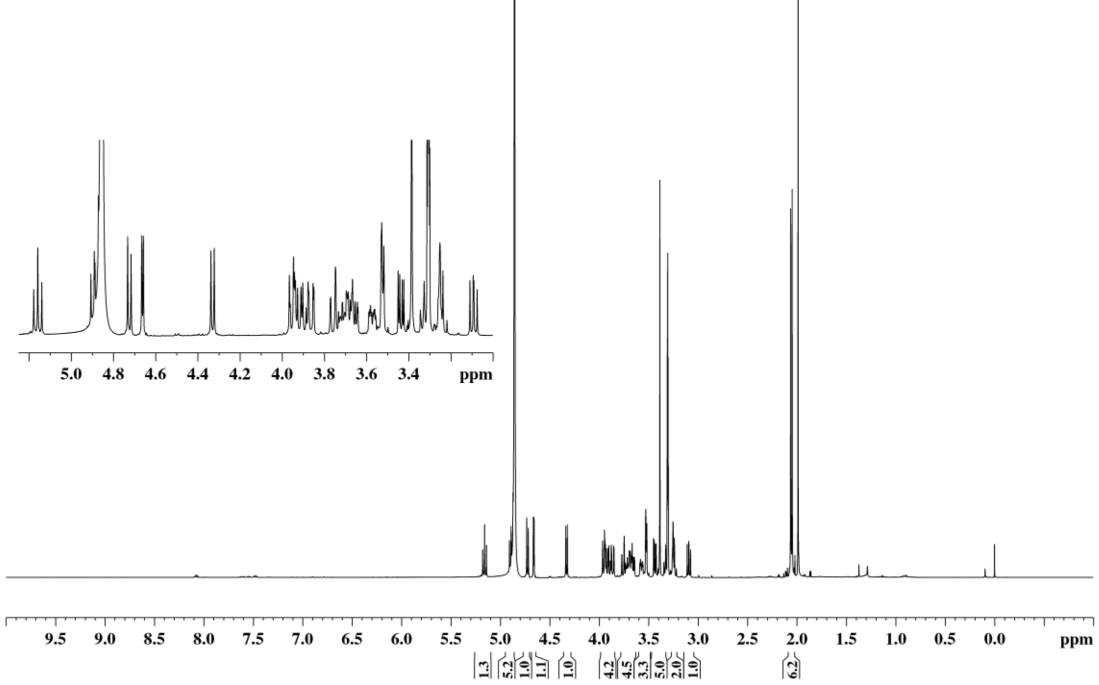

$^{13}C\{^{1}H\}$ NMR (125.8 MHz, MeOD, 25°C):

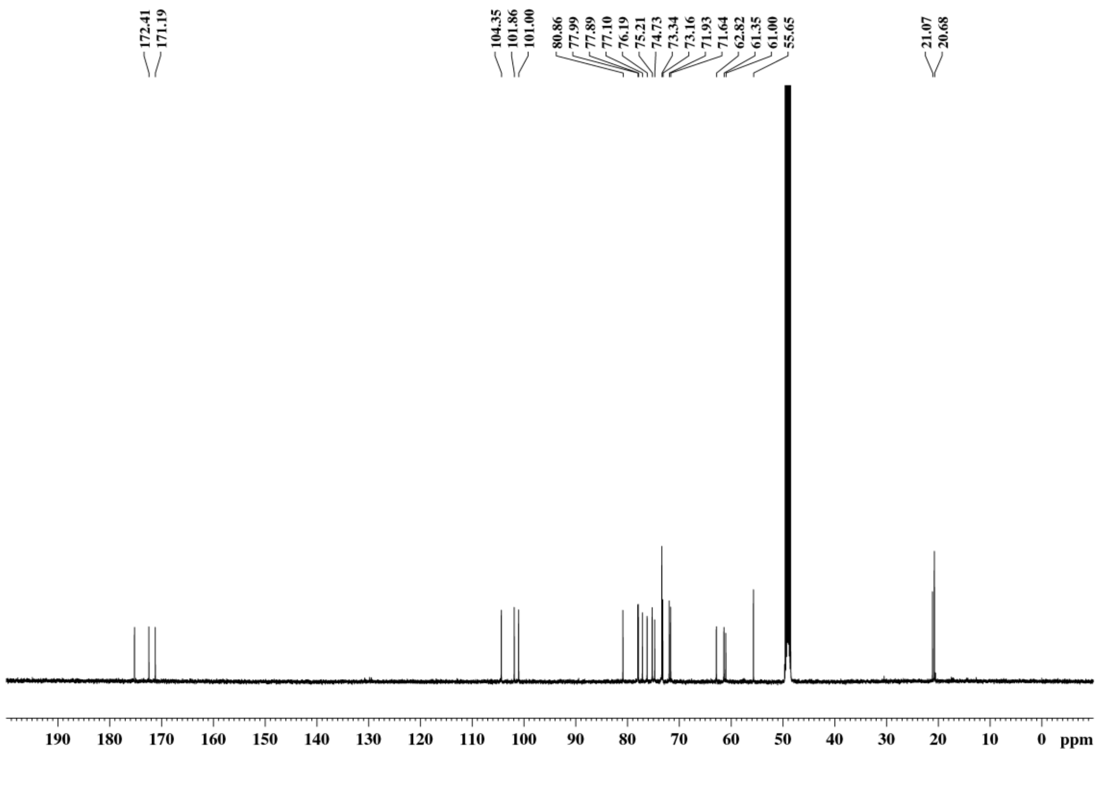

DQF-COSY:

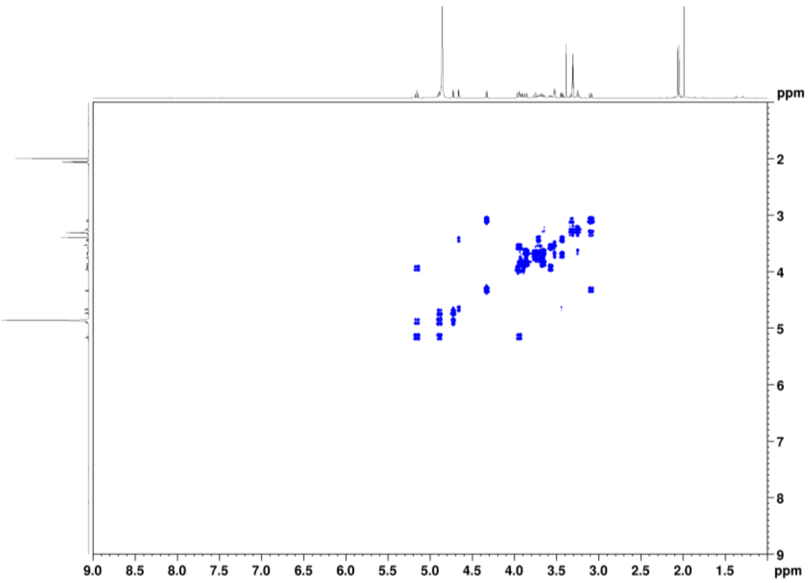

Multiplicity edited HSQC (CH and CH_3 positive, CH_2 negative):

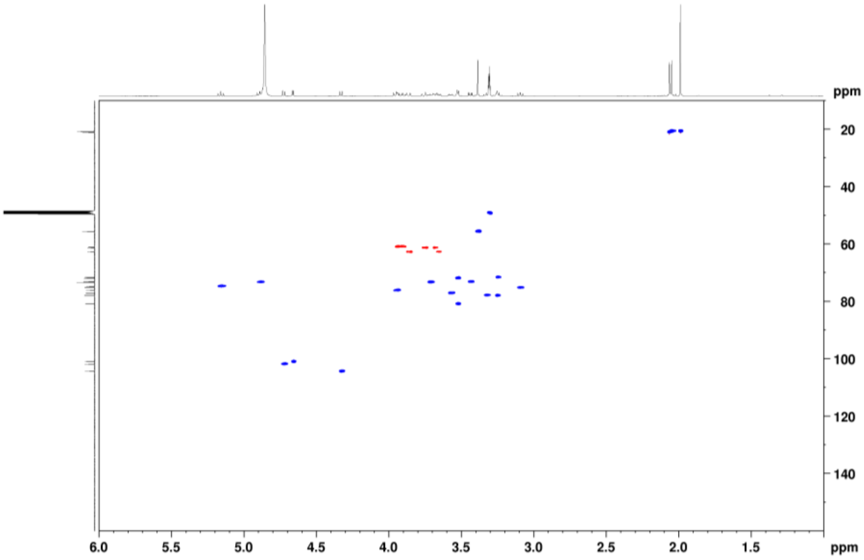

HMBC**:**

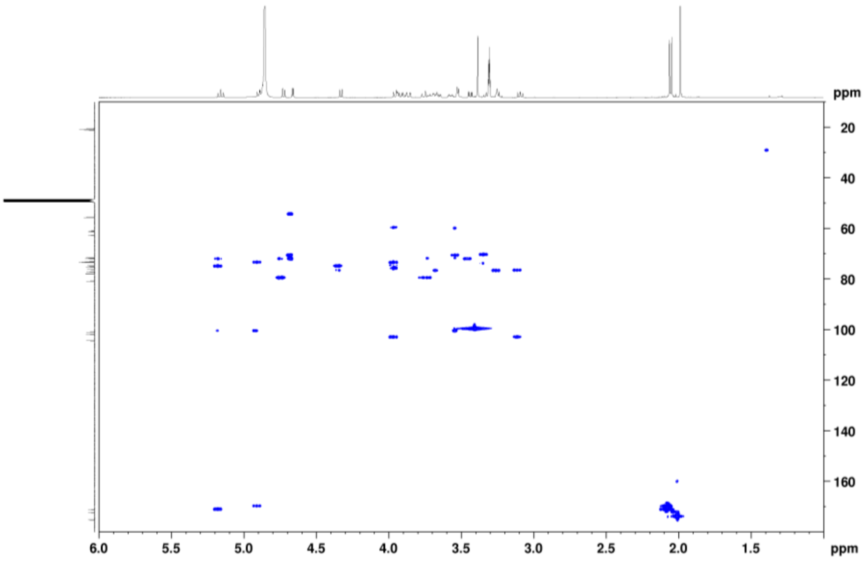

Methyl *O*-(β-D-glucopyranosyl)-(1→4)-*O*-(2-*O*-acetyl-β-D-glucopyranosyl)-(1→4)-α-D-glucopyranoside (2e):

^{1}H NMR (500.20 MHz, MeOD, 25°C):

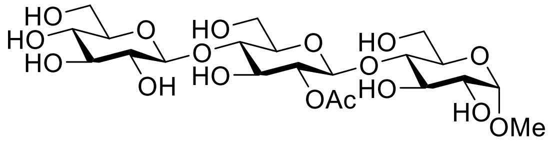

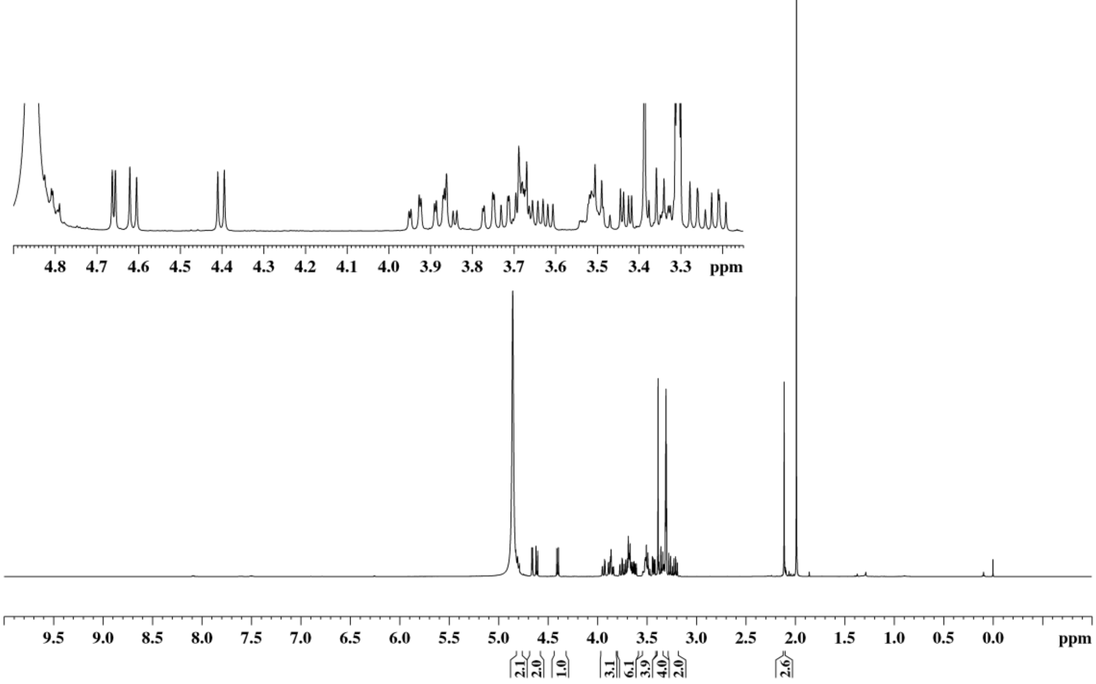

^{13}C{^{1}H} NMR (125.8 MHz, MeOD, 25°C):

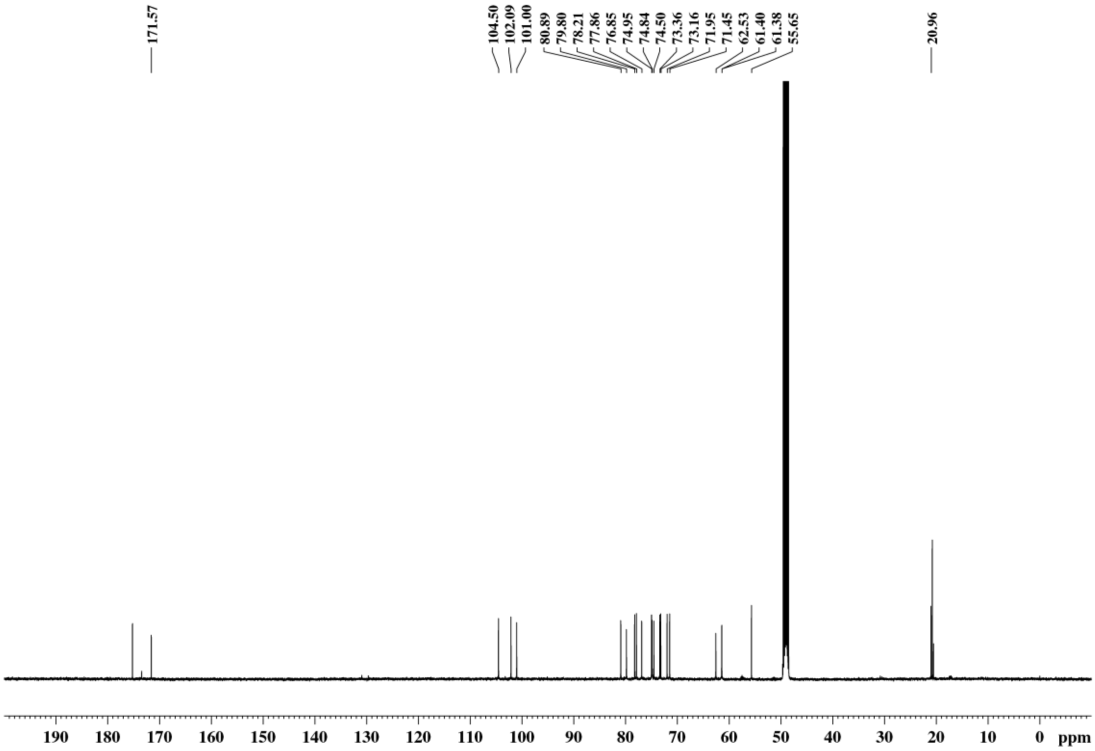

DQF-COSY:

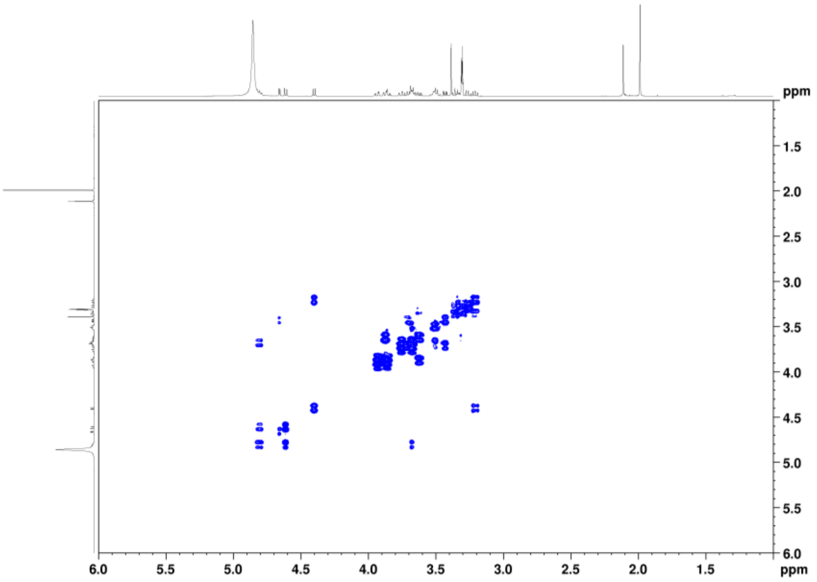

Multiplicity edited HSQC (CH and CH_3 positive, CH_2 negative):

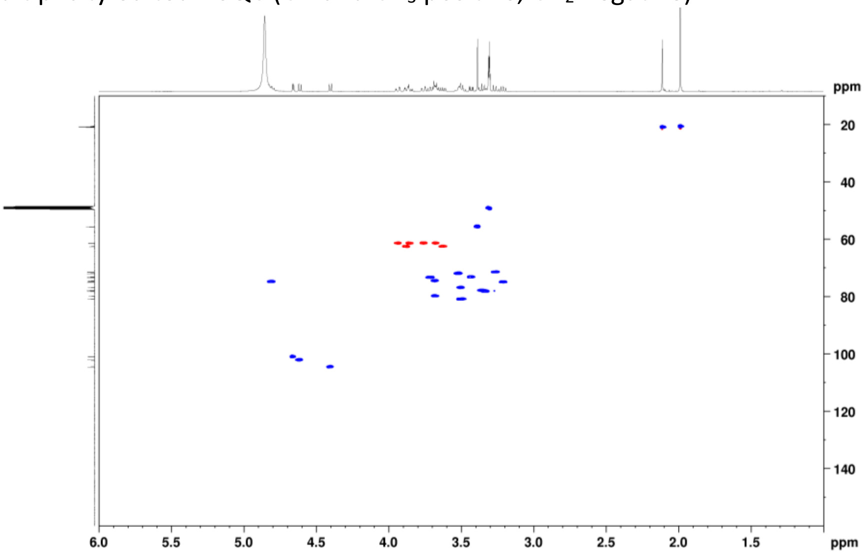

HMBC:

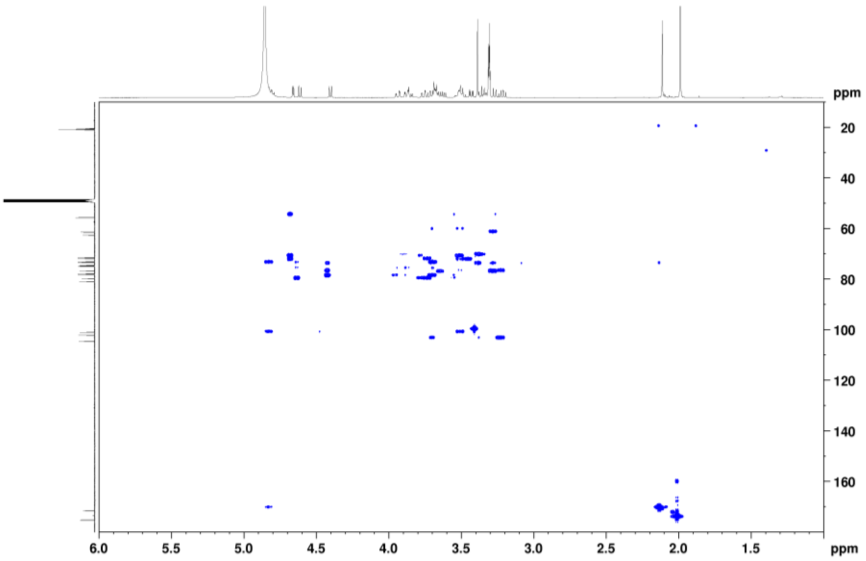

Migration data

Changes in pH

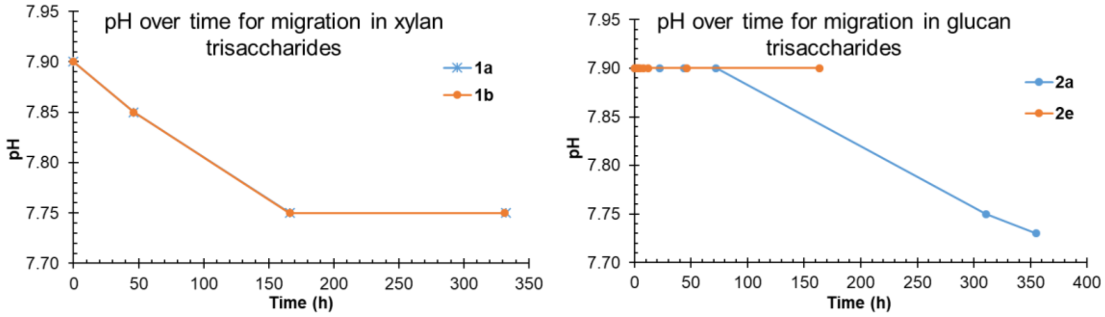

Figure S1. The pH over time for the xylan and glucan trisaccharides.

Experimental data points

Xylan trisaccharides

Table S1. The extracted data points with the ratio of trisaccharides for the migration starting form **1a**.

Time (h)	1a data	1b data	1c data	1d data
**0.0**	0.9827	0.0045	0.0129	0
**46.1**	0.8316	0.056	0.0976	0.0148
**166.5**	0.6115	0.1222	0.225	0.0413
**332.3**	0.3684	0.1854	0.3532	0.093

Table S2. The extracted data points with the ratio of trisaccharides for the migration starting form **1b**.

Time (h)	1b data	1c data	1d data
**0**	0.8685	0.1315	0
**0.5**	0.6668	0.3313	0.0019
**1**	0.5426	0.4573	0.0001
**1.5**	0.4666	0.5325	0.0009
**2**	0.4175	0.581	0.0015
**2.5**	0.3828	0.6121	0.0051
**3.0**	0.3658	0.6285	0.0057
**4.0**	0.3447	0.6452	0.0102
**5.0**	0.3381	0.6497	0.0122
**6.0**	0.3320	0.6521	0.0159
**8.0**	0.3298	0.6481	0.0221
**10.0**	0.3285	0.6437	0.0278
**12.0**	0.3276	0.638	0.0345
**14.0**	0.3280	0.6363	0.0357
**46.1**	0.2989	0.5641	0.1370
**166.3**	0.2227	0.4104	0.3670
**331.6**	0.1488	0.2718	0.5794

Glucan trisaccharides

Table S3. The extracted data points with the ratio of trisaccharides for the migration starting form **2a**.

Time (h)	2a data	2b data	2c data	2d data	2e data	2f data	2g data
**0**	0.9874	0	0	0.0062	0.0064	0	0
**22.3**	0.4120	0.2349	0.2359	0.0283	0.0287	0.0603	0
**43.7**	0.2006	0.3028	0.3102	0.0423	0.0433	0.1008	0
**72.2**	0.0823	0.3097	0.3157	0.0576	0.0578	0.1740	0.0029
**310.7**	0.0081	0.1332	0.1312	0.0528	0.0534	0.4030	0.2183
**355.3**	0.0007	0.1154	0.1162	0.0503	0.0513	0.4172	0.2489

Table S4. The extracted data points with the ratio of trisaccharides for the migration starting form **2e**.

Time (h)	2d data	2e data	2f data	2g data
**0**	0.0747	0.9253	0	0
**1.0**	0.3469	0.6469	0.0062	0
**2.0**	0.4144	0.5777	0.0079	0
**3.0**	0.4696	0.5127	0.0154	0.0022
**5.0**	0.4821	0.4875	0.0221	0.0083
**8.0**	0.4769	0.4800	0.0254	0.0176
**12.0**	0.4693	0.4701	0.0308	0.0297
**46.2**	0.3603	0.3667	0.1208	0.1522
**163.9**	0.2067	0.2109	0.2092	0.3732

Computational Studies

DFT calculations. General methods.

All of the calculations were performed using the Gaussian09 program.[9] In line with our previous paper on acyl migration in monosaccharides,[10] we consider a model with three explicit water molecules; geometry optimizations were done with wb97xd[11] functionals and basis set 6-31+G(d,p),[12,13] the former being more expeditious in convergence so, it was selected; the wb97xd/6-31+G(d,p) level is also recommended by previous calculations with thiols carried out in the presence of three explicit water molecules. Additional solvent effects were considered using the SMD model.[14] The nature of stationary points was defined based on calculations of normal vibrational frequencies (force constant Hessian matrix). The optimizations were carried out using the Berny analytical gradient optimization method.[15] Minimum energy pathways for the reactions studied were found by gradient descent of transition states in the forward and backward direction of the transition vector (IRC analysis).[16] Analytical second derivatives of the energy were calculated to classify the nature of every stationary point, to determine the harmonic vibrational frequencies, and to provide zero-point vibrational energy corrections. The thermal and entropic contributions to the free energies were also obtained from the vibrational frequency calculations, using the unscaled frequencies. Free energy calculations were calculated using a (99,590) grid to minimize errors.[17]

Since the observed process consists of the acyl group migration from the corresponding monosaccharide in its neutral form, formal direct $\Delta G_{1(obs)}$ and inverse $\Delta G_{-1(obs)}$ energy barriers in kcal/mol, have been obtained from the observed rate constants $k_{1(obs)}$ and $k_{--1(obs)}$ using the Eyring's equation (eq 1):

$$k_T = \frac{k_B T}{hc^o}\, e^{\frac{-\Delta G^0}{RT}} \qquad \text{(Eq. 1)}$$

where k_T: rate constant in s^{-1}. T: temperature in K. k_B: Boltzman's constant ($1.380662 \cdot 10^{-23}$ $J \cdot K^{-1}$). h: Planck's constant ($6.626176 \cdot 10^{-34}$ $J \cdot s^{-1}$). c^0: concentration. R: gas constant (0.001987 $kcal \cdot mol^{-1} \cdot K^{-1}$). ΔG^0: energy barrier in $kcal \cdot mol^{-1}$

Those values have been compared with the computed formal barriers $\Delta G_{1(calc)}$ and $\Delta G_{-1(calc)}$ which have been calculated assuming that protonation/deprotonation processes are fast; under such conditions, the energy barrier of the process depends on the two steps of the acyl migration. Essentially the same values were obtained by using eq.1 and canonical variational theory,[18,19] including partition coefficients (Eq. 2):

$$k_T = \frac{k_B T}{hc^o} \frac{Q_{TS}}{\Pi Q_R} e^{\frac{-\Delta G^0}{RT}} \qquad \text{(Eq. 2)}$$

where k_T: rate constant in s^{-1}. T: temperature in K. k_B: Boltzman's constant ($1.380662 \cdot 10^{-23}$ $J \cdot K^{-1}$). h: Planck's constant ($6.626176 \cdot 10^{-34}$ $J \cdot s^{-1}$). c^0: concentration. R: gas constant (0.001987 $kcal \cdot mol^{-1} \cdot K^{-1}$). ΔG^0: energy barrier in $kcal \cdot mol^{-1}$ Q_{TS}: partition coefficients of transition structure. Q_R: partition coefficients of reactant.

Kinetics

The analysis of the acyl migration through the different hydroxyl groups requires to consider all the species involved in the process. In our previous report, we demonstrated that among the two possible mechanisms, i.e., (i) a mechanism under neutral or slightly acidic conditions, and (ii) a mechanism under basic conditions in which the anion is formed in some extent, the former can be neglected. Consequently, a single migration can be described by Eq. 3:

$$k_i^{obs} = \frac{k_j^{anionic} \cdot K_{eq}^m}{[H]^+} \qquad \text{(Eq. 3)}$$

where k_i^{obs} is the rate constant observed experimentally, where $k_j^{anionic}$ is the formal rate constant of the anionic mechanism (which actually is a two-step process) and K_{eq}^m is the equilibrium constant corresponding to the pK_a of the involved hydroxyl group.

The processes illustrated in Schemes S3 and S4 experimentally showed the direct and reverse rate constants indicated in Table S5. Although, rate constants are directly related with energy barriers through Eqs. 1 and 2, discussions are usually preferred to be place on the basis of energy barriers for which DFT calculations of free energies can vary by as much as 5 kcal/mol, considering several orientations of the molecule; however, by using fine grids it is possible to reduce the differences around 1 – 2 kcal/mol. When translated to rate constants, increasing 2 kcal/mol the barrier for a constant of 0.878 s^{-1} results in a constant of 0.00315 s^{-1}. This exponential difference makes it very difficult to obtain accurate values of rate constants, particularly with carbohydrates that have a high conformational flexibility.

Table S5. Experimental rate constants and the corresponding formal energy barriers, calculated through the Eyring's equation for the acetyl migration in Me α-D-manno- and Me α-D-glucotrisaccharides at pH=8 (Scheme S3 and S4).

Me α-D-glucotrisaccharides				Me α-D-mannotrisaccharides			
rate constants (s^{-1})		energy barriers ($kcal \cdot mol^{-1}$)		rate constants (s^{-1})		energy barriers ($kcal \cdot mol^{-1}$)	
k_1^{obs}	9.25E-06	ΔG_1	24.3	k_1^{obs}	5.72E-07	ΔG_1	26.0
k_{-1}^{obs}	< 1.00E-14	ΔG_{-1}	>36.0	k_{-1}^{obs}	< 1.00E-14	ΔG_{-1}	>36.0
k_2^{obs}	1.37E-04	ΔG_2	22.7	k_2^{obs}	5.22E-04	ΔG_2	21.9
k_{-2}^{obs}	1.34E-04	ΔG_{-2}	22.7	k_{-2}^{obs}	2.81E-04	ΔG_{-2}	22.3
k_3^{obs}	1.37E-04	ΔG_3	22.7	k_3^{obs}	5.22E-04	ΔG_3	21.9
k_{-3}^{obs}	1.34E-04	ΔG_{-3}	22.7	k_{-3}^{obs}	2.81E-04	ΔG_{-3}	22.3
k_4^{obs}	1.35E-06	ΔG_4	25.5	k_4^{obs}	5.72E-07	ΔG_4	26.0
k_{-4}^{obs}	< 1.00E-14	ΔG_{-4}	>36.0	k_{-4}^{obs}	< 1.00E-14	ΔG_{-4}	> 36.0

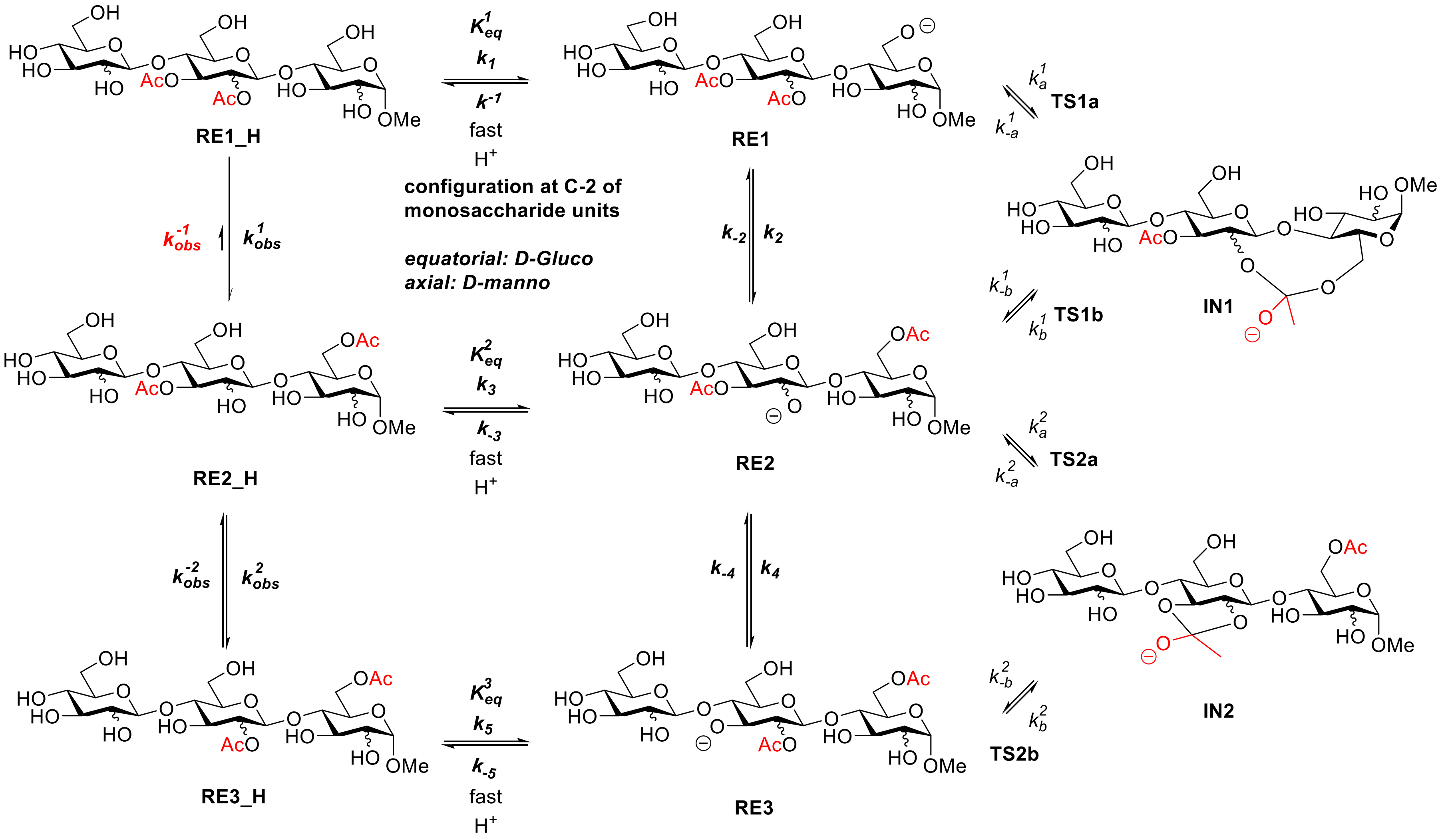

Scheme S3. Acetyl migration in diacetylated Me α-D-manno- and Me α-D-glucotrisaccharides.

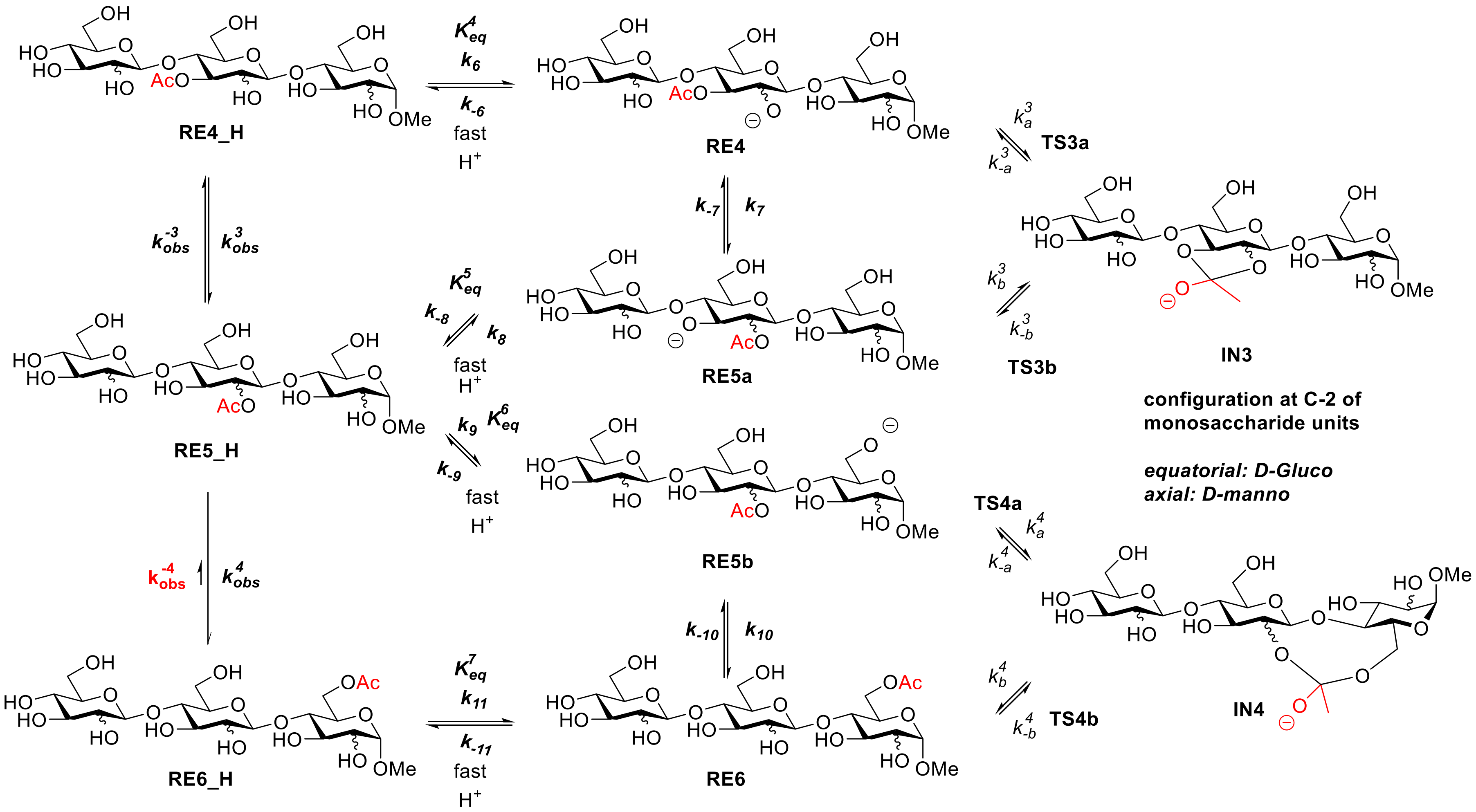

Scheme S4. Acetyl migration in monoacetylated Me α-D-manno- and Me α-D-glucotrisaccharides.

Following the same treatment given in our previous article, the equations governing the kinetics for the processes illustrated in Schemes S3 and S4 are:

$$k_1^{obs} = \frac{k_2 \cdot K_{eq}^1}{[H]^+} \quad \text{(Eq. 3)}$$

$$k_{-1}^{obs} = \frac{k_{-2} \cdot K_{eq}^2}{[H]^+} \quad \text{(Eq. 5)}$$

$$k_2^{obs} = \frac{k_4 \cdot K_{eq}^2}{[H]^+} \quad \text{(Eq. 6)}$$

$$k_{-2}^{obs} = \frac{k_{-4} \cdot K_{eq}^3}{[H]^+} \quad \text{(Eq. 7)}$$

$$k_3^{obs} = \frac{k_7 \cdot K_{eq}^4}{[H]^+} \quad \text{(Eq. 8)}$$

$$k_{-3}^{obs} = \frac{k_{-7} \cdot K_{eq}^5}{[H]^+} \quad \text{(Eq. 9)}$$

$$k_4^{obs} = \frac{k_{10} \cdot K_{eq}^6}{[H]^+} \quad \text{(Eq. 10)}$$

$$k_{-4}^{obs} = \frac{k_{-10} \cdot K_{eq}^7}{[H]^+} \quad \text{(Eq. 11)}$$

We need to calculate the anionic mechanism to determine the values of k_2, k_{-2}, k_4, k_{-4}, k_7, k_{-7}, k_{10} and k_{-10} and the pK_a's of the corresponding hydroxyl groups to obtain the equilibrium constant at a given pH (Eq. 8).

$$pK_a^n = -\log K_{eq}^n \qquad (n = 1 - 7) \quad \text{(Eq. 12)}$$

Formal rate constants k_2, k_{-2}, k_4, k_{-4}, k_7, k_{-7}, k_{10} and k_{-10} are calculated from the corresponding constants of the individual two-step acyl migrations (Figure S2) according to Eqs. 14 and 15.

$$k_j = \frac{k_a^i \cdot k_b^i}{k_{-a}^i + k_b^i} \text{ (i=1, j=2; i=2, j=4; i=3, j=7; i=4, j=10)} \quad \text{(Eq. 13)}$$

$$k_{-j} = \frac{k_{-a}^i \cdot k_{-b}^i}{k_{-a}^i + k_b^i} \text{ (i=1, j=2; i=2, j=4; i=3, j=7; i=4, j=10)} \quad \text{(Eq. 14)}$$

In Equations 13 and 14, $k_a^i, k_{-a}^i, \ k_b^i, and \ k_{-b}^i$ (i = 1-4) can be obtained from the corresponding calculated energy barriers by using the Eyring's equation.

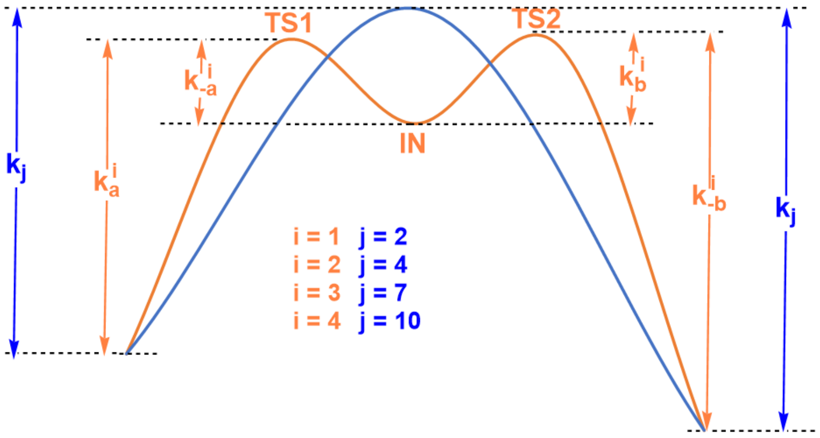

Figure S2. Formal rate constants (in blue) and real barriers (orange) of the acyl group migrations illustrated in Schemes S3 and S4.

Conformational studies

Molecular Dynamics

The conformations of the trisaccharides and the corresponding anions were studied by molecular dynamics. MD simulations were carried out with AMBER20 suite of programs.[20] Parameters for trisaccharides were generated with the antechamber module using the general Amber force field (GAFF2),[21] with partial charges calculated using AM1-BCC method and GLYCAM06 force field. The trisaccharide was neutralized, if necessary, and immersed in a water box with a 12 Å buffer of TIP3P[22] water molecules. A two-stage geometry optimization approach was carried out: (i) minimization of only the positions of solvent molecules executed by 500 cycles of steepest descent minimization followed by 500 cycles of conjugate gradient minimization, and (ii) unrestrained minimization of all the atoms in the simulation cell executed by 2500 cycles of steepest descent minimization followed by 2500 cycles of conjugate gradient minimization. After system optimization, running of MD simulations was started on the systems by gradually heating each system in the NVT ensemble from 0 to 300 K for 100 ps using a Langevin thermostat with a coupling coefficient of 1.0/ps. Harmonic restraints of 10 kcal·mol^{-1} were applied to the solute, and the Langevin temperature coupling scheme[23] was used to control and equalize the temperature. The time step was kept at 2 fs during the heating stages, allowing potential inhomogeneities to self-adjust. Water molecules are treated with the SHAKE algorithm such that the angle between the hydrogen atoms is kept fixed. Long-range electrostatic effects are modelled using the particle-mesh-Ewald method.[24] Then 5 ns of density equilibration with a force constant of 2.0 kcal/mol·Å was performed by releasing all the restraints. Finally, production trajectories were then run for 100 ns under the same simulation conditions with an integration time step of 2 fs, recording geometry every 0.05 ps and with snapshots written each 2 ps, producing 50,000 frames per simulation. We checked a writing step of 0.05 ps with a tri-gluco derivative demonstrating that recording geometries each 2 ps is enough. All MD simulations were replicated three times to ensure feasibility. The cluster analysis of trisaccharide conformations was carried out using cpptraj module with average linkage as the clustering algorithm. We identified the three monosaccharide units as A, B and C (Figure S3). There is a limited conformational freedom due to the presence of hydrogen bonds between the hydroxyl groups at C3 and C6 and the endocyclic oxygen of the adjacent unit even in water as a solvent. No substantial differences were observed between neutral and deprotonated forms. These results were combined with a conformational search using Macromodel as implemented in Schrödinger package.[25] (for combined graphics see next section).

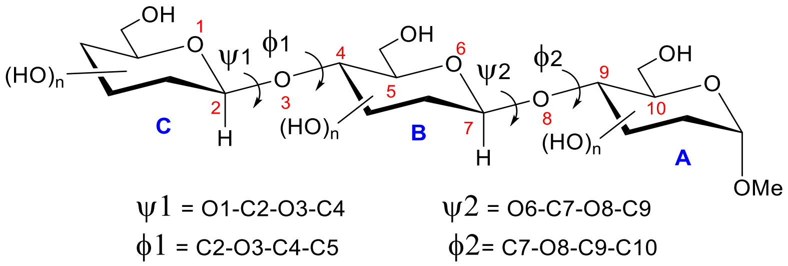

Figure S3. Conformational analysis of trisaccharides.

Conformational search

Locating stationery points of carbohydrates is not an easy task due to the multiple conformations that can be present within a relatively small range of energy (ca. 5 kcal/mol). For this reason, it is necessary to explore the conformational space of those points following a protocol. For that purpose, we used the software Macromodel as implemented in Schrödinger package. The conformational searches were carried out using the Monte Carlo algorithm with OPLS-2005 force field,[26] GB/SA solvation model for water, intermediate torsion sampling with 50 000 Monte Carlo steps and an RMSD cut-off set to 2.0 Å. A Molecular Mechanics energy minimization was performed at each Monte Carlo step, as implemented in Macromodel. Each conformation was energy minimized using Polak-Ribière type conjugate gradient (PRCG) with a maximum of 5000 steps. All conformations within 5 kcal/mol from the global minimum were saved. All ensembles generated by the conformational searches were combined and elimination of redundant conformations was performed by comparison of heavy atom coordinates applying an RMSD cutoff set to 2.0 Å to give the final set of conformations. As an example, the different conformations for D-gluco derivatives are shown in the following figures

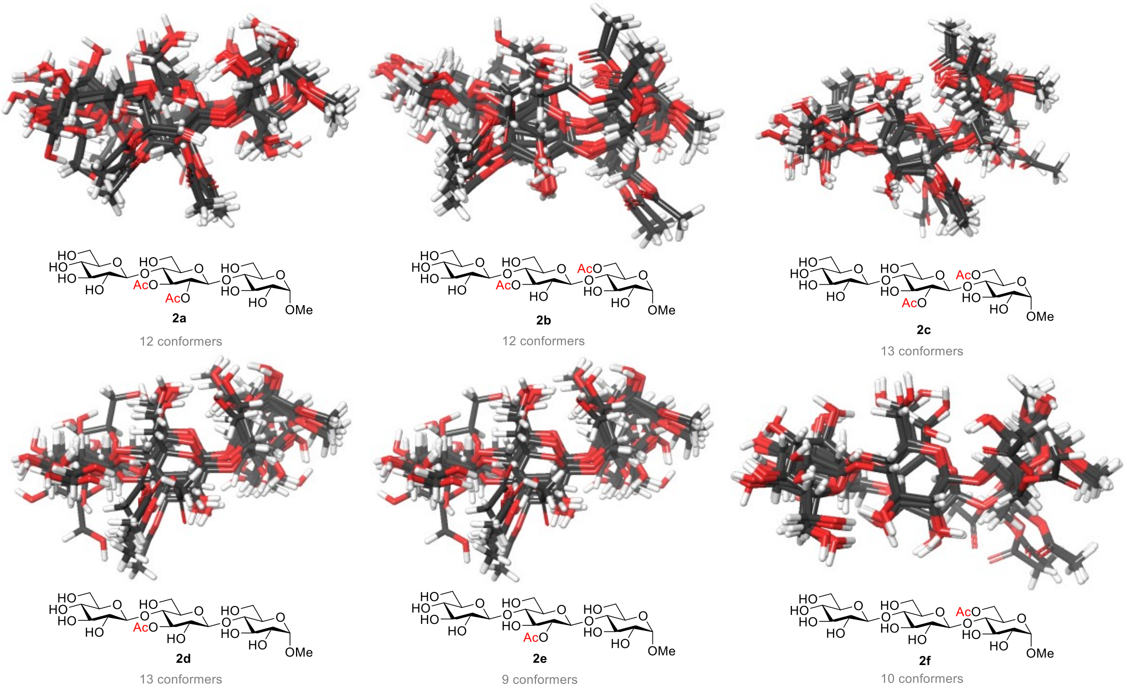

Figure S4. Conformers found for neutral Me α-D-glucotrisaccharides.

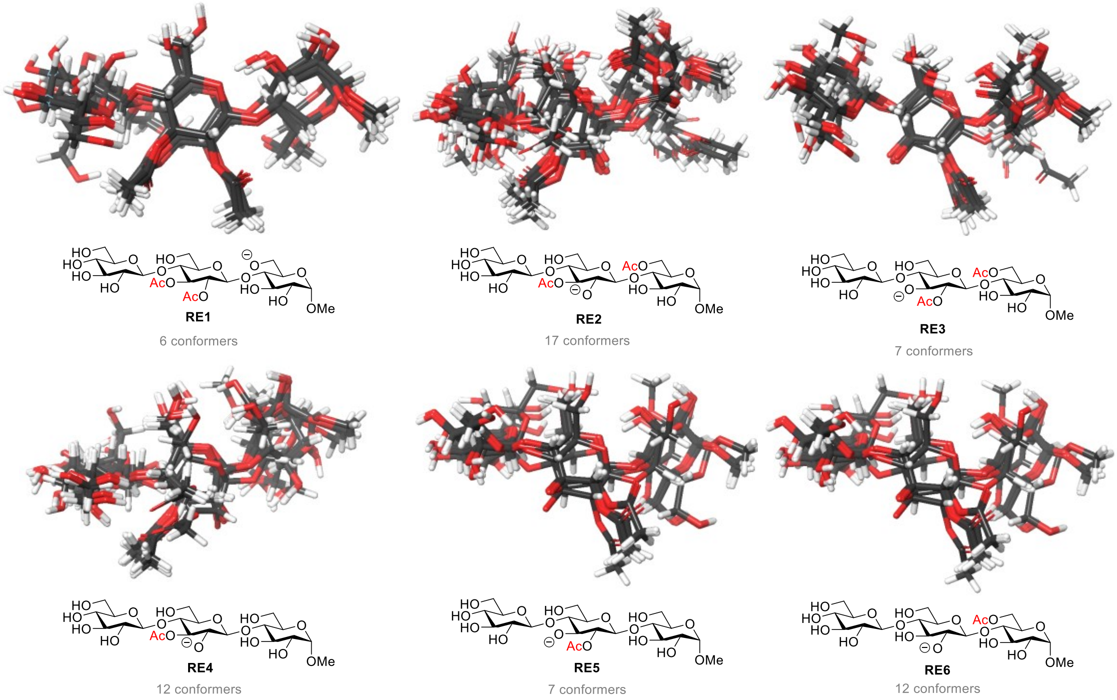

Figure S5. Conformers found for deprotonated Me α-D-glucotrisaccharides.

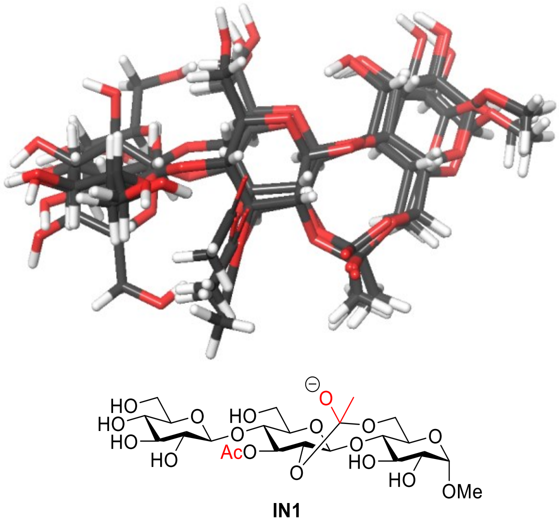

IN1

5 conformers

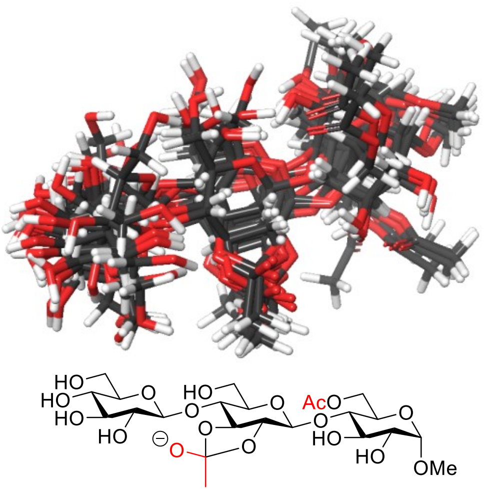

IN2

13 conformers

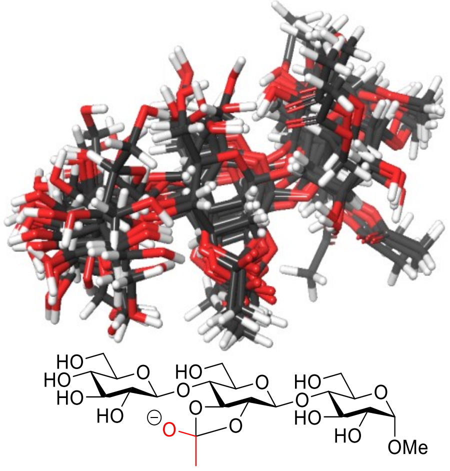

IN3

11 conformers

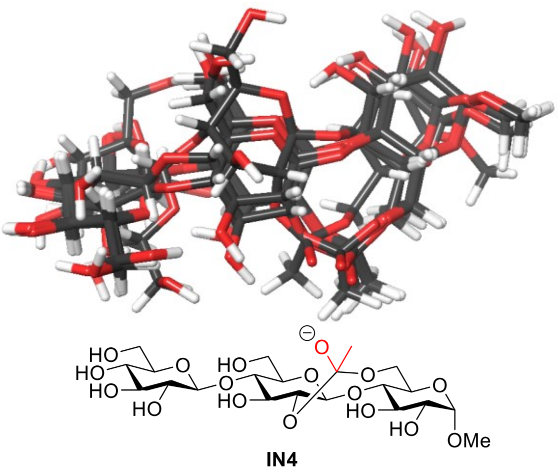

IN4

8 conformers

Figure S6. Conformers found for intermediate orthoesters derived from Me α-D-glucotrisaccharides.

This set was graphically combined with MD simulations and the results are illustrated in the following Figures:

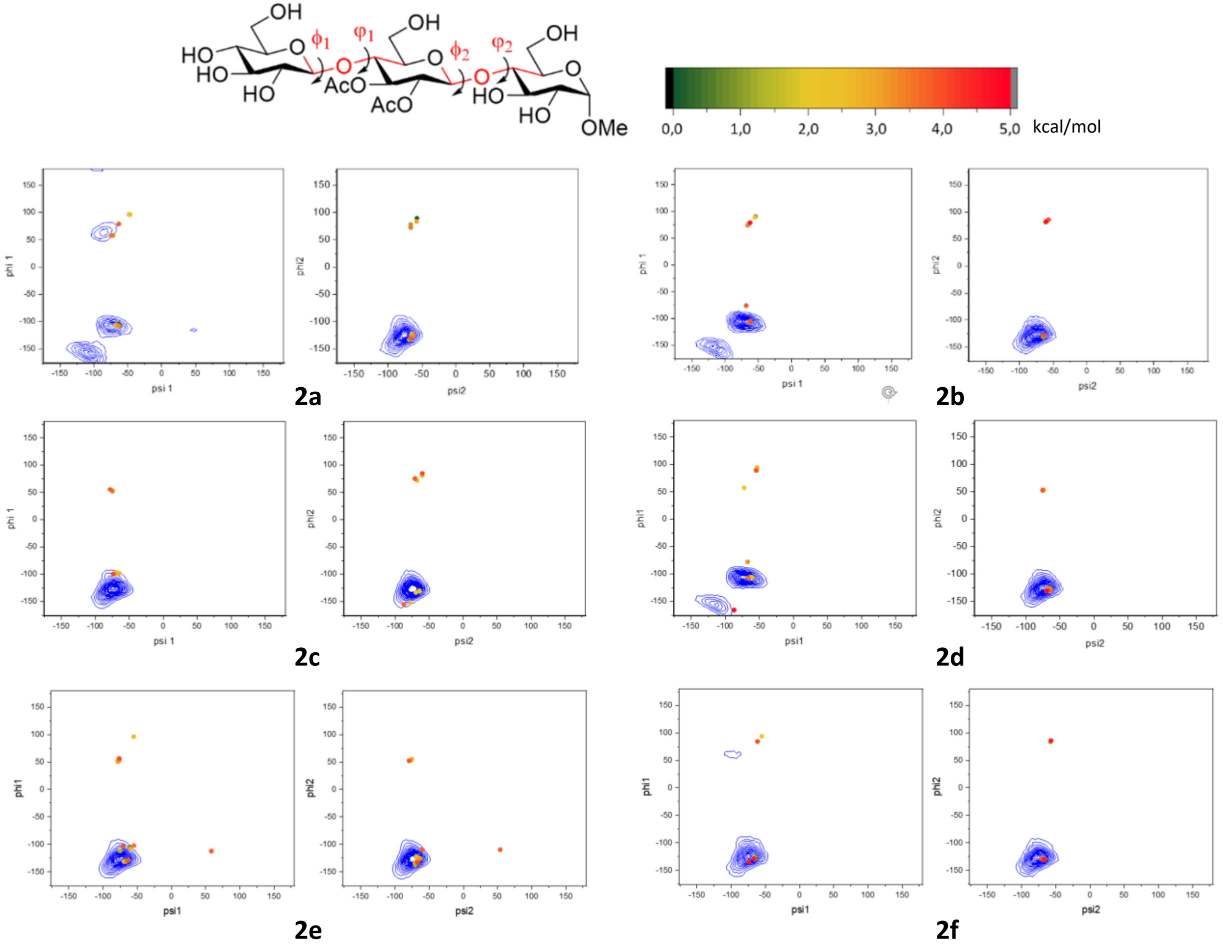

Figure S7. Conformational analyses of neutral Me α-D-glucotrisaccharides using MD simulations. The population analysis was carried out with 50.000 snapshots. Colored dots represent conformers found with Macromodel. The color corresponds to the energy of the points (green: lower and red: higher).

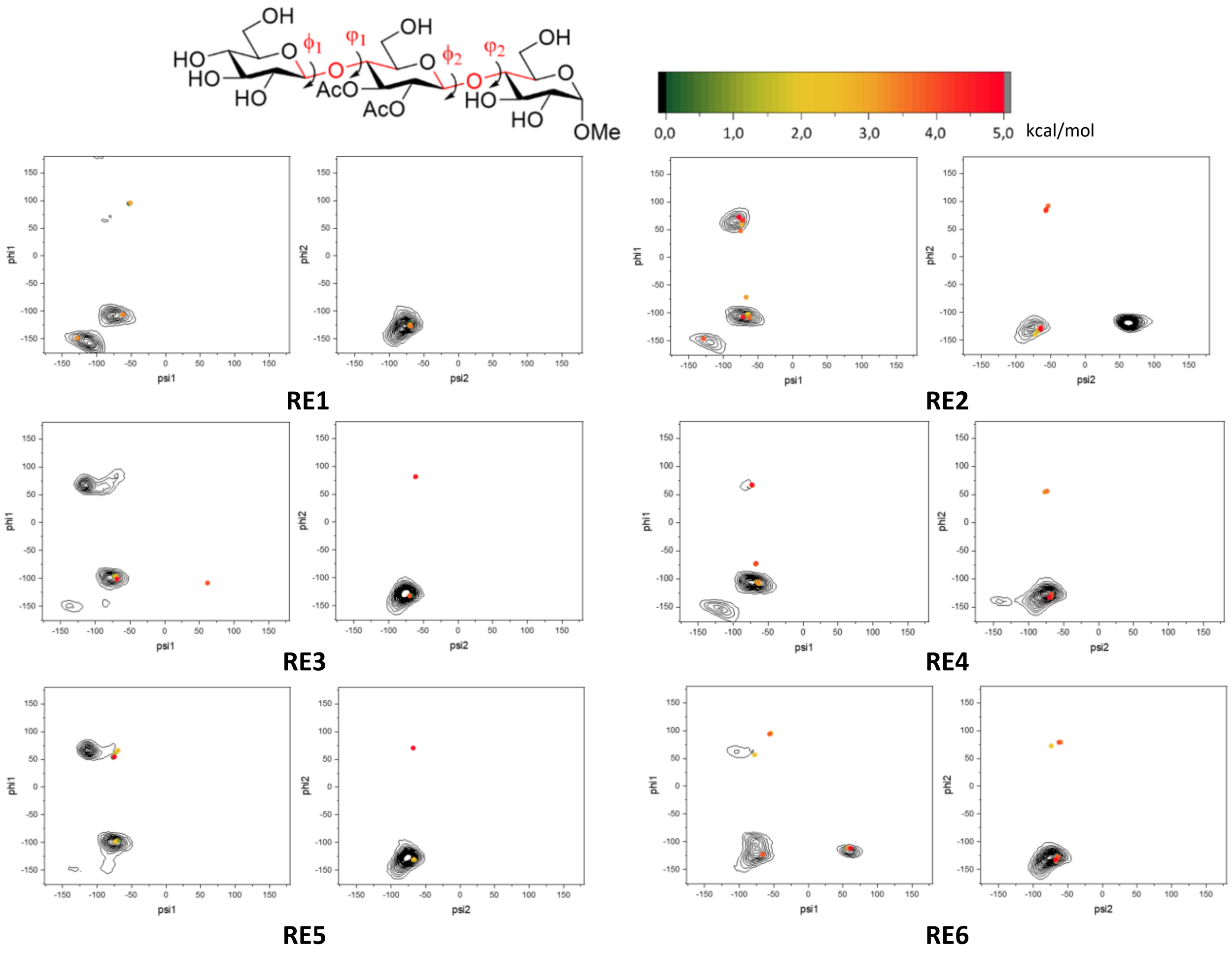

Figure S8. Conformational analyses of deprotonated Me α-D-glucotrisaccharides using MD simulations. The population analysis was carried out with 50.000 snapshots. Colored dots represent conformers found with Macromodel. The color corresponds to the energy of the points (green: lower and red: higher).

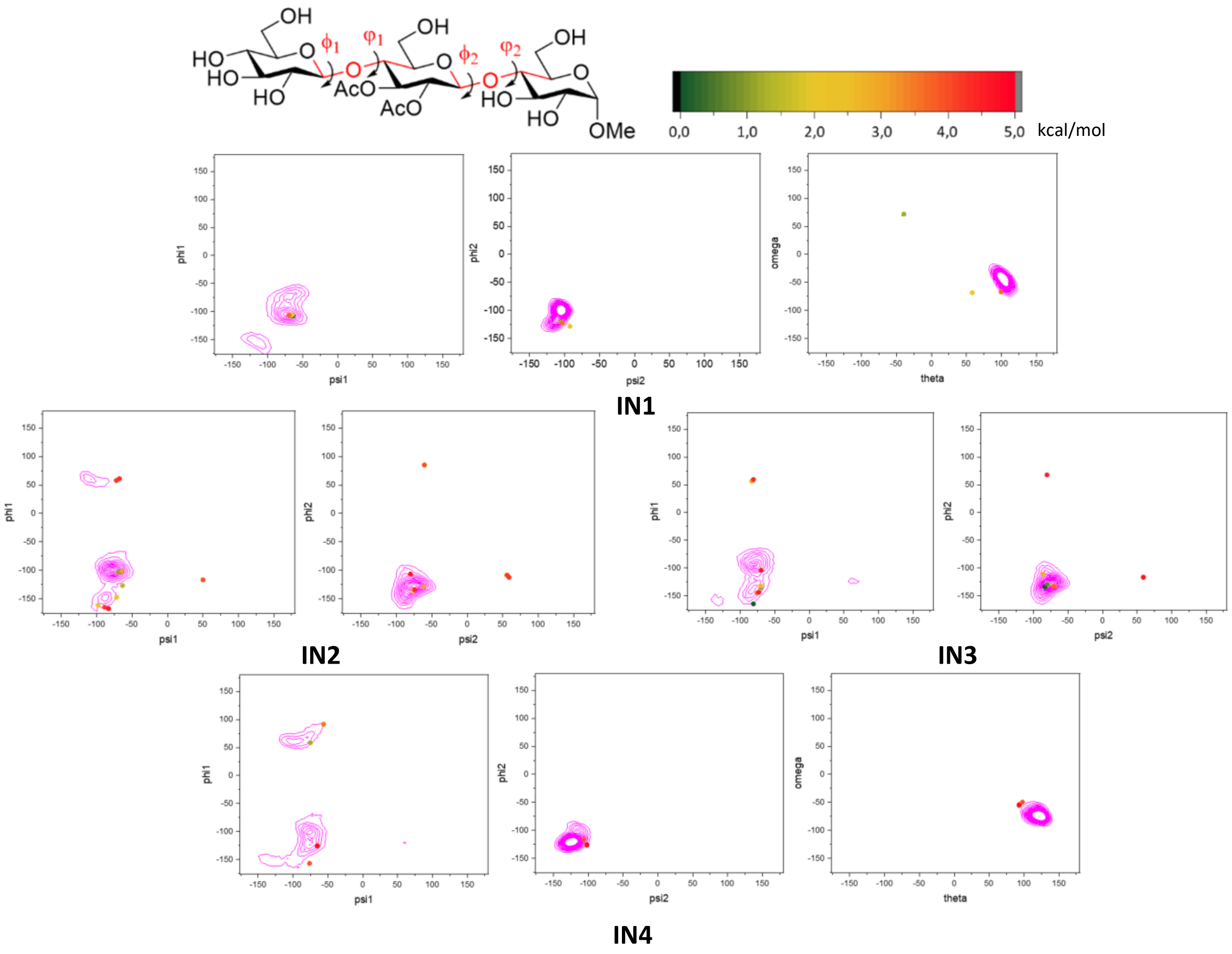

Figure S9. Conformational analyses of intermediate orthoesters derived from Me α-D-glucotrisaccharides using MD simulations. The population analysis was carried out with 50.000 snapshots. Colored dots represent conformers found with Macromodel. The color corresponds to the energy of the points (green: lower and red: higher).

Determination of pKa values

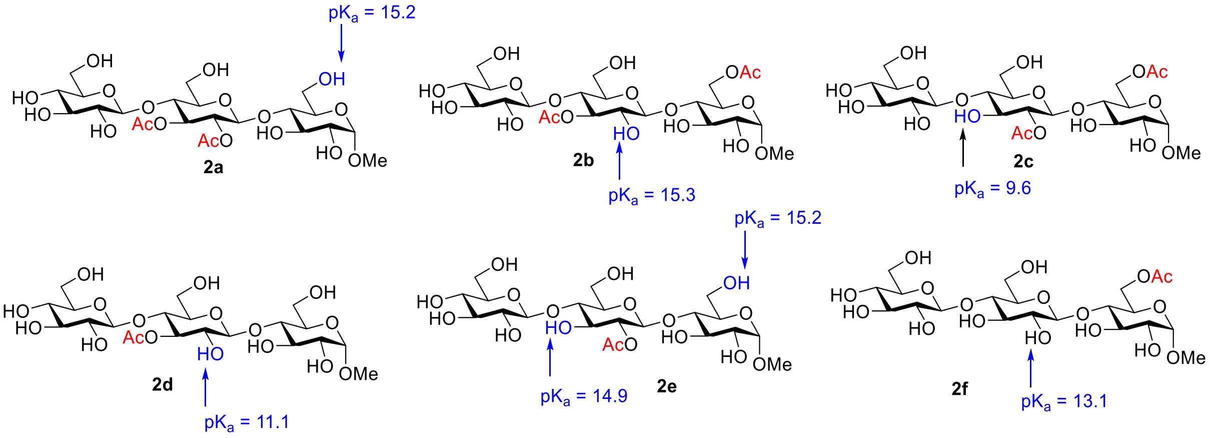

Figure S10. pKa values of the hydroxyl groups involved in acyl transfers of Me α-D-glucotrisaccharides.

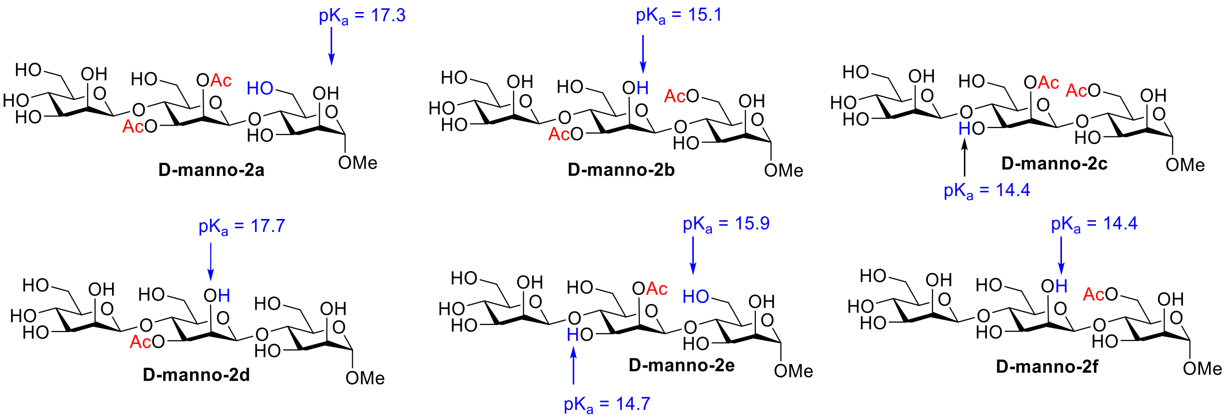

Figure S11. pKa values of the hydroxyl groups involved in acyl transfers of Me α-D-mannotrisaccharides.

Energies

Table S6. Calculated (wb97xd/6-311++G(d,p)/SMD=water//wb97xd/6-31+G(d,p)/SMD=water) absolute (hartree) and relative (kcal/mol) energies for neutral Me α-D-glucotrisaccharides.[a]

	E_0	ΔE_0[a]	G	ΔG[a]
**α-D-Glc-2a**	-2482.040283	0.0	-2482.125383	0.0
**α-D-Glc-2b**	-2482.053389	-8.2	-2482.1357	-6.5
**α-D-Glc-2c**	-2482.052238	-7.5	-2482.135468	-6.3

[a] Referred to the first entry.

Table S7. Calculated (wb97xd/6-311++G(d,p)/SMD=water//wb97xd/6-31+G(d,p)/SMD=water) absolute (hartree) and relative (kcal/mol) energies for neutral Me α-D-glucotrisaccharides.[a]

	E_0	ΔE_0[a]	G	ΔG[a]
**α-D-Glc-2d**	-2329.431809	0.0	-2329.510148	0.0
**α-D-Glc-2e**[b]	-2329.434222	-1.5	-2329.514769	-2.9
**α-D-Glc-2e**[b]	-2329.434624	-1.8	-2329.515454	-3.3
**α-D-Glc-2f**	-2329.439294	-4.7	-2329.518141	-5.0

[a] Referred to the first entry. [b] explicit molecules of water placed in the oxygen atom to be deprotonated

Table S8. Calculated (wb97xd/6-311++G(d,p)/SMD=water//wb97xd/6-31+G(d,p)/SMD=water) absolute (hartree) and relative (kcal/mol) energies for acyl migration in deprotonated diacetylated Me α-D-glucotrisaccharides.[a]

	E_0	ΔE_0[a]	G	ΔG[a]	im. freq
**α-D-Glc-RE1**	-2481.575970	0.0	-2481.661481	0.0	
**α-D-Glc-TS1a**	-2481.563877	7.6	-2481.642843	11.7	-263.3
**α-D-Glc-IN1**	-2481.580462	-2.8	-2481.661558	0.0	
**α-D-Glc-TS1b**	-2481.558342	11.1	-2481.634602	16.9	-270.3
**α-D-Glc-RE2**	-2481.589121	-8.3	-2481.671720	-6.4	
**α-D-Glc-TS2a**	-2481.571640	2.7	-2481.651469	6.3	-203.2
**α-D-Glc-IN2**	-2481.579631	-2.3	-2481.661175	0.2	
**α-D-Glc-TS2b**	-2481.567980	5.0	-2481.649631	7.4	-233.4
**α-D-Glc-RE3**	-2481.600225	-15.2	-2481.683746	-14.0	

[a] Referred to the first entry.

Table S9. Calculated (wb97xd/6-311++G(d,p)/SMD=water//wb97xd/6-31+G(d,p)/SMD=water) absolute (hartree) and relative (kcal/mol) energies for acyl migration in deprotonated monoacetylated Me α-D-glucotrisaccharides.[a]

	E_0	ΔE_0[a]	G	ΔG[a]	im. freq
**α-D-Glc-RE4**	-2328.976706	0.0	-2329.055300	0.0	
**α-D-Glc-TS3a**	-2328.950387	16.5	-2329.029950	15.9	-231.1
**α-D-Glc-IN3**	-2328.961701	9.4	-2329.037532	11.1	
**α-D-Glc-TS3b**	-2328.949595	17.0	-2329.025385	18.8	-229.4
**α-D-Glc-RE5a**	-2328.974810	1.2	-2329.051616	2.3	
**α-D-Glc-RE5b**	-2328.963253	8.4	-2329.043248	7.6	
**α-D-Glc-TS4a**	-2328.937366	24.7	-2329.014184	25.8	-294.3
**α-D-Glc-IN4**	-2328.953428	14.6	-2329.034139	13.3	
**α-D-Glc-TS4b**	-2328.938357	24.1	-2329.014184	25.8	-294.3
**α-D-Glc-RE6**	-2328.978686	-1.2	-2329.058994	-2.3	

[a] Referred to the first entry.

Table S10. Calculated (wb97xd/6-311++G(d,p)/SMD=water//wb97xd/6-31+G(d,p)/SMD=water) absolute (hartree) and relative (kcal/mol) energies for neutral Me α-D-mannotrisaccharides.[a]

	E_0	ΔE_0[a]	G	ΔG[a]
**α-D-Man-2a**	-2482.043902	0.0	-2482.128121	0.0
**α-D-Man-2b**	-2482.05338	-5.9	-2482.138014	-6.2
**α-D-Man-2c**	-2482.05121	-4.6	-2482.136985	-5.6

[a] Referred to the first entry.

Table S11. Calculated (wb97xd/6-311++G(d,p)/SMD=water//wb97xd/6-31+G(d,p)/SMD=water) absolute (hartree) and relative (kcal/mol) energies for neutral Me α-D-mannotrisaccharides.[a]

	E_0	ΔE_0[a]	G	ΔG[a]
**α-D-Man-2d**	-2329.430095	0.0	-2329.507418	0.0
**α-D-Man-2e[b]**	-2329.431608	-0.9	-2329.512202	-3.0
**α-D-Man-2e[b]**	-2329.430208	-0.1	-2329.509126	-1.1
**α-D-Man-2f**	-2329.437834	-4.9	-2329.518069	-6.7

[a] Referred to the first entry. b: explicit molecules of water placed in the oxygen atom to be deprotonated

Table S12. Calculated (wb97xd/6-311++G(d,p)/SMD=water//wb97xd/6-31+G(d,p)/SMD=water) absolute (hartree) and relative (kcal/mol) energies for acyl migration in deprotonated diacetylated Me α-D-mannotrisaccharides.[a]

	E_0	ΔE_0[a]	G	ΔG[a]	im. freq
**α-D-Man-RE1**	-2481.576407	0.0	-2481.659797	0.0	
**α-D-Man-TS1a**	-2481.558167	11.4	-2481.637442	14.0	-267.3
**α-D-Man-IN1**	-2481.569298	4.5	-2481.651689	5.1	
**α-D-Man-TS1b**	-2481.558204	11.4	-2481.635929	15.0	-252.6
**α-D-Man-RE2**	-2481.591813	-9.7	-2481.674514	-9.2	
**α-D-Man-TS2a**	-2481.575779	0.4	-2481.657884	1.2	-225.0
**α-D-Man-IN2**	-2481.585183	-5.5	-2481.666007	-3.9	
**α-D-Man-TS2b**	-2481.569649	4.2	-2481.651793	5.0	-272.9
**α-D-Man-RE3**	-2481.590590	-8.9	-2481.674856	-9.4	

[a] Referred to the first entry.

Table S13. Calculated (wb97xd/6-311++G(d,p)/SMD=water//wb97xd/6-31+G(d,p)/SMD=water) absolute (hartree) and relative (kcal/mol) energies for acyl migration in deprotonated monoacetylated Me α-D-mannotrisaccharides.[a]

	E_0	ΔE_0[a]	G	ΔG[a]	im. freq
**α-D-Man-RE4**	-2328.962227	0.0	-2329.038046	0.0	
**α-D-Man-TS3a**	-2328.950553	7.3	-2329.026156	7.5	-225.5
**α-D-Man-IN3**	-2328.965928	-2.3	-2329.044913	-4.3	
**α-D-Man-TS3b**	-2328.949851	7.8	-2329.026137	7.5	-267.5
**α-D-Man-RE5a**	-2328.970715	-5.3	-2329.049477	-7.2	
**α-D-Man-RE5b**	-2328.964319	-1.3	-2329.043816	-3.6	
**α-D-Man-TS4a**	-2328.941576	13.0	-2329.017038	13.2	-290.0
**α-D-Man-IN4**	-2328.962725	-0.3	-2329.038851	-0.5	
**α-D-Man-TS4b**	-2328.940041	13.9	-2329.014533	14.8	-241.6
**α-D-Man-RE6**	-2328.976943	-9.2	-2329.055959	-11.2	

[a] Referred to the first entry.

Predicted Values

Table S14. Calculated (wb97xd/6-311++G(d,p)/SMD=water//wb97xd/6-31+G(d,p)/ SMD=water) formal energy barriers[a] (kcal/mol) and rate constants[b] (s^{-1}) for the acetyl group migration in Me α-D-glucotrisaccharides at pH=8.

	experimental rate constants	ΔG	predicted rate constants	ΔG	ΔG error
K_{1}^{obs}	9.25E-06	24.3	1.56E-07	26.7	**2.4**
K_{-1}^{obs} [c]	<1.00E-14	>36.0	2.80E-12	33.2	[d]
K_{2}^{obs}	1.37E-04	22.7	2.01E-05	23.9	**1.2**
K_{-2}^{obs}	1.34E-04	22.7	2.52E-05	23.7	**1.0**
K_{3}^{obs}	1.37E-04	22.7	8.97E-05	23.0	**0.3**
K_{-3}^{obs}	1.34E-04	22.7	6.79E-07	25.9	**3.2**
K_{4}^{obs}	1.35E-06	25.5	8.25E-09	28.5	**3.0**
K_{-4}^{obs}[c]	< 1.00E-14	>36.0	6.73E-14	35.4	[d]

[a] Obtained from the individual barriers of the stepwise mechanism. [b] Obtained by applying previously reported kinetic equations (see Supporting Information). [c] Since this constant has not been observed experimentally it is considered very low and an arbitrary valor has been employed. [d] This error cannot be considered because the experimental value has not ben measured, just confirming that this process is very slow.

Table S15. Calculated (wb97xd/6-311++G(d,p)/SMD=water//wb97xd/6-31+G(d,p)/ SMD=water) formal energy barriers[a] (kcal/mol) and rate constants[b] (s^{-1}) for the acetyl group migration in Me α-D-mannotrisaccharides at pH=8.

	experimental rate constants	ΔG	predicted rate constants	ΔG	ΔG error
K_{1}^{obs}	5.72E-07	26.0	2.93E-08	27.7	**1.7**
K_{-1}^{obs} [c]	> 1.00E-14	> 36.0	8.19E-13	33.9	[d]
K_{2}^{obs}	5.22E-04	21.9	1.95E-05	23.9	**2.0**
K_{-2}^{obs}	2.81E-04	22.3	5.78E-05	23.2	**0.9**
K_{3}^{obs}	5.22E-04	21.9	1.86E-03	21.2	**0.3**
K_{-3}^{obs}	2.81E-04	22.3	1.16E-05	24.2	**0.9**
K_{4}^{obs}	5.72E-07	26.0	2.57E-09	29.2	**3.2**
K_{-4}^{obs}[c]	> 1.00E-14	> 36.0	1.97E-13	34.8	[d]

[a] Obtained from the individual barriers of the stepwise mechanism. [b] Obtained by applying previously reported kinetic equations (see Supporting Information). [c] Since this constant has not been observed experimentally it is considered very low and an arbitrary valor has been employed. [d] This error cannot be considered because the experimental value has not been measured, just confirming that this process is very slow.

Transition Structures

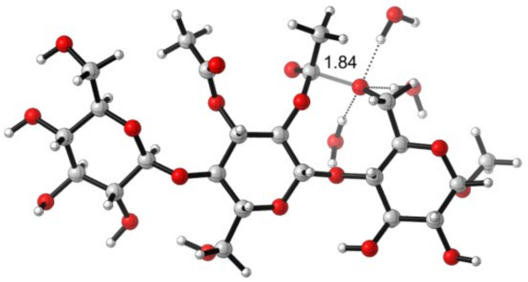

D-Glc-TS1a

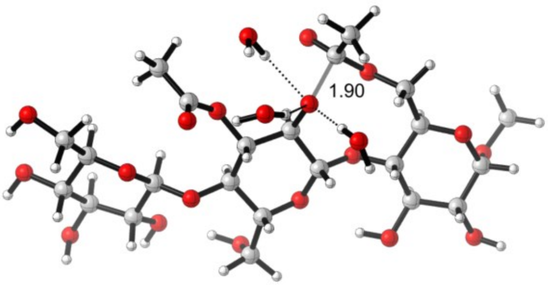

D-Glc-TS1b

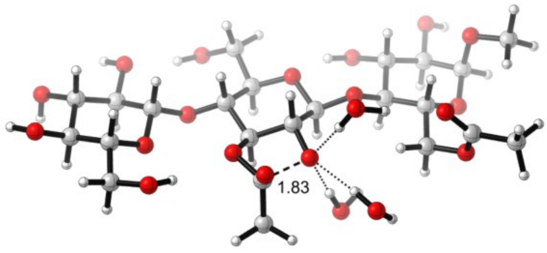

D-Glc-TS2a

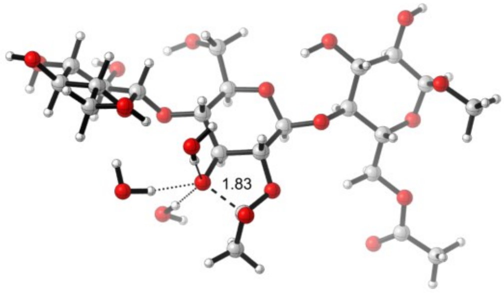

D-Glc-TS2b

Figure S12. Optimized (wb97xd/6-31+G(d,p)/SMD=water) geometries of transition structures for the acyl migration in diacetylated derivatives of Me α-D-glucotrisaccharides.

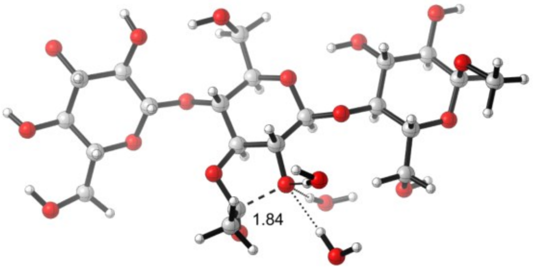

D-Glc-TS3a

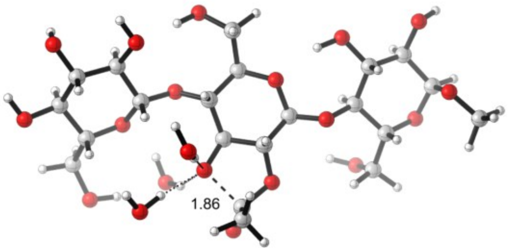

D-Glc-TS3b

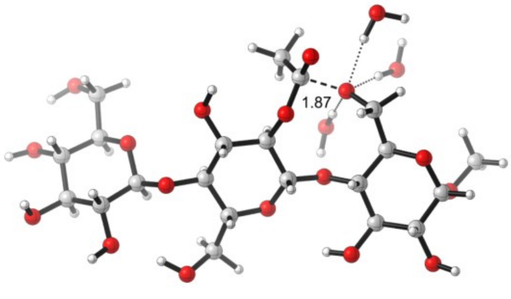

D-Glc-TS4a

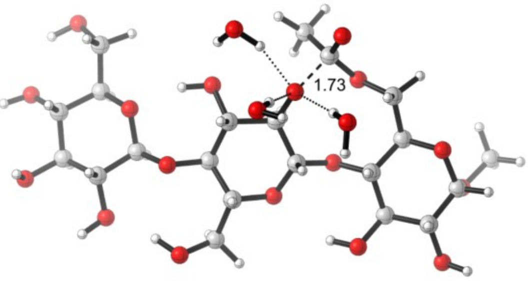

D-Glc-TS4b

Figure S13. Optimized (wb97xd/6-31+G(d,p)/SMD=water) geometries of transition structures for the acyl migration in monoacetylated derivatives of Me α-D-glucotrisaccharides.

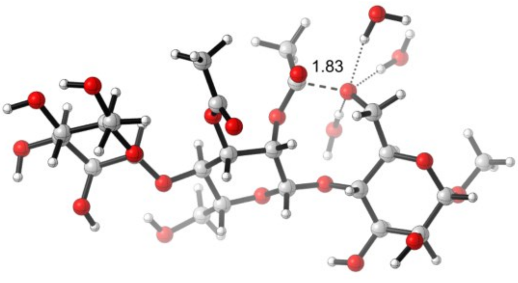

D-Man-TS1a

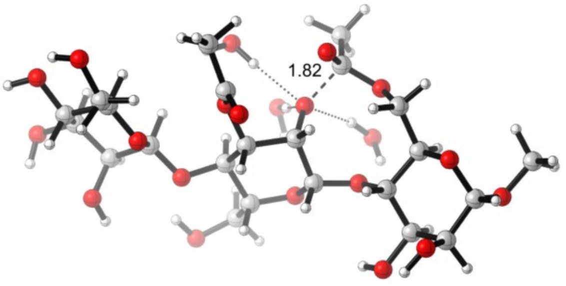

D-Man-TS1b

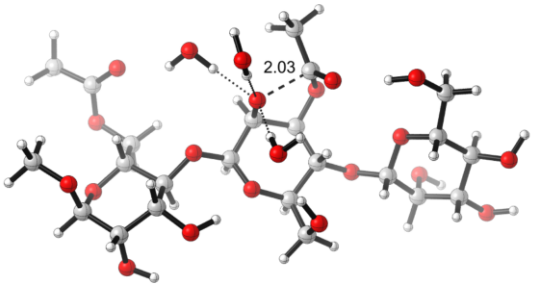

D-Man-TS2a

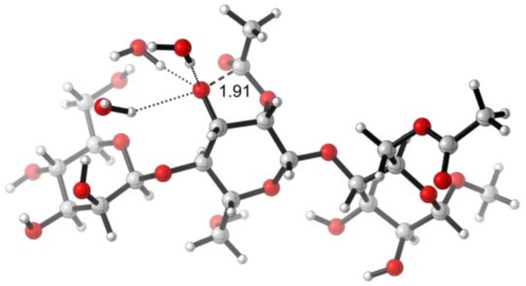

D-Man-TS2b

Figure S14. Optimized (wb97xd/6-31+G(d,p)/SMD=water) geometries of transition structures for the acyl migration in diacetylated derivatives of Me α-D-mannotrisaccharides.

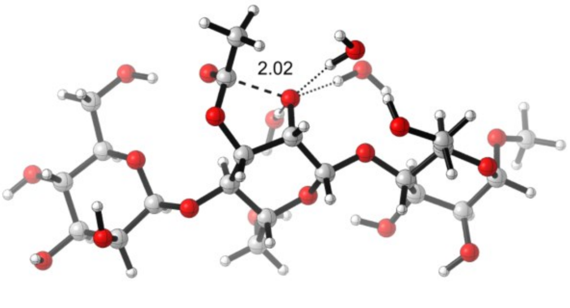

D-Man-TS3a

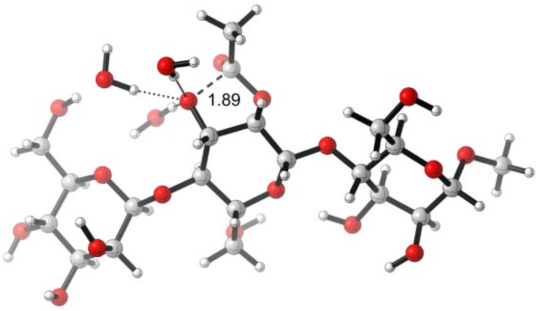

D-Man-TS3b

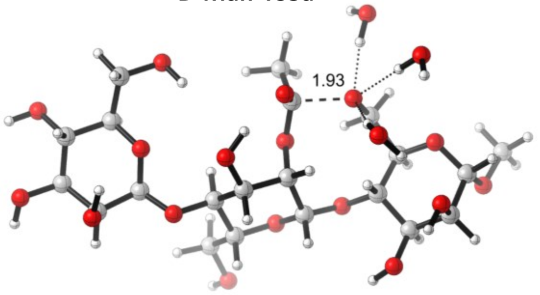

D-Man-TS4a

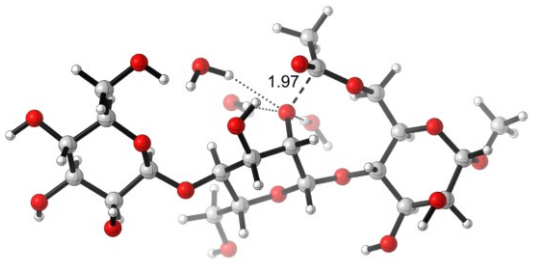

D-Man-TS4b

Figure S15. Optimized (wb97xd/6-31+G(d,p)/SMD=water) geometries of transition structures for the acyl migration in monoacetylated derivatives of Me α-D-mannotrisaccharides.

Cartesian Coordinates

D-Glc-2a

0 1

C	6.7650535916	0.7196121463	-0.6998428351
C	6.4694115116	-0.5766095066	0.0522075971
C	5.1373988413	-1.1535649288	-0.4214954419
C	4.0476744216	-0.0955755737	-0.2881317839
O	4.3994737972	1.0428975941	-1.0509603337
C	5.5803073649	1.6858655782	-0.5841005364
H	6.9243202016	0.4881441342	-1.7628563602
O	7.9091745164	1.3796450611	-0.1733895146
H	6.4011792695	-0.3576191113	1.1236040340
O	7.5216305711	-1.5206816250	-0.0764961680
H	5.2124709173	-1.4410638103	-1.4793670471
O	4.7371255392	-2.2686790184	0.3606104739
H	3.9156093185	0.1873409134	0.7661442143
H	5.4527179362	1.9783356496	0.4691137625
C	5.7595875378	2.9397331949	-1.4355374673
H	8.6920685711	0.8611728917	-0.3947075542
H	7.6372030579	-1.7347435992	-1.0121328902
H	5.4363286015	-2.9339282789	0.3207570654
H	4.8127234368	3.4818378414	-1.4761705888
H	6.0371144367	2.6518283150	-2.4581445473
O	6.7229729991	3.8240726765	-0.8807224573
H	7.5220139505	3.3033164778	-0.7093739530
O	2.8635013359	-0.5995052156	-0.8226313138
O	-2.4602791546	-0.2989060556	0.3212909918
O	-1.0077521425	2.1082044357	-0.1971870095
C	2.0955688269	2.9953849694	-0.2988466818
C	2.8558150595	3.9324345014	0.5831314761
O	1.7919133967	3.2053970476	-1.4593299466
H	3.6887961071	3.4087402878	1.0584806229
H	2.1900020210	4.2928769117	1.3731951359
H	3.2230132963	4.7766950087	0.0013791748
C	1.6801971245	-0.4816868209	-0.0470298022
C	0.9996614543	0.8583598244	-0.3029394394
C	-0.4154308301	0.8934457783	0.2555747652
C	-1.2047769682	-0.3060379330	-0.2593304058
O	-0.5033233964	-1.4712959622	0.1740937150
C	0.7692763347	-1.6466891937	-0.4524694634
H	1.9158021228	-0.5732464465	1.0205217969
H	0.9797133879	1.0581267107	-1.3771906909
O	1.7604606209	1.8738824672	0.3633419791

H	-0.4028415277	0.8631300861	1.3474581758
H	0.6464162638	-1.6564853163	-1.5443257568
C	1.2728288228	-3.0111583879	0.0267624868
H	0.4701201789	-3.7360667371	-0.1322895725
H	1.4806645121	-2.9606235513	1.1030347999
O	2.4007187023	-3.4815442515	-0.6861390239
H	3.1942053122	-3.0130970912	-0.3653114511
H	-1.2694314356	-0.3120597672	-1.3559266838
O	-4.9007171807	-4.3989101349	0.5966826918
C	-1.5228813872	2.9630319839	0.7004909641
C	-1.9698745421	4.2361313762	0.0585432613
O	-1.5707460080	2.7308949343	1.8956476543
H	-2.1881435576	4.0943458471	-0.9998155037
H	-1.1535304554	4.9601124325	0.1542640897
H	-2.8414517195	4.6271872353	0.5847073021
C	-3.5116553413	-1.0337900905	-0.3261617684
C	-3.5573222462	-2.4656222913	0.2369482225
C	-4.8772061677	-3.1522326333	-0.0751289119
C	-6.0505074423	-2.2617262222	0.3279505074
O	-5.9563127919	-1.0398726305	-0.3716889880
C	-4.8081864644	-0.2498611648	-0.0617212260
H	-3.3188973230	-1.0799511204	-1.4066049986
H	-3.4415183369	-2.4007262448	1.3269228786
O	-2.5259246310	-3.2700673752	-0.3116929895
H	-4.9502869461	-3.3033754366	-1.1592143826
O	-6.0399632635	-2.0869122636	1.7157476869
H	-4.8259224749	0.0399682069	0.9966204633
C	-5.0047329121	0.9806222118	-0.9391935413
H	-5.6196169093	-4.9273206935	0.2310542673
H	-6.0057252068	1.3753462168	-0.7334084866
H	-4.9579158354	0.6831519601	-1.9947797658
O	-4.0255856881	1.9719231910	-0.6611896025
C	-7.1523846471	-1.3449105196	2.2064141831
H	-7.0031898655	-2.6985547534	0.0045885937
H	-8.0937035067	-1.7767917410	1.8457811596
H	-7.0932622306	-0.2940804970	1.9021514417
H	-7.1147443429	-1.4080332445	3.2943333919
H	-1.6790155207	-2.8372870862	-0.1041444521
H	-4.3661658097	2.8266707529	-1.0103563174
O	-5.0921895585	4.3298942185	-1.5194484604
H	-4.3515291463	4.9340514389	-1.6523445032
H	-4.5028946900	2.4721948486	1.1203891729
O	-4.9727712494	2.7156782016	1.9393060259
H	-5.6080978523	2.0019931164	2.0681840799
H	-2.8044101099	1.6984310777	-2.1015305699
O	-2.4146963659	1.5279682335	-2.9791475683

H	-2.5229472197	0.5788804650	-3.1064895683
H	-5.5363090582	4.6571460833	-0.7275895005

D-Glc-2b

0 1

C	-6.6551300000	-0.7604260000	-0.0703930000
C	-6.0091630000	-1.9104380000	-0.8411470000
C	-4.6429760000	-2.2493650000	-0.2424590000
C	-3.7926200000	-0.9872550000	-0.1255630000
O	-4.4808260000	-0.0064030000	0.6231800000
C	-5.6880920000	0.4242380000	0.0056990000
H	-6.8822330000	-1.0926900000	0.9528040000
O	-7.8435450000	-0.3084930000	-0.7063560000
H	-5.8701630000	-1.6001670000	-1.8826630000
O	-6.8501480000	-3.0535840000	-0.8932050000
H	-4.7743100000	-2.6710930000	0.7633260000
O	-3.9315360000	-3.1590120000	-1.0696830000
H	-3.5587300000	-0.5962900000	-1.1259710000
H	-5.4772150000	0.7871200000	-1.0118610000
C	-6.2203380000	1.5791300000	0.8484980000
H	-8.5132770000	-0.9987950000	-0.6313300000
H	-7.0024420000	-3.3660780000	0.0086110000
H	-4.4026300000	-4.0011990000	-1.0781090000
H	-5.4062470000	2.2818280000	1.0390420000
H	-6.5810400000	1.1970730000	1.8125790000
O	-7.2434600000	2.2987150000	0.1754060000
H	-7.9073380000	1.6523410000	-0.1065290000
O	-2.6246340000	-1.2736060000	0.5840360000
O	2.6453650000	-0.2732520000	-0.4302960000
C	-2.4340280000	2.3286280000	0.1476520000
C	-3.3064020000	3.1259430000	-0.7666550000
O	-2.2713190000	2.5670530000	1.3344220000
H	-3.9241890000	2.4644900000	-1.3776540000
H	-2.6674650000	3.7060990000	-1.4397160000
H	-3.9310820000	3.8040270000	-0.1868830000
C	-1.3920290000	-0.9870970000	-0.0572640000
C	-0.9361420000	0.4423990000	0.1953810000
C	0.4761880000	0.6860230000	-0.3360930000
C	1.4112700000	-0.3993180000	0.1908240000
O	0.8912550000	-1.6830800000	-0.1390340000
C	-0.3539540000	-1.9572660000	0.5072520000
H	-1.4810170000	-1.1592590000	-1.1357900000
H	-0.9687100000	0.6539090000	1.2662040000
O	-1.8281830000	1.3282630000	-0.5013710000

H	0.4696680000	0.6320930000	-1.4319110000
H	-0.2401040000	-1.7947850000	1.5891120000
C	-0.7157160000	-3.4195500000	0.2912470000
H	-1.4893000000	-3.6935170000	1.0156880000
H	0.1728720000	-4.0222740000	0.4994410000
O	-1.1417110000	-3.7173900000	-1.0276150000
H	-2.0878980000	-3.4910640000	-1.0901590000
H	1.5115680000	-0.3210500000	1.2836410000
O	5.8285590000	-3.7123770000	0.7331820000
C	3.4215890000	3.3660950000	-1.2900200000
C	3.4734560000	4.2725410000	-0.1019270000
H	2.9204970000	3.8278220000	0.7307290000
H	3.0206130000	5.2266500000	-0.3668070000
H	4.5057610000	4.4257080000	0.2237950000
C	3.8019350000	-0.6071190000	0.3346190000
C	4.1316380000	-2.1011330000	0.2529030000
C	5.4790420000	-2.3529100000	0.9185590000
C	6.5565200000	-1.4107560000	0.3667420000
O	6.1397180000	-0.0639860000	0.5050350000
C	4.9506290000	0.2390940000	-0.2197870000
H	3.6350530000	-0.3330700000	1.3847790000
H	4.1823490000	-2.3929450000	-0.8047490000
O	3.1679590000	-2.8925830000	0.9296300000
H	5.3795850000	-2.1298180000	1.9882720000
O	6.8142620000	-1.7493470000	-0.9645020000
H	5.0950090000	0.0152260000	-1.2837930000
C	4.7247030000	1.7374250000	-0.0650570000
H	6.5455960000	-3.9307910000	1.3400370000
H	5.6897660000	2.2466640000	-0.0086770000
H	4.1340040000	1.9569780000	0.8272940000
O	4.0335760000	2.1833200000	-1.2458140000
C	7.9049360000	-1.0346420000	-1.5379740000
H	7.4729990000	-1.4815340000	0.9643440000
H	8.7993180000	-1.1267590000	-0.9103360000
H	7.6596170000	0.0244210000	-1.6693040000
H	8.0954650000	-1.4841340000	-2.5130000000
H	2.3014340000	-2.7349810000	0.5146560000
O	2.8372180000	3.6486680000	-2.3310520000
O	0.9684340000	1.9338680000	0.0971010000
H	-0.1014300000	4.7266330000	-1.0224240000
H	0.2383630000	2.0164340000	3.3799080000
H	1.6559630000	2.0811130000	1.7457520000
O	0.1305380000	3.9441660000	-1.5360200000
O	-0.6916120000	1.9949210000	3.6783890000
O	1.9436120000	2.0775140000	2.6815510000
H	1.0125340000	4.1233830000	-1.9053380000

H	-1.2091620000	2.1512720000	2.8726800000
H	2.3175680000	2.9524620000	2.8355600000
H	0.6003030000	2.6560900000	-0.4613430000

D-Glc-2c

0 1

O	2.8236880137	-1.9754383148	0.7720049102
C	3.2301548642	-2.9343085175	-0.2015224413
C	2.6548191967	-4.2706138625	0.2271269800
O	1.2349924026	-4.2842073376	0.1631857081
H	0.8985813893	-3.6515781236	0.8090579700
H	3.0058049733	-5.0490444410	-0.4520393702
H	2.9996551565	-4.5080417842	1.2414539355
H	2.8173864741	-2.6579494949	-1.1836497084
C	4.7577894067	-2.9324883798	-0.2719655084
O	5.1616500920	-3.8425485244	-1.2811817683
H	6.1242705093	-3.8095034725	-1.3440554979
H	5.1608640910	-3.2428366265	0.7034454205
C	5.2410083673	-1.5171890437	-0.5875463243
O	6.6589853563	-1.4344285745	-0.6088316386
H	6.9971650976	-1.6724355691	0.2651114233
H	4.9206589788	-1.2634763932	-1.6044550419
C	4.6475216492	-0.4890117882	0.3774378570
O	4.9614519043	0.8267287453	-0.0554854040
H	5.9074651537	0.8573414613	-0.2527753512
H	5.0365102879	-0.6592309404	1.3906626806
C	3.1309496460	-0.6409293186	0.4119076850
H	2.7100786590	-0.4221016483	-0.5808258474
O	2.5989238084	0.1929699446	1.3962337828
C	1.2706286714	0.6971738948	1.1969304882
C	0.2559436953	-0.4162522333	0.8758147403
O	-0.9801993385	0.1818713690	0.5078384060
C	-0.0441840001	-1.3289603132	2.0577592245
O	-0.5101805748	-0.6390622514	3.2044394868
H	-1.4053431234	-0.3021299820	3.0324900109
H	-0.7807436686	-2.0738994402	1.7282222403
H	0.8713527028	-1.8472190461	2.3472581256
H	0.6096984962	-1.0412824645	0.0447862692
H	1.0008977407	1.1337285025	2.1621840917
C	1.2875184133	1.8430521242	0.1688241946
H	1.8704976612	1.5376129194	-0.7098531250
C	-0.1127715497	2.2114176866	-0.3094948414
O	0.0245382099	3.0288495765	-1.4778472187
C	-0.6359258170	4.1936993837	-1.5518615041

C	-0.4719992230	4.8484847127	-2.8849761846
H	0.4280403752	4.5030297713	-3.3944777881
H	-1.3429362255	4.5877234129	-3.4953849710
H	-0.4523176692	5.9313090390	-2.7579441571
O	-1.3116576953	4.6442408100	-0.6406744663
H	-0.6581424443	2.7488815306	0.4680085908
C	-0.8881213626	0.9530039699	-0.6655531684
H	-0.3514878923	0.3833027349	-1.4391673180
O	-2.1632312583	1.3254947076	-1.0824943957
C	-3.0927058676	0.3017257100	-1.4645025317
H	-3.8554827415	0.8380216422	-2.0385754523
C	-2.4873393719	-0.7731054299	-2.3738857773
O	-2.1311260748	-0.2374312351	-3.6414509723
H	-1.2916106431	0.2307182071	-3.5714870265
H	-1.6125326729	-1.2324761551	-1.8996421148
C	-3.5045209062	-1.8831788782	-2.6366301804
O	-2.9196541558	-2.9671597540	-3.3345782592
H	-2.6584676306	-2.6517899100	-4.2081581976
H	-4.3388347509	-1.4669531498	-3.2150812307
C	-4.0924973665	-2.4038638851	-1.3290663585
O	-3.0715586140	-3.0105030712	-0.5865804264
C	-3.5071416064	-3.5625875865	0.6526721300
H	-4.3803941904	-4.2082861054	0.5018622244
H	-2.6776045904	-4.1551911632	1.0393563572
H	-3.7582866894	-2.7753704554	1.3728307099
H	-4.9051929011	-3.1143763296	-1.5175215904
O	-4.6947562554	-1.3263283363	-0.6424273323
C	-3.7603047377	-0.3282957019	-0.2290026834
H	-2.9955028264	-0.7853298864	0.4011035206
C	-4.5349440311	0.6923958153	0.5763185945
H	-3.9068685847	1.5473243214	0.8268599134
H	-5.4190683418	1.0272063141	0.0322657268
O	-5.0450500694	0.0824313254	1.7782429566
C	-4.2143132215	-0.0331097400	2.8191371556
O	-3.0967979856	0.4632164552	2.8262215302
C	-4.8003886238	-0.8413754828	3.9294657007
H	-4.2373173095	-0.6757982111	4.8470888578
H	-5.8542878940	-0.5971716738	4.0738898124
H	-4.7334568932	-1.8986653113	3.6519736536
H	0.7896154440	4.2593590875	1.6317118359
O	0.1464553939	4.9293583823	1.9269269542
H	-0.4903163837	4.9530015279	1.1975191167
H	2.2131533000	4.3690417912	-0.4552750442
O	2.5241976735	5.1282191004	-0.9862254621
H	2.2701505156	4.9192023361	-1.8915685569
H	2.7546934450	2.8514239721	1.0949143480

O	4.3449257499	2.7863667423	1.7570783518
H	4.8198030914	3.5814116836	1.4890377443
O	1.8529068769	3.0176214204	0.7287808501
H	4.6557748400	2.0803032069	1.1513309708

D-Glc-2d

0 1

C	6.2669963559	-0.7493601475	0.7484881596
C	5.8136211501	-2.0643618812	0.1178937489
C	4.5538535737	-1.8556772082	-0.7238263582
C	3.4962399777	-1.1096441723	0.0828132131
O	4.0312346295	0.0961222143	0.5951549245
C	5.1034315993	-0.1085859835	1.5086890573
H	6.5865237865	-0.0571279590	-0.0436866689
O	7.3324502133	-0.9578353609	1.6663951398
H	5.5854519943	-2.7746765060	0.9200900556
O	6.8487800112	-2.6677904906	-0.6435559306
H	4.7964971383	-1.2536762000	-1.6092179177
O	3.9902927186	-3.0996524106	-1.1102250320
H	3.1389369829	-1.7350555858	0.9159035562
H	4.7818643309	-0.7773935010	2.3209833754
C	5.4454407072	1.2599606049	2.0900803848
H	8.1117058485	-1.2374280289	1.1712687295
H	7.0792240052	-2.0780252780	-1.3738221512
H	4.5071710819	-3.4697497193	-1.8354574442
H	4.5312956750	1.7187000047	2.4727960762
H	5.8534185225	1.9023448504	1.2985725984
O	6.3501788417	1.1655381752	3.1812443419
H	7.0947515543	0.6182844688	2.8893085298
O	2.4508918635	-0.7839088928	-0.7772187755
O	-2.7926623137	0.3135096392	0.3019795631
C	2.5773046250	2.4788810547	-0.6648988890
C	3.3607191665	3.4682315195	0.1346950111
O	2.7498087113	2.2685041221	-1.8568011867
H	4.4084119655	3.4277353465	-0.1661824292
H	3.2605779065	3.2940785029	1.2053147583
H	2.9763475685	4.4663490944	-0.1024657474
C	1.1983348183	-0.4912579472	-0.1703915540
C	0.7597915975	0.9143276092	-0.5545368027
C	-0.6472674412	1.2240504532	-0.0478365351
C	-1.6228755264	0.1054327596	-0.4138559190
O	-1.0876428797	-1.1543410976	-0.0138246844
C	0.1421798594	-1.4836725214	-0.6648398257
H	1.2760444534	-0.5697887916	0.9203661708

H	0.7980636816	1.0238124296	-1.6414434484
O	1.6258606802	1.8814908743	0.0587165917
H	-0.6250260087	1.2988254656	1.0458694700
H	0.0187323822	-1.3885861601	-1.7531294303
C	0.4543272509	-2.9454329002	-0.3370997812
H	-0.4944670353	-3.4866976077	-0.2931353007
H	0.9364667086	-3.0217023925	0.6454529793
O	1.2372822159	-3.5709990390	-1.3374856423
H	2.1680374232	-3.2942388302	-1.2492943268
H	-1.8128377482	0.0865076610	-1.4975931736
O	-5.8262583015	-3.4159115108	0.0084757146
C	-4.0313391561	-0.1430516111	-0.2505458583
C	-4.2240818280	-1.6504803048	-0.0336654457
C	-5.6659001034	-2.0473252797	-0.3192402195
C	-6.6421882498	-1.1587807897	0.4556591182
O	-6.4091298749	0.1943354893	0.1298362987
C	-5.1093735472	0.6707607267	0.4767834425
H	-4.0539511095	0.0723737600	-1.3270173456
H	-3.9878493993	-1.8809183907	1.0136951157
O	-3.3949474112	-2.4168092767	-0.8944225734
H	-5.8678178319	-1.8868430969	-1.3858490074
O	-6.4999051564	-1.4110436885	1.8254020272
H	-4.9492793698	0.5600655264	1.5578896057
C	-5.0881168541	2.1597786441	0.1627372386
H	-6.6551500940	-3.7277047197	-0.3730552690
H	-4.1081433224	2.5655248212	0.4380039356
H	-5.8426085333	2.6442169799	0.7893265561
O	-5.4028797718	2.4608492661	-1.1854759986
C	-7.4483243002	-0.7199975295	2.6322939401
H	-7.6746866996	-1.3528835753	0.1404475104
H	-8.4695972675	-0.9222145429	2.2875562638
H	-7.2675575567	0.3599579764	2.6193068734
H	-7.3267916754	-1.0944274608	3.6493070486
H	-2.4754754405	-2.2622262912	-0.6191708482
H	-2.4154716184	2.4265375008	-1.8953470410
O	-3.0728467965	2.3804620086	-2.6161284560
H	-2.9584514121	1.5008689866	-2.9953637384
H	-0.2411004841	3.0836474125	-2.2064840657
O	0.2588909443	3.4232690497	-2.9688151376
H	1.1477931930	3.0650607626	-2.8324484769
H	-0.8629661847	3.1860532660	-0.0255813101
O	-0.2761408484	4.4253802308	0.9931090988
H	-0.6851184877	5.2701520426	0.7696109021
O	-1.1202268294	2.4333139939	-0.6090050135
H	-4.5858453280	2.3904304932	-1.7223966007
H	0.6595664632	4.5359122635	0.7825539747

D-Glc-2e-1

0 1

C	6.4062030756	-0.7534698454	0.4228511681
C	5.8132907651	-2.1621751995	0.3769406121
C	4.5134482462	-2.1969608073	-0.4312568854
C	3.5590073180	-1.1347545548	0.1012196092
O	4.2001808402	0.1208730128	0.0406779012
C	5.3404118537	0.2417884388	0.8835726973
H	6.7369254430	-0.4743517850	-0.5887636942
O	7.4943824887	-0.6857539745	1.3296268620
H	5.5912792311	-2.4794123056	1.4020604654
O	6.7516084738	-3.1068610470	-0.1168097651
H	4.7209250302	-1.9775045444	-1.4872871748
O	3.8796603708	-3.4592904284	-0.3022642961
H	3.2735642516	-1.3623003441	1.1399035339
H	5.0542177682	0.0223402938	1.9236978908
C	5.8002384437	1.6845846094	0.7645783403
H	8.1653973205	-1.3159149482	1.0400290531
H	6.9923072462	-2.8618101814	-1.0204945312
H	4.4408900330	-4.1297089616	-0.7114807887
H	6.0931552424	1.8846655801	-0.2742416249
H	6.6652554471	1.8457335316	1.4095839041
O	4.7972168953	2.5985069981	1.1842348949
H	4.0345254237	2.4929908033	0.6008294257
O	2.4295605168	-1.0759820846	-0.7163829194
O	-2.7964486832	0.1019740773	0.4109992662
C	-1.2233911793	3.3552056148	0.0667144380
C	1.1955035313	-0.7233472921	-0.0935923274
C	0.7598412433	0.6897145343	-0.4994503839
C	-0.6315805918	0.9835594818	0.0597438440
C	-1.6159304851	-0.1259251549	-0.2785281608
O	-1.0889308228	-1.3666864528	0.1652711033
C	0.1203417364	-1.7381697707	-0.5018371091
H	1.3114545077	-0.7692193700	0.9957788515
H	0.7140429985	0.7416273313	-1.5926134673
H	-0.5873287159	1.0889920509	1.1459473244
H	-0.0350260646	-1.7065781022	-1.5897047760
C	0.4120393690	-3.1797863944	-0.0867271777
H	-0.5445906893	-3.7091469014	-0.0570803010
H	0.8472503471	-3.2031718700	0.9203758154
O	1.2383019140	-3.8593166015	-1.0129952581
H	2.1692228817	-3.6310667779	-0.8280073049
H	-1.7900004539	-0.1688237564	-1.3633342666

O	-6.2632687337	-3.2399574628	0.1823622046
C	-4.0077145644	-0.3030887763	-0.2256076586
C	-4.4363753512	-1.7001654653	0.2262964713
C	-5.8173716117	-2.0023410957	-0.3428563681
C	-6.8084943632	-0.8676001275	-0.0454009536
O	-6.2924541573	0.3664599507	-0.5053627020
C	-5.0662173639	0.7392709336	0.1289476358
H	-3.8627607057	-0.3100293022	-1.3127917219
H	-4.4766849975	-1.7185456585	1.3240656331
O	-3.5502740563	-2.7043793238	-0.2441728452
H	-5.7284736164	-2.0656289386	-1.4346154713
O	-7.0771108458	-0.8498239550	1.3279491899
H	-5.2125269987	0.7608286564	1.2169526612
C	-4.6998624331	2.1509322392	-0.3025685483
H	-7.0099745853	-3.5474996661	-0.3449676413
H	-3.9459037442	2.5430678318	0.3870632693
H	-5.5935074551	2.7744327698	-0.2140485943
O	-4.2468910657	2.2504066567	-1.6460001521
C	-8.1181011782	0.0509856167	1.6928127539
H	-7.7364665445	-1.0149120905	-0.6107645845
H	-9.0283986068	-0.1604807016	1.1187436509
H	-7.8187364640	1.0914551002	1.5287684109
H	-8.3119378517	-0.1071058647	2.7542113981
H	-2.6435411899	-2.4662009518	0.0168257090
O	-1.1285732488	2.1722450317	-0.5644381727
C	-0.8611025726	3.4563832558	1.5153632014
H	-0.9839397982	4.4918025595	1.8283196066
H	-1.5150327893	2.8158197150	2.1150526050
H	0.1725137052	3.1417116561	1.6806099429
O	-1.6254575349	4.2954536104	-0.5981521166
H	-3.2981112356	2.0684110925	-1.6577959775
O	1.6719929690	1.6826484454	-0.0883845337
H	2.7980815559	0.5562489784	2.5531934553
H	1.7857489133	3.3189096155	-0.7645783107
H	2.9769999856	1.7200831885	-2.1348153700
O	2.1019187593	1.2246200375	2.6029654024
O	1.9646105039	4.2086826352	-1.1287450725
O	3.1639587919	1.0373794933	-2.7924638836
H	1.3081758369	0.7350611337	2.8530699747
H	2.8929381521	4.1806618741	-1.3848913720
H	3.0047884037	0.2160942082	-2.3033091011
H	1.8291050092	1.5955439125	0.8781491671

D-Glc-2e-2

0 1

C	7.1799360000	0.4990990000	0.3247980000
C	6.9503900000	-0.9131670000	-0.2092000000
C	5.6573720000	-0.9816210000	-1.0256970000
C	4.5037210000	-0.3928660000	-0.2204690000
O	4.8252360000	0.9204310000	0.1971300000
C	5.9380350000	0.9761200000	1.0817910000
H	7.3562650000	1.1818610000	-0.5187590000
O	8.2851680000	0.5410550000	1.2183030000
H	6.8614080000	-1.5980220000	0.6413500000
O	8.0611080000	-1.3810210000	-0.9587780000
H	5.7696350000	-0.3987970000	-1.9492920000
O	5.3221100000	-2.3270280000	-1.3265870000
H	4.3003660000	-1.0177760000	0.6630450000
H	5.7626650000	0.3217810000	1.9489620000
C	6.0383810000	2.4237550000	1.5531790000
H	9.0932740000	0.3943440000	0.7120430000
H	8.1901330000	-0.7980510000	-1.7186490000
H	5.9245090000	-2.6589120000	-2.0027030000
H	5.0653720000	2.7365620000	1.9391750000
H	6.2983540000	3.0674000000	0.7022500000
O	6.9761810000	2.5794900000	2.6087430000
H	7.7982700000	2.1504030000	2.3266000000
O	3.3808250000	-0.3171910000	-1.0417980000
O	-1.9407960000	-0.2678960000	0.2040020000
O	-0.7288540000	2.1271130000	-0.7590260000
C	2.1241160000	-0.2442460000	-0.3765530000
C	1.3965470000	1.0411240000	-0.7806530000
C	-0.0074610000	1.0354790000	-0.1766690000
C	-0.7491850000	-0.2593950000	-0.4995080000
O	0.0347700000	-1.3586520000	-0.0571700000
C	1.2791340000	-1.4684350000	-0.7521020000
H	2.2765710000	-0.2442820000	0.7107750000
H	1.3148050000	1.0695890000	-1.8724030000
O	2.0962010000	2.2128550000	-0.4137260000
H	0.0476950000	1.1585280000	0.9081940000
H	1.0923840000	-1.4730320000	-1.8355070000
C	1.8994810000	-2.8119040000	-0.3613170000
H	1.0812080000	-3.5205360000	-0.2092790000
H	2.4523470000	-2.7200530000	0.5817190000
O	2.7224100000	-3.3481660000	-1.3816980000
H	3.5867970000	-2.8975160000	-1.3754620000
H	-0.9243940000	-0.3496380000	-1.5821890000
O	-4.3987470000	-4.2538240000	1.1630330000
C	-1.5142880000	2.8874510000	0.0164030000
C	-2.1956860000	3.9620140000	-0.7672360000

H	-2.4917220000	3.5961310000	-1.7518290000
H	-1.4880290000	4.7855880000	-0.9076500000
H	-3.0605700000	4.3268150000	-0.2134250000
C	-3.0017130000	-1.0955480000	-0.2839860000
C	-3.0393550000	-2.4073340000	0.5095830000
C	-4.3640910000	-3.1277200000	0.3037730000
C	-5.5430060000	-2.1819760000	0.5513700000
O	-5.4406320000	-1.0744750000	-0.3176690000
C	-4.2806780000	-0.2703950000	-0.1102990000
H	-2.8400220000	-1.3217650000	-1.3459710000
H	-2.9284640000	-2.1606330000	1.5739640000
O	-2.0029970000	-3.2884820000	0.1052890000
H	-4.4304510000	-3.4478060000	-0.7434970000
O	-5.5545460000	-1.8063310000	1.8995150000
H	-4.2893440000	0.1397680000	0.9076780000
C	-4.3521580000	0.8662790000	-1.1061180000
H	-5.1180280000	-4.8321290000	0.8822310000
H	-4.6171520000	0.4689340000	-2.0930500000
H	-3.3736060000	1.3484600000	-1.1791170000
C	-6.6578030000	-0.9753020000	2.2500060000
H	-6.4905830000	-2.6650720000	0.2828770000
H	-7.5998040000	-1.4163940000	1.9029010000
H	-6.5467180000	0.0286870000	1.8270590000
H	-6.6654600000	-0.9090740000	3.3385510000
H	-1.1612690000	-2.8009650000	0.1360380000
O	-1.6313480000	2.7201280000	1.2202050000
H	2.3029680000	2.1723110000	0.5294260000
O	-5.3323850000	1.8031000000	-0.6687160000
H	-4.7341180000	4.5475910000	-2.1512910000
H	-7.0539340000	1.1905420000	-0.6236180000
H	-4.8246180000	2.4784820000	0.9647920000
O	-5.4089720000	3.9047050000	-2.4008630000
O	-7.9980810000	0.9356020000	-0.6163070000
O	-4.5050850000	2.8335780000	1.8170170000
H	-6.2484650000	4.3671900000	-2.2908690000
H	-8.4599230000	1.6916390000	-0.9938490000
H	-3.5397250000	2.8401880000	1.7233140000
H	-5.3512840000	2.5592420000	-1.2983040000

D-Glc-2f

-1 1

C	-6.7726973071	-0.0756791411	0.0076507704
C	-6.4111732853	-1.5159032785	-0.3573243477
C	-5.0471952983	-1.8888778524	0.2253316495

C	-4.0101147424	-0.8474135988	-0.1728761130
O	-4.4448019779	0.4386235734	0.2551034180
C	-5.6391680357	0.8745576771	-0.3897625210
H	-6.9181661073	-0.0050057687	1.0949960273
O	-7.9501055950	0.3496903945	-0.6658591690
H	-6.3641872756	-1.6005686726	-1.4485394348
O	-7.4152934610	-2.4313077613	0.0538951763
H	-5.1088168028	-1.9125811717	1.3218226554
O	-4.5902111125	-3.1376781421	-0.2693644331
H	-3.8677791576	-0.8382842727	-1.2637401443
H	-5.5016498115	0.8530598926	-1.4808122104
C	-5.8703262889	2.3126114405	0.0644355543
H	-8.7051030068	-0.1163854568	-0.2876281109
H	-7.4835817010	-2.4028009476	1.0177378867
H	-5.1793035621	-3.8306975006	0.0538082567
H	-4.9538747191	2.8845415423	-0.0995555969
H	-6.0998843019	2.3239276092	1.1381189666
O	-6.8989294797	2.9477924286	-0.6803923636
H	-7.6671728937	2.3570311830	-0.6673496704
O	-2.8221525941	-1.1291542583	0.4808001871
O	2.4639833682	-0.0982332054	-0.3811556724
C	-1.5999181207	-0.7712094091	-0.1723537402
C	-1.1277428251	0.6150507829	0.2637311256
C	0.3118195586	0.9154389265	-0.1874960048
C	1.2017137850	-0.2793539110	0.1783882318
O	0.6704267917	-1.4885719254	-0.3751002102
C	-0.5860306302	-1.8541398175	0.1912675950
H	-1.7525080483	-0.7786668154	-1.2594307769
H	-1.1570706021	0.6567006932	1.3632130445
O	-1.9646652824	1.6305684783	-0.2788694502
H	0.3028567623	0.9574846331	-1.2938300714
H	-0.4915835063	-1.9256785134	1.2848267147
C	-0.9271569763	-3.2352925100	-0.3738401534
H	-0.0213561361	-3.8462482821	-0.3314614525
H	-1.2271561612	-3.1410694535	-1.4253123672
O	-1.9161074132	-3.9201282461	0.3743414945
H	-2.7902245481	-3.5362901580	0.1799506768
H	1.2750214953	-0.3995409162	1.2695848901
O	5.4486097414	-3.8736957211	0.0557643979
C	3.6007739394	3.6443178806	-0.3596394987
C	3.0121940813	4.3288792112	-1.5509923645
H	2.4784077945	3.6214568409	-2.1865222813
H	3.8336444212	4.7625668208	-2.1311292950
H	2.3500429369	5.1309450283	-1.2267642744
C	3.5846081308	-0.6464750623	0.3092573636
C	3.8446832529	-2.1090190722	-0.0724463387

C	5.1711254770	-2.5656933945	0.5230753051
C	6.3027873586	-1.5897419577	0.1847082999
O	5.9497868966	-0.2844065543	0.6019655960
C	4.7889931421	0.2289083958	-0.0498989470
H	3.4112282770	-0.5880081852	1.3924735981
H	3.8895383808	-2.1819638317	-1.1678289804
O	2.8398509475	-2.9739772083	0.4313425686
H	5.0716849528	-2.5680497880	1.6156446728
O	6.5644256137	-1.6527781126	-1.1878792291
H	4.9391088192	0.2181452322	-1.1365697385
C	4.6535353810	1.6717358080	0.4127290096
H	6.1228705454	-4.2655250723	0.6229195631
H	5.6477859542	2.0935035218	0.5813112613
H	4.0641687687	1.7523821453	1.3281493871
O	4.0141240749	2.4065052434	-0.6418872880
C	7.6881299031	-0.8748555769	-1.5894029327
H	7.2066655940	-1.8326197384	0.7559794664
H	8.5742529147	-1.1476982913	-1.0035109704
H	7.4906783535	0.1959160228	-1.4738163614
H	7.8638469806	-1.0979275236	-2.6421880341
H	1.9832970627	-2.6632669644	0.0843809497
O	3.7400809656	4.1527333107	0.7428365809
H	-2.8815303122	1.4562742797	-0.0043775024
O	0.7913515398	2.0977379688	0.3597136599
H	0.2380514061	3.2503727370	-0.7641743634
H	-0.2420039484	2.9877692094	1.3797373015
H	1.5124170353	1.8893732387	1.8584035175
O	-0.2851171237	3.7928362375	-1.4139043499
O	-0.7594997460	3.5590174194	2.0080239614
O	1.8763804249	1.8196765962	2.7855348281
H	-1.1527179564	3.3717849281	-1.3543420816
H	-0.2918362746	3.4808760273	2.8461917734
H	2.4313642994	1.0325596823	2.7766804627

D-Glc-IN1

-1 1

C	6.5836386316	-0.6043055238	-0.1965203647
C	6.0348681983	-1.5292825563	0.8886839105
C	4.7064282325	-2.1418110341	0.4430819675
C	3.7546234464	-1.0398341504	-0.0125515565
O	4.3571853008	-0.2743148222	-1.0362745569
C	5.5188098267	0.4204783417	-0.5986731181
H	6.8460158783	-1.2009722891	-1.0820398030
O	7.7243761254	0.1119860912	0.2583650929

H	5.8640408791	-0.9414121928	1.7973100660
O	6.9721066515	-2.5316436693	1.2545103125
H	4.8765656178	-2.8235296578	-0.4013929404
O	4.0723435321	-2.8286983610	1.5124041990
H	3.5000331717	-0.3899946183	0.8366635120
H	5.2711606542	1.0493104312	0.2703386730
C	5.9532723203	1.3136045150	-1.7562471701
H	8.4498300459	-0.5122229399	0.3780763406
H	7.1607274659	-3.0773012831	0.4791630319
H	4.6346379027	-3.5657445648	1.7803832034
H	5.0863590283	1.8711141678	-2.1175706085
H	6.3330533494	0.6919354786	-2.5778929772
O	6.9251927197	2.2682678075	-1.3540052116
H	7.6322958629	1.7828640572	-0.9034073456
O	2.6063929020	-1.6092330964	-0.5656417462
O	-2.6985670134	-0.6768848346	0.3375920165
O	-1.0708571952	1.4849711974	-0.7478677576
C	2.1086754497	1.9766467140	-1.2699040034
C	2.9085226479	3.1044918519	-0.7014186075
O	1.8431878408	1.8277543809	-2.4503632697
H	3.6448071523	2.7292460745	0.0130317245
H	2.2283662437	3.7729401082	-0.1634229489
H	3.4002151403	3.6542823568	-1.5028317928
C	1.3667478402	-1.2139380521	0.0013084809
C	0.8185524586	0.0526894492	-0.6330902431
C	-0.5656636150	0.3611488158	-0.0698816229
C	-1.4620205739	-0.8641497767	-0.2758517884
O	-0.8798643372	-1.9968705365	0.3469645516
C	0.3763541020	-2.3597942578	-0.2245698405
H	1.4844176784	-1.0582837627	1.0800827998
H	0.7751352267	-0.0597610089	-1.7193899494
O	1.6901518773	1.1436349509	-0.3069699636
H	-0.4806993904	0.5544036108	1.0026097883
H	0.2460767794	-2.5226021693	-1.3050513695
C	0.8423181123	-3.6724047631	0.3909938288
H	1.6168792577	-4.1019040159	-0.2523541531
H	-0.0062191072	-4.3616136156	0.4051260038
O	1.3172114977	-3.5462558421	1.7218422402
H	2.2493402633	-3.2644843949	1.6796547990
H	-1.5909051275	-1.0620830589	-1.3497075065
O	-6.8512633821	-2.9442887582	-0.3745623589
C	-1.6660322851	2.5650555240	0.0248717647
C	-1.5407661766	3.7798668830	-0.8897292884
H	-1.9933836328	3.6004195231	-1.8693388455
H	-0.4822030680	4.0161283724	-1.0306107745
H	-2.0381592376	4.6331635994	-0.4208570995

C	-3.8613936852	-0.7258779360	-0.4837211442
C	-4.7195596394	-1.9132661773	-0.0567210487
C	-6.0111314350	-1.9138968913	-0.8621908237
C	-6.6971394199	-0.5399912885	-0.7997757035
O	-5.7992696525	0.4778393162	-1.2003910513
C	-4.6409468754	0.5846014622	-0.3688004097
H	-3.5657694629	-0.8628594452	-1.5306833217
H	-4.9508647308	-1.8219012081	1.0128460260
O	-4.0473124632	-3.1398421430	-0.3027617968
H	-5.7605156526	-2.0965396943	-1.9145622331
O	-7.1818886731	-0.3387449967	0.4970864287
H	-4.9466039061	0.7452584350	0.6726226146
C	-3.8607005417	1.8110578971	-0.8311678218
H	-7.5362396570	-3.1197660982	-1.0298949678
H	-4.5755817983	2.5968469486	-1.1032389610
H	-3.2542563424	1.5708225969	-1.7093764588
O	-3.0570766678	2.2500118241	0.2497249800
C	-7.9619791417	0.8446482339	0.6385286277
H	-7.5191941205	-0.4894730205	-1.5239362284
H	-8.7628577392	0.8696810786	-0.1103868227
H	-7.3428847664	1.7424003208	0.5404817189
H	-8.3975632395	0.8137790369	1.6377380464
H	-3.2459164608	-3.1563901092	0.2363223341
H	0.4145545120	2.4314601386	1.9122983363
O	1.2704182856	2.2452722447	2.3668796893
H	1.7627624087	1.7547596870	1.6960917213
H	-1.8702242209	1.5698611827	2.3541337078
O	-2.2742856808	0.8477160023	2.8930195145
H	-2.5688298613	0.2226684043	2.2139758576
H	-1.7169788830	4.0636678246	2.0319916676
O	-2.0451033765	4.8521395590	2.5411695888
H	-2.3429379932	5.4720629101	1.8670492411
O	-1.1273982888	2.7233302087	1.2300669675

D-Glc-IN2

-1 1

C	6.7359914210	-0.1782284001	0.2723040104
C	6.4811604671	-1.6852927472	0.2067958700
C	5.2147683106	-2.0061077881	-0.5863592223
C	4.0578156567	-1.1697787991	-0.0568316448
O	4.3947371140	0.2020176164	-0.0833033867
C	5.4791382647	0.5334230376	0.7815427127
H	6.9686244487	0.1933164218	-0.7370842913
O	7.8089919291	0.1055917991	1.1560557838

H	6.3523792616	-2.0563704587	1.2297988468
O	7.6102724947	-2.3768445927	-0.3074905212
H	5.3651515467	-1.7633509279	-1.6462240586
O	4.8461992994	-3.3686499379	-0.4361849912
H	3.8181354275	-1.4699386430	0.9756918855
H	5.2545659252	0.1849961946	1.8013435056
C	5.6160709230	2.0430853656	0.7899786855
H	8.5619992663	-0.4350453692	0.8857221744
H	7.7637540947	-2.0850874403	-1.2162493396
H	5.4365504634	-3.9158513772	-0.9669757469
H	5.6510168917	2.4079525087	-0.2428089439
H	6.5581123088	2.3074354618	1.2803842251
O	4.5383943546	2.6423879544	1.4955249303
H	4.0807101245	3.2962319292	0.9202500075
O	2.9594113379	-1.3622107804	-0.8902032888
O	-2.3832790782	-0.5595125667	0.1697797913
C	0.5787873281	2.4247749117	-0.9638517379
C	1.7032508364	-1.0157389927	-0.3203040826
C	1.0351511087	0.1396560423	-1.0283359346
C	-0.2802385520	0.4085795448	-0.3352463181
C	-1.2096327429	-0.7582327260	-0.5399433498
O	-0.5517022809	-1.8923067107	0.0430167457
C	0.7415660927	-2.2167878355	-0.4872975959
H	1.8277531850	-0.7906828906	0.7451266048
H	0.8573982603	-0.1461908455	-2.0768263504
O	1.6465707308	1.4108771482	-0.9861568544
H	-0.1282982525	0.5270899532	0.7468741712
H	0.6474577442	-2.4498424395	-1.5574753583
C	1.1958632553	-3.4766219204	0.2525341827
H	0.3161190223	-4.1097043291	0.3954820781
H	1.5938178214	-3.2120312609	1.2400934099
O	2.1334069181	-4.2422090136	-0.4841314186
H	3.0042942707	-3.8067264241	-0.4686417567
H	-1.4103447136	-0.9522718075	-1.6021846416
O	-5.5342434632	-4.1555085249	0.5870439303
C	-5.0698036929	3.4441149638	-0.4832633036
C	-6.0342854491	4.3810966550	0.1686626609
O	-4.1010705088	3.7833777799	-1.1482603036
H	-5.9112137295	5.3831495680	-0.2403145510
H	-5.8261717076	4.4010940347	1.2430885118
H	-7.0600756386	4.0340181473	0.0302462870
C	-3.5820537302	-1.1196436544	-0.3564649223
C	-3.8564670032	-2.4862801314	0.2726687988
C	-5.2579370346	-2.9488009287	-0.1005104427
C	-6.2961652770	-1.8629369101	0.2183630767
O	-5.9548162572	-0.6563201662	-0.4379835925

C	-4.6963627950	-0.1199940479	-0.0370436117
H	-3.4938215707	-1.2342125110	-1.4449401465
H	-3.7840918347	-2.3854576563	1.3638430185
O	-2.9313437224	-3.4599112118	-0.1864920906
H	-5.2926735918	-3.1140951835	-1.1845230105
O	-6.3760282063	-1.7076074455	1.6060707952
H	-4.7006361360	0.0784378646	1.0420203916
C	-4.4685166914	1.1746692079	-0.7794846098
H	-6.2990915262	-4.5740023425	0.1752283588
H	-4.6682304804	1.0569038836	-1.8484080984
H	-3.4386907255	1.5026294723	-0.6342970596
O	-5.3646963773	2.1633607168	-0.2421589876
C	-7.4087285599	-0.8193980327	2.0225604774
H	-7.2755017100	-2.1365684650	-0.1921667559
H	-8.3701592037	-1.1143843376	1.5850515411
H	-7.1806623512	0.2134265546	1.7393579609
H	-7.4643553333	-0.8917239740	3.1091397050
H	-2.0312692696	-3.1460359057	0.0115456037
O	-0.6557683866	1.6332671847	-0.9192633308
C	0.5714922414	3.1785382140	-2.2814276764
H	-0.2778899558	3.8677345606	-2.2975345710
H	1.4935658503	3.7580787093	-2.3725602663
H	0.4921650065	2.4912170366	-3.1276530136
O	0.7151290769	3.1869063314	0.1173123634
H	-0.8151001770	3.9417709635	0.4131950350
H	2.1807080330	3.9859115520	0.0590139250
O	-1.6722396909	4.3922270322	0.6125463826
O	3.0837700539	4.4093160904	0.0347664562
H	-2.3119548539	4.0243976425	-0.0119137340
H	3.0001776962	5.2262932724	0.5389121796
H	1.4243011998	2.2739404590	1.4607828226
O	1.8876431493	1.8441001527	2.2181681729
H	2.8296213484	1.9150916345	1.9851784365

D-Glc-IN3

-1 1

C	-6.2091361406	-2.0794284253	0.1792925159
C	-5.5652549009	-3.4658664649	0.1637872074
C	-4.1398100799	-3.4000183911	0.7117658664
C	-3.3540338208	-2.3092473870	-0.0059168799
O	-4.0332130819	-1.0708073561	0.1253501921
C	-5.3009244436	-1.0661427467	-0.5244824441
H	-6.3475217096	-1.7559551626	1.2208370249
O	-7.4608307532	-2.0840004667	-0.4949902992

H	-5.5290061814	-3.8231680155	-0.8712152450
O	-6.3474091090	-4.4184024203	0.8689601358
H	-4.1661725708	-3.1558180041	1.7825822014
O	-3.4399414116	-4.6171912375	0.5020874216
H	-3.2433013764	-2.5527632986	-1.0729287692
H	-5.1768337938	-1.3540485378	-1.5791346393
C	-5.8261395764	0.3645367251	-0.4572165131
H	-8.0918115924	-2.5871535591	0.0332971504
H	-6.4086473274	-4.1434564521	1.7937301006
H	-3.8879745470	-5.3165204448	0.9938301178
H	-5.0464636665	1.0420628830	-0.8126976660
H	-6.0649581795	0.6219852791	0.5830101436
O	-6.9563856755	0.5525438524	-1.2971507041
H	-7.5887723655	-0.1503962677	-1.0856532595
O	-2.1166825648	-2.1908508126	0.6083337605
O	2.9387542210	-0.5433298865	-0.7426076219
C	-0.8490774967	1.6194323984	-1.2651132128
C	-0.9960425878	-1.9154507069	-0.2239801107
C	-0.7154165989	-0.4403795509	-0.3495272038
C	0.5899009129	-0.2136954317	-1.0790769731
C	1.6867601037	-0.7322065570	-0.1847308404
O	1.4559027113	-2.1460583068	-0.1035921778
C	0.2222458602	-2.5347734156	0.5092431231
H	-1.1281408389	-2.3875256041	-1.2050107713
H	-0.6477339831	-0.0364753725	0.6711659037
O	-1.5856701349	0.3687087131	-1.1133333227
H	0.6171783404	-0.7803387977	-2.0204929481
H	0.2063328928	-2.1803060867	1.5495495694
C	0.2229887741	-4.0648800251	0.4936494354
H	1.2168440833	-4.3980942047	0.8039199158
H	0.0435140196	-4.4221596634	-0.5282833844
O	-0.7042128848	-4.6373924750	1.3988547266
H	-1.6084889780	-4.5258545580	1.0529551347
H	1.6345016316	-0.2925530124	0.8199877820
O	7.0126150543	-2.6654001948	0.5999865988
C	4.0259992337	-0.4811462306	0.1813972160
C	4.8927423716	-1.7265252627	0.0270587052
C	6.1618692286	-1.5607752926	0.8498058119
C	6.8520285219	-0.2270178039	0.5321997428
O	5.9572203903	0.8478476176	0.7386511950
C	4.7980582631	0.8063890596	-0.0981841245
H	3.6381567861	-0.4509582527	1.2067230148
H	5.1566094931	-1.8442499945	-1.0327191125
O	4.2115966800	-2.8858381416	0.4867470244
H	5.8843737718	-1.5292876538	1.9108025202
O	7.3377182364	-0.2704739240	-0.7801082456

H	5.1028252592	0.8246020304	-1.1529542574
C	3.9806154916	2.0622278544	0.1635758162
H	7.7034659457	-2.6768545340	1.2726614464
H	3.1538845593	2.0927437568	-0.5533543420
H	4.6241887171	2.9284551740	-0.0246039675
O	3.4925756669	2.1383283393	1.4938116665
C	8.1366547318	0.8554863891	-1.1287675740
H	7.6755318674	-0.0481352121	1.2344178471
H	8.9477247070	0.9922735963	-0.4031704560
H	7.5347791174	1.7690877020	-1.1771546779
H	8.5591258999	0.6487032408	-2.1126437923
H	3.2861885056	-2.8421118483	0.1911961416
O	0.5614973062	1.1700509700	-1.3401483530
C	-1.2397907693	2.2203020266	-2.6011607432
H	-2.2992992735	2.4900466701	-2.5798682810
H	-0.6529960767	3.1261494329	-2.7735375183
H	-1.0635971254	1.5160222627	-3.4178973996
O	-1.0041842416	2.4267669223	-0.2178448017
H	-2.2642338170	1.7637877068	0.8410855482
H	0.1514410869	3.7240492484	-0.3508618378
O	-2.9612149069	1.3936844579	1.4309039728
O	0.8569779585	4.4155393094	-0.3687292097
H	-3.2406855747	0.5776020004	0.9882719187
H	1.6249815809	3.9742108276	0.0123566038
H	0.1164612896	2.0910399373	1.0618829657
O	0.7362697109	1.9937342555	1.8301475923
H	0.6300933299	2.8106741900	2.3315709277
H	2.5220580723	2.0119881318	1.4973189555

D-Glc-IN4

-1 1

C	-6.8250375904	0.3692123612	-0.2826762569
C	-6.6538175047	-1.0740713920	0.1853107204
C	-5.3331759473	-1.2489587902	0.9375838819
C	-4.1799420360	-0.6901000934	0.1099634080
O	-4.4456535552	0.6557663757	-0.2405278262
C	-5.5893258326	0.8080276538	-1.0731801442
H	-6.9299201100	1.0260780890	0.5925533695
O	-7.9604344073	0.5067735625	-1.1281007287
H	-6.6426223211	-1.7264449689	-0.6948796878
O	-7.7546805773	-1.5107411113	0.9679787419
H	-5.3720362162	-0.7012994284	1.8883286765
O	-5.0651215228	-2.6228135591	1.1690960881
H	-4.0452251063	-1.2860225152	-0.8061029801

H	-5.4863403239	0.1814033980	-1.9719386960
C	-5.6295456880	2.2768283655	-1.4851690000
H	-8.7552892829	0.3870534458	-0.5948202895
H	-7.7960805012	-0.9757702083	1.7717805364
H	-5.6350248213	-2.9420144166	1.8783868893
H	-4.6574303594	2.5541088977	-1.8987897905
H	-5.8219319940	2.8988308500	-0.6008653140
O	-6.5974078187	2.5213608045	-2.4959391303
H	-7.4315263871	2.1321826524	-2.1923948827
O	-3.0266759981	-0.7112157537	0.8892659024
O	2.2354206019	-0.9992090647	-0.5910852496
O	1.1824320934	1.5055053083	0.6439096076
C	-1.7872328863	-0.6696858754	0.1809565531
C	-0.9533548640	0.5066030607	0.6633353800
C	0.4315902876	0.4861226714	0.0088688537
C	1.0905247172	-0.8846876243	0.1966137191
O	0.2220791261	-1.9062755475	-0.2634642012
C	-1.0140408524	-1.9682189301	0.4442673343
H	-1.9760074383	-0.5738448662	-0.8951783968
H	-0.8217856602	0.4102768613	1.7511967339
O	-1.6225561707	1.7202682278	0.3702186974
H	0.3247374635	0.6650887634	-1.0657587803
H	-0.8155704580	-2.0606120317	1.5225136657
C	-1.7289324376	-3.2387266947	-0.0232097996
H	-0.9597400778	-3.9796001476	-0.2556448487
H	-2.3031017860	-3.0427704886	-0.9376564393
O	-2.5535996144	-3.8073084012	0.9788146797
H	-3.3811651549	-3.2972403573	1.0517454348
H	1.3256992045	-1.0494687669	1.2579657128
O	5.9196949467	-4.0393722317	-0.2853628517
C	1.9708161580	2.4572716228	-0.1202599368
C	1.1911486117	3.0513155677	-1.2916240876
H	0.9740480602	2.3219034212	-2.0746865654
H	1.7966697035	3.8497225785	-1.7277792722
H	0.2537445995	3.4817836864	-0.9327943847
C	3.4791625399	-1.2450795424	0.0511282122
C	4.0038449007	-2.6125224496	-0.3790694586
C	5.3916549143	-2.8218733752	0.2087388469
C	6.3079550035	-1.6316164026	-0.1154266355
O	5.7158958952	-0.4206912004	0.3133317265
C	4.4645007013	-0.1348190981	-0.3159021676
H	3.3435389093	-1.2450580345	1.1397850753
H	4.0581348168	-2.6472060790	-1.4752934024
O	3.1602174294	-3.6518690011	0.0956050639
H	5.2999261681	-2.8648190507	1.3013731285
O	6.5744753431	-1.6335652261	-1.4891439562

H	4.5961573513	-0.0973405462	-1.4046731726
C	4.0269679198	1.2350657049	0.1808376000
H	6.7052066776	-4.2631648509	0.2278021036
H	4.8994393546	1.8973222382	0.2206278261
H	3.6133252608	1.1454552701	1.1893055938
O	3.0634398413	1.7581424004	-0.7217583367
C	7.5159866989	-0.6434818125	-1.8909180734
H	7.2428327797	-1.7031850345	0.4535047661
H	8.4344710494	-0.7204349911	-1.2963677802
H	7.1001244214	0.3643092991	-1.7889032052
H	7.7429555429	-0.8361509402	-2.9399701502
H	2.2814947611	-3.5202665383	-0.2837690892
H	3.3858298225	4.5034370591	0.0894352739
O	3.9757362158	5.2117515455	-0.2864566894
H	3.3820449383	5.7731184517	-0.7962652111
H	2.9846700738	3.0519877659	2.2870616491
O	3.3436763843	2.9695547136	3.2096803139
H	4.1494787865	2.4508254477	3.1152563481
H	1.2476196594	4.5566408365	1.0736121929
O	0.6100354688	5.2972573757	1.2625586373
H	0.5225460281	5.7590811654	0.4218716115
O	2.3732767470	3.3768917734	0.7659150914
H	-1.0551546762	2.4399541657	0.6765322218

D-Glc-RE1

-1 1

C	6.7282955731	-0.2482458602	0.4153661940
C	6.3878901944	-1.5746864204	-0.2595748460
C	5.0786049336	-1.4647647177	-1.0431762338
C	3.9849479908	-0.8860586894	-0.1517117730
O	4.4072435970	0.3530177180	0.3870006589
C	5.5359770246	0.2345819729	1.2459665207
H	6.9452183194	0.5068951588	-0.3537033914
O	7.8451863410	-0.3844370794	1.2856579256
H	6.2662195246	-2.3388291181	0.5161113284
O	7.4474074750	-2.0355196311	-1.0841574699
H	5.2136623255	-0.7935124651	-1.9012468536
O	4.6429099575	-2.7427737993	-1.4789451609
H	3.7524471755	-1.5820881428	0.6690038568
H	5.3254605239	-0.4947856531	2.0424506109
C	5.7543576749	1.6098322399	1.8693758154
H	8.6349920699	-0.5277629850	0.7505389373
H	7.6114395808	-1.3799477227	-1.7750156410
H	5.1880885896	-3.0252787109	-2.2227350000

H	4.8172859257	1.9474280279	2.3171294157
H	6.0451078022	2.3242982012	1.0878286288
O	6.7224338028	1.5741620196	2.9085684430
H	7.4987724209	1.1106386325	2.5589693678
O	2.8589665078	-0.6533343376	-0.9366200148
O	-2.4670116657	-0.3196651620	0.3045711569
O	-0.9895217543	2.0798560871	-0.3964745509
C	2.5347511776	2.6079049442	-0.5583974971
C	3.2001717296	3.6483309275	0.2831035758
O	2.6650803531	2.5085339941	-1.7663921185
H	3.1991224286	3.3764406833	1.3379337234
H	2.6471653552	4.5848870250	0.1569864142
H	4.2193574985	3.8030952465	-0.0737433684
C	1.6152867582	-0.5759164176	-0.2518482732
C	0.9827939003	0.7877490840	-0.4953116073
C	-0.4266334577	0.8576390945	0.0782739077
C	-1.2733247007	-0.3247457795	-0.3954814522
O	-0.5630826551	-1.5192782035	-0.0683646501
C	0.6700428017	-1.6607397459	-0.7778039335
H	1.7638411095	-0.7255056135	0.8239943667
H	0.9549733913	0.9907182808	-1.5690702340
O	1.7356037461	1.8094692912	0.1669727239
H	-0.3965214531	0.8511670056	1.1701873674
H	0.4932889569	-1.5150951766	-1.8525923073
C	1.1770026651	-3.0877903541	-0.5555099026
H	0.3085638687	-3.7480963132	-0.4974496660
H	1.7226408415	-3.1566158862	0.3936409428
O	1.9713079348	-3.5536183409	-1.6314582104
H	2.8667667764	-3.1734710507	-1.5677361169
H	-1.4429099688	-0.2897320712	-1.4810944532
O	-4.9646859004	-4.3210480092	1.0571389173
C	-1.4888056327	2.9645221998	0.4779355476
C	-1.8999132979	4.2340013887	-0.1933125869
O	-1.5507597489	2.7577006451	1.6789062067
H	-2.2194042505	4.0509779705	-1.2193618398
H	-1.0294211731	4.8986266579	-0.2128240335
H	-2.6937335439	4.7127543366	0.3800623913
C	-3.5714543524	-1.0745584273	-0.2260776697
C	-3.6121250646	-2.4507653742	0.4640623548
C	-4.9515969539	-3.1431950734	0.2679831274
C	-6.1042671889	-2.2052631863	0.6172007582
O	-6.0094085262	-1.0494252233	-0.1823356799
C	-4.8420068299	-0.2472369318	0.0308769902
H	-3.4333317674	-1.2163134317	-1.3062117216
H	-3.4512107606	-2.2878846461	1.5384894808
O	-2.6188541410	-3.3265558966	-0.0461484047

H	-5.0618971840	-3.3982505415	-0.7932946433
O	-6.0675540817	-1.9192648259	1.9878927567
H	-4.8206803743	0.1075035543	1.0694787831
C	-5.0432281768	0.9448600112	-0.9145364539
H	-5.7115664413	-4.8657657967	0.7811669289
H	-6.0726593855	1.2993476207	-0.7257345792
H	-5.0348658645	0.5484766800	-1.9472974494
O	-4.1160810241	1.9684003372	-0.7518545815
C	-7.1635493924	-1.1257159656	2.4315885802
H	-7.0684486091	-2.6568266386	0.3512757138
H	-8.1155603512	-1.5801842739	2.1312788932
H	-7.1042112187	-0.1077146871	2.0321702821
H	-7.1051198575	-1.0903333335	3.5199773754
H	-1.7651063464	-2.8598428052	-0.0101801025
H	-4.7495624551	3.3185078229	-1.3508897507
O	-5.1602193629	4.1583842609	-1.7259725374
H	-4.5444203832	4.8594982261	-1.4882090148
H	-4.4065159294	2.5616123567	0.7513763269
O	-4.6108820266	2.9175014741	1.6664218718
H	-3.7415169765	2.9969763393	2.0781775650
H	-3.1812312502	1.8852334383	-2.0839490832
O	-2.6943831644	1.8117739407	-2.9592904087
H	-3.3900359802	1.8272646205	-3.6248444669

D-Glc-RE2

-1 1

C	7.0043702208	0.0389800743	0.1100043796
C	6.5690343598	1.3482487262	-0.5469366483
C	5.2379177746	1.8045108559	0.0453434253
C	4.2172552987	0.6784836097	-0.0737952283
O	4.7001973116	-0.4622743859	0.6107331452
C	5.8810640397	-1.0020617935	0.0283164325
H	7.2264829277	0.2282996904	1.1701195275
O	8.1489583491	-0.5105204345	-0.5305004828
H	6.4418337402	1.1807254778	-1.6221802907
O	7.5618941712	2.3570862495	-0.4338471156
H	5.3684445322	2.0433136299	1.1100820678
O	4.7085473712	2.9266316655	-0.6444516574
H	4.0421146241	0.4321741224	-1.1306646987
H	5.6927060446	-1.2559310607	-1.0258111140
C	6.2021972981	-2.2768552917	0.8036532773
H	8.9102798922	0.0463503209	-0.3286891692
H	7.7204913591	2.5346415523	0.5031357802
H	5.3678409077	3.6323568302	-0.6270914945

H	5.3044203901	-2.8960147285	0.8566165374
H	6.5056032271	-2.0155709613	1.8262732160
O	7.2045941014	-3.0525768590	0.1617805781
H	7.9413311871	-2.4562681411	-0.0404895906
O	3.0346054867	1.0648352107	0.5512574712
O	-2.2877342151	0.5003327539	-0.4213097564
C	2.6131700338	-2.4983720034	-0.3269672990
C	1.8202156083	0.9192991925	-0.1800499471
C	1.2463928761	-0.4842924194	-0.0174436167
C	-0.1915817636	-0.6176233846	-0.5376470116
C	-1.0041215488	0.5139739464	0.1045936219
O	-0.4253955801	1.7770105396	-0.2271613618
C	0.8562842298	1.9837677754	0.3577198778
H	2.0001613763	1.1164432734	-1.2443726648
H	1.2776449689	-0.7596985156	1.0394007948
O	2.0823510201	-1.3640646496	-0.7941189793
H	-0.1677445469	-0.3957172168	-1.6214942616
H	0.7860231515	1.8958846477	1.4516086626
C	1.2412653413	3.4149959572	-0.0238582034
H	0.4017508245	4.0627039850	0.2430617013
H	1.3846035736	3.4702706661	-1.1105700135
O	2.3791943149	3.9051033106	0.6609892113
H	3.1770663744	3.5167609564	0.2560550927
H	-1.0305425574	0.4138541323	1.2001299988
O	-5.3704158134	4.1532557233	-0.1496482358
C	-5.6352956142	-2.7926996655	-0.1170009216
C	-6.9239938681	-3.5394241741	0.0001209085
O	-4.8930533728	-2.8318359965	-1.0889485107
H	-7.0452923011	-4.2065356895	-0.8518405155
H	-7.7485579638	-2.8207283975	0.0264367937
H	-6.9440968581	-4.1077648721	0.9328940290
C	-3.3293399996	1.0614951944	0.3728158220
C	-3.6967682931	2.4519911362	-0.1555631521
C	-4.9927717377	2.9408637391	0.4778731463
C	-6.0939583647	1.8796362698	0.3769830595
O	-5.6422373321	0.6761595140	0.9641947139
C	-4.5203008453	0.1037351566	0.2959370300
H	-2.9980870752	1.1489685909	1.4161236982
H	-3.8331227989	2.3762286001	-1.2431375473
O	-2.6852449284	3.4019526374	0.1420883256
H	-4.8128445124	3.1075354316	1.5471862195
O	-6.4504525625	1.7122739582	-0.9660724312
H	-4.7662603109	-0.0775986244	-0.7573609850
C	-4.2180699888	-1.2118484442	0.9850079550
H	-6.0435787738	4.5810700252	0.3924377588
H	-4.0076379318	-1.0515385922	2.0437991637

H	-3.3717862237	-1.7104357086	0.5111929854
O	-5.3772873961	-2.0654888764	0.9716265736
C	-7.5233557041	0.7949102962	-1.1582599140
H	-6.9692156048	2.1727817970	0.9693431838
H	-8.3886867491	1.0801941623	-0.5475905561
H	-7.2233507154	-0.2282429697	-0.9051550431
H	-7.7896026748	0.8416057408	-2.2145250118
H	-1.8196453398	3.0087931752	-0.0740312282
C	2.3718643632	-2.9157622408	1.0909280421
H	1.3018502324	-3.0380822075	1.2759752465
H	2.8835304658	-3.8616193607	1.2628875210
H	2.7551868562	-2.1634683008	1.7850316169
O	3.2852051401	-3.1537058437	-1.1122674968
O	-0.7243260417	-1.8654057930	-0.2784792244
H	-0.8759334148	-1.9823103050	1.3864934254
H	-1.7321041562	-3.4004613032	-0.0279481305
H	-1.7925185571	-1.9991146499	-1.6135820937
O	-0.9641976132	-2.0418785074	2.3802690196
O	-2.2920894166	-4.1923804595	0.0691738552
O	-2.4246244400	-2.0542568177	-2.3772805033
H	-1.6947970347	-1.4511553964	2.5931601059
H	-3.1285232206	-3.9344921944	-0.3402488783
H	-3.2860066048	-2.1729770645	-1.9498550135

D-Glc-RE3

-1 1

C	6.8351502355	-0.5681812005	0.4897733338
C	6.5549447740	-1.6536075324	-0.5513746675
C	5.2742783663	-1.3629269982	-1.3342828076
C	4.1305193182	-1.0423856008	-0.3780659079
O	4.5107005969	-0.0170623809	0.5172525329
C	5.5981216180	-0.3766866932	1.3672854005
H	7.0516281827	0.3801650123	-0.0257032583
O	7.9282681881	-0.9360530753	1.3151872170
H	6.4325169782	-2.6082153611	-0.0274464313
O	7.6671405542	-1.8349750157	-1.4157866081
H	5.4270315006	-0.4947569467	-1.9865966033
O	4.8918198737	-2.4986603566	-2.0995537374
H	3.8583791327	-1.9386613702	0.2018405553
H	5.3648855631	-1.3201125974	1.8837849565
C	5.7455013836	0.7313710893	2.3942857110
H	8.6717686402	-1.1521073032	0.7380714445
H	7.8176590542	-1.0126250849	-1.9011682219
H	5.1522536790	-2.3692853850	-3.0177065829

H	5.8383944318	1.6933112009	1.8722597002
H	6.6598626562	0.5620824669	2.9671467870
O	4.6693607576	0.7541954517	3.3144143224
H	3.8676357342	1.1276483778	2.8831511981
O	3.0559775480	-0.5855878389	-1.1356141919
O	-2.2208369010	-0.4340028435	0.2728454095
C	-1.3225377129	2.8116507907	0.6726422586
C	1.7969318540	-0.5478774633	-0.4658583947
C	1.1649811580	0.8435647216	-0.6581616888
C	-0.2166092402	0.7932339829	0.0106023785
C	-1.0625180142	-0.3686623242	-0.4899979439
O	-0.3519357588	-1.5821171818	-0.3000453857
C	0.8735312692	-1.6327559423	-1.0373123128
H	1.9450160492	-0.7332626826	0.6055331133
H	1.0046057956	0.9732522619	-1.7454245671
H	-0.1001364543	0.7129764410	1.0940040276
H	0.6696884819	-1.4222265743	-2.0975243119
C	1.3860414634	-3.0694118511	-0.9270466384
H	0.5147611906	-3.7291717307	-0.9548242408
H	1.8997206536	-3.2253044086	0.0298405140
O	2.2102850436	-3.4462917679	-2.0172201307
H	3.0819743119	-3.0161376480	-1.9538213798
H	-1.2993808486	-0.2489838172	-1.5578845664
O	-5.0883702379	-4.2313142121	0.7306166778
C	-5.4813164993	3.1617725418	-1.2018725034
C	-6.4605880625	4.1152847049	-0.5961847664
O	-4.8010949992	3.3839948970	-2.1915759419
H	-6.6606190613	4.9303453310	-1.2905810219
H	-6.0278238266	4.5223547286	0.3231526002
H	-7.3869738074	3.6005369851	-0.3333742293
C	-3.3482833901	-1.0915926674	-0.3062652624
C	-3.5360204366	-2.4585332563	0.3555794387
C	-4.9023410497	-3.0292540298	0.0050849213
C	-6.0092889497	-2.0099993308	0.2978620146
O	-5.7668019659	-0.8243608182	-0.4336111717
C	-4.5500803949	-0.1659997237	-0.0888142126
H	-3.1848992883	-1.2382828830	-1.3817494779
H	-3.4685815484	-2.3245923272	1.4435067825
O	-2.5505773327	-3.3816484890	-0.0818885447
H	-4.9321255829	-3.2287723664	-1.0733624264
O	-6.0679131499	-1.7841102198	1.6769916149
H	-4.5740483359	0.1387719264	0.9648515038
C	-4.4348782113	1.0547143696	-0.9706943951
H	-5.8459318265	-4.6988690636	0.3589720517
H	-4.6358927533	0.7934775720	-2.0130972580
H	-3.4361675963	1.4880894606	-0.8893033765

O	-5.4036125200	2.0150303415	-0.5180698365
C	-7.1512007125	-0.9477161202	2.0717169475
H	-6.9757177249	-2.3748322466	-0.0704652604
H	-8.0998816180	-1.3391004177	1.6848877492
H	-7.0088268376	0.0787108652	1.7178092666
H	-7.1708474319	-0.9549797530	3.1619565039
H	-1.6817236006	-2.9439951057	-0.0579410628
O	-0.9310832927	1.9939656631	-0.3122404486
C	-2.1378799618	3.9611519340	0.1688301608
H	-3.1653810267	3.8256308042	0.5213722289
H	-1.7538410299	4.8873184397	0.6013083164
H	-2.1326187893	4.0199775320	-0.9190666985
O	-1.0592531178	2.6249693117	1.8496010673
O	1.9228931109	1.8821286584	-0.1487320012
H	2.1746468429	1.8457653969	1.3912159090
H	3.4245917320	2.1993642376	-0.7790538932
H	1.4507600570	3.3215901526	-0.7752159200
O	2.3698048591	1.8854333138	2.3862959615
O	4.3601065051	2.4108728522	-1.0537427273
O	1.2351195910	4.2172452172	-1.1675777883
H	1.6060556029	1.4783040581	2.8099554531
H	4.8516866649	1.6848006420	-0.6447766859
H	0.3183962564	4.1502566727	-1.4541232602

D-Glc-RE4

-1 1

C	6.5008479092	-0.8190395737	-0.1180661744
C	5.7805727504	-1.9185409027	0.6596310200
C	4.3957462600	-2.1687888279	0.0615449517
C	3.6303633831	-0.8521603906	-0.0407581468
O	4.3730407001	0.0776009304	-0.8027391189
C	5.6170948571	0.4289406918	-0.2093451030
H	6.7093817402	-1.1757761789	-1.1369401342
O	7.7142812798	-0.4381927372	0.5177968718
H	5.6622930786	-1.5947845513	1.6995752387
O	6.5433554181	-3.1151574886	0.7177143279
H	4.4976942785	-2.5908964562	-0.9475791635
O	3.6332016327	-3.0389969475	0.8856480598
H	3.4449782453	-0.4484141832	0.9642758915
H	5.4470354163	0.8294809417	0.8014553441
C	6.2214503134	1.5199229594	-1.0887374618
H	8.3481660975	-1.1593813511	0.4276570984
H	6.6784953129	-3.4384280591	-0.1830724672
H	4.0896268946	-3.8878787231	0.9381331592

H	5.4609073555	2.2791891715	-1.2835670144
H	6.5315078526	1.0861707247	-2.0487651004
O	7.3117351519	2.1714156570	-0.4532996959
H	7.9187478890	1.4792620885	-0.1513196843
O	2.4321344142	-1.0467484801	-0.7301635881
O	-2.7755276034	0.1468110513	0.4564803476
C	2.4783923657	2.4788352036	-0.1095194281
C	1.2294547905	-0.7382790505	-0.0353653303
C	0.8333957564	0.7219207974	-0.1776490859
C	-0.5461646526	1.0257775125	0.4362017469
C	-1.5406176815	0.0214437756	-0.1709958863
O	-1.0824040714	-1.3189032079	0.0599581428
C	0.1321591975	-1.6184650079	-0.6283783477
H	1.3411041538	-0.9857100135	1.0264057110
H	0.8337859607	1.0080835292	-1.2328907881
O	1.8171327074	1.5046819065	0.5234066505
H	-0.4896900260	0.7837396378	1.5148366138
H	0.0073444487	-1.3870348596	-1.6967346240
C	0.4226723036	-3.1079878897	-0.5080773387
H	1.1817597508	-3.3713902752	-1.2517671742
H	-0.4913728183	-3.6572536298	-0.7502821096
O	0.8362458779	-3.5095954029	0.7876544478
H	1.7900312355	-3.3224003134	0.8640879370
H	-1.6415636770	0.1738309291	-1.2557805713
O	-5.9268951167	-3.3476815886	-0.6175493247
C	-3.9414100038	-0.1912569695	-0.3004627117
C	-4.2481321745	-1.6944517566	-0.2387552975
C	-5.6339478006	-1.9741480910	-0.8056528673
C	-6.6914566133	-1.0751298182	-0.1608476490
O	-6.3220259936	0.2789155631	-0.3201363607
C	-5.0856551237	0.6276533576	0.3057496844
H	-3.7923839320	0.1007450700	-1.3479340872
H	-4.2157003239	-2.0102916856	0.8130716539
O	-3.3258205025	-2.4558516814	-1.0017765633
H	-5.6234944274	-1.7326854001	-1.8759298035
O	-6.8352199930	-1.4317085006	1.1845225022
H	-5.1412585802	0.4199524867	1.3824148658
C	-4.9227355939	2.1242032778	0.1357154960
H	-6.6709809145	-3.5828271257	-1.1841215308
H	-4.0073186606	2.4420468764	0.6342649241
H	-5.7718585700	2.6265324628	0.6127650495
O	-4.8110026113	2.5321255544	-1.2234515728
C	-7.8895288242	-0.7411610957	1.8475799461
H	-7.6509286265	-1.1699952416	-0.6835866597
H	-8.8287696118	-0.8460237987	1.2910337331
H	-7.6545176574	0.3218036150	1.9665461012

H	-7.9956082959	-1.1993856881	2.8314189313
H	-2.4381882009	-2.2816215040	-0.6373253607
C	3.4565517066	3.1592611226	0.7964447232
H	3.8698583409	2.4610299749	1.5258099339
H	4.2515513886	3.6156218238	0.2069086408
H	2.9274828465	3.9501569283	1.3385664964
O	2.2839383155	2.7851736881	-1.2752952441
H	-5.5886421884	2.2278934973	-1.7073036043
O	-0.8970323900	2.3452699270	0.2213602843
H	-1.6220861502	2.5771695353	-1.2795178246
H	-2.0061757317	3.6229070613	0.6498182932
H	-0.1701872806	3.1157674931	1.5317459086
O	-2.1151729633	2.7339079675	-2.1298527004
O	-2.4924219320	4.4658233805	0.8013558100
O	0.2090498717	3.5347958556	2.3524597643
H	-3.0499947864	2.6202395848	-1.8862844448
H	-3.1071695566	4.5273443426	0.0619996126
H	1.0253574121	3.0503524925	2.5149132287

D-Glc-RE5a

-1 1

C	6.5095884019	-0.7231612139	0.0604562378
C	5.8283822588	-1.9846112083	0.5912274384
C	4.4798300797	-2.2121678111	-0.0891181413
C	3.6300972933	-0.9525155411	0.0427644698
O	4.3380882618	0.1209242852	-0.5452529840
C	5.5401424837	0.4598034774	0.1395922796
H	6.7898147306	-0.8834861728	-0.9914619845
O	7.6591841290	-0.4073004432	0.8293355465
H	5.6585569507	-1.8600459831	1.6667515039
O	6.6668336651	-3.1259807774	0.4724853858
H	4.6283841798	-2.4225874094	-1.1571295231
O	3.7781426539	-3.2806948169	0.5286916747
H	3.4325867789	-0.7367688299	1.1027864530
H	5.3145325797	0.6706299332	1.1964166531
C	6.0967112811	1.7111648150	-0.5190000430
H	8.2519804199	-1.1684296188	0.8044474152
H	6.8576250196	-3.2750025269	-0.4635196177
H	4.3425103761	-4.0644095790	0.5064194504
H	6.2503968598	1.5133335706	-1.5885287631
H	7.0660252654	1.9403836607	-0.0709043187
O	5.2655907286	2.8433703277	-0.3320935514
H	4.4922588005	2.7615673776	-0.9311643587
O	2.4378231804	-1.1123947171	-0.6579575691

O	-2.7906380298	0.1788778438	0.3202519136
C	-1.4399071817	3.3032163710	0.1722358990
C	1.2249013569	-0.7013587507	-0.0239908736
C	0.8292778790	0.7221313193	-0.4596798877
C	-0.5768138377	1.0157645723	0.0782285137
C	-1.5722012477	-0.0704096474	-0.3003940576
O	-1.0895805384	-1.3150622132	0.1862536319
C	0.1496380565	-1.7267536706	-0.4014986028
H	1.3590073980	-0.7219952229	1.0651977948
H	0.7562449450	0.7040174844	-1.5644293451
H	-0.5390226343	1.0999301418	1.1658289940
H	0.0395635976	-1.7670118116	-1.4950248602
C	0.3869238976	-3.1411576131	0.1330217302
H	-0.5647252160	-3.6773592973	0.0728923446
H	0.6843458403	-3.0918026077	1.1882032934
O	1.3299369660	-3.8773716230	-0.6232065989
H	2.2221134382	-3.6380706960	-0.3070999757
H	-1.6930625591	-0.1256190777	-1.3920254923
O	-6.0330490829	-3.3336568635	-0.3708800864
C	-3.9745410806	-0.2184146875	-0.3742058395
C	-4.3140841838	-1.6939021012	-0.1322745426
C	-5.6899503710	-2.0018535081	-0.7098125810
C	-6.7385998703	-1.0002382940	-0.2137206725
O	-6.3201948621	0.3172649889	-0.5057701781
C	-5.0967195153	0.6905542718	0.1323395331
H	-3.8329255713	-0.0586698444	-1.4508954323
H	-4.3198407844	-1.8786082084	0.9504659214
O	-3.3879960533	-2.5619397077	-0.7669664276
H	-5.6364870804	-1.8904744458	-1.8001148632
O	-6.9452741457	-1.2025697954	1.1558774340
H	-5.1974517830	0.5680874920	1.2191144844
C	-4.8685407450	2.1692097349	-0.1339578219
H	-6.7866944705	-3.6027389840	-0.9092316685
H	-4.0781772064	2.5310845548	0.5297610707
H	-5.7885444928	2.7019761395	0.1201855583
O	-4.5723846536	2.4745957016	-1.4897057194
C	-7.9929529837	-0.4058023265	1.6988950798
H	-7.6801544377	-1.1259415578	-0.7619525587
H	-8.9164882139	-0.5372197447	1.1221386340
H	-7.7204162920	0.6547147105	1.7103066886
H	-8.1500516515	-0.7501235923	2.7215957831
H	-2.5099734002	-2.3919043345	-0.3832643283
O	-1.0309719117	2.2353834586	-0.5263617748
C	-1.4046395660	3.2768888993	1.6698637862
H	-1.7525100990	4.2403058424	2.0387296636
H	-2.0525859950	2.4832616067	2.0529698026

H	-0.3893192886	3.0917940604	2.0308031304
O	-1.8352119474	4.2622470993	-0.4738619200
H	-3.6294786794	2.3349549826	-1.6399033459
O	1.7323950086	1.6811355574	-0.0375390719
H	2.2212662802	1.5564825271	1.5191671279
H	1.4992158676	3.3418818928	0.2135918093
H	2.5487987746	2.1833702703	-1.2721297478
O	2.5133100439	1.5443535951	2.4769582418
O	1.5434739720	4.3231272910	0.3513317642
O	3.0948906069	2.5734124004	-2.0334436806
H	3.4054352547	1.1815352713	2.4602127910
H	2.3010015702	4.6020501938	-0.1741217665
H	3.3647695310	1.8162101531	-2.5649523519

D-Glc-RE5b

-1 1

C	7.0275931281	0.1299537713	0.3719610832
C	6.7078183643	-1.3385184816	0.1002351061
C	5.4227856722	-1.4725537049	-0.7195485925
C	4.3023870993	-0.6652448554	-0.0727108302
O	4.7120242833	0.6791061781	0.0954658291
C	5.8096068052	0.8211091690	0.9905846052
H	7.2669692432	0.6316115454	-0.5764829336
O	8.1171513815	0.2630657238	1.2761501918
H	6.5643021151	-1.8474571981	1.0596936220
O	7.7899146200	-2.0081764975	-0.5289336808
H	5.5870515173	-1.0820991563	-1.7325329551
O	4.9977378199	-2.8247173307	-0.7732221927
H	4.0418721140	-1.0918454629	0.9083108811
H	5.5697207336	0.3504508644	1.9559121504
C	6.0109337103	2.3193764132	1.1975586130
H	8.9249015736	-0.0094241388	0.8247277254
H	7.9487850075	-1.5981818209	-1.3897091644
H	5.5719567455	-3.3164892152	-1.3718008876
H	5.0553948125	2.7692811790	1.4757155673
H	6.3512937662	2.7755148686	0.2584061919
O	6.9235740972	2.5929414741	2.2513912944
H	7.7152366748	2.0577736987	2.0891107307
O	3.2015667232	-0.6780404495	-0.9265567623
O	-2.1445179127	-0.1067760172	0.0671420625
O	-0.8274838552	1.8632654960	-1.4644562295
C	1.9402959129	-0.3848857406	-0.3308887832
C	1.2823188493	0.7627487940	-1.0924490262
C	-0.1400942456	0.9784273829	-0.5726526207

C	-0.9309846424	-0.3235662747	-0.5611210660
O	-0.2137378604	-1.2896206355	0.1957102940
C	1.0449653626	-1.6304795156	-0.3923053446
H	2.0772569298	-0.0944785293	0.7177457868
H	1.2310540390	0.4869409142	-2.1544059289
O	2.0633960661	1.9313397996	-0.9307870541
H	-0.1185846720	1.4003197263	0.4352311914
H	0.8894598692	-1.9213390995	-1.4410934928
C	1.5770583924	-2.8459621213	0.3719905608
H	0.7128190017	-3.4343083883	0.6911750298
H	2.1237967370	-2.5290353244	1.2687649363
O	2.3730939454	-3.6958520253	-0.4341748691
H	3.2552669713	-3.3011965007	-0.5623368278
H	-1.0765996529	-0.6996709396	-1.5840008896
O	-4.8713873366	-4.0404261901	0.6645958963
C	-1.1058927778	3.1211747084	-1.0760988259
C	-1.8366474786	3.8655847786	-2.1461033156
H	-2.6805708675	3.2750294565	-2.5092417563
H	-1.1540106035	4.0300686110	-2.9856321399
H	-2.1802991902	4.8245987701	-1.7623444646
C	-3.2787914363	-0.8402789386	-0.3997206826
C	-3.3981166335	-2.1912253527	0.3114541054
C	-4.7121676041	-2.8498440058	-0.0866080539
C	-5.8933126040	-1.8912939110	0.1068875074
O	-5.6635647958	-0.6875612257	-0.5959036395
C	-4.5079538130	0.0363233021	-0.1489214881
H	-3.1773250703	-1.0078824456	-1.4804329376
H	-3.3858216923	-2.0175407793	1.3960779560
O	-2.3501421026	-3.0785502940	-0.0494881970
H	-4.6671733197	-3.0837658519	-1.1579751217
O	-6.0843009587	-1.6800575356	1.4779899151
H	-4.6034948270	0.2340619664	0.9255861571
C	-4.4711922471	1.3871255704	-0.8893481003
H	-5.5877920060	-4.5549847389	0.2741186662
H	-5.4597130284	1.5022487941	-1.3667674340
H	-3.7355281973	1.3206049491	-1.7098682607
C	-7.2545198086	-0.9262459634	1.7763744283
H	-6.8029674717	-2.3112802904	-0.3398796401
H	-8.1411115020	-1.4044797329	1.3419748808
H	-7.1726237867	0.0989284218	1.4014685210
H	-7.3470160918	-0.9060482280	2.8627554296
H	-1.5034824467	-2.6600321947	0.1856848815
O	-0.7656732988	3.5793993824	-0.0006959991
H	1.7963826058	2.5772142479	-1.5952308763
O	-4.1952671809	2.4721508728	-0.0513561775
H	-4.6995366603	3.7975749018	-0.8191784203

H	-5.4982877281	2.6886798413	0.8754399314
H	-3.2768545272	2.1538016004	1.2788048525
O	-5.0759693348	4.6068092931	-1.2845096706
O	-6.3181320845	2.8395856299	1.4401119559
O	-2.7278902879	1.9177258317	2.0812528671
H	-5.9976349275	4.6413771414	-1.0078195030
H	-6.7756728194	3.5766031197	1.0222465569
H	-2.2457822218	1.1410884330	1.7679870743

D-Glc-RE6

-1 1

C	-6.9950425501	0.3832566537	0.1202863827
C	-6.7358298799	-1.1237874725	0.1193360282
C	-5.3746405705	-1.4351036284	0.7446032768
C	-4.2963651659	-0.5997563801	0.0680872748
O	-4.6329707376	0.7791253341	0.1810876419
C	-5.8216129274	1.1244910198	-0.5268555079
H	-7.0944599320	0.7306804880	1.1587837181
O	-8.1686953272	0.7083792815	-0.6125188328
H	-6.7363943135	-1.4794514236	-0.9168103046
O	-7.7781974990	-1.8359636350	0.7681925957
H	-5.3908846088	-1.1803044442	1.8129677942
O	-5.0179540386	-2.7965511401	0.5673075505
H	-4.2121226521	-0.8648686057	-0.9962270141
H	-5.7275879887	0.8226571192	-1.5802504507
C	-5.9467158148	2.6425115459	-0.4453328301
H	-8.9334933232	0.3693732293	-0.1324563092
H	-7.8186579291	-1.5527801863	1.6915804191
H	-5.6329339858	-3.3438194356	1.0713988693
H	-5.0020941441	3.0904018338	-0.7630405353
H	-6.1415330185	2.9393148179	0.5936284909
O	-6.9557099846	3.1402037435	-1.3118017657
H	-7.7610818660	2.6311875843	-1.1356712025
O	-3.0930847084	-0.7962016629	0.7248758386
O	2.1664828393	-0.4252755239	-0.6402652419
C	-1.8920736824	-0.7029306279	-0.0487550945
C	-1.3068080260	0.7101999740	0.0067346574
C	0.1043492207	0.7756144485	-0.6023376852
C	0.9391489217	-0.3538815488	0.0100793144
O	0.3068137550	-1.6182652170	-0.1777596918
C	-0.9242982985	-1.7376329383	0.5260142939
H	-2.1108628038	-0.9657262811	-1.0920584543
H	-1.2365868985	1.0093643129	1.0637128274
O	-2.1262800927	1.6321195431	-0.7008314639

H	0.0104163195	0.5515237142	-1.6816756170
H	-0.7613053106	-1.5416098727	1.5960660193
C	-1.3803561818	-3.1861604005	0.3432722222
H	-0.5096987592	-3.8277911601	0.5039612434
H	-1.7316471621	-3.3359599575	-0.6856765710
O	-2.3661470804	-3.5885074324	1.2778054258
H	-3.2238173420	-3.1988138557	1.0281910284
H	1.0810386163	-0.1833299424	1.0888814103
O	5.1792917443	-4.1392171481	-0.7848037434
C	5.0747870767	3.2411686827	1.2226584681
C	6.2281583278	4.1404835522	0.9151742126
O	4.1097515627	3.5454036263	1.9100238245
H	6.1164856058	5.0836987405	1.4477217910
H	6.2618872623	4.3255341491	-0.1621835887
H	7.1642217126	3.6547964061	1.2016922522
C	3.2238033043	-1.0726672056	0.0654497054
C	3.5455967411	-2.4103215924	-0.6022320021
C	4.8452032099	-2.9778129887	-0.0458315009
C	5.9695627598	-1.9361530995	-0.0854427258
O	5.5676207286	-0.7751237974	0.6161283963
C	4.4304542877	-0.1297555791	0.0488005873
H	2.9233777307	-1.2575578085	1.1049563968
H	3.6598786288	-2.2356659472	-1.6809437713
O	2.5205856239	-3.3654971296	-0.3727336643
H	4.6865348374	-3.2325516316	1.0091763660
O	6.2932232355	-1.6649559855	-1.4190779146
H	4.6457917277	0.1584770344	-0.9883610172
C	4.1367181807	1.1026113789	0.8668467517
H	5.8386098535	-4.6379746023	-0.2886380457
H	4.0756009534	0.8608936911	1.9317477397
H	3.1964825894	1.5444275050	0.5335380697
O	5.2037774304	2.0420142261	0.6515577562
C	7.4074465394	-0.7900280133	-1.5678223175
H	6.8516623652	-2.2996371933	0.4554177318
H	8.2715902422	-1.1674280130	-1.0078218019
H	7.1655078182	0.2217603597	-1.2262963953
H	7.6451011043	-0.7651048389	-2.6317539560
H	1.6591612217	-2.9120446676	-0.4050919458
H	1.9427449267	2.2788139301	-1.4073734841
O	2.6703770974	2.5298999325	-2.0512201356
H	3.3242295557	1.8275642975	-1.9736438076
H	2.2043900995	2.9306638106	2.0664596484
O	1.2623493842	2.7074378970	2.0972223497
H	1.0397603136	2.4071130935	1.1690887443
H	-0.0496762745	3.2203909543	-1.1838286223
O	-0.5266729363	3.9550295201	-1.6700882678

H	-1.4436698423	3.6581783346	-1.6428678707
O	0.7046607877	2.0077657520	-0.3895565151
H	-3.0357067366	1.5632832360	-0.3603662089

D-Glc-TS1a

-1 1

C	6.8659621734	-0.1986841125	0.0710298956
C	6.3355101507	-1.3357473114	0.9417301437
C	5.0618896678	-1.9227891387	0.3330240300
C	4.0616944228	-0.8083949934	0.0389771350
O	4.6559481970	0.1655755356	-0.7958263405
C	5.7579534608	0.8269347079	-0.1848659950
H	7.1914968619	-0.6059784312	-0.8969280043
O	7.9479389147	0.4759765247	0.6990926876
H	6.0996194241	-0.9366052537	1.9344037660
O	7.3175616771	-2.3386847824	1.1577385927
H	5.3011902519	-2.4304254208	-0.6113827882
O	4.4328712891	-2.8249107358	1.2318771862
H	3.7319768840	-0.3417713775	0.9779235370
H	5.4426491173	1.2714377919	0.7714953545
C	6.1801184710	1.9389587555	-1.1398257557
H	8.7013269836	-0.1252275159	0.7371538939
H	7.5603048382	-2.7230884224	0.3045311295
H	5.0248428619	-3.5720892995	1.3816879304
H	5.2955423361	2.5103415129	-1.4301173839
H	6.6211177130	1.4992427850	-2.0441595226
O	7.0833781676	2.8496371485	-0.5293345034
H	7.8018684733	2.3252337637	-0.1451283803
O	2.9770541694	-1.3296789864	-0.6681321913
O	-2.3900904555	-0.8982351459	0.1446315007
O	-0.9650803024	1.4296195613	-0.7384905836
C	2.2563108518	2.2816247999	-0.7815881047
C	2.9504425058	3.3324112570	0.0234767616
O	2.0746836575	2.3232717188	-1.9868042689
H	3.7196157407	2.8773159684	0.6522178945
H	2.2180163779	3.8100318969	0.6817169890
H	3.3922784681	4.0780184386	-0.6360677650
C	1.6864347045	-1.1170535534	-0.1168068587
C	1.0804221941	0.1991885859	-0.5771440650
C	-0.3628960619	0.3196219587	-0.1025923985
C	-1.1536522564	-0.9307189880	-0.4865086066
O	-0.5011930311	-2.0959260666	-0.0153393694
C	0.7961795107	-2.2722481225	-0.5832513548
H	1.7385450108	-1.1359446668	0.9776865285

H	1.1283572069	0.2651069253	-1.6669025976
O	1.8328148276	1.2764486833	-0.0016372465
H	-0.3882286215	0.4339311458	0.9844747102
H	0.7153130796	-2.2556436454	-1.6805127780
C	1.3334735722	-3.6373018143	-0.1781390148
H	2.1696380990	-3.8910179452	-0.8373558869
H	0.5415148055	-4.3740695032	-0.3370249640
O	1.7315870022	-3.7163212518	1.1809076276
H	2.6404256117	-3.3693285658	1.2428439711
H	-1.2708258139	-0.9891680122	-1.5780603013
O	-6.3156611144	-3.4582672335	-0.7467442277
C	-1.3151745496	2.5476454959	0.0340734159
C	-1.4507162662	3.7177345568	-0.9151167834
H	-2.1192759593	3.5043632813	-1.7515351908
H	-0.4530387846	3.9326627871	-1.3137503087
H	-1.8036964701	4.5954989999	-0.3741605864
C	-3.5716112503	-0.9349449371	-0.6466477583
C	-4.2811683904	-2.2606608101	-0.3819719797
C	-5.6208502887	-2.2756258551	-1.0999809908
C	-6.4255300473	-1.0134790988	-0.7559446946
O	-5.6682579299	0.1443235365	-1.0467792973
C	-4.4569556319	0.2589239397	-0.2866392174
H	-3.3094591855	-0.8744777004	-1.7099429257
H	-4.4432025563	-2.3641948656	0.6992616319
O	-3.5052614888	-3.3512069319	-0.8587960730
H	-5.4367508418	-2.2532067031	-2.1812962969
O	-6.8001169086	-1.0750495927	0.5915918267
H	-4.6936230416	0.2254444561	0.7833691038
C	-3.8604880377	1.6333996238	-0.6239423591
H	-7.0404485432	-3.5868706222	-1.3694483095
H	-4.7147454328	2.2945246553	-0.8360272557
H	-3.2775268237	1.5505899197	-1.5521927078
C	-7.6872523388	-0.0331560894	0.9843418156
H	-7.3137548244	-0.9386565924	-1.3949900553
H	-8.6050651183	-0.0625174641	0.3841904972
H	-7.2176861917	0.9502010495	0.8789525928
H	-7.9306913868	-0.2080547964	2.0327321361
H	-2.6730811504	-3.3638840077	-0.3673440444
O	-0.8084031703	2.6984523718	1.1709778694
O	-3.0768765870	2.1784582262	0.4100630304
H	-3.7162850091	3.8651183271	0.5036858894
H	-4.4408738338	2.3628761102	1.7978657433
H	-2.7175930436	1.1247115733	1.9132231436
O	-4.1509705286	4.7478471846	0.5372387617
O	-5.2033544102	2.4824211603	2.3944064772
O	-2.6152766376	0.4552063059	2.6228894158

H	-4.9497002028	4.6003380196	1.0557349690
H	-5.5908998456	1.6029494182	2.4687392872
H	-2.4840033690	-0.3480971013	2.1000732199

D-Glc-TS1b

-1 1

C	-6.5909088651	-1.0389033849	-0.1447493367
C	-5.7805600224	-2.1405213636	-0.8177421909
C	-4.4117336994	-2.2872234631	-0.1511541337
C	-3.7007130447	-0.9351460366	-0.0595721087
O	-4.5499533115	0.0033798215	0.5668576100
C	-5.7637322657	0.2471453096	-0.1328325243
H	-6.8144804087	-1.3256418114	0.8926579935
O	-7.7987798292	-0.7784528780	-0.8465781097
H	-5.6321987807	-1.8702421740	-1.8690781544
O	-6.4724093813	-3.3808067297	-0.8403803060
H	-4.5376999299	-2.6747933704	0.8691792894
O	-3.5923605260	-3.1615022735	-0.9141386391
H	-3.4223643221	-0.5819279694	-1.0632256402
H	-5.5470205665	0.5423567389	-1.1707122569
C	-6.4476188495	1.4061967447	0.5847382129
H	-8.3751324715	-1.5464436926	-0.7556784923
H	-6.6320709010	-3.6631972809	0.0701176845
H	-4.0522764990	-4.0069857049	-0.9971917144
H	-5.7266159949	2.2173882402	0.7090801088
H	-6.7790480601	1.0792298467	1.5791814366
O	-7.5362658321	1.9272370552	-0.1645862870
H	-8.1084258810	1.1802633420	-0.3952089030
O	-2.5777547359	-1.0193037671	0.7735140074
O	2.6952231846	-0.5849740646	-0.4186940189
C	-2.5114452880	2.2016940667	0.0705389330
C	-3.3676903938	2.8650569307	-0.9593973688
O	-2.6360379392	2.3444512949	1.2786383011
H	-4.0457749092	2.1201279405	-1.3861832415
H	-2.7510318215	3.2564123425	-1.7700732157
H	-3.9484552006	3.6648991699	-0.5020172114
C	-1.2890357477	-0.8776009225	0.1848084896
C	-0.7607980050	0.5535701314	0.3139165732
C	0.6874775079	0.6949981144	-0.1950499398
C	1.5421630727	-0.4706935351	0.3507949237
O	0.9198033260	-1.7412676592	0.2432691833
C	-0.3294891634	-1.8197727845	0.9146243690
H	-1.3272011188	-1.1642572553	-0.8722074681
H	-0.8227430849	0.8683432461	1.3585856464

O	-1.5975538471	1.4052984009	-0.4936318481
H	0.6579051738	0.5839495140	-1.2934340394
H	-0.2143714555	-1.4907460851	1.9593223309
C	-0.7563523337	-3.2771623887	0.9396910500
H	-1.6531329988	-3.3738562063	1.5610664137
H	0.0468556919	-3.8517436874	1.4095905548
O	-0.9751683457	-3.8218859834	-0.3501868646
H	-1.8795070179	-3.5700716596	-0.6175444659
H	1.7840115228	-0.2884719776	1.4069203741
O	6.4955340988	-3.2464914500	0.7989168423
C	2.2474841301	2.7774891431	-1.2176186688
C	2.6120128180	4.0168610615	-0.4247587710
H	2.9326889583	3.8078160475	0.5951772820
H	1.7596320851	4.6926068058	-0.3962828250
H	3.4336761908	4.5136272417	-0.9521979488
C	3.9513739084	-0.5931281482	0.2318221324
C	4.5162827531	-2.0088390113	0.2713348930
C	5.9211766345	-1.9534472341	0.8523318347
C	6.7786908825	-0.9195473023	0.1006642435
O	6.1507672273	0.3500768770	0.1048790541
C	4.8717096672	0.3555175960	-0.5292206385
H	3.8459894494	-0.2265822825	1.2605258388
H	4.5538929175	-2.4090559615	-0.7505783976
O	3.7133307430	-2.8497309638	1.0887151700
H	5.8532289275	-1.6126451366	1.8931452771
O	7.0103637325	-1.3878028469	-1.1970610340
H	4.9693535453	0.0277771398	-1.5718390213
C	4.3656199974	1.7901150559	-0.5001990425
H	7.2824023183	-3.2563585950	1.3565805043
H	5.1763229982	2.4674661721	-0.7898105084
H	4.0360033323	2.0394985826	0.5099997456
O	3.2981242201	1.9000617688	-1.4390183352
C	7.9180505735	-0.5808658563	-1.9411504811
H	7.7296762161	-0.7619283550	0.6238311298
H	8.8621010207	-0.4606590630	-1.3959303671
H	7.4906903151	0.4049316998	-2.1512261217
H	8.1028500020	-1.1023227001	-2.8808023295
H	2.8051564731	-2.8204694284	0.7494340046
O	1.4542402019	2.8443539596	-2.1871557992
O	1.2569296330	1.9177485172	0.1621364915
H	-0.0778335191	3.5350765725	-0.2979562787
H	0.3398497040	2.8667456518	2.0463504772
H	2.1343764369	1.9457408269	1.6590426280
O	-0.5908609214	4.3540681748	-0.1771568946
O	-0.3162210672	3.3343670407	2.5863209957
O	2.5527025420	2.0783174592	2.5473820387

H	-0.6391695280	4.4299519685	0.7864527786
H	-1.1592347760	2.9397342604	2.3074700648
H	2.8693015265	1.2075885704	2.8104050301

D-Glc-TS2a

-1 1

C	7.1342043984	-0.2734314152	-0.1444778102
C	6.8644919695	1.1917582848	-0.5052032357
C	5.5638165389	1.6935490079	0.1273676160
C	4.4325893804	0.7322237701	-0.2125726489
O	4.7851687085	-0.5614918666	0.2284822513
C	5.8985064297	-1.1127829865	-0.4678593805
H	7.3397093983	-0.3438517457	0.9340421635
O	8.2288289656	-0.7874607370	-0.8852103029
H	6.7735844705	1.2684723354	-1.5944582522
O	7.9635212620	2.0214134488	-0.1573886630
H	5.6716182923	1.7291229622	1.2197544970
O	5.1987585303	2.9697730532	-0.3737454471
H	4.2522476944	0.7197685447	-1.2983217749
H	5.7061819226	-1.0814513175	-1.5509451126
C	5.9878070015	-2.5555974603	-0.0088450720
H	8.9972423127	-0.2305423139	-0.7085153819
H	8.0800086609	1.9945322654	0.8018212283
H	5.8450311614	3.6182569628	-0.0687399906
H	6.1650326263	-2.5806183214	1.0746526401
H	6.8155610756	-3.0587391967	-0.5110461122
O	4.8031470109	-3.2680461483	-0.3398568100
H	4.0453495522	-2.7513638904	-0.0267900169
O	3.2890605585	1.1212836941	0.4725681363
O	-2.1153642425	0.4844230876	-0.2515354755
C	1.1905637247	-2.7828459790	0.2429265402
C	2.0376643937	0.8230694590	-0.1380393187
C	1.4027527569	-0.4487824854	0.3853597181
C	-0.0062544613	-0.6033371211	-0.1643625497
C	-0.8428232048	0.5743750981	0.2910981183
O	-0.2219696526	1.7473690812	-0.2517233420
C	1.1021194387	1.9962259186	0.2271462383
H	2.1567771287	0.7720743469	-1.2272245960
H	1.3711685891	-0.3960256823	1.4832658303
O	2.0178764189	-1.6561310067	-0.0304811798
H	0.0358064844	-0.5453812775	-1.2648431420
H	1.0803010658	2.1026264632	1.3213902428
C	1.5225455039	3.3278481436	-0.4002235343
H	0.6666917101	4.0048982266	-0.3358418310

H	1.7617202578	3.1766351996	-1.4604891516
O	2.5944723233	3.9596949341	0.2760123156
H	3.4265624822	3.4940901944	0.0748990185
H	-0.8827561844	0.6663829932	1.3854043935
O	-4.9698195061	4.3429801252	0.0281895881
C	-5.5242910250	-2.5370981866	-0.7129630804
C	-6.8068129557	-3.2897466337	-0.8630569186
O	-4.6763248262	-2.4222779494	-1.5853852244
H	-6.7903056541	-3.8718860968	-1.7833359711
H	-7.6321165563	-2.5718546963	-0.8999504289
H	-6.9670936565	-3.9422339840	-0.0022046590
C	-3.1864449490	1.0880435299	0.4699054205
C	-3.4151063584	2.5325005161	0.0103265425
C	-4.7248803779	3.0624111331	0.5807694969
C	-5.8830310912	2.0936342696	0.3109211646
O	-5.5607696982	0.8157287975	0.8237578088
C	-4.4217811409	0.2274562555	0.2004265456
H	-2.9599749654	1.0793732782	1.5450222534
H	-3.4680588760	2.5378360781	-1.0868734966
O	-2.3755212192	3.3917807882	0.4507130044
H	-4.6220503096	3.1338665363	1.6709015690
O	-6.1443824673	2.0625155220	-1.0633411696
H	-4.5823149065	0.1645786734	-0.8823284069
C	-4.2577619987	-1.1646557120	0.7731022715
H	-5.6578013386	4.7730527449	0.5493858369
H	-4.2018654899	-1.1284988593	1.8618766383
H	-3.3605119322	-1.6366742323	0.3713062290
O	-5.4116228705	-1.9770844147	0.4956179815
C	-7.2600567014	1.2478506071	-1.4119634394
H	-6.7799337724	2.4016379589	0.8619434962
H	-8.1447473968	1.5362495978	-0.8313770811
H	-7.0452720530	0.1865961450	-1.2437708835
H	-7.4491858843	1.4143250753	-2.4728274537
H	-1.5283129885	3.0359555572	0.1259411267
C	1.2502202083	-3.2266399027	1.6886879149
H	0.4081049071	-3.8878217567	1.9027037688
H	2.1798473442	-3.7899475969	1.8215889513
H	1.2410071457	-2.3925735904	2.3916706010
O	1.1686570801	-3.6421483574	-0.6688673442
O	-0.4009442554	-1.8703751795	0.2574526505
H	-1.0282511317	-1.6855882538	1.9606598368
H	-1.6718998736	-3.3500088864	0.5813385757
H	-1.3294752908	-2.3372914234	-1.2590993929
O	-1.3962024574	-1.5541974318	2.8616614426
O	-2.2707466984	-4.0751360992	0.8238738264
O	-1.8395803359	-2.4931032556	-2.0796976136

H	-2.0371112979	-0.8416553333	2.7586635286
H	-2.8091739058	-3.7065526883	1.5334254378
H	-2.7682319266	-2.4108401932	-1.8119497601

D-Glc-TS2b

-1 1

C	6.7382493766	-0.5412511242	0.3454409856
C	6.3208924397	-1.4250754953	-0.8321500027
C	5.0294746120	-0.9175552425	-1.4715692950
C	3.9647782294	-0.7419838910	-0.3989339855
O	4.4473414944	0.1145744054	0.6176363208
C	5.5690525449	-0.4197396768	1.3242783564
H	6.9938643437	0.4617652234	-0.0277456176
O	7.8419343110	-1.1050513722	1.0333480741
H	6.1521289500	-2.4412450823	-0.4582222280
O	7.3580604589	-1.5446916371	-1.7945999212
H	5.2091067248	0.0588459448	-1.9431203742
O	4.5180192151	-1.8384063164	-2.4200298280
H	3.7157238717	-1.7136095062	0.0529239345
H	5.3101277272	-1.4155153735	1.7129006510
C	5.8448132306	0.5187003437	2.4843053974
H	8.5536053348	-1.2352296903	0.3941199471
H	7.5429801749	-0.6698735925	-2.1626206829
H	5.2262446629	-2.0975299115	-3.0224916521
H	6.0250810889	1.5301814182	2.0961049505
H	6.7448646090	0.1851437591	3.0043592843
O	4.7907610273	0.5180358700	3.4297952418
H	3.9769337849	0.8783521904	3.0196808719
O	2.8505700618	-0.1512842993	-0.9868451934
O	-2.4537770590	-0.3700118840	0.4985216741
C	-0.0489644924	3.2009716768	0.6222266693
C	1.6272395547	-0.2559402481	-0.2603148098
C	0.8555681990	1.0511190226	-0.3857915330
C	-0.4595027750	0.9262426504	0.3696025912
C	-1.2973004045	-0.1703679845	-0.2422554024
O	-0.5186378437	-1.3651821707	-0.1492806458
C	0.7166729429	-1.3450803438	-0.8766499086
H	1.8337225138	-0.4918668328	0.7898255442
H	0.6214328730	1.1799493889	-1.4576671856
H	-0.2788142765	0.6782935262	1.4251047619
H	0.5135710339	-1.0826119614	-1.9247508199
C	1.2548542487	-2.7791352866	-0.8462788656
H	0.3923115687	-3.4490720362	-0.8179976053
H	1.8483412871	-2.9570390690	0.0586499469

O	1.9834343296	-3.1234027199	-2.0135535899
H	2.8366122482	-2.6583815675	-2.0441223716
H	-1.5309415561	0.0343200349	-1.2972769771
O	-5.0310596160	-4.3882397422	0.8048543987
C	-6.0342952390	2.9065723984	-1.1308759325
C	-7.2731716879	3.6290358155	-0.7062575806
O	-5.2386954675	3.2997104877	-1.9700988167
H	-7.3550981463	4.5717504249	-1.2453644515
H	-7.2405452983	3.8161281890	0.3702207812
H	-8.1476942377	3.0058086943	-0.9115459998
C	-3.4983875673	-1.1122519014	-0.1355826728
C	-3.6134703222	-2.4899920804	0.5215381095
C	-4.8959697501	-3.1802446526	0.0776460867
C	-6.1096364193	-2.2634855568	0.2650899655
O	-5.9041323779	-1.0574155219	-0.4444856218
C	-4.7876786237	-0.2995078646	0.0160442147
H	-3.2707130543	-1.2438863308	-1.2012321447
H	-3.6372230849	-2.3476691376	1.6105828579
O	-2.5249967375	-3.3275781906	0.1654597391
H	-4.8221143352	-3.3910080786	-0.9964545675
O	-6.3164172497	-2.0476834353	1.6314096531
H	-4.9234607939	-0.0363555785	1.0726185712
C	-4.7261861995	0.9599065973	-0.8149713414
H	-5.6951689352	-4.9352788987	0.3689315552
H	-4.7431150610	0.7208390724	-1.8818452656
H	-3.8218201183	1.5252260770	-0.5798018658
O	-5.8805773253	1.7483542648	-0.4808540585
C	-7.5076594370	-1.3204285269	1.9169153782
H	-7.0020743187	-2.7094733360	-0.1900034673
H	-8.3765829126	-1.8134043842	1.4644414853
H	-7.4411168726	-0.2910045852	1.5495829262
H	-7.6176242324	-1.3113146138	3.0016854877
H	-1.7088633338	-2.7970870113	0.1672229739
O	-1.0190943218	2.2167647067	0.2475375132
C	-0.0430830948	4.3248616410	-0.3854958374
H	-0.9304567049	4.9420397747	-0.2088552538
H	0.8442658138	4.9440333634	-0.2409194548
H	-0.0732059058	3.9530274197	-1.4102918491
O	0.0788512732	3.4407060588	1.8455725456
O	1.4189227496	2.2253528320	0.1172343532
H	2.0293552668	1.7785255460	1.8033929419
H	3.1922701110	2.7648728781	0.1927853894
H	2.0436192137	2.8949541897	-1.5563007156
O	2.3052829917	1.4666421959	2.6930005970
O	4.1577729270	2.9019449826	0.2256158179
O	2.3346472935	3.1195766847	-2.4618332597

H	1.7010348729	0.7405292085	2.8862811723
H	4.4858003566	1.9910390063	0.3059898745
H	1.5174462456	3.1785487787	-2.9684979399

D-Glc-TS3a

-1 1

C	6.7184518171	-0.5317878289	-0.1096611786
C	6.0956553997	-1.7475174496	0.5745946386
C	4.7255194381	-2.0580677685	-0.0296533323
C	3.8599587113	-0.8010841881	-0.0428288854
O	4.5296673746	0.2401061991	-0.7263198096
C	5.7355693602	0.6429570581	-0.0861779870
H	6.9388481264	-0.7782419054	-1.1582254606
O	7.9098531291	-0.1199412272	0.5491750048
H	5.9664455913	-1.5217171957	1.6389512826
O	6.9532633105	-2.8786213072	0.5267460222
H	4.8459879757	-2.4035629121	-1.0652512869
O	4.0353209287	-3.0337233365	0.7386661574
H	3.6397743217	-0.4856268020	0.9872659549
H	5.5280954953	0.9265491101	0.9566486192
C	6.2396752857	1.8649026056	-0.8482329213
H	8.5924489819	-0.7827772685	0.3906443558
H	7.1180304509	-3.1026079110	-0.3990147209
H	4.4986421198	-3.8760029021	0.6569423509
H	5.4270600235	2.5908786078	-0.9236591233
H	6.5369779948	1.5669175208	-1.8625140252
O	7.3127927645	2.5086273657	-0.1760917577
H	7.9643401700	1.8245986556	0.0407087251
O	2.6834503780	-1.0542068761	-0.7471327764
O	-2.6002752453	-0.0701498759	0.4103744497
C	1.0257748061	2.9107789812	0.1551938340
C	1.4668025593	-0.7630584985	-0.0726252227
C	0.9559921517	0.6388604067	-0.3049674373
C	-0.3900507128	0.8185102167	0.3808473935
C	-1.3766995848	-0.1569095077	-0.2342817980
O	-0.8527281532	-1.4730741353	0.0021089156
C	0.4027502531	-1.7218130558	-0.6401766790
H	1.5836959346	-0.9518999119	1.0002036316
H	0.8567523561	0.8089750724	-1.3869298766
O	1.7289851801	1.6749692019	0.2736044733
H	-0.2869265815	0.5335078567	1.4420702971
H	0.2909491281	-1.5346605574	-1.7182768377
C	0.7652866817	-3.1891321639	-0.4595267001
H	1.5190762627	-3.4531913078	-1.2080181067

H	-0.1294231955	-3.7869320933	-0.6547977981
O	1.2276151606	-3.5128729053	0.8419020473
H	2.1792503603	-3.3048816622	0.8735756137
H	-1.4891088903	-0.0065345195	-1.3166296352
O	-5.8802908646	-3.4895824415	-0.4142296234
C	-3.7632654642	-0.4156045179	-0.3402498232
C	-4.1310305956	-1.8877097110	-0.1346983504
C	-5.5112210137	-2.1561301943	-0.7179765001
C	-6.5379646860	-1.1485931656	-0.1867326900
O	-6.1102064221	0.1689946184	-0.4663816638
C	-4.8694486221	0.5227282747	0.1464672914
H	-3.5785319487	-0.2399586908	-1.4073834046
H	-4.1437943835	-2.0957860432	0.9437586091
O	-3.2123477890	-2.7536192918	-0.7841587594
H	-5.4615459424	-2.0150847194	-1.8050515902
O	-6.7205316551	-1.3671296900	1.1831642864
H	-4.9541088890	0.4413760710	1.2380814906
C	-4.6080258687	1.9794593670	-0.1885855719
H	-6.6521410275	-3.7222877132	-0.9439732982
H	-3.6735874283	2.2906573219	0.2816332559
H	-5.4242268756	2.5877396107	0.2169057537
O	-4.4602690417	2.2179467122	-1.5832926048
C	-7.7521061293	-0.5698347757	1.7563667197
H	-7.4907979316	-1.2546149952	-0.7194527835
H	-8.6872495500	-0.6874695298	1.1955739237
H	-7.4710891488	0.4882399774	1.7760280216
H	-7.8920426907	-0.9262511081	2.7773777544
H	-2.3145829346	-2.5339040765	-0.4742699904
C	1.1011808693	3.6716965114	1.4562262588
H	2.1169395194	4.0676323052	1.5627399177
H	0.4028269683	4.5102717387	1.4324480073
H	0.8798903690	3.0310469983	2.3114108495
O	1.0840550089	3.5062776087	-0.9468081667
H	-5.2480167290	1.9019570051	-2.0427134087
O	-0.6624922210	2.1780384676	0.2566710708
H	-1.2863345466	2.3532895133	-1.5179532336
H	-1.7214931771	3.8178971221	0.0896891555
H	-1.4907527847	2.4712033592	1.8058291546
O	-1.7423039341	2.3898517915	-2.3821151384
O	-2.2153345514	4.6541447062	0.1025213477
O	-1.9470515335	2.5577228211	2.6731055000
H	-2.6833887950	2.2777583445	-2.1582920647
H	-2.9331906474	4.4947094390	0.7254926484
H	-1.2765265317	2.3200241939	3.3224382386

D-Glc-TS3b

-1 1

C	6.3041626977	-0.8896394031	-0.4594281725
C	5.6224849020	-1.9059386032	0.4566659113
C	4.1926338887	-2.1570854104	-0.0048126560
C	3.4397914169	-0.8334420252	-0.0937973235
O	4.1280010595	0.0167324321	-0.9905691657
C	5.4473634566	0.3763626875	-0.5653110895
H	6.4183277027	-1.3309933985	-1.4610284757
O	7.5715115933	-0.5189414235	0.0579328251
H	5.6037098281	-1.5030412281	1.4755353287
O	6.3657966601	-3.1136487108	0.5403893778
H	4.2008908195	-2.6092958129	-1.0064247031
O	3.4828117675	-2.9921107472	0.8984516851
H	3.3653497993	-0.3574482184	0.8941964823
H	5.3940439887	0.8560931027	0.4222445569
C	6.0017031215	1.3601352424	-1.5837647999
H	8.0820932536	-1.3271202726	0.1920042186
H	6.4206073042	-3.5053461925	-0.3419186252
H	3.9894720141	-3.8062210810	1.0173233982
H	5.8208690188	0.9599331796	-2.5907052841
H	7.0803621102	1.4426636309	-1.4381304001
O	5.4770154285	2.6709257180	-1.4544562009
H	4.5112603369	2.6470684771	-1.5995887196
O	2.1790353924	-1.0510679668	-0.6489804091
O	-3.0324328651	0.2367219336	0.4519984225
C	0.1809403164	3.0995464881	0.6904330796
C	1.0348831675	-0.7416258234	0.1476367197
C	0.6117890655	0.7075962653	-0.0417182282
C	-0.7614167099	0.9589886885	0.5589214061
C	-1.7760813556	0.0685616250	-0.1111549051
O	-1.3560267504	-1.2660735917	0.1781812627
C	-0.0925915963	-1.6654144194	-0.3623556192
H	1.2358309529	-0.9666156483	1.2010044086
H	0.5316342904	0.8614503269	-1.1328156133
H	-0.7646967999	0.7351945414	1.6343049844
H	-0.1268058435	-1.6040324332	-1.4591109245
C	0.0443398994	-3.1247107990	0.0818320817
H	-0.8559276569	-3.6541552551	-0.2413467804
H	0.0867796775	-3.1567899300	1.1782066492
O	1.1508582862	-3.8003020828	-0.4848225899
H	1.9590639211	-3.5114037726	-0.0210711536
H	-1.8054239878	0.2226936345	-1.1997095654
O	-6.5968511960	-2.9548856689	-0.0308974635
C	-4.1497749653	-0.1595373797	-0.3457815119

C	-4.7130653808	-1.4959966191	0.1397806328
C	-6.0281467776	-1.7774236948	-0.5758175134
C	-6.9855975534	-0.5835895160	-0.4744185824
O	-6.3590886438	0.5828094821	-0.9691214377
C	-5.1874569136	0.9594013820	-0.2433395546
H	-3.8345379363	-0.2740034291	-1.3905328293
H	-4.8910474010	-1.4281310843	1.2218810391
O	-3.8319034836	-2.5726197693	-0.1404066115
H	-5.8131151292	-1.9202491439	-1.6422101148
O	-7.3967943982	-0.4439247030	0.8565823914
H	-5.4425643360	1.1163637804	0.8132490669
C	-4.6997644343	2.2908882740	-0.7907598117
H	-7.2942009149	-3.2610683996	-0.6229075037
H	-3.9231194660	2.6835869501	-0.1279502379
H	-5.5398335278	2.9899960055	-0.7843498563
O	-4.2274433490	2.2254492848	-2.1310941486
C	-8.3906573887	0.5587145140	1.0419375530
H	-7.8555793806	-0.7337488606	-1.1253435612
H	-9.2328833996	0.3987043744	0.3578334813
H	-7.9775635957	1.5601874292	0.8819305341
H	-8.7371495560	0.4716718919	2.0722927552
H	-2.9251104271	-2.3003744217	0.0869097176
O	-0.9626528127	2.3371576571	0.3234439685
C	0.2714398045	3.3551653923	2.1780381396
H	1.2867824972	3.6594656299	2.4379919276
H	-0.4119113119	4.1785641275	2.4106681715
H	-0.0099624879	2.4873286281	2.7754381439
O	0.4902480182	4.0011800019	-0.1198428864
H	-3.3121545280	1.9213131173	-2.1213256746
O	1.4006683732	1.7042198008	0.5346678464
H	1.8072991134	1.0723734868	2.2500341862
H	3.0207661812	2.4390691750	1.0169096366
H	2.2225792864	2.3736328142	-1.0646556082
O	2.0313531065	0.7458550016	3.1472721164
O	3.7905828773	2.9027729182	1.3955863467
O	2.7056953595	2.3018006496	-1.9123559274
H	2.3993724007	-0.1331697162	3.0057448912
H	4.2264836932	2.2361286735	1.9379427187
H	2.9905584114	1.3734282402	-1.8967328235

D-Glc-TS4a

-1 1

C	6.9139586066	0.4887018194	0.2362152621
C	6.7396404851	-1.0253725202	0.1460074463

C	5.4644805099	-1.3803400775	-0.6218211837
C	4.2760121651	-0.6184482930	-0.0433154570
O	4.5501877523	0.7705693718	-0.0372448023
C	5.6422829994	1.1259199029	0.8024003179
H	7.0876269535	0.8952867884	-0.7703181315
O	7.9980954856	0.8349135795	1.0892900318
H	6.6592360219	-1.4285326906	1.1615491831
O	7.8774849342	-1.6540308034	-0.4245164754
H	5.5744014261	-1.0962046324	-1.6762871959
O	5.1812005773	-2.7661826117	-0.5115032075
H	4.0728601397	-0.9549974466	0.9855018190
H	5.4679245755	0.7536782821	1.8231923903
C	5.6937145635	2.6506452771	0.8246273308
H	8.8222525622	0.5928922519	0.6503977384
H	7.9879703848	-1.3322131954	-1.3291781231
H	5.7494014773	-3.2542143932	-1.1182982896
H	4.7036784660	3.0337895347	1.0813367935
H	5.9633892565	3.0219212285	-0.1730281904
O	6.5975591592	3.1385554946	1.8062120391
H	7.4350101467	2.6669954855	1.6797626391
O	3.1711605643	-0.8355211079	-0.8623486512
O	-2.1609384398	-0.6670312147	0.2875936221
O	-1.0621664295	1.3266595732	-1.4840814636
C	1.8934046751	-0.5984397459	-0.2724382919
C	1.1202926123	0.4139532320	-1.1093999758
C	-0.2985897757	0.5696187363	-0.5544037909
C	-0.9698305877	-0.7967861168	-0.4131708876
O	-0.1645177038	-1.6623458546	0.3636371005
C	1.1108791143	-1.9183247545	-0.2209099665
H	2.0180042163	-0.2139614454	0.7472354332
H	1.0503368746	0.0285534314	-2.1366081247
O	1.8136450898	1.6483570723	-1.1080077600
H	-0.2655495256	1.0410944444	0.4306304009
H	0.9748349748	-2.2997661152	-1.2438150885
C	1.7669814958	-3.0179017721	0.6180076916
H	0.9674802420	-3.6646060391	0.9882984104
H	2.2855784841	-2.5851203319	1.4827331463
O	2.6411099251	-3.8385306891	-0.1366511987
H	3.4825952490	-3.3701478652	-0.2896293457
H	-1.1393232696	-1.2389563035	-1.4055229273
O	-5.7612507038	-3.8098531395	0.3758657181
C	-1.5933280063	2.5955316424	-1.2299737695
C	-0.9014771684	3.4558426301	-0.1959892902
H	-0.7212004496	2.9580914531	0.7558565119
H	-1.5227820355	4.3346842622	-0.0188978810
H	0.0560916670	3.7861084555	-0.6092159029

C	-3.3804574823	-1.0285894968	-0.3464986639
C	-3.8794745573	-2.3388251834	0.2608037225
C	-5.2649889866	-2.6557301479	-0.2781853032
C	-6.1958398028	-1.4490488258	-0.0925319546
O	-5.6380830715	-0.3033779370	-0.7020761556
C	-4.3815818197	0.1092899222	-0.1486663230
H	-3.2153317608	-1.1714443130	-1.4215374303
H	-3.9281688517	-2.2260644277	1.3517820109
O	-3.0113312291	-3.4135279424	-0.0724864856
H	-5.1881900796	-2.8367679461	-1.3576853218
O	-6.4246333364	-1.2641354331	1.2768132374
H	-4.4992941313	0.3120827777	0.9235716902
C	-4.0149428894	1.4123641208	-0.8656785133
H	-6.5159069898	-4.1452832809	-0.1222853475
H	-4.9593722277	1.9657346963	-0.9903004394
H	-3.6500572388	1.1782132077	-1.8779749575
C	-7.3693516822	-0.2374205326	1.5612714564
H	-7.1458518494	-1.6141510309	-0.6155157115
H	-8.3062218355	-0.4128738345	1.0183445139
H	-6.9746737754	0.7490038669	1.2961739218
H	-7.5579663062	-0.2733221293	2.6347446791
H	-2.1560928357	-3.2523807500	0.3476182906
O	-2.0374710565	3.1215894899	-2.2798992366
H	1.3864845557	2.2334836721	-1.7454337981
O	-3.0796564252	2.1946687588	-0.1717179601
H	-3.7824771323	3.8958279578	-0.6723908296
H	-4.1399127842	2.8581945375	1.1902022513
H	-2.4729887348	1.6389258279	1.4497229759
O	-4.2717432441	4.7231400332	-0.8610397275
O	-4.7573473596	3.1491886032	1.8931425268
O	-2.1675529843	1.3087244337	2.3241707121
H	-5.1875864105	4.5167606621	-0.6451063312
H	-4.9930641487	4.0506106559	1.6485856522
H	-1.9690822738	0.3860701978	2.1195101955

D-Glc-TS4b

-1 1

C	-6.6364866158	0.0627997911	-0.4048664113
C	-6.3477992088	-1.4390919452	-0.4224209265
C	-5.0540398417	-1.7424696526	0.3353685534
C	-3.9275379964	-0.8707882355	-0.2016011706
O	-4.3022558055	0.4982366532	-0.0973197438
C	-5.4203360514	0.8427723783	-0.9120951459
H	-6.8422726007	0.3768103329	0.6285155697

O	-7.7415584345	0.3887413182	-1.2367880291
H	-6.2320666219	-1.7633766791	-1.4624392579
O	-7.4355689337	-2.1907488930	0.0931806683
H	-5.1883980382	-1.5146105301	1.4014403909
O	-4.6535634610	-3.0924798394	0.1664677223
H	-3.7190173434	-1.1098048986	-1.2551693471
H	-5.2155557955	0.5729174078	-1.9582854288
C	-5.5835567120	2.3553899327	-0.8012168768
H	-8.5431544528	0.0375745911	-0.8307871548
H	-7.5822701754	-1.9317936420	1.0127955214
H	-5.3057584689	-3.6616856966	0.5935431310
H	-4.6218338854	2.8294840326	-1.0113021391
H	-5.8845587723	2.6169378883	0.2218220668
O	-6.5135926059	2.8596679062	-1.7481286792
H	-7.3225454932	2.3331621732	-1.6624809499
O	-2.8044849470	-1.0614323076	0.5849138185
O	2.4503853311	-0.4596966687	-0.5503094195
C	-1.5263756396	-0.8389896257	-0.0169881667
C	-1.0125622876	0.5539963301	0.3410669269
C	0.4738931294	0.7403052729	0.0008506830
C	1.3219281021	-0.5165371646	0.2673220352
O	0.6767978146	-1.7219296229	-0.1112401031
C	-0.5847762448	-1.9166840727	0.5138873709
H	-1.6081197054	-0.9463065891	-1.1062070537
H	-1.1576353062	0.6703202207	1.4217886500
O	-1.7396862486	1.5713093682	-0.3453188106
H	0.5321801695	0.9155938490	-1.0812852017
H	-0.4842655899	-1.8261173557	1.6058956529
C	-1.0176070629	-3.3426657158	0.1686722776
H	-0.1448273945	-3.9889420306	0.2950256220
H	-1.3312583302	-3.3911445980	-0.8819161806
O	-2.0308959606	-3.8451182493	1.0218938371
H	-2.8886102673	-3.4692677238	0.7534709704
H	1.5991467542	-0.5710895568	1.3295019344
O	6.3695449651	-3.1736697938	-0.2020004508
C	1.9429678127	3.0313141370	-0.1230456721
C	0.9865480148	3.5692775519	-1.1745743118
H	0.6615373518	2.8072330557	-1.8849394803
H	1.5216241342	4.3450174611	-1.7312304711
H	0.1154726202	4.0190978225	-0.6995432605
C	3.7422407068	-0.5370435461	0.0253627486
C	4.3441650544	-1.9052028999	-0.2880949465
C	5.7892034481	-1.9401973106	0.1813148435
C	6.5634577457	-0.7434964676	-0.3944410912
O	5.9242824739	0.4704474399	-0.0520732264
C	4.5919386837	0.5934205044	-0.5566811068

H	3.6828731372	-0.4176643035	1.1125020534
H	4.3074240063	-2.0685598357	-1.3732924540
O	3.6215040200	-2.9334223204	0.3753649080
H	5.8059563592	-1.8362640788	1.2734577046
O	6.6767516228	-0.9099573742	-1.7793687268
H	4.5995608043	0.5149787795	-1.6516069175
C	4.1316550987	1.9881289381	-0.1517721491
H	7.2050758696	-3.2772218779	0.2683517638
H	4.9032714972	2.7069628640	-0.4503858923
H	4.0140602780	2.0447160030	0.9308899124
O	2.9183610360	2.2971923062	-0.8225500709
C	7.4785846664	0.0831460197	-2.4103184891
H	7.5581163511	-0.6747246986	0.0623978496
H	8.4551868822	0.1617504013	-1.9172893564
H	6.9846686005	1.0601764686	-2.3943583968
H	7.6167127948	-0.2355675918	-3.4439488641
H	2.7031659863	-2.8978442145	0.0701128161
O	2.3306195611	3.7992018262	0.8144778627
H	-2.6925142611	1.3933031826	-0.2387588699
O	1.0291378633	1.8265086754	0.7193472504
H	-0.2063916246	3.2110458117	1.3550199281
H	0.3848640522	1.6009007484	2.4442778549
H	2.4199887566	1.4840056247	2.1313445492
O	-1.0730818495	3.6476987225	1.4561689679
O	0.1113868874	1.4242403919	3.3686538698
O	2.9317847259	1.3545377025	2.9540401475
H	-1.5994635755	3.1248331397	0.8276375722
H	0.9607806295	1.3483223942	3.8234674082
H	2.9851318110	0.3977241877	3.0585549873

D-Man-2a

0 1

C	-6.6748513238	0.0480350259	0.3650542519
C	-6.4616503903	-0.6333733743	-0.9873021724
C	-5.2327174150	-1.5405906541	-0.9407105971
C	-4.0409636439	-0.7249577221	-0.4367093100
O	-4.3196784458	-0.1947886319	0.8480081120
C	-5.3883408554	0.7445768426	0.8253056626
H	-6.9539446869	-0.7052048825	1.1134218429
O	-7.6900848712	1.0434746290	0.2858222210
H	-6.2927682589	0.1419977322	-1.7413677369
O	-7.6167026960	-1.3443979652	-1.3992665142
H	-5.0058809531	-1.9031162332	-1.9499263070
O	-5.5355609056	-2.6257976961	-0.0777863981

H	-3.8378430729	0.0951002139	-1.1400167719
H	-5.1494061017	1.5591419128	0.1242392778
C	-5.5040671948	1.3155292702	2.2359981117
H	-8.5409396070	0.6030898890	0.1755188648
H	-7.6869400233	-2.1342085697	-0.8456908719
H	-4.9114702996	-3.3424001342	-0.2366536123
H	-4.5137253427	1.6176851574	2.5835364943
H	-5.8857045443	0.5410566752	2.9146065258
O	-6.3304481505	2.4708195693	2.2738608380
H	-7.1537033747	2.2448579567	1.8144688796
O	-2.9272246417	-1.5581028980	-0.3111611940
O	2.4660902706	-0.7861173040	-0.3356298905
O	0.5456051341	0.9913116933	-1.1142227728
C	-2.0503629009	2.0159097067	1.1830904083
C	-2.7547199876	3.2769357678	0.8008488256
O	-1.6537083689	1.7457494841	2.3036048470
H	-3.4015685150	3.1256210622	-0.0637805938
H	-1.9921640946	4.0183915302	0.5396942840
H	-3.3269801844	3.6500052802	1.6505825749
C	-1.6560184457	-1.0293807804	-0.6870845723
C	-1.1295160423	-0.0075601865	0.3186365574
C	0.3586504729	0.2854340743	0.1156281608
C	1.1378946671	-1.0210444523	0.0180469609
O	0.6172566279	-1.8283651044	-1.0130379641
C	-0.7188472074	-2.2481912194	-0.7648717684
H	-1.7303525428	-0.5668514166	-1.6791816289
H	-1.2805014288	-0.3837820951	1.3337926058
O	-1.8652267032	1.2071878067	0.1291057439
H	0.7434379768	0.8775051746	0.9476446379
H	-0.7636975309	-2.7936221893	0.1890982827
C	-1.0674790907	-3.1966711206	-1.9117747622
H	-0.2361545550	-3.8931108372	-2.0391470544
H	-1.1854509915	-2.6217381038	-2.8392219057
O	-2.2201136851	-3.9810097201	-1.6519731005
H	-2.9468028917	-3.3739158562	-1.4617683049
H	1.0668678498	-1.5476845168	0.9794425023
O	4.7412887891	-2.2311245842	3.0338084269
C	0.7003997320	2.3249508492	-1.0729692522
C	0.8943786588	2.9035851510	-2.4367562340
H	0.3519324594	2.3304219303	-3.1894833113
H	1.9616014717	2.8633169991	-2.6769315823
H	0.5729588747	3.9453826891	-2.4397121693
C	3.4415724961	-0.8867297119	0.7004480583
C	4.0879791277	-2.2694397038	0.6838924007
C	5.2248951290	-2.3300861379	1.7027349634
C	6.1852008917	-1.1594721384	1.4973037807

O	5.5042231039	0.0794411002	1.4659639393
C	4.5064839625	0.1850859457	0.4448623371
H	2.9709965689	-0.6944793673	1.6708470623
H	4.5061621222	-2.4432238925	-0.3151251451
O	3.1651546753	-3.2994127147	1.0083263312
H	5.7809738966	-3.2647127606	1.5703898584
O	6.8949274410	-1.4052969257	0.3148345151
H	4.9663709782	0.0098739288	-0.5362404707
C	3.9690949864	1.6142584935	0.4752056830
H	4.1182442643	-2.9538207923	3.1782689902
H	4.6413121734	2.2229909867	1.0891043527
H	2.9670364783	1.6503646747	0.9171767400
C	7.9443005669	-0.4717663556	0.0767010996
H	6.8687187099	-1.0771073066	2.3499298378
H	8.6553248042	-0.4715277795	0.9116070873
H	7.5485131871	0.5384119477	-0.0645219974
H	8.4480253932	-0.7946623769	-0.8346279182
H	2.5860296999	-3.4489958005	0.2515172416
O	0.6959301288	2.9640024627	-0.0358242218
O	3.9298888344	2.1311010273	-0.8531093813
H	2.4527030292	4.9379001323	-1.0584317158
H	5.5517093932	2.1661770422	-1.6675365818
H	3.5243386597	1.0179290691	-2.3128461946
O	3.3002190785	4.7944940140	-0.6190632576
O	6.3940401466	2.2331771428	-2.1603970462
O	3.2959611180	0.2543620160	-2.8730840839
H	3.1010244351	4.8774503234	0.3217219724
H	7.0771856538	2.2421204190	-1.4813483018
H	2.9052738022	-0.3482768928	-2.2224022621
H	3.6522680445	3.0723991058	-0.8127702204

D-Man-2b

0 1

C	-6.4518280931	0.2826680018	-0.4150918910
C	-6.0334734117	1.5943579076	0.2503494963
C	-4.6800695219	2.0598429254	-0.2924995272
C	-3.6768775579	0.9148232963	-0.1458903746
O	-4.1337773328	-0.2278992837	-0.8485615001
C	-5.3324563021	-0.7572037666	-0.2953207907
H	-6.6522400775	0.4618254286	-1.4797473837
O	-7.6103786741	-0.2666242714	0.2042113005
H	-5.9383368623	1.4218538715	1.3273636244
O	-7.0231996897	2.5959766567	0.0911127514
H	-4.3260524208	2.9134252539	0.2940826431

O	-4.8510046815	2.4296671318	-1.6539617114
H	-3.5611205871	0.6717409646	0.9199960940
H	-5.1758992836	-0.9935091309	0.7681134164
C	-5.6376215368	-2.0446664769	-1.0547059892
H	-8.3688110270	0.2833119574	-0.0249976872
H	-6.9988878740	2.8792917014	-0.8334778349
H	-4.3508531921	3.2324140735	-1.8310258827
H	-4.7326345174	-2.6541065145	-1.1042513939
H	-5.9457123465	-1.8012457820	-2.0802649187
O	-6.6300394579	-2.8235450741	-0.4011252157
H	-7.3780724760	-2.2346015364	-0.2196077449
O	-2.4495289574	1.2744681359	-0.7080347494
O	2.7207936154	-0.0706619016	0.3880255485
C	-2.1960338833	-2.5167334980	0.3561106557
C	-3.1169170249	-3.1773595491	1.3302903281
O	-1.7311739814	-3.0457504118	-0.6392341581
H	-3.7769350967	-2.4541543606	1.8104952691
H	-2.4987819796	-3.6438096063	2.1050584787
H	-3.6939496908	-3.9538866795	0.8283139420
C	-1.2700168605	0.9489340110	0.0252793970
C	-0.9224920865	-0.5373468484	-0.0305292550
C	0.4618436804	-0.8039625977	0.5623873770
C	1.4646407345	0.0770439964	-0.1776590858
O	1.0983778170	1.4408449217	-0.0295796005
C	-0.1447990586	1.7652435684	-0.6357534273
H	-1.3860550707	1.2692391865	1.0681602364
H	-0.9402168674	-0.8747575005	-1.0701971957
O	-1.8931659758	-1.2662049234	0.7279777460
H	0.7198224350	-1.8547010354	0.4027617519
H	-0.1085255477	1.5214766203	-1.7073459661
C	-0.3189712024	3.2724563535	-0.4638648023
H	0.6236914841	3.7576432366	-0.7268075638
H	-0.5489623399	3.4995639877	0.5854564839
O	-1.3128538429	3.8036163679	-1.3254641693
H	-2.1142823383	3.2798810358	-1.1899653618
H	1.4761562444	-0.1825760410	-1.2472566451
O	5.4399948820	2.7612868840	-1.4438823918
C	2.5752259276	-3.2954232571	-1.3087769794
C	1.8798102558	-4.2376834486	-0.3773426849
H	0.8225225144	-4.2950229093	-0.6399181209
H	2.0008411176	-3.9367054212	0.6636886539
H	2.3217153021	-5.2308252234	-0.5090635277
C	3.8112517174	0.4892029842	-0.3487528356
C	4.4091833947	1.6445802125	0.4478465056
C	5.7077487489	2.1265096266	-0.2041864779
C	6.6436876991	0.9557496045	-0.4929829282

O	5.9908027398	-0.0631684470	-1.2258696827
C	4.8491936703	-0.6138107941	-0.5747761938
H	3.4544138038	0.8520251266	-1.3193889722
H	4.6321301027	1.2838294377	1.4599986209
O	3.5383311354	2.7647819499	0.5097633807
H	6.2169292403	2.8203181202	0.4746906509
O	7.1306035566	0.4959483977	0.7361688170
H	5.1374505979	-1.0488757583	0.3912178561
C	4.3512903614	-1.7189711213	-1.4951924232
H	4.6570724737	3.3134332569	-1.3092525967
H	5.2031989564	-2.2684919851	-1.9028699764
H	3.7518758323	-1.3164763638	-2.3139216517
O	3.5670189657	-2.6302134764	-0.7096697094
C	8.1059259138	-0.5356133210	0.6126264595
H	7.4665540044	1.2754481710	-1.1424447921
H	8.9068836827	-0.2281113013	-0.0700779585
H	7.6549764994	-1.4653963844	0.2512864299
H	8.5177354650	-0.6963987602	1.6092902004
H	2.6179688093	2.4622343101	0.4195762909
O	2.2974512603	-3.1580809604	-2.4902657167
O	0.4387053114	-0.5167759062	1.9499190901
H	3.1927919462	-1.2490327214	3.4483273821
H	0.6370946528	1.2414989751	2.5158149804
H	-1.1525506389	-0.2897401984	2.9676089982
O	2.7887163721	-1.7304939960	2.7167282521
O	0.7725477466	2.1177461620	2.9234600268
O	-1.9954045350	-0.1954142742	3.4455929806
H	3.1438117991	-1.3131983288	1.9151553405
H	1.0250635963	2.6758734023	2.1791279464
H	-2.6435812189	-0.4890555954	2.7938192745
H	1.1976770553	-0.9749134703	2.3721417676

D-Man-2c

0 1

O	4.4302535793	-0.0774115293	0.2077579882
C	5.5648511779	-0.5605181919	0.9243631651
C	5.7892101014	0.3673084235	2.1050890002
O	4.7256993107	0.3072103512	3.0400970368
H	4.0224561628	0.9214922860	2.7415583035
H	6.7022531837	0.0624236687	2.6214314107
H	5.9265459066	1.3934544850	1.7389141590
H	5.3559034539	-1.5754653556	1.2967206846
C	6.7559716485	-0.6051004935	-0.0351116854
O	7.8828476265	-1.1150909801	0.6625012389

H	8.5974877987	-1.2203474717	0.0218332170
H	6.9617117646	0.4165946048	-0.3872409769
C	6.4172649904	-1.4914964472	-1.2345865498
O	7.4793096115	-1.4931824543	-2.1751820025
H	7.4386931580	-0.6470502628	-2.6436956995
H	6.3152234202	-2.5228839694	-0.8802375638
C	5.1033559295	-1.0636648931	-1.9016241531
O	5.2782362703	0.1322260364	-2.6391690664
H	5.1153970343	0.9210285407	-2.0786937125
H	4.8047784530	-1.8373637417	-2.6154605095
C	4.0240707946	-0.9541698218	-0.8273404826
H	3.8386988343	-1.9482221180	-0.3929015533
O	2.8731017388	-0.4295308647	-1.4138718833
C	1.7266309273	-0.3538823757	-0.5741646440
C	0.7237391771	-1.4479407465	-0.9747922852
O	-0.4782454315	-1.2717820904	-0.2209942521
C	1.1971939293	-2.8665057822	-0.7151343970
O	1.5956048339	-3.0921867926	0.6287886733
H	0.8224224495	-2.9917100603	1.1967298369
H	0.3911653328	-3.5525413942	-1.0005524695
H	2.0622384389	-3.0807098690	-1.3462859441
H	0.5000928777	-1.3537653389	-2.0474279518
H	2.0141690279	-0.4758608466	0.4744521399
C	1.1012837069	1.0231520008	-0.7734590163
H	0.9020193041	1.1606982381	-1.8448328341
C	-0.2442639693	1.1209101304	-0.0495760393
O	-0.0415379773	1.0128930867	1.3645561464
C	-0.2957791240	2.0587470456	2.1578331862
C	-0.1808407165	1.7041620338	3.6039923363
H	0.5129798049	0.8776722842	3.7568337989
H	0.1294558523	2.5800811884	4.1735774323
H	-1.1730305559	1.3951675613	3.9498867510
O	-0.6225254430	3.1606840683	1.7410841317
H	-0.7415304781	2.0617718954	-0.2884637773
C	-1.1319503122	-0.0387257323	-0.4763130303
H	-1.3416143563	0.0448964377	-1.5532815384
O	-2.2981780145	-0.0180526598	0.2697658508
C	-3.3972960112	-0.7683169535	-0.2551164231
H	-3.1993460413	-1.0392520402	-1.2990581952
C	-3.6124185144	-2.0272948286	0.5814044642
O	-2.5942821420	-2.9951843904	0.3786796855
H	-1.7482208732	-2.5396802279	0.2170783416
H	-3.6490027323	-1.7285554747	1.6369122834
C	-4.9365799512	-2.7005072798	0.2192594866
O	-4.8834666975	-3.2538456349	-1.0865929608
H	-4.0324642504	-3.7061439519	-1.1660867251

H	-5.1459873016	-3.4878100673	0.9541136233
C	-6.0931059649	-1.6997868588	0.2395443541
O	-6.3756623885	-1.2946444219	1.5589984616
C	-7.4000682965	-2.0608199107	2.1854299727
H	-7.1166474562	-3.1164451265	2.2658808700
H	-7.5318263129	-1.6496935257	3.1867582074
H	-8.3400976417	-1.9762362236	1.6275429013
H	-6.9791302164	-2.1518714666	-0.2184175718
O	-5.8075005566	-0.5684586132	-0.5427953274
C	-4.6270295267	0.1427648096	-0.1759786092
H	-4.7224841967	0.5194745403	0.8502528741
C	-4.4913566601	1.3122334835	-1.1284794402
H	-3.5453678642	1.8249454845	-0.9536662173
H	-4.5467807080	0.9798120603	-2.1664693044
O	-5.5169391472	2.2819813351	-0.8601038546
C	-6.5845821453	2.3499100976	-1.6706583506
O	-6.7151740114	1.6631200093	-2.6699268422
C	-7.5723652015	3.3642840486	-1.1879000452
H	-8.3728939747	3.4810469198	-1.9169699299
H	-7.9893918427	3.0278213815	-0.2341503699
H	-7.0749806560	4.3218632382	-1.0166913020
H	2.5657961332	2.0497745900	1.3789230114
O	2.8957010600	2.2010579399	2.2885806420
H	3.4436607882	2.9917185011	2.2233989804
H	1.6008858746	2.9022354012	-0.4546975938
O	0.7246788233	4.4121132067	-0.4219249902
H	0.1794462054	4.4553919772	-1.2163029784
H	3.7039766509	2.2589391139	-1.0173622203
O	4.5856484173	2.5057546860	-1.3603200620
H	4.4083964303	3.1123565682	-2.0882337391
O	2.0078937817	2.0130789512	-0.3258557423
H	0.1245560045	4.1126967690	0.2842120352

D-Man-2d

0 1

O	4.2050035546	0.1930077094	0.0288842993
C	5.2820421984	0.0804448608	0.9600278417
C	5.6426208003	1.4865744024	1.4030714090
O	4.6616140791	2.0548227554	2.2574002092
H	3.8354770389	2.1742991688	1.7568223325
H	6.5732266663	1.4533300483	1.9726781348
H	5.8006835212	2.1139169818	0.5156347621
H	4.9493910085	-0.4994119206	1.8345797735
C	6.4378872013	-0.6563617184	0.2780889056

O	7.4750083920	-0.8398416802	1.2287731230
H	8.1809634537	-1.3415458192	0.8024022637
H	6.8024195126	-0.0440256189	-0.5604524156
C	5.9493056907	-2.0073497582	-0.2579643976
O	6.9787693344	-2.6677829261	-0.9754287317
H	7.0802216116	-2.2116559770	-1.8226679642
H	5.7134543310	-2.6463451799	0.5996008485
C	4.6853384958	-1.8615878498	-1.1167209931
O	4.9745032136	-1.2612373081	-2.3697400274
H	5.1460433806	-0.3185360220	-2.2408964429
H	4.2851448949	-2.8538187741	-1.3381843132
C	3.6564114741	-1.0644512820	-0.3212991112
H	3.3955143664	-1.6076634977	0.5996207734
O	2.5336725318	-0.8435411916	-1.1152182724
C	1.3837751766	-0.3396579658	-0.4364308091
C	0.3138101987	-1.4477434115	-0.4069022972
O	-0.9412148943	-0.9199812755	0.0154575957
C	0.6397689284	-2.5887031655	0.5404853305
O	0.6795377612	-2.1666131583	1.9027388086
H	-0.1873453101	-2.3322131561	2.2907798214
H	-0.1070287243	-3.3778606962	0.4146227173
H	1.6164334726	-3.0061081817	0.2842729173
H	0.2195207656	-1.8584099004	-1.4229640039
H	1.6527327605	-0.0382034784	0.5746746645
C	0.8461232829	0.8385588921	-1.2454330878
H	0.7466409001	0.4831718824	-2.2762772116
C	-0.5537818593	1.3104157716	-0.8333012882
C	2.4245007909	2.5105541199	-0.3810849055
C	3.4383238802	3.4891488579	-0.8831339921
H	2.9641106368	4.2039630253	-1.5601888802
H	3.8939180229	4.0129055849	-0.0436530716
H	4.2065041468	2.9517827678	-1.4462441327
H	-0.9106831131	2.0048613933	-1.5984906807
C	-1.4682534888	0.0817485577	-0.8505158492
H	-1.5460635162	-0.3237889013	-1.8691256426
O	-2.7073339539	0.4421347604	-0.3572186278
C	-3.8567179591	-0.3005762274	-0.7867983175
H	-3.6920467730	-0.6747155451	-1.8044489888
C	-4.1100238575	-1.4696993768	0.1695936217
O	-3.1941073298	-2.5360379211	-0.0284996720
H	-2.2946562089	-2.1787893207	0.0848492211
H	-4.0269272606	-1.0907521673	1.1948706356
C	-5.5096138762	-2.0421343421	-0.0035068567
O	-5.6742873884	-2.6642329365	-1.2694054096
H	-4.9505928494	-3.2933483763	-1.3818377451
H	-5.6978945820	-2.7658468157	0.7987807361

C	-6.5487123799	-0.9258661452	0.0852449867
O	-6.5660397047	-0.3591425141	1.3752436744
C	-7.4804830952	-0.9840490807	2.2707648573
H	-7.2318535600	-2.0397733174	2.4269333067
H	-7.3992136635	-0.4536186341	3.2199075179
H	-8.5065928983	-0.9030610264	1.8928796744
H	-7.5393178050	-1.3154618204	-0.1744170918
O	-6.2855503238	0.0797191209	-0.8561469512
C	-5.0086953355	0.7161650735	-0.7711440174
H	-4.9417441407	1.2880219040	0.1637506657
C	-5.0143110211	1.6696171934	-1.9715020729
H	-5.2016040846	1.0818057227	-2.8752067899
H	-5.8509284666	2.3671361132	-1.8365414960
O	-3.8044482428	2.3699508903	-2.1826823697
O	1.7768774724	1.9270062124	-1.3967342865
O	2.2201747733	2.2697725183	0.7975157517
H	-3.5442015653	2.8322925567	-1.3592360009
O	-0.6332775125	2.0044146757	0.3899165500
H	-2.1197247815	3.0436693893	0.3345299245
H	-2.8750178211	0.9763091182	1.5016288623
O	-2.8877571291	3.6170034906	0.1347624431
O	-3.1742187527	1.1485450004	2.4109834586
O	0.2958647473	0.6489618288	2.6398798667
H	-2.5098004991	4.4728298655	-0.0964029482
H	-4.1067075018	0.9028897647	2.3985439632
H	0.4465873071	-0.2807120516	2.3904273856
H	-0.3582827851	1.4412557473	1.1470359197
H	1.1665512392	1.0635137072	2.5813596088

D-Man-2e-1

0 1

O	-4.3962086364	0.1806416513	0.2465785696
C	-5.4940863611	0.0872138734	-0.6606926176
C	-6.2376828700	1.4132844227	-0.6168978423
O	-5.4048342435	2.5163135958	-0.9405212762
H	-4.9331035403	2.7752308003	-0.1202534649
H	-7.0490450112	1.3835579929	-1.3472389952
H	-6.6745626689	1.5512479310	0.3799180487
H	-5.1056987567	-0.0916487716	-1.6748214933
C	-6.4039118582	-1.0756438249	-0.2488371339
O	-7.4060923851	-1.2246641016	-1.2425290943
H	-7.9484397387	-1.9860268290	-1.0014515315
H	-6.8703923649	-0.8270696836	0.7166946256
C	-5.5980349460	-2.3664430805	-0.0860496546

O	-6.4176434229	-3.4131463949	0.4088460428
H	-6.5994245045	-3.2272944162	1.3405660577
H	-5.2574719970	-2.6874926276	-1.0762085207
C	-4.3649410041	-2.1530664383	0.7996319796
O	-4.7159850763	-1.9583835832	2.1612854363
H	-5.0682835492	-1.0654718324	2.2761250483
H	-3.7471165790	-3.0534227250	0.7706428846
C	-3.5754632349	-0.9766793730	0.2318472501
H	-3.2785010338	-1.2022918210	-0.8020917271
O	-2.4592013913	-0.7088178885	1.0211376359
C	-1.2577918311	-0.3616079284	0.3380090834
C	-0.2262432661	-1.4684503804	0.6082463033
O	1.0350119415	-1.0897587460	0.0588241400
C	-0.5787181236	-2.8080053281	-0.0138029487
O	-0.7452891625	-2.7421330307	-1.4223938475
H	0.1034872736	-2.5083682516	-1.8175912891
H	0.2064014701	-3.5262117789	0.2495436722
H	-1.5213945440	-3.1649795546	0.4033665471
H	-0.1305110913	-1.6069007020	1.6950916238
H	-1.4457575286	-0.2843202734	-0.7377854891
C	-0.7470368622	0.9791218559	0.8547189795
H	-0.6735380840	0.9270693466	1.9493985295
C	0.6588390975	1.2743392331	0.3237712000
O	0.5659606181	1.4526941187	-1.0929302158
C	1.2939404882	2.3584069757	-1.7671860823
C	2.1356765472	3.3474431785	-1.0211426722
H	1.5049595945	3.9671041027	-0.3771343619
H	2.8744701291	2.8406501761	-0.3954155066
H	2.6428230495	3.9818807729	-1.7460558800
H	1.0452782044	2.1708199605	0.8120071196
C	1.5793151877	0.0956025207	0.6142715418
H	1.6911568288	-0.0249977919	1.7021452006
O	2.7966258106	0.3166202014	-0.0020491050
C	3.9014540508	-0.4608906603	0.4661685524
H	3.6330569488	-0.9623022263	1.4040212146
C	4.2840534478	-1.4947923328	-0.5880047198
O	3.3299267296	-2.5409799472	-0.6959844129
H	2.4391181792	-2.1762167879	-0.5492852803
H	4.3878472724	-0.9754585006	-1.5493485387
C	5.6211581996	-2.1486829914	-0.2425265363
O	5.5069429264	-2.9640016738	0.9143215691
H	4.6921199609	-3.4755429929	0.8190807184
H	5.9501395479	-2.7538626509	-1.0961428734
C	6.6917102075	-1.0951304630	0.0522836261
O	7.0387787549	-0.3916165940	-1.1185369747
C	8.1098373402	-0.9775085975	-1.8519885369

H	7.8490754971	-1.9788366349	-2.2127368253
H	8.2936521308	-0.3262904901	-2.7070126772
H	9.0146471722	-1.0356558552	-1.2353048859
H	7.5757275564	-1.5785818549	0.4823845275
O	6.2551865910	-0.1796086759	1.0238041953
C	5.0502590479	0.5216831361	0.7075724651
H	5.1985643843	1.1255009158	-0.1970260178
C	4.7555750696	1.4366394166	1.8821701608
H	3.7975188841	1.9342369916	1.7174941719
H	4.6854159576	0.8368840001	2.7988316308
O	5.7279298035	2.4615670309	2.0271430619
O	1.1991533636	2.3353845191	-2.9837020354
H	6.5732053235	2.0464148166	2.2359787947
O	-1.6491633919	1.9961972571	0.4716217924
H	-2.3482102438	1.9505608433	-1.2536376914
H	-3.2964373509	2.4645366997	1.2029296871
H	-1.2294656313	4.5207876752	2.1998967934
O	-2.8337489213	2.0018838653	-2.0973728248
O	-4.0815053327	2.9973381760	1.4328610422
O	-0.8196321722	4.4221151613	1.3317433546
H	-3.7622842381	2.1086382194	-1.8224787116
H	-3.7493811339	3.8992782999	1.5108346104
H	0.1306445129	4.4208904382	1.5000726583
H	-1.2906950177	2.8720659149	0.7519091454

D-Man-2e-2

0 1

C	-6.7580085418	0.4697055202	-0.2128585652
C	-6.7234193981	-1.0023413232	0.2021874145
C	-5.4848614388	-1.3358077014	1.0553092175
C	-4.2640925782	-0.8706948514	0.2681214144
O	-4.3667228790	0.5259308282	0.0504202457
C	-5.4208068245	0.8624828750	-0.8466215941
H	-6.9276920227	1.0926951560	0.6772088946
O	-7.7802478182	0.7028669490	-1.1689620682
H	-6.6670567658	-1.6071037957	-0.7130952803
O	-7.9347268828	-1.2747778176	0.8876977172
H	-5.4346829523	-2.4178588236	1.2026956734
O	-5.5271033951	-0.7593744110	2.3478584242
H	-4.2301875479	-1.3823879254	-0.7065936867
H	-5.2884758754	0.3075194498	-1.7877519079
C	-5.2888169857	2.3500078514	-1.1142296524
H	-8.6158887850	0.4082271092	-0.7861647192
H	-8.0777562896	-2.2282599325	0.8984882775

H	-5.4143614727	0.1974359334	2.2718668686
H	-5.3885796054	2.9014540489	-0.1705074956
H	-6.0786810193	2.6733188070	-1.7939544080
O	-4.0517621511	2.6570348301	-1.7453201615
H	-3.3374085506	2.3934071145	-1.1508141657
O	-3.0981113509	-1.1065570290	0.9937460840
O	2.0786256035	-0.7480975526	-0.7542980778
O	-0.0354798509	0.9200314397	-1.0500741186
C	-1.9071006049	-0.8163338529	0.2693420382
C	-1.0425173588	0.1563635333	1.0771540847
C	0.2323634509	0.4249693073	0.2821370530
C	0.9560717008	-0.9069558208	0.0609801818
O	0.1205947911	-1.8044739749	-0.6347798861
C	-1.1009530130	-2.1088218623	0.0431369383
H	-2.1623855496	-0.3684445037	-0.6968673077
H	-0.7336256282	-0.3565026821	1.9969682426
O	-1.7362196804	1.3197324378	1.4799481649
H	0.8889511205	1.1197992341	0.8101319834
H	-0.8690056297	-2.5574697007	1.0208769835
C	-1.8438226785	-3.1140366339	-0.8073784741
H	-1.9828184290	-2.7081497190	-1.8172309079
H	-2.8268611428	-3.2979148689	-0.3608752941
O	-1.0864882031	-4.3184572473	-0.8481401426
H	-1.5687836054	-4.9573144382	-1.3845762932
H	1.2307244686	-1.3246471496	1.0388326693
O	5.5790688393	-2.0243095419	1.4756121752
C	-0.3074775808	2.2016500746	-1.2672408241
C	-0.3999020395	2.5210212875	-2.7206560000
H	-0.7881164492	3.5290452735	-2.8562534938
H	-1.0420115474	1.7929817296	-3.2210572357
H	0.5994235613	2.4463607989	-3.1601430505
C	3.3520776450	-0.7503566481	-0.1097353983
C	4.0801289827	-2.0478113718	-0.4518342693
C	5.5197946239	-2.0168660647	0.0541370683
C	6.2218072150	-0.7419251719	-0.4086232887
O	5.4766041911	0.4095065127	-0.0805280527
C	4.1544339736	0.4515061818	-0.6244007638
H	3.2210051018	-0.6705857653	0.9727668810
H	4.1036669381	-2.1529269227	-1.5435726957
O	3.4513190154	-3.1800043191	0.1311398332
H	6.0622876140	-2.8816627857	-0.3420141911
O	6.4375244979	-0.8635907801	-1.7879580675
H	4.2016287042	0.4008042357	-1.7207238428
C	3.5682158526	1.7983374645	-0.2602371545
H	5.0787072467	-2.7873696469	1.7924268162
H	2.5736711864	1.8875978334	-0.7072928972

H	4.2057318341	2.5804778011	-0.6894581734
C	7.1786896898	0.2186387419	-2.3448943506
H	7.1728923100	-0.6172013130	0.1218826105
H	8.1204498645	0.3593799685	-1.8010961910
H	6.6013583215	1.1484066801	-2.3213174619
H	7.3914494603	-0.0486522178	-3.3803086109
H	2.6065499599	-3.3265027947	-0.3111522887
O	-0.4677255970	3.0150888557	-0.3619405488
H	-1.4765651266	2.0633797461	0.9142976266
O	3.4952356547	1.9517515799	1.1536406699
H	4.2545643151	0.7815656570	2.4362852171
H	5.0270963959	2.8150548089	1.6157261756
H	0.7464641408	3.4132617469	1.0311306824
O	4.6745715898	0.1661032948	3.0618288652
O	5.8744717109	3.2618608053	1.8121362214
O	1.4229404245	3.6181577848	1.6991949679
H	5.0000327204	-0.5567017892	2.4955226291
H	6.5278839192	2.5545729534	1.7763836345
H	1.6946558255	4.5244084688	1.5131478049
H	2.7505973842	2.5573040405	1.3709876079

D-Man-2f

0 1

C	5.7045026824	2.8378579336	3.3374580559
C	5.8651275879	1.3316431618	3.5610671289
C	5.0724836783	0.5297813235	2.5255382102
C	3.6316606733	1.0381406640	2.5012057712
O	3.6103811067	2.4370770522	2.2505522799
C	4.2197177730	3.2085702017	3.2820907159
H	6.1701488304	3.1193043193	2.3835907470
O	6.2948544883	3.5795313476	4.3975447852
H	5.4747904747	1.0904565998	4.5549707346
O	7.2305093822	0.9530225462	3.5673284666
H	5.0686653933	-0.5262660542	2.8193905671
O	5.7073803423	0.6934792536	1.2692180688
H	3.1455078497	0.8394025067	3.4680246932
H	3.7433637659	2.9791027205	4.2464329920
C	3.9707724768	4.6712591428	2.9285519936
H	7.2519565244	3.4678925759	4.3500486581
H	7.5528515536	0.9997860299	2.6568539506
H	5.3834689413	0.0118299689	0.6692879913
H	2.9020719909	4.8130028101	2.7495086175
H	4.5150109772	4.9222874690	2.0084873993
O	4.3365962714	5.5424244955	3.9882571241

H	5.2353615641	5.3017223193	4.2595340935
O	2.9456171756	0.4184490763	1.4613384973
O	-2.3661113598	0.0635845222	0.2561617745
C	1.5182361348	0.5394635501	1.4852024436
C	1.0286404959	1.5286406099	0.4259981800
C	-0.5051780014	1.5263675179	0.3855273563
C	-0.9898595279	0.0994892492	0.1597037624
O	-0.4756321019	-0.7732374109	1.1664653418
C	0.9460845253	-0.8537590861	1.1880242606
H	1.1932504885	0.8586484046	2.4827511993
H	1.3862625345	1.1763026820	-0.5517738511
O	1.4739724002	2.8593194220	0.6300846917
H	-0.8310333254	2.1315293057	-0.4682966232
H	1.3098110542	-1.1960262151	0.2082033758
C	1.3080827005	-1.8950544113	2.2462153167
H	0.5846240321	-2.7110790619	2.1825122053
H	1.2450847824	-1.4517038947	3.2481021472
O	2.5871193251	-2.4699643083	2.0269626888
H	3.2385719888	-1.7576558055	2.0312295436
H	-0.6445377556	-0.2525077038	-0.8230614778
O	-3.6774722430	-3.7937329069	-1.2495260171
C	-3.1026302965	2.9341655025	-1.9997706939
C	-2.8690460308	4.1345524017	-1.1381464144
O	-2.7383323228	2.8190498199	-3.1594350308
H	-2.0988425227	4.7624171563	-1.5847629508
H	-2.5814007886	3.8303513116	-0.1294187796
H	-3.8018288151	4.7026609058	-1.0656754708
C	-3.0573656150	-0.9662719345	-0.4472016800
C	-3.4226317971	-2.1159976381	0.4913414001
C	-4.3569202290	-3.1020959425	-0.2136714394
C	-5.5336769996	-2.3753323650	-0.8604147315
O	-5.0973579220	-1.3181201139	-1.6931993497
C	-4.3268435128	-0.3261478707	-1.0201078529
H	-2.4340113883	-1.3364365206	-1.2704041460
H	-3.9442951684	-1.6954740137	1.3606555736
O	-2.2895330597	-2.8606630510	0.9088187973
H	-4.7494405197	-3.8135373300	0.5220766259
O	-6.3638294548	-1.9298152689	0.1750194519
H	-4.9131500675	0.1115383551	-0.2019270290
C	-4.0411262893	0.7498806517	-2.0582227280
H	-2.8134399688	-4.0492601238	-0.8993533267
H	-4.9183945751	0.8896000174	-2.6944948852
H	-3.1793229302	0.4902832910	-2.6767392664
O	-3.7824188200	1.9784131605	-1.3586176737
C	-7.5589620797	-1.2973895969	-0.2741760509
H	-6.0774231509	-3.0474765664	-1.5339682280

H	-8.0954150995	-1.9433575277	-0.9793001534
H	-7.3428902316	-0.3367659196	-0.7527132132
H	-8.1745545898	-1.1302498643	0.6099681905
H	-1.5756095053	-2.2408754586	1.1479174682
H	-0.9185630878	2.9860106644	1.6417533893
O	-0.3820203522	4.6933249632	1.5746745785
H	-0.8668833450	5.1269954667	0.8625647985
H	-1.2749928538	0.9671597530	3.1042388142
O	-1.3926277580	0.2493423796	3.7528909068
H	-1.1939564190	-0.5299577051	3.2168963841
H	-2.9922655520	2.1151467941	1.6551362736
O	-3.9483801054	2.3032854790	1.6660614322
H	-4.2013821827	2.1892387973	0.7399267364
O	-1.0832763020	2.0206812071	1.5819693966
H	2.2767051906	2.8557087017	1.1863273391
H	0.4011914369	4.2947024790	1.1509017065

D-Man-IN1

-1 1

C	5.6886596636	3.2441659004	1.1614215012
C	5.4618804144	2.7604215585	2.5939170601
C	4.7439525068	1.4112949656	2.5886416878
C	3.4675880093	1.5405720702	1.7539176611
O	3.7880144386	1.9340534410	0.4301608537
C	4.3753033012	3.2288514690	0.3685854344
H	6.4069881839	2.5805412684	0.6628529659
O	6.1783206070	4.5815669198	1.1383763680
H	4.8312103844	3.4902399752	3.1114365791
O	6.6777037409	2.6959701975	3.3197374560
H	4.4658862857	1.1387693108	3.6129018123
O	5.6414151403	0.4550861226	2.0445778511
H	2.8087935634	2.2897374316	2.2145816272
H	3.6845799750	3.9691835378	0.8013671675
C	4.5893598707	3.5417698234	-1.1103908651
H	7.0964368462	4.5759445480	1.4331827501
H	7.1735588720	1.9346779514	2.9880346641
H	5.3869532813	-0.4267422025	2.3361779502
H	3.6706438133	3.3289456798	-1.6608151604
H	5.3856180169	2.8980256415	-1.5074201090
O	4.8971002796	4.9126821125	-1.3235257207
H	5.6012689661	5.1454238023	-0.6988160045
O	2.8287882353	0.3010219170	1.6701825860
O	-2.2952445392	-1.4048841607	0.9111004894
O	-1.4008751670	1.1710518368	1.7387320213

C	0.9964766845	2.8658868438	-0.6254887845
C	1.0725474286	4.3530556800	-0.4989340419
O	1.0300450417	2.2613454299	-1.6846972888
H	1.3209660435	4.6615483646	0.5167314742
H	0.0916576325	4.7593071653	-0.7664802683
H	1.8052447199	4.7415181114	-1.2081097844
C	1.4042284546	0.2743359421	1.7998889068
C	0.6831697512	0.8366689251	0.5793714957
C	-0.8173545632	0.5282287996	0.6292515373
C	-0.9650103316	-0.9897401187	0.7669313783
O	-0.3526106728	-1.4173600882	1.9618888701
C	1.0535520354	-1.2168839660	1.9794294469
H	1.1115700045	0.8321858530	2.6974915710
H	1.1151763020	0.4043540895	-0.3265958544
O	0.8572539200	2.2596564415	0.5604427336
H	-1.2626663531	0.8610173713	-0.3127822675
H	1.5181910981	-1.7833305725	1.1587371201
C	1.5196171345	-1.7796659078	3.3213453272
H	1.0690474281	-2.7654409481	3.4542189925
H	1.1703383424	-1.1241544728	4.1297968558
O	2.9251794657	-1.9591272693	3.3891273184
H	3.3324757840	-1.1229803344	3.1271141900
H	-0.4964627900	-1.4893619616	-0.0954105791
O	-3.6702312564	-2.6899220145	-2.9691796893
C	-2.5575646146	2.0298480828	1.5178382738
C	-2.7942076598	2.6593849237	2.8864567633
H	-1.9167071324	3.2405575622	3.1793371572
H	-2.9834443164	1.8954311261	3.6450407316
H	-3.6576810665	3.3268298033	2.8278486735
C	-3.0521667031	-1.4744830165	-0.2959074167
C	-3.4084456551	-2.9358459720	-0.5568517567
C	-4.3300239230	-3.0593512338	-1.7674742607
C	-5.5254374714	-2.1198403021	-1.6184141393
O	-5.1158479911	-0.7979939781	-1.3377348171
C	-4.3326880761	-0.6451206297	-0.1492611888
H	-2.4674200954	-1.0801504968	-1.1350851891
H	-3.9381068122	-3.3241997348	0.3221556990
O	-2.2572892862	-3.7261772421	-0.8160115701
H	-4.6992874629	-4.0885070915	-1.8404746481
O	-6.3487640529	-2.6475763608	-0.6147012495
H	-4.9002725222	-1.0050358166	0.7189398771
C	-4.1109059431	0.8566321578	-0.0119912473
H	-2.8750220569	-3.2311211794	-3.0488232655
H	-5.0675906381	1.3513579673	-0.2169653795
H	-3.3915504164	1.2019219019	-0.7570431431
O	-3.7038950728	1.1740219807	1.3082152831

C	-7.5604297347	-1.9209780674	-0.4323926976
H	-6.0707848847	-2.0442736619	-2.5662722386
H	-8.1027271147	-1.8322528085	-1.3814054114
H	-7.3664607140	-0.9218569293	-0.0292868774
H	-8.1607483832	-2.4881176181	0.2794554207
H	-1.7147598433	-3.7481339503	-0.0184283597
O	-2.3649197234	2.9007149278	0.5316011636
H	-3.8344460859	3.7147042410	0.1615677533
O	-4.6535612246	4.2223871969	-0.0631941930
H	-4.6164824882	5.0016095520	0.5016409774
H	-1.9148194318	4.4744109103	1.0092779813
O	-1.7370936900	5.4187558712	1.2510696973
H	-0.7923286318	5.4559009599	1.4333516373
H	-2.1117943892	2.8707433464	-1.1601368302
O	-2.0537806114	2.9800927077	-2.1392983922
H	-2.9674526845	3.1100836800	-2.4150103044

D-Man-IN2

-1 1

O	-4.2846320534	0.2939587479	-0.6817741626
C	-5.3243691327	-0.4649247496	-0.0796066397
C	-5.6523356336	-1.5657132681	-1.0670766395
O	-4.5359939397	-2.4311885953	-1.2486866196
H	-3.7184000925	-1.8980888744	-1.2894955042
H	-6.4764798529	-2.1758496785	-0.6934894003
H	-5.9493230863	-1.1182893114	-2.0243473287
H	-4.9531604079	-0.9166300743	0.8546315192
C	-6.4904910855	0.4712638015	0.2430001997
O	-7.4800066642	-0.2807205750	0.9290523241
H	-8.1979032836	0.3192743106	1.1651972341
H	-6.9016986058	0.8716072944	-0.6932538578
C	-5.9846221701	1.6229619415	1.1154955474
O	-7.0692336478	2.5166294645	1.3106838403
H	-6.8618747875	3.0994741887	2.0505838066
H	-5.6763644270	1.1945175583	2.0788143173
C	-4.7569046644	2.3179075493	0.5142374356
O	-5.1431871138	3.0168741878	-0.6562845584
H	-4.3767099388	3.5005983148	-0.9863435539
H	-4.3402607976	3.0122179934	1.2548047081
C	-3.7208611892	1.2361921721	0.2084808232
H	-3.4252098602	0.7314772582	1.1403669648
O	-2.6143043100	1.8027188900	-0.4117711011
C	-1.3959416119	1.0638496813	-0.3139296105
C	-0.2682783969	2.0810374570	-0.5169951616

O	0.9810152281	1.4033549897	-0.5183687307
C	-0.2232195988	3.0969678590	0.6037881624
O	0.7368865234	4.0946839729	0.2726042157
H	0.7439232379	4.7498033477	0.9792586866
H	-1.2157023138	3.5414724513	0.7245405966
H	0.0532430834	2.5891947698	1.5366601468
H	-0.4065911981	2.5976826015	-1.4788946330
H	-1.3081912382	0.6367345636	0.6923743727
C	-1.3297850811	-0.0769862150	-1.3447171485
H	-1.8395022526	0.2383245616	-2.2585637934
C	0.0993139750	-0.5564837717	-1.6336973992
O	0.2976213114	-1.4978503888	-0.5955174992
C	-0.9418504602	-2.2397874466	-0.4291265352
C	-0.9890947904	-3.4093065217	-1.4069555869
H	-1.9731125074	-3.8817120356	-1.3455937152
H	-0.8197313216	-3.0877657579	-2.4378926607
H	-0.2291909457	-4.1455368799	-1.1307850754
H	0.1415557102	-1.0518708174	-2.6111956925
C	1.1440079922	0.5553960861	-1.6287050677
H	1.0110589868	1.1281260267	-2.5557836048
O	2.4713562879	0.1025885361	-1.6669459260
C	3.0352740190	-0.4230876201	-0.4616380054
H	2.2865246392	-0.4407133802	0.3324029964
C	3.5829154241	-1.8321137957	-0.7115220770
O	2.6164627246	-2.7476253178	-1.1928867305
H	1.7326390117	-2.4640838526	-0.8879593245
H	4.3613728442	-1.7661177915	-1.4800457248
C	4.2333779573	-2.3536860986	0.5784014730
O	3.3062128425	-2.4734815499	1.6407256060
H	2.5610826800	-3.0643964977	1.3982604304
H	4.7087913952	-3.3195544013	0.3681399426
C	5.3042319420	-1.3742800511	1.0582112798
O	6.3758639102	-1.4300463543	0.1549390423
C	7.4655858915	-0.5842941446	0.5103509482
H	7.7747290572	-0.7665478264	1.5467102487
H	8.2860652699	-0.8316901927	-0.1639868416
H	7.2047450405	0.4730334088	0.3900449157
H	5.6402220139	-1.6336324209	2.0689013011
O	4.7758958896	-0.0703939573	1.1833229784
C	4.2160912408	0.4586115237	-0.0202770536
H	4.9749728186	0.4495000839	-0.8125927894
C	3.8297934794	1.8932058992	0.2801959799
H	3.3746861836	2.3658206468	-0.5888832783
H	3.1468054282	1.9444458951	1.1284136926
O	4.9896117372	2.6414063667	0.6939220780
C	5.8154568892	3.1033973290	-0.2537423273

O	5.6115877395	2.9484700771	-1.4477221931
C	6.9842217699	3.8290988737	0.3333378572
H	7.6794035013	4.1123570346	-0.4556763936
H	6.6288746395	4.7247066920	0.8511192700
H	7.4851318845	3.1936802389	1.0679090799
O	-1.9452166187	-1.2605941936	-0.8476054404
O	-1.0821489504	-2.5948725501	0.8373924066
H	-2.6693096662	-3.3328382180	1.0552561049
H	-1.6617967565	-1.5055036509	1.9952054599
H	0.2754914332	-3.5403772142	1.2385547333
O	-3.5888515811	-3.6767201419	1.1375290691
O	-2.0356870331	-0.9788623409	2.7472051067
O	1.0770846102	-4.0842328102	1.4585254833
H	-4.0370986331	-3.3056226136	0.3563112316
H	-2.9878116994	-0.9921644996	2.5990282436
H	1.1683911554	-4.6975532001	0.7205595248

D-Man-IN3

-1 1

O	-3.7537337828	0.6818328266	1.9879376976
C	-4.8664682524	1.5356595744	1.7367158928
C	-4.7435733710	2.6750730531	2.7265769088
O	-3.5677438147	3.4387119138	2.4775269051
H	-2.8262901110	2.8340930315	2.2801162860
H	-5.5936795777	3.3524320480	2.6279535877
H	-4.7300326702	2.2716405856	3.7473140447
H	-4.7950365929	1.9373469931	0.7140533275
C	-6.1424982177	0.7052756340	1.8683007229
O	-7.2542537806	1.5280941244	1.5539824797
H	-8.0459908991	0.9763489305	1.5810476996
H	-6.2244642835	0.3436562678	2.9042357000
C	-6.0663448097	-0.4976523241	0.9165773096
O	-7.1918199745	-1.3435178206	1.0853667865
H	-7.0672144030	-1.8316581876	1.9112457906
H	-6.1181273829	-0.1188036732	-0.1097624317
C	-4.7543492066	-1.2840951834	1.0754152148
O	-4.7317553708	-2.0168980613	2.2906626057
H	-4.6104346405	-1.4024657163	3.0271100716
H	-4.6792344871	-2.0209968770	0.2723919953
C	-3.5908853578	-0.3000597628	0.9794009817
H	-3.5915102878	0.1843101141	-0.0094628544
O	-2.3867993792	-0.9521792284	1.2056296949
C	-1.2239629428	-0.3171485239	0.6771999289
C	-0.2013343254	-1.4329650052	0.4526397862

O	1.0049824168	-0.8384062311	-0.0208391642
C	-0.6351154123	-2.4600553269	-0.5796123590
O	-1.0122377377	-1.8892416869	-1.8241682258
H	-0.2506784519	-1.4271681217	-2.1953172822
H	0.1829170287	-3.1775261632	-0.7147557185
H	-1.5069973584	-2.9984044841	-0.2048392032
H	-0.0051147783	-1.9615593336	1.3972068991
H	-1.4613414781	0.1284905784	-0.2930013611
C	-0.6865074542	0.7896759092	1.6030686751
H	-0.8458739454	0.4984142860	2.6444175697
C	0.7789853448	1.1513079121	1.3338264373
O	0.6723784187	2.0671225644	0.2635395157
C	-0.5025950880	2.8813791089	0.4900193517
C	-0.1294803051	4.1254484405	1.2870322256
H	0.4109961969	3.8692882350	2.2026174453
H	0.4964480661	4.7743894740	0.6686318127
H	-1.0399648735	4.6682300602	1.5560921099
H	1.2168705485	1.6312554235	2.2187802064
C	1.6399417927	-0.0375420094	0.9619739749
H	1.8076425699	-0.6505437335	1.8611936203
O	2.8410392958	0.4101706674	0.4311936008
C	3.8853678613	-0.5645668175	0.3428776965
H	3.6031536934	-1.4656333578	0.9012138755
C	4.1331514157	-0.9166076603	-1.1215288675
O	3.0824293638	-1.6912097904	-1.6816130724
H	2.2446455109	-1.4562237429	-1.2434299275
H	4.2493193337	0.0216247367	-1.6789388255
C	5.4148394836	-1.7359006035	-1.2762596819
O	5.2603215412	-3.0306678747	-0.7145997782
H	4.3952510064	-3.3569117361	-0.9981153213
H	5.6567030609	-1.8168450230	-2.3432021267
C	6.5893786709	-1.0684365938	-0.5587901936
O	6.9609603434	0.1235337160	-1.2122249513
C	7.9683339566	-0.0480408229	-2.2038863657
H	7.6242637671	-0.6967442063	-3.0174767082
H	8.1840740608	0.9427943316	-2.6048524513
H	8.8787067425	-0.4699043755	-1.7614731294
H	7.4381312735	-1.7598808305	-0.5154970820
O	6.2724995017	-0.7796671880	0.7784067007
C	5.1334755922	0.0631177872	0.9703741109
H	5.3142719920	1.0409007015	0.5061995194
C	4.9724948467	0.2372538641	2.4696539918
H	4.0722361659	0.8219947351	2.6679679370
H	4.8596789290	-0.7482177774	2.9396427338
O	6.0571561277	0.9478536955	3.0496831449
O	-1.3282303527	2.0339445859	1.3631594561

O	-1.1093327774	3.1438862705	-0.6585755384
H	-0.0905977121	3.8954857741	-1.7733032902
H	-2.5972061625	3.9773756884	-0.4104565247
H	-1.7003384787	2.0110501890	-1.7536283760
O	0.4837177398	4.3275505118	-2.4567110188
O	-3.4639416856	4.4085791190	-0.2119786324
O	-2.0663576790	1.4833840414	-2.5102544172
H	1.0126133459	3.6104881961	-2.8218046623
H	-3.6319305463	4.1477261304	0.7110322376
H	-2.5009055253	0.7234143000	-2.1086448943
H	6.8561259888	0.4182526141	2.9410529101

D-Man-IN4

-1 1

C	-6.2834120297	0.5289091747	-0.2752979213
C	-6.3787804917	-0.9868601749	-0.1073198105
C	-5.2096948501	-1.5482246856	0.7196293170
C	-3.9191807595	-1.0635257241	0.0662476255
O	-3.9018851212	0.3550840030	0.0784926188
C	-4.8891411972	0.9207333700	-0.7795365932
H	-6.4575929877	1.0107415769	0.6976608469
O	-7.2347122408	0.9939495883	-1.2214362023
H	-6.3267203322	-1.4392987575	-1.1070680605
O	-7.6360705772	-1.2688113833	0.4865897701
H	-5.2457841875	-2.6407001296	0.6908189734
O	-5.2736891670	-1.1836478781	2.0871440659
H	-3.8721065030	-1.4109417002	-0.9777237715
H	-4.7527857034	0.5264783143	-1.7983374864
C	-4.6779391084	2.4249105445	-0.7912433362
H	-8.1123827492	0.7470663312	-0.9061390451
H	-7.8217703883	-2.2095656986	0.3860982125
H	-5.0497700056	-0.2476325250	2.1762253531
H	-4.7193539914	2.8031372176	0.2373262432
H	-5.4941844272	2.8839897579	-1.3563770017
O	-3.4488831054	2.7769691777	-1.4056321165
H	-2.8101415949	3.0522240873	-0.7148315756
O	-2.8233064724	-1.5197452585	0.7945005768
O	2.4715766283	-1.5687571991	-0.6455198021
O	0.5524315791	0.5249574691	-0.6076290806
C	-1.5605446923	-1.2609840039	0.1832589205
C	-0.6545942644	-0.5480272479	1.1843167932
C	0.7041979731	-0.3157297327	0.5239810571
C	1.2767213703	-1.6661421251	0.0919707809
O	0.3959323650	-2.3118562504	-0.7977291427

C	-0.8889817004	-2.5838864795	-0.2307451857
H	-1.6994262981	-0.6299635662	-0.7002563016
H	-0.5133311045	-1.2145004517	2.0462330173
O	-1.2101048605	0.6664588624	1.6463928558
H	1.3659350122	0.1392407814	1.2627374486
H	-0.7637321444	-3.2148457917	0.6625856267
C	-1.6845045046	-3.3420062481	-1.2690089867
H	-1.7197024307	-2.7624761203	-2.1999040687
H	-2.7057333811	-3.4826201846	-0.8990128195
O	-1.0612705794	-4.6037038859	-1.4853361349
H	-1.5724982686	-5.0780050495	-2.1498984755
H	1.4287949526	-2.2893452972	0.9874926138
O	5.4919542295	-0.6061871000	2.2513469411
C	1.0412569625	1.8707173378	-0.4588323236
C	0.5897554727	2.6040902480	-1.7170508604
H	0.9591210010	3.6317791297	-1.6758868627
H	-0.5022662018	2.6214953729	-1.7486974770
H	0.9538934562	2.1307824708	-2.6317534641
C	3.5569272279	-0.9125221864	0.0047501932
C	4.6462552356	-1.9138196100	0.3768014655
C	5.8353983411	-1.1756465633	0.9962203958
C	6.2858736261	-0.0187244532	0.1039270313
O	5.2061239363	0.8159680243	-0.2599615093
C	4.1468930903	0.1379425725	-0.9429963795
H	3.2190148047	-0.4128921192	0.9154901081
H	4.9853383336	-2.4296154056	-0.5300255561
O	4.2006531820	-2.8604179456	1.3374204411
H	6.6729037468	-1.8723770131	1.1144035686
O	6.9264851207	-0.5785192231	-1.0106443092
H	4.5502090454	-0.3652943211	-1.8326246350
C	3.1529261985	1.1650117751	-1.4412766495
H	5.1177763201	-1.3065550447	2.8003620185
H	2.4485233768	0.6445123758	-2.0923688369
H	3.6883598359	1.9121664396	-2.0380694999
O	2.4758818407	1.8114919210	-0.3718423891
C	7.4797415488	0.3901397296	-1.8967796245
H	6.9715356853	0.6369196973	0.6530278505
H	8.1230033440	1.0902151782	-1.3502003275
H	6.6928436442	0.9481655574	-2.4145895425
H	8.0749206162	-0.1584596373	-2.6273438251
H	3.5519598072	-3.4399228258	0.9211887812
O	0.6083134388	2.4426564471	0.6797748215
H	1.2512605355	4.0448024241	0.8462335062
O	1.6010527049	4.9591370297	0.9728632506
H	2.4138214443	4.8390895272	1.4755855494
H	-0.8665721925	3.3119458008	0.5213485860

O	-1.7367896550	3.7831367471	0.4998335230
H	-1.5314121368	4.6759440358	0.2005784226
H	1.7446953400	2.1630992710	2.0113055591
O	2.3751266845	2.0124932476	2.7545101707
H	3.1025760059	1.5250722881	2.3501391177
H	-0.6214276586	1.3965650926	1.3429549716

D-Man-RE1

-1 1

C	-6.6394914299	0.1279666729	0.4153846366
C	-6.4563417894	-0.4984038708	-0.9676261915
C	-5.2486960592	-1.4351056278	-0.9731852390
C	-4.0329615088	-0.6700687142	-0.4477888722
O	-4.2865946810	-0.1903235836	0.8617380795
C	-5.3324907695	0.7738389371	0.8913182206
H	-6.9287331818	-0.6508532889	1.1330534172
O	-7.6319799001	1.1487888629	0.3909436572
H	-6.2773542935	0.3045723919	-1.6897891667
O	-7.6319162068	-1.1651134972	-1.3952628762
H	-5.0404529031	-1.7594605445	-1.9991671292
O	-5.5696161354	-2.5489156287	-0.1541146435
H	-3.8177892494	0.1752007615	-1.1168677798
H	-5.0810145749	1.6121201864	0.2232412386
C	-5.4215850905	1.2863624491	2.3262572881
H	-8.4933333651	0.7330075540	0.2678759986
H	-7.7087844792	-1.9799444235	-0.8799397976
H	-4.9741359443	-3.2778142207	-0.3596958639
H	-4.4221464589	1.5553604267	2.6747778925
H	-5.8112468880	0.4912662045	2.9758413261
O	-6.2255452150	2.4543196320	2.4213528660
H	-7.0558576623	2.2650199280	1.9578355132
O	-2.9379893385	-1.5332470169	-0.3701344626
O	2.4747320164	-0.9018354825	-0.4180197694
O	0.5782633470	0.9627152049	-1.1263225840
C	-1.9669118868	1.9587322264	1.2509085705
C	-2.6499015066	3.2475144428	0.9271343568
O	-1.5635110158	1.6369742587	2.3553541680
H	-3.3036523058	3.1462194159	0.0604921176
H	-1.8753583584	3.9859277115	0.6942750779
H	-3.2111886287	3.5939487015	1.7953660324
C	-1.6578723315	-1.0203561026	-0.7382847924
C	-1.0979384124	-0.0477758758	0.2975749946
C	0.3934043804	0.2234809862	0.0828076205
C	1.1414605513	-1.0981430232	-0.0607290492

O	0.5928764394	-1.8592709883	-1.1124601666
C	-0.7496543501	-2.2566141404	-0.8643535192
H	-1.7294320319	-0.5220580868	-1.7130889845
H	-1.2429124582	-0.4593110460	1.2998526467
O	-1.8130043146	1.1868725692	0.1647875618
H	0.8002513837	0.7827480705	0.9271791094
H	-0.7983325893	-2.8304351898	0.0726851022
C	-1.1311529152	-3.1617252773	-2.0354796241
H	-0.3172653112	-3.8724292939	-2.1932444437
H	-1.2463331043	-2.5569661596	-2.9441011066
O	-2.2985982110	-3.9278947448	-1.7863680835
H	-3.0091303039	-3.3106573422	-1.5692559853
H	1.0575101785	-1.6513358451	0.8853233953
O	4.6467888230	-2.1698177184	3.0836779270
C	0.7162149657	2.2987342826	-1.0491795691
C	0.8130654162	2.9138681623	-2.4068321480
H	-0.1530086907	2.8029874910	-2.9092486626
H	1.5660724327	2.3952427831	-3.0036579741
H	1.0593258283	3.9708232518	-2.3182901398
C	3.4304348265	-0.9300423518	0.6427285418
C	4.0858474604	-2.3073669284	0.7131525647
C	5.1829707648	-2.3158056834	1.7767442002
C	6.1378115051	-1.1423183800	1.5619136085
O	5.4471665611	0.0826795955	1.4440948491
C	4.4951848661	0.1375928511	0.3697346692
H	2.9346135039	-0.6941371622	1.5910978211
H	4.5442414916	-2.5209984737	-0.2603277076
O	3.1604890673	-3.3341317154	1.0453697234
H	5.7524891648	-3.2490954466	1.7049552160
O	6.9047363309	-1.4369573742	0.4249892189
H	5.0056238435	-0.0870249189	-0.5756737245
C	3.9550073069	1.5762036839	0.2940851862
H	4.0225397434	-2.8907103082	3.2312329855
H	4.6562373499	2.1961048683	0.8792784517
H	2.9869262702	1.6221986802	0.8215078393
C	7.9048457652	-0.4625438274	0.1429738217
H	6.7838095238	-1.0140744220	2.4384276354
H	8.5348816701	-0.2906291091	1.0241068730
H	7.4571269797	0.4840461541	-0.1760779797
H	8.5122250002	-0.8666379926	-0.6674407485
H	2.5994505968	-3.5051277166	0.2797232289
O	0.7219565822	2.9072364339	0.0055190973
O	3.8429449465	2.0568251051	-1.0132403985
H	3.6205409417	3.6415155668	-0.9086312002
H	5.3212463234	2.3994113545	-1.5118287473
H	3.5569781886	0.9728213536	-2.1973055457

O	3.5481301129	4.6438449514	-0.8349723848
O	6.2513406604	2.6472511524	-1.8149288819
O	3.3908503811	0.2795004733	-2.9019965464
H	4.4592059087	4.9555129688	-0.8428743365
H	6.2781040750	3.6086552370	-1.7652430787
H	2.9479393761	-0.4116884511	-2.3919064423

D-Man-RE2

-1 1

C	-6.4780897910	0.2827657543	-0.4996022870
C	-6.1567901130	1.4719136237	0.4063757802
C	-4.8070335677	2.0813783149	0.0242745761
C	-3.7526987421	0.9726238421	0.0320601027
O	-4.1160278569	-0.0526531483	-0.8782245399
C	-5.3167490515	-0.7185632182	-0.5073535787
H	-6.6388908271	0.6404841147	-1.5252088645
O	-7.6372889576	-0.4104479816	-0.0477999451
H	-6.1010231835	1.1159706532	1.4400812017
O	-7.1894561931	2.4421008356	0.3718316929
H	-4.5244510517	2.8385044087	0.7631123002
O	-4.9454697046	2.6692816346	-1.2615028451
H	-3.6727609639	0.5573335062	1.0459621053
H	-5.2053351516	-1.1505226301	0.4993281101
C	-5.5177813769	-1.8416761650	-1.5210741374
H	-8.4053004605	0.1536640855	-0.1974699360
H	-7.1294762115	2.8966589690	-0.4798432532
H	-4.3771334536	3.4437583209	-1.3208283414
H	-4.5919649689	-2.4149905812	-1.6078196165
H	-5.7498469111	-1.4082899933	-2.5030651658
O	-6.5344707277	-2.7481987056	-1.1171453829
H	-7.3083688601	-2.2174342594	-0.8745643920
O	-2.5266587192	1.4803004833	-0.3979799414
O	2.6718317975	0.0042600562	0.3183851394
C	-2.2445202779	-2.4298558100	0.1964854014
C	-3.2133250372	-3.1931689109	1.0449831340
O	-1.7681506156	-2.8544533214	-0.8456418388
H	-3.5664589699	-2.6049958252	1.8917500890
H	-2.7134039285	-4.0969434904	1.4054036408
H	-4.0583356055	-3.5074395298	0.4279602141
C	-1.3414265607	1.0468362618	0.2799270945
C	-0.9871312226	-0.4118271254	0.0129477397
C	0.4264996222	-0.7680544081	0.5263551900
C	1.3840112341	0.2230503962	-0.1415552985
O	1.0292316658	1.5567306464	0.2254479911

C	-0.2346938691	1.9658693157	-0.2690322016
H	-1.4584803215	1.2143943349	1.3576016118
H	-1.0584168602	-0.6080014652	-1.0607424657
O	-1.9432932834	-1.2336913457	0.7063723730
H	0.6606392905	-1.7612887599	0.1118558383
H	-0.2402825940	1.9053884364	-1.3675179484
C	-0.3971299006	3.4217638840	0.1632207996
H	0.5267889403	3.9547522293	-0.0729453734
H	-0.5573699518	3.4643447552	1.2487446536
O	-1.4445169703	4.0842578196	-0.5278949004
H	-2.2285900904	3.5227993058	-0.4551892051
H	1.3375247776	0.1379752641	-1.2387061069
O	5.3005335313	2.9417059853	-1.4971940647
C	2.5068431766	-3.0954228414	-1.2265874342
C	1.9298189213	-4.0841017370	-0.2628747442
H	0.8929976301	-4.2938084866	-0.5248403221
H	1.9962091437	-3.7075816559	0.7604144393
H	2.5114788710	-5.0096275145	-0.3231274618
C	3.7324744004	0.6176757732	-0.4113375396
C	4.3203481459	1.7766353569	0.3937134165
C	5.5985789710	2.2963170884	-0.2690995540
C	6.5594190160	1.1543124260	-0.5859708794
O	5.9201977301	0.1272637970	-1.3183437905
C	4.8007358473	-0.4542845012	-0.6538640351
H	3.3605136927	0.9816661711	-1.3767068635
H	4.5673373753	1.4024144857	1.3956168832
O	3.4360101985	2.8830329452	0.4882939005
H	6.0993000047	2.9940160747	0.4122697470
O	7.0787875567	0.6946414093	0.6303583036
H	5.1147672704	-0.8780811529	0.3091738687
C	4.3278631923	-1.5818752310	-1.5618863850
H	4.5065356235	3.4730021094	-1.3451574049
H	5.1943532835	-2.0784595894	-2.0041139565
H	3.6754341756	-1.2122692221	-2.3543487464
O	3.6367688113	-2.5523395300	-0.7607383798
C	8.0771021859	-0.3108189953	0.4807177140
H	7.3632102126	1.5022948593	-1.2448531246
H	8.8627750609	0.0261734453	-0.2060285310
H	7.6449805094	-1.2458809407	0.1100035748
H	8.5037791450	-0.4765357399	1.4702624399
H	2.5170555504	2.5562426895	0.4770915237
O	2.0296348597	-2.8200736860	-2.3163518093
O	0.5199246101	-0.7646164397	1.9059734796
H	2.0005827875	-1.3878084763	2.3822128010
H	0.7197814723	0.6613910149	2.6864335981
H	-0.0495483408	-2.1467709050	2.5489220007

O	2.8778870825	-1.7632305315	2.6730237178
O	0.8680406106	1.5053970603	3.2033470601
O	-0.3188079859	-3.0084123123	2.9842779794
H	3.4694372250	-1.5091070467	1.9551829331
H	1.0958693998	2.1396692581	2.5137809994
H	-1.2789863786	-2.9733589921	3.0429684326

D-Man-RE3

0 1

O	4.4176800186	-0.2195394362	0.1455776386
C	5.5325705786	-0.7061468831	0.8906739636
C	5.8353397900	0.3119493684	1.9760707744
O	4.7804877986	0.4208468583	2.9166322368
H	4.1328760480	1.0688264855	2.5686489604
H	6.7305962283	-0.0070100502	2.5146950681
H	6.0412633146	1.2852256601	1.5111441462
H	5.2669423237	-1.6673348907	1.3571212444
C	6.7064418159	-0.9149747623	-0.0697209607
O	7.8019608343	-1.4434897267	0.6627785489
H	8.5000492574	-1.6531921929	0.0294931004
H	6.9804459463	0.0567790440	-0.5065019364
C	6.2926008097	-1.8700772065	-1.1904663695
O	7.3440145838	-2.0349585176	-2.1286192561
H	7.3801915207	-1.2284204421	-2.6618757124
H	6.1150022205	-2.8577585331	-0.7515044309
C	5.0077857724	-1.3985331924	-1.8825791396
O	5.2526443064	-0.2598101497	-2.6880065731
H	5.1303664581	0.5685630134	-2.1761936397
H	4.6556059600	-2.1924007575	-2.5479575042
C	3.9496237100	-1.1535160832	-0.8108478309
H	3.7220467728	-2.0997784002	-0.2969512129
O	2.8162850219	-0.6192139537	-1.4218501810
C	1.6851370551	-0.4515674199	-0.5762989359
C	0.6304901921	-1.5194423239	-0.9106207632
O	-0.5439263629	-1.2665907790	-0.1361048809
C	1.0515929896	-2.9466913265	-0.6138147406
O	1.4222171773	-3.1533625008	0.7407468475
H	0.6447265428	-3.0043423212	1.2923135155
H	0.2274229297	-3.6125627656	-0.8950513752
H	1.9171048752	-3.2026596162	-1.2285512750
H	0.3849448942	-1.4529667960	-1.9807396952
H	1.9812451960	-0.5305654060	0.4739412952
C	1.1028079002	0.9318709964	-0.8447237019
H	0.8678279833	1.0042247405	-1.9153017094

C	-0.2087250283	1.1202314612	-0.0783766957
O	0.0464496225	1.0616062775	1.3286415415
C	-0.0807068773	2.1162244821	2.1498364241
C	-0.4205091213	3.4612888147	1.5876545848
H	-1.4130165784	3.4447184933	1.1277316871
H	-0.4141458057	4.1852500557	2.4006601986
H	0.3068351510	3.7555152071	0.8272357886
O	0.0988111171	1.9072152451	3.3385482531
H	-0.6751643574	2.0638980915	-0.3623999783
C	-1.1588111856	-0.0198865897	-0.4196611171
H	-1.4086591245	0.0260434828	-1.4905348949
O	-2.2916715608	0.0808464733	0.3701525549
C	-3.4267880037	-0.6795623190	-0.0548131628
H	-3.2685812806	-1.0491057460	-1.0749982779
C	-3.6467577013	-1.8508555397	0.8990803084
O	-2.6633076375	-2.8641335193	0.7542140840
H	-1.8119796785	-2.4517791138	0.5226740952
H	-3.6374072157	-1.4564152115	1.9232815850
C	-5.0010958602	-2.5123993988	0.6456302547
O	-5.0171260310	-3.1764804333	-0.6087345047
H	-4.1942926901	-3.6803210179	-0.6708549258
H	-5.1989377708	-3.2297641981	1.4513504451
C	-6.1270550909	-1.4767151581	0.6205345495
O	-6.3400064525	-0.9293133694	1.9012542407
C	-7.2714512748	-1.6584562649	2.6941235937
H	-6.9068221679	-2.6673957551	2.9173744186
H	-7.3862778427	-1.1068453648	3.6276831448
H	-8.2408697275	-1.7256258754	2.1861123069
H	-7.0467923272	-1.9401458933	0.2471949889
O	-5.8414779406	-0.4360549374	-0.2782105223
C	-4.6289215904	0.2709136659	-0.0241121742
H	-4.6762089066	0.7483254799	0.9625706571
C	-4.4944999628	1.3363228067	-1.0923447178
H	-3.5303209075	1.8362224462	-0.9972945679
H	-4.5904731179	0.9032791477	-2.0894416508
O	-5.4847258757	2.3583321813	-0.8952530088
C	-6.5788249156	2.3711773279	-1.6729367143
O	-6.7649101378	1.5833805954	-2.5849521690
C	-7.5182668933	3.4643926100	-1.2738094070
H	-8.3487900841	3.5164832485	-1.9763816298
H	-7.8978990513	3.2592890071	-0.2686185348
H	-6.9900201071	4.4203987527	-1.2437209786
H	2.7333400069	2.1755831482	1.1249673479
O	3.1887119228	2.4387624936	1.9510524539
H	3.8794551204	3.0466743367	1.6621511216
O	1.1718936927	4.2198931518	-1.6404221319

H	1.2593761728	4.1099839990	-2.5950945879
H	3.7990572972	2.0127025063	-1.1788495681
O	4.6683356682	2.2236706500	-1.5704364897
H	4.4674170373	2.7733551192	-2.3365895180
O	2.0436798642	1.9272415999	-0.4988758507
H	1.7341978302	2.7863241847	-0.8732658117
H	0.2219933566	4.2873063587	-1.4824842131

D-Man-RE4

-1 1

O	4.1341160119	0.4589220116	0.0141212430
C	5.2457172633	0.4476950763	0.9100090251
C	5.5713835569	1.8931994447	1.2386175324
O	4.5967879213	2.4931816522	2.0773799968
H	3.7465750767	2.5276610865	1.6031615034
H	6.5183019648	1.9311313769	1.7804962414
H	5.6838326357	2.4568446681	0.3024564418
H	4.9646398569	-0.0798455202	1.8343295220
C	6.4050030350	-0.2952579397	0.2402116386
O	7.4773449691	-0.3798024480	1.1657354021
H	8.1739971504	-0.9109237672	0.7602365775
H	6.7221366280	0.2703918629	-0.6489474315
C	5.9491490346	-1.6947278903	-0.1879005801
O	6.9799428093	-2.3656213379	-0.8944331448
H	7.0357413858	-1.9642828986	-1.7730611033
H	5.7631545179	-2.2827411478	0.7171341825
C	4.6552076926	-1.6491643123	-1.0112569452
O	4.8851768203	-1.1213931266	-2.3088487581
H	5.0118396145	-0.1649210819	-2.2457698438
H	4.2850618273	-2.6669578301	-1.1560068973
C	3.6201695480	-0.8374689784	-0.2374512506
H	3.4036762510	-1.3281701531	0.7235700462
O	2.4720803328	-0.7054203021	-1.0112116998
C	1.3002974049	-0.2505996549	-0.3279939211
C	0.3039281851	-1.4289709809	-0.2758595507
O	-0.9892420559	-0.9925318151	0.1279231140
C	0.7073076494	-2.5393595121	0.6789742253
O	0.7233966403	-2.1204299922	2.0406879016
H	-0.1718599291	-2.2080503523	2.3884556421
H	0.0170698918	-3.3796595764	0.5536487304
H	1.7114353413	-2.8884956324	0.4273963951
H	0.2480019672	-1.8574155409	-1.2883701281
H	1.5630905435	0.0872235769	0.6740810972
C	0.6731409827	0.8655420241	-1.1549308143

H	0.5583558685	0.4655060431	-2.1683293832
C	-0.7379108956	1.2798772287	-0.6932353515
C	2.2217952267	2.6628436158	-0.4700459364
C	3.1697293245	3.6425443411	-1.0897070747
H	2.6454369654	4.2672150036	-1.8167606906
H	3.6196229482	4.2631721572	-0.3155700920
H	3.9516446871	3.0920179431	-1.6208639103
H	-1.1326424814	1.9151135520	-1.5073073964
C	-1.5634426344	-0.0158226865	-0.7485219868
H	-1.5955977695	-0.4345446183	-1.7659338120
O	-2.8332012350	0.2592573211	-0.2838117473
C	-3.9231137873	-0.5446475339	-0.7257859018
H	-3.7113145696	-0.9584797460	-1.7199469006
C	-4.1658001500	-1.6743882525	0.2779022108
O	-3.1779254894	-2.6906235179	0.1914329084
H	-2.3014318350	-2.2657656585	0.2590510231
H	-4.1657213486	-1.2331971547	1.2832147124
C	-5.5181109436	-2.3383030325	0.0583648679
O	-5.5673696426	-3.0353469636	-1.1782586633
H	-4.7810528793	-3.5936665539	-1.2280018759
H	-5.7158925964	-3.0290229576	0.8873936715
C	-6.6255440198	-1.2852270404	0.0260374462
O	-6.7758961456	-0.6842629557	1.2920956387
C	-7.7404109451	-1.3219750132	2.1233674321
H	-7.4656576925	-2.3623358170	2.3312919473
H	-7.7633182980	-0.7638486801	3.0597842417
H	-8.7321586507	-1.2921953580	1.6563968637
H	-7.5675399669	-1.7455760109	-0.2917251241
O	-6.3570486755	-0.2925598970	-0.9282017308
C	-5.1207827047	0.4131758570	-0.7865221644
H	-5.1312847987	0.9960054733	0.1443682811
C	-5.1174082827	1.3573655442	-1.9949226253
H	-5.2764944456	0.7559245693	-2.8952578833
H	-5.9710973580	2.0386630052	-1.8851009654
O	-3.9198262646	2.0798050656	-2.2002037170
O	1.5498793352	1.9893933472	-1.4050552503
O	2.0942831177	2.4966311969	0.7328209122
H	-3.6536983010	2.5623230574	-1.3895311237
O	-0.8431611263	1.9126863453	0.5314574180
H	-2.0470695471	2.9804567472	0.1899387522
H	-2.1587283865	1.5193066320	1.7007130157
H	-0.0795273561	1.1634822445	1.9034640972
O	-2.8153175455	3.5627708676	-0.0925537026
O	-2.8553358310	1.4129959562	2.3907856470
O	0.3014692856	0.7795867395	2.7351855284
H	-2.4171783427	4.2978242172	-0.5713934940

H	-3.5491106451	0.9324619078	1.9251826739
H	0.4763275033	-0.1484296794	2.5185197851

D-Man-RE5a

-1 1

O	-4.4199721871	0.0823981457	0.3101796293
C	-5.5304308056	0.0494001984	-0.5814317110
C	-6.2714542587	1.3676394574	-0.4134312265
O	-5.4349853738	2.4888582912	-0.6476099298
H	-4.8989897104	2.6352854266	0.1656438263
H	-7.0925013982	1.4079313071	-1.1325843618
H	-6.6955287606	1.4164572762	0.5977621235
H	-5.1603795285	-0.0445434857	-1.6139818955
C	-6.4205856035	-1.1519793710	-0.2454235883
O	-7.4352050845	-1.2410193417	-1.2341912103
H	-7.9648622123	-2.0250234506	-1.0427460664
H	-6.8766380369	-0.9820147434	0.7418309096
C	-5.5952713333	-2.4406213267	-0.1903132327
O	-6.3945459322	-3.5315372860	0.2388282511
H	-6.5624149712	-3.4167238376	1.1844638830
H	-5.2695528314	-2.6834309498	-1.2074462399
C	-4.3471560843	-2.2806366985	0.6870793927
O	-4.6762176962	-2.1920189087	2.0652401137
H	-5.0330896327	-1.3127907665	2.2500811523
H	-3.7225438564	-3.1703963731	0.5793158920
C	-3.5808258652	-1.0551865723	0.1952549294
H	-3.3016537585	-1.2010497350	-0.8580688419
O	-2.4564868629	-0.8217277294	0.9790146254
C	-1.2723502754	-0.3963873042	0.2998517061
C	-0.2135319781	-1.4858518528	0.5295649769
O	1.0380466209	-1.0677702339	-0.0186678335
C	-0.5366763574	-2.8192547148	-0.1210645556
O	-0.7272515330	-2.7249978877	-1.5260596231
H	0.0971757628	-2.4183588671	-1.9228500539
H	0.2716853315	-3.5229559121	0.1110357988
H	-1.4631386565	-3.2126167757	0.2997075828
H	-0.1024995383	-1.6508733014	1.6116823983
H	-1.4765646230	-0.3003202619	-0.7715144980
C	-0.8142512042	0.9657731083	0.8464319872
H	-0.6916416416	0.8435705112	1.9394470149
C	0.5956319588	1.2742528927	0.3108849127
O	0.4973543526	1.4718737609	-1.1089372268
C	1.0486127423	2.5064304202	-1.7517672785
C	1.7690970156	3.5710240782	-0.9823938216

H	1.0795504887	4.0701612081	-0.2953120332
H	2.5884092144	3.1442820388	-0.3977211986
H	2.1647346891	4.2995064708	-1.6881759102
H	0.9719533202	2.1696505538	0.8052438156
C	1.5505274111	0.1210865495	0.5628711156
H	1.6670528496	-0.0249851488	1.6470678205
O	2.7683662297	0.3865735125	-0.0432153150
C	3.9021927502	-0.3303102389	0.4504089794
H	3.6760901214	-0.7467113976	1.4397833210
C	4.2826624071	-1.4519492052	-0.5136638130
O	3.3488850912	-2.5205690745	-0.5058529013
H	2.4486139293	-2.1533635810	-0.4259273207
H	4.3607341328	-1.0212179267	-1.5203376981
C	5.6361771641	-2.0523641480	-0.1337489354
O	5.5517999479	-2.7589836890	1.0950403118
H	4.7355945868	-3.2765576581	1.0667822945
H	5.9605507212	-2.7290221650	-0.9340151560
C	6.6971886542	-0.9645520141	0.0430621329
O	7.0263237684	-0.3845348899	-1.1988605926
C	8.1229132707	-1.0156673108	-1.8526388466
H	7.9141384152	-2.0718753410	-2.0572021959
H	8.2679980590	-0.4922244059	-2.7981221836
H	9.0325922483	-0.9354439178	-1.2457285562
H	7.5902731742	-1.3930542812	0.5107666423
O	6.2616371678	0.0401734375	0.9221034273
C	5.0400182427	0.6888366456	0.5599810829
H	5.1617204219	1.1942259573	-0.4068799943
C	4.7637910663	1.7222873096	1.6374462549
H	3.8080004139	2.2084184263	1.4329399053
H	4.7006244835	1.2225208596	2.6127150427
O	5.7474476661	2.7465434125	1.6621382212
O	0.9195506904	2.5309913315	-2.9676850689
H	6.5901938661	2.3462582188	1.9072119072
O	-1.6803566357	1.9996990249	0.5429353632
H	-2.3409799219	2.0042227649	-0.9859400919
H	-3.0598374073	2.3708961414	1.2696921807
H	-1.1099147012	3.3452420371	1.3328302698
O	-2.8173254868	2.0726433692	-1.8594017259
O	-3.9227801378	2.7470206593	1.6248880867
O	-0.8173150240	4.1431051860	1.8590991436
H	-3.7531285585	2.1334223109	-1.6047880640
H	-3.7223782351	3.6612086643	1.8536249063
H	0.1453762421	4.1202711165	1.8398724652

D-Man-RE5b

-1 1

C	-6.7344657889	0.4569170643	-0.1746092821
C	-6.6946826726	-1.0161677079	0.2361627236
C	-5.4493248977	-1.3492885501	1.0793281036
C	-4.2349218958	-0.8814706306	0.2839451572
O	-4.3413263353	0.5154523994	0.0700273634
C	-5.4025644350	0.8517101499	-0.8186191688
H	-6.8966842293	1.0773965681	0.7185478416
O	-7.7650252295	0.6918631106	-1.1213259062
H	-6.6440113114	-1.6182800068	-0.6812131243
O	-7.9003829251	-1.2931850372	0.9297768399
H	-5.3970303031	-2.4314654491	1.2251948709
O	-5.4827010260	-0.7735173100	2.3725068686
H	-4.2077860448	-1.3910804410	-0.6920141998
H	-5.2769196794	0.2973989902	-1.7610846796
C	-5.2734161931	2.3394880663	-1.0861736339
H	-8.5977722539	0.3994351519	-0.7306272156
H	-8.0422825635	-2.2468772522	0.9369833490
H	-5.3591518481	0.1820477828	2.2964749307
H	-5.3690250420	2.8903976213	-0.1417150926
H	-6.0670038514	2.6622765759	-1.7617932568
O	-4.0399355030	2.6478529853	-1.7233011408
H	-3.3219663209	2.3848053764	-1.1327504409
O	-3.0635648613	-1.1173565222	1.0002701094
O	2.1061171653	-0.7721803483	-0.7698589354
O	-0.0031699564	0.9099121698	-1.0627967605
C	-1.8774486573	-0.8270185316	0.2670472508
C	-1.0070314015	0.1444277981	1.0695077790
C	0.2650009590	0.4121276305	0.2686456733
C	0.9834150558	-0.9225902966	0.0452495715
O	0.1425862455	-1.8166505408	-0.6500502801
C	-1.0755401865	-2.1203515872	0.0331464784
H	-2.1396430568	-0.3773850916	-0.6965500882
H	-0.6932410370	-0.3693649119	1.9873226838
O	-1.6999191553	1.3077584893	1.4734905334
H	0.9267894152	1.1046418823	0.7947812385
H	-0.8397135721	-2.5722271379	1.0085336146
C	-1.8257117496	-3.1211110860	-0.8161526457
H	-1.9722224071	-2.7101567337	-1.8229082971
H	-2.8054018218	-3.3066342089	-0.3629818570
O	-1.0699553030	-4.3260814482	-0.8692249136
H	-1.5577168195	-4.9615216247	-1.4047325439
H	1.2555951132	-1.3424924049	1.0230395291
O	5.6077869352	-2.0062783651	1.4558631928
C	-0.2818042041	2.1926441123	-1.2726547792

C	-0.3213930857	2.5341662812	-2.7239924569
H	-0.8252622012	3.4890229788	-2.8663677628
H	-0.8215357131	1.7450175424	-3.2880668770
H	0.7089158093	2.6099619370	-3.0865674923
C	3.3792646912	-0.7632630009	-0.1198630927
C	4.1227787264	-2.0446199172	-0.4815609649
C	5.5577561021	-2.0007242773	0.0336297017
C	6.2434175797	-0.7152183170	-0.4237503433
O	5.4935192148	0.4237932161	-0.0771788651
C	4.1576348557	0.4685729631	-0.5978625066
H	3.2381688902	-0.7119616247	0.9626358031
H	4.1535648252	-2.1335513250	-1.5745608511
O	3.5061718274	-3.1958911401	0.0801511219
H	6.1131433878	-2.8588302846	-0.3599644699
O	6.4488241298	-0.8258163129	-1.8077226081
H	4.1939363736	0.4557980145	-1.6966160103
C	3.5558616455	1.7968824369	-0.1503928921
H	5.0947074968	-2.7617968861	1.7701368765
H	2.5766998410	1.8909262355	-0.6508644256
H	4.1947793575	2.5848464282	-0.5895341407
C	7.1687453249	0.2702791372	-2.3634238385
H	7.2005656642	-0.5895228847	0.0962659497
H	8.1149744169	0.4199037384	-1.8294369329
H	6.5799691452	1.1924928749	-2.3257694068
H	7.3738988490	0.0150542339	-3.4035383547
H	2.6485855124	-3.3233953254	-0.3430685381
O	-0.4771854213	2.9927290825	-0.3638818837
H	-1.4183296127	2.0549279564	0.9208498479
O	3.4493074865	1.9380072984	1.2332882037
H	4.1760723409	0.8428412344	2.3079394965
H	4.6387037270	2.9047559007	1.6668498230
H	2.2563803187	2.9712506041	1.5445494859
O	4.5843102434	0.2408678614	2.9850727265
O	5.3902365519	3.5293767615	1.9184761291
O	1.5171341311	3.6239045455	1.7432720039
H	4.9602168049	-0.4877749933	2.4629981048
H	6.1914768185	3.0078141531	1.8043123468
H	0.8092365946	3.3936251721	1.1258666319

D-Man-RE6

-1 1

C	6.5635497639	-0.1489838433	0.3999919823
C	6.3146704823	-1.3738624931	-0.4881021059
C	5.0930979976	-1.1807740330	-1.3858299145

C	3.9159563961	-0.7593049003	-0.5092370229
O	4.2338986857	0.4147005653	0.2148097715
C	5.2808006305	0.2051146395	1.1591034136
H	6.8575529721	0.7048990034	-0.2261905610
O	7.5810164761	-0.4254896645	1.3512081240
H	6.1250127791	-2.2305205590	0.1669373704
O	7.4708123132	-1.7036088816	-1.2409968372
H	4.8484088925	-2.1300093273	-1.8761951275
O	5.4178616831	-0.1924787258	-2.3503025703
H	3.6853638320	-1.5636831356	0.2063008801
H	5.0135619603	-0.6330612892	1.8211481061
C	5.3994145970	1.4722145574	1.9866189488
H	8.3654074101	-0.7107362150	0.8663215026
H	7.5643998434	-1.0447151090	-1.9422222405
H	4.7335398612	-0.1854495395	-3.0291937063
H	5.5275177568	2.3306799554	1.3138919467
H	6.2842100129	1.3994388186	2.6225461182
O	4.2867942196	1.6607364446	2.8443522231
H	3.5023039263	1.9234242253	2.3208665028
O	2.8234080792	-0.4828046675	-1.3275886049
O	-2.4114698478	0.1307542993	0.1052808511
C	1.6024369723	-0.2396560366	-0.6297019246
C	0.9344237585	1.0093335684	-1.2050803219
C	-0.3976030896	1.2610704070	-0.4686612987
C	-1.2540265357	0.0014667732	-0.6494306092
O	-0.5745793607	-1.1534151182	-0.1604922962
C	0.6625061818	-1.4402451609	-0.8080164064
H	1.8075823687	-0.0873584075	0.4343908084
H	0.7188299206	0.8239616275	-2.2644583012
O	1.7830610013	2.1430195813	-1.1854141957
H	-0.9212993599	2.0664090907	-1.0137631392
H	0.4896450053	-1.6019670412	-1.8828513122
C	1.1644478330	-2.7425426110	-0.2106737077
H	2.0940567261	-3.0359025470	-0.7033751958
H	0.4165727326	-3.5211406778	-0.4040226633
O	1.4395885256	-2.6500871406	1.1800204280
H	0.6349308734	-2.3592511206	1.6284393485
H	-1.4937974187	-0.1592345776	-1.7126271789
O	-4.7524999874	-3.5257351334	-0.1892542594
C	-6.8812081476	1.5530771319	-2.2825665155
C	-7.9982452753	2.5167816385	-2.0358388717
O	-6.9061759592	0.6503733115	-3.1029521833
H	-8.7079321461	2.4807408278	-2.8614966936
H	-7.6137718880	3.5300370304	-1.9057167722
H	-8.5071081640	2.2270314710	-1.1110577651
C	-3.4549873618	-0.8052959269	-0.1493201776

C	-3.5725268894	-1.7816491118	1.0200315329
C	-4.8445129433	-2.6219486967	0.9018818687
C	-6.0754299898	-1.7458786590	0.6609846149
O	-5.8755845829	-0.8601745301	-0.4110466966
C	-4.7493028813	0.0050004776	-0.2771389710
H	-3.2581483633	-1.3492058213	-1.0813234331
H	-3.6263504764	-1.1903199431	1.9436856375
O	-2.4854031509	-2.6921436045	1.0872215910
H	-4.9900911482	-3.1768629072	1.8370731284
O	-6.3963347094	-1.0060079823	1.8169179021
H	-4.8636166169	0.6287791429	0.6193403377
C	-4.7126326248	0.8962711648	-1.4999805603
H	-3.8639455656	-3.9067316897	-0.1615590914
H	-4.7185289139	0.3059416157	-2.4177568788
H	-3.8250812604	1.5285248041	-1.4702313882
O	-5.8370662609	1.7908792937	-1.4751026102
C	-7.3250255288	-1.6577207839	2.6777057916
H	-6.9221031857	-2.3744364913	0.3645897098
H	-6.9195649001	-2.5981723218	3.0680062738
H	-8.2647163184	-1.8583139366	2.1494943311
H	-7.5114184351	-0.9770748338	3.5089037929
H	-1.6921354766	-2.2635549725	0.7140381206
H	1.0914624091	2.1961756527	1.3723640205
O	2.0031417855	2.5862810564	1.6076564836
H	1.8557327005	3.5152316126	1.8157008301
H	-0.4088975534	0.4369435429	2.0508856685
O	-0.5138290019	-0.1652817827	2.8401514688
H	-1.0413000879	-0.9016791878	2.5110988015
H	-1.6029291937	2.3818980023	1.2891260087
O	-2.4733247942	2.8358559637	1.4869688367
H	-3.1267894230	2.2250829549	1.1264616404
O	-0.2303937356	1.5893680178	0.8727632424
H	2.0229134583	2.3445253692	-0.2617401903

D-Man-TS1a

-1 1

C	-6.5703989343	0.2695266380	0.4163091599
C	-6.4139993182	-0.4233671980	-0.9380708264
C	-5.2415032755	-1.4033633428	-0.9036326681
C	-3.9942766924	-0.6653930808	-0.4112486883
O	-4.2306823757	-0.1158655373	0.8743633625
C	-5.2380007808	0.8884537553	0.8568225137
H	-6.8835344651	-0.4648552254	1.1696967721
O	-7.5257170501	1.3237794946	0.3488809966

H	-6.2081160543	0.3405642712	-1.6945949563
O	-7.6125331465	-1.0675817183	-1.3351061221
H	-5.0484905054	-1.7801504474	-1.9145256056
O	-5.6040890797	-2.4677857244	-0.0367153162
H	-3.7432122247	0.1389223415	-1.1171724515
H	-4.9586003096	1.6811412381	0.1460217568
C	-5.3002299085	1.4760192516	2.2638551647
H	-8.4056643833	0.9355421738	0.2797819169
H	-7.7083325309	-1.8615138785	-0.7910770586
H	-5.0560722082	-3.2368078298	-0.2276668322
H	-4.2899650599	1.7248561562	2.5954088808
H	-5.7147260547	0.7290487981	2.9540952774
O	-6.0606005725	2.6758566015	2.3041339001
H	-6.8976828179	2.4956328230	1.8490525611
O	-2.9372721563	-1.5687938385	-0.2918279429
O	2.4961166229	-1.2643334036	-0.3119882829
O	0.7332593004	0.7178174529	-1.0900074646
C	-1.7778506338	1.9180424936	1.2253995003
C	-2.3985179001	3.2292216499	0.8665530874
O	-1.3810525927	1.6132621164	2.3385302631
H	-3.1495259392	3.1127050769	0.0841649016
H	-1.6052947722	3.8806829153	0.4845163760
H	-2.8344909576	3.6881668514	1.7537206377
C	-1.6315300813	-1.1288157257	-0.6729588223
C	-1.0221140740	-0.1582904691	0.3346807188
C	0.4852732340	0.0333320269	0.1208833331
C	1.1477104654	-1.3404846703	0.0459167979
O	0.5743098558	-2.1011720342	-0.9921748002
C	-0.7907980316	-2.4152860234	-0.7598594941
H	-1.6790109092	-0.6568850854	-1.6622052382
H	-1.1971577540	-0.5345519126	1.3463856941
O	-1.6795672294	1.1048752629	0.1671306647
H	0.8931943936	0.5897540134	0.9673154059
H	-0.8888611974	-2.9658418710	0.1875341402
C	-1.2010357046	-3.3220154732	-1.9196697601
H	-0.4295988270	-4.0849150067	-2.0436913191
H	-1.2585442327	-2.7315038672	-2.8431984227
O	-2.4194462736	-4.0088333186	-1.6826376750
H	-3.0883444488	-3.3448494687	-1.4710605673
H	1.0243346630	-1.8645435123	1.0063235114
O	4.8760280132	-1.6094138347	3.2789012266
C	1.2087320835	2.0408815954	-1.0143891688
C	1.0449151793	2.6536239738	-2.3869211531
H	-0.0262895453	2.7874797497	-2.5691864024
H	1.4570769265	2.0112956529	-3.1672142750
H	1.5279652273	3.6306175133	-2.4246495480

C	3.4229105249	-0.9557458544	0.7282022702
C	4.2598352138	-2.2060686362	0.9961378421
C	5.3813334841	-1.9140427570	1.9873660995
C	6.1735800508	-0.6933971687	1.5261119557
O	5.3281695324	0.4080021651	1.2800083741
C	4.3229047793	0.2013751778	0.2762314398
H	2.8868837053	-0.6603610249	1.6373784490
H	4.7119488312	-2.5236365282	0.0482717904
O	3.4793124682	-3.2628038652	1.5367766942
H	6.0561797792	-2.7759931796	2.0382991609
O	6.9007772179	-1.0761448659	0.3902600502
H	4.8030373994	-0.0652460556	-0.6743711816
C	3.6409716508	1.5666944103	0.1256321821
H	4.2819514559	-2.3235062186	3.5413609849
H	4.4450468637	2.3052790296	0.2657109460
H	2.9250218404	1.7212633824	0.9471801378
C	7.7549758353	-0.0532387086	-0.1121762375
H	6.8491576399	-0.3530185222	2.3196238258
H	8.4103469359	0.3262898273	0.6811346286
H	7.1762937545	0.7748492228	-0.5341226481
H	8.3584432706	-0.5093420175	-0.8974334048
H	2.8509338395	-3.5538911400	0.8654137659
O	0.9895197244	2.6964134421	0.0311950590
O	3.0136414100	1.7807785445	-1.1108890329
H	3.2692559309	3.7019980735	-1.0112920393
H	4.2909523475	2.2327066589	-2.2544115596
H	3.1128053525	0.4593889794	-2.3472455727
O	3.5447887434	4.6331940295	-0.9074035834
O	5.0442379774	2.4777965941	-2.8370969184
O	3.1656159858	-0.3272602214	-2.9332119277
H	4.4711152519	4.5779619176	-0.6475301331
H	5.2064419027	3.4064791564	-2.6379573111
H	2.9230113445	-1.0318322371	-2.3165197656

D-Man-TS1b

-1 1

C	-6.2513608109	-0.1973720617	-0.6886764097
C	-6.0918599820	0.7657033202	0.4892019401
C	-4.8719420963	1.6662582313	0.2846212489
C	-3.6558833805	0.7828363237	0.0001381999
O	-3.8930116279	-0.0092377821	-1.1512439716
C	-4.9398535385	-0.9492222003	-0.9446029474
H	-6.5113518480	0.3701310974	-1.5921109607
O	-7.2579505231	-1.1700684235	-0.4282713297

H	-5.9409287457	0.1759985196	1.3991788798
O	-7.2668423955	1.5301530568	0.6980124910
H	-4.6824426688	2.2389187595	1.1989749532
O	-5.1550333763	2.5398259985	-0.7989755835
H	-3.4750471412	0.1290869125	0.8654494404
H	-4.7070232431	-1.5749019222	-0.0686891957
C	-4.9971418328	-1.8304945326	-2.1885446689
H	-8.1168113861	-0.7315577172	-0.4423876619
H	-7.3115048359	2.1888978851	-0.0089047526
H	-4.6909549515	3.3727526456	-0.6661893141
H	-3.9896483436	-2.1748642907	-2.4310920140
H	-5.3741420097	-1.2442881909	-3.0371787491
O	-5.7942644362	-2.9885394292	-1.9820448763
H	-6.6415932786	-2.6886417663	-1.6196636220
O	-2.5358371264	1.5740738209	-0.2608723543
O	2.8693532851	0.9967315388	0.2318987136
C	-1.4322276757	-2.1911276165	-1.0261687834
C	-2.1657961130	-3.4000255238	-0.5400283263
O	-0.8547014942	-2.1107466286	-2.0993598048
H	-3.0450184248	-3.1227146728	0.0436921661
H	-1.4887761216	-3.9612337297	0.1128623450
H	-2.4473036458	-4.0287055198	-1.3843411594
C	-1.2965742344	1.1593579693	0.3254232616
C	-0.6638355884	-0.0302615703	-0.3833622975
C	0.7685371195	-0.2717000961	0.1244849331
C	1.5215658086	1.0323397069	-0.1518256845
O	0.9584431053	2.0765358843	0.6119900142
C	-0.3680014500	2.3870410935	0.2045087561
H	-1.4622865114	0.9264034792	1.3830484546
H	-0.6469781790	0.1616515091	-1.4601531607
O	-1.4510022830	-1.2010657574	-0.1301333781
H	1.1942179900	-1.0495371600	-0.5243480027
H	-0.3597858656	2.7086774444	-0.8480070365
C	-0.8094951828	3.5561889830	1.0828625594
H	0.0042117661	4.2836387660	1.1179996667
H	-1.0048967435	3.2008600018	2.1027429961
O	-1.9427643545	4.2329464530	0.5605321870
H	-2.6183378779	3.5634288122	0.3889556596
H	1.4488208896	1.2835716783	-1.2218691150
O	5.2784746906	0.6088915248	-3.3169046322
C	1.7649070717	-2.2026189217	1.6602557763
C	1.5318625862	-2.3835980326	3.1448715267
H	0.4812685800	-2.5979510366	3.3392916473
H	1.8398853757	-1.5104768911	3.7224799348
H	2.1269804866	-3.2425185180	3.4710849683
C	3.7758452524	0.4471485639	-0.7208493026

C	4.7433049273	1.5534532450	-1.1351232508
C	5.8220041202	1.0123309814	-2.0691035115
C	6.4769884949	-0.2235653339	-1.4551675662
O	5.5156428920	-1.1800831858	-1.0612213008
C	4.5582825762	-0.7187144872	-0.1027837375
H	3.2276439392	0.0816598449	-1.5968963801
H	5.2292789106	1.9407654755	-0.2305700753
O	4.0799329053	2.6071203035	-1.8174639799
H	6.5904238507	1.7787188581	-2.2212246047
O	7.2671777897	0.2121544786	-0.3833179432
H	5.0737234465	-0.3702521309	0.8020048736
C	3.7258859380	-1.9468593160	0.2440408975
H	4.7730419400	1.3506964491	-3.6722395503
H	4.4071827241	-2.7987028852	0.3487906914
H	3.0135785494	-2.1752461898	-0.5513019201
O	3.0714461351	-1.7276694805	1.4855843934
C	8.0012973183	-0.8339286335	0.2459903341
H	7.0898347398	-0.7408795386	-2.2023926698
H	8.5749348123	-1.4032000359	-0.4953636307
H	7.3367937227	-1.5120784809	0.7911515498
H	8.6852260073	-0.3568324665	0.9483820559
H	3.4459512260	3.0107085967	-1.2119354482
O	1.3126356422	-3.0348083301	0.8303552624
O	0.8196407250	-0.6651135562	1.4600168386
H	2.2661490753	0.3983035974	2.7252791017
H	0.1676504352	0.3275038143	2.8122292790
H	-0.9742190615	-1.4319760663	2.1634665130
O	2.9746790365	0.9884939110	3.0312294529
O	-0.1498807688	0.8270487010	3.5966077570
O	-1.7711555903	-1.7355870578	2.6364142089
H	3.3616264808	1.2541497786	2.1849507668
H	0.1990841231	1.7158015957	3.4665391467
H	-2.4454737462	-1.7449884438	1.9474208223

D-Man-TS2a

-1 1

O	4.5835508109	0.4310045488	-0.2156704075
C	5.7478568593	0.4020079304	0.6083767347
C	5.9844215784	1.8249447279	1.0746817584
O	4.9840835436	2.2721637668	1.9745470586
H	4.1079126380	2.2120227194	1.5498688642
H	6.0453209919	2.4832620160	0.1973374028
H	6.9349398833	1.8766759109	1.6108067466
H	5.5644396052	-0.2429437519	1.4817929293

C	6.9175946721	-0.1732353073	-0.2038336393
O	8.0799678198	-0.3474247575	0.5972829019
H	8.5906564774	0.4694432630	0.5924671009
H	7.1376853513	0.4929204483	-1.0465202384
C	6.5348163570	-1.5537721765	-0.7316577391
O	7.5242398526	-2.0723413507	-1.6065722755
H	8.3784006411	-1.9984982698	-1.1623996940
H	6.4076917606	-2.2239334994	0.1307454640
C	5.2137657374	-1.5189343149	-1.5002072330
O	5.3234220997	-0.7795809770	-2.7047240690
H	6.0853900023	-1.1214384205	-3.1887323697
H	4.8960184877	-2.5477207250	-1.7112874870
C	4.1624717298	-0.8599649807	-0.6153830508
H	4.0090655579	-1.4783759088	0.2819798357
O	2.9813015112	-0.7175663919	-1.3370784437
C	1.8222690560	-0.4131368990	-0.5574517053
C	0.9110545687	-1.6506463227	-0.5433086902
O	-0.3434672335	-1.3184143395	0.0469527804
C	1.4724847137	-2.8066557638	0.2672177753
O	1.8390268939	-2.4502788506	1.5913885746
H	1.2580419394	-1.7371680705	1.9304191544
H	0.7181416741	-3.6037978115	0.2770727949
H	2.3661428185	-3.1943279159	-0.2304770659
H	0.7519007730	-1.9931039000	-1.5765358403
H	2.1159557721	-0.1628428980	0.4636883107
C	1.1202971171	0.7890159402	-1.1867606776
H	1.1242666397	0.6683382871	-2.2737272831
C	-0.2994948742	1.0064275279	-0.6517261990
O	-0.1710135947	1.5871679900	0.6001344010
C	1.5826034468	2.5729526414	0.3499604123
C	1.0413508590	3.9724293678	0.2549143382
H	1.8638998906	4.6275104341	-0.0548044158
H	0.2392055030	4.0444381531	-0.4797016060
H	0.6872108158	4.3018337717	1.2328319209
H	-0.8283043517	1.6725782975	-1.3558029480
C	-1.0593333884	-0.3120139076	-0.6509058775
H	-1.2269003251	-0.6502082472	-1.6860718763
O	-2.2638528478	-0.1316731828	0.0161385434
C	-3.2450155595	-1.1481568483	-0.1757000596
H	-2.9281624624	-1.8329888477	-0.9716291230
C	-3.4400343028	-1.9191686494	1.1278586140
O	-2.3274176865	-2.7462778273	1.4338936883
H	-1.5169407601	-2.3289130806	1.0892166517
H	-3.5990938387	-1.1876450129	1.9303241370
C	-4.6671338848	-2.8306883354	1.0479195547
O	-4.4421512477	-3.9011722764	0.1439013149

H	-3.5483813145	-4.2279341648	0.3160821715
H	-4.8918042458	-3.2205471267	2.0476447859
C	-5.8876958528	-2.0676386765	0.5364799648
O	-6.2920987015	-1.1875163428	1.5487804869
C	-7.4712274514	-0.4550363527	1.2263216239
H	-8.2773801689	-1.1351909814	0.9260899751
H	-7.7631883605	0.0800759000	2.1303024445
H	-7.2839436617	0.2648272334	0.4222117812
H	-6.6948934870	-2.7648870441	0.2839640233
O	-5.6048699677	-1.3980479606	-0.6747550941
C	-4.5449535136	-0.4494839602	-0.5807913550
H	-4.7872118828	0.3074722442	0.1758599180
C	-4.4076759454	0.2093999541	-1.9373804956
H	-3.5332769159	0.8617101936	-1.9605355066
H	-4.3276555575	-0.5441665232	-2.7220317811
O	-5.5899409901	0.9608267550	-2.2623885801
C	-5.7098513352	2.1944436109	-1.7561741193
O	-4.8451958487	2.7208935470	-1.0729698202
C	-7.0162113301	2.8228709532	-2.1194413183
H	-7.8141010989	2.3153945472	-1.5683749463
H	-7.0066243220	3.8792496100	-1.8546280337
H	-7.2133210803	2.6989643801	-3.1863894922
O	1.7953941272	2.0163832339	-0.9187432617
O	2.2835745759	2.2057257614	1.3043581210
H	-0.5082821197	2.3160384347	2.3228099908
O	-0.6455073023	2.7543751457	3.1802616807
H	-0.9094113767	3.6514301415	2.9478042562
H	-1.6265039135	2.6280126907	0.5148959524
O	-2.4050540098	3.2237284655	0.4319625884
H	-3.0600553384	2.7443520442	-0.0947664992
H	0.2618649735	0.3457287159	1.8615946092
O	0.5047851422	-0.2155849243	2.6357159861
H	1.2065921820	0.2790705711	3.0744361947

D-Man-TS2b

-1 1

O	4.4514823173	-0.0500820163	0.2283870656
C	5.5613627744	-0.6774071146	0.8644846638
C	6.1142643801	0.3016627447	1.8885674513
O	5.1481544116	0.6838918513	2.8517234130
H	4.6317095706	1.4168162599	2.4569871815
H	6.9434639145	-0.1756261669	2.4157295416
H	6.5025781889	1.1858395393	1.3653294887
H	5.2197873305	-1.5939436814	1.3698372241

C	6.6045067054	-1.0344620118	-0.1979202793
O	7.6756892848	-1.7149394282	0.4396001549
H	8.2966110678	-1.9905128329	-0.2461463547
H	6.9703538964	-0.1025810251	-0.6542513071
C	5.9755203135	-1.9039381722	-1.2859628536
O	6.9126939979	-2.1790488632	-2.3157387138
H	7.0179777045	-1.3644313776	-2.8273912836
H	5.7096052366	-2.8710307437	-0.8458611429
C	4.7077493146	-1.2569883991	-1.8560542669
O	5.0315361586	-0.1490908606	-2.6794550793
H	4.9864655150	0.6871813741	-2.1750699192
H	4.1956455580	-1.9885645096	-2.4888717902
C	3.7873513716	-0.8974492330	-0.6932800319
H	3.4891658019	-1.8197206397	-0.1737541403
O	2.6830811584	-0.2050504745	-1.1806997035
C	1.5266751765	-0.1990183390	-0.3401351068
C	0.5095193800	-1.2157096413	-0.8733286894
O	-0.6712743409	-1.1180300231	-0.0743377852
C	0.9497692581	-2.6679385500	-0.8327451082
O	1.3313309189	-3.1086434471	0.4625277919
H	0.5607256095	-3.0541487588	1.0405607020
H	0.1315070924	-3.2845594198	-1.2231555554
H	1.8147042386	-2.8034830288	-1.4859637939
H	0.2657741538	-0.9695408798	-1.9178665920
H	1.8068515860	-0.4591697510	0.6854321015
C	0.9540701126	1.2258617445	-0.3568499620
H	0.9259436504	1.5646271727	-1.4040577477
C	-0.4696040803	1.2742264754	0.2266088389
O	-0.2752633122	1.2168438079	1.6276238273
C	0.7083684294	2.1057029834	2.0929362015
C	0.3021892010	3.5613241879	2.0853244379
H	-0.2508152761	3.8485822085	1.1905475020
H	-0.3306539426	3.7328676532	2.9628202612
H	1.1937678386	4.1849106477	2.1711404843
O	1.4317657387	1.6607518892	3.0058416695
H	-0.9585146809	2.2112203089	-0.0648349860
C	-1.3397118785	0.1234985438	-0.2331647889
H	-1.5683545481	0.2657407428	-1.3009440371
O	-2.5041042006	0.0729318016	0.5222608276
C	-3.5642548770	-0.7145173987	-0.0270664202
H	-3.3327758522	-0.9780961045	-1.0661228656
C	-3.7502476389	-1.9837799774	0.8005404551
O	-2.6881264970	-2.9086278091	0.6207775998
H	-1.8696483839	-2.4186672428	0.4208336147
H	-3.8244305145	-1.6920833009	1.8559115389
C	-5.0367663032	-2.7087475179	0.4024593376

O	-4.9268693094	-3.2560635426	-0.9025375227
H	-4.0547128306	-3.6700115430	-0.9606241570
H	-5.2333893422	-3.5062080912	1.1297694062
C	-6.2321779759	-1.7552905370	0.3926840837
O	-6.5628351259	-1.3605142187	1.7042939580
C	-7.5646612623	-2.1709342370	2.3112494281
H	-7.2392919833	-3.2143308419	2.3925400778
H	-7.7289575733	-1.7698322462	3.3118246259
H	-8.4985214546	-2.1229671437	1.7387110120
H	-7.0883184813	-2.2416940972	-0.0867731836
O	-5.9708922760	-0.6141606565	-0.3838007796
C	-4.8319278816	0.1456214104	0.0166333974
H	-4.9748961540	0.5205193873	1.0378735698
C	-4.7140241148	1.3155966090	-0.9384507428
H	-3.8291069105	1.9040178801	-0.6952777939
H	-4.6572086878	0.9722654157	-1.9726144654
O	-5.8344820971	2.1985506687	-0.7683497526
C	-6.8260473081	2.1779863180	-1.6726950376
O	-6.8055430286	1.4873993889	-2.6778388241
C	-7.9392108941	3.0996674969	-1.2869213536
H	-8.6642130232	3.1636407910	-2.0972162677
H	-8.4269671060	2.7104177518	-0.3883484516
H	-7.5441812964	4.0906956392	-1.0522474515
H	3.2056281502	2.4754292249	1.1392346693
O	4.0298168320	2.8443307840	1.5326751661
H	4.6321695217	2.9047377573	0.7794713735
H	1.7997212659	3.5825065620	-0.3417323123
O	1.8915712704	4.4329120842	-0.8458222782
H	2.8324546831	4.4961508671	-1.0422948372
H	3.6533226770	2.0144950543	-1.1205021552
O	4.4767371986	2.3628070833	-1.4960950500
H	4.1963734012	2.9455994063	-2.2117094135
O	1.6793380859	2.0984543779	0.4444029911

D-Man-TS3a

-1 1

O	4.2278535349	0.2696695146	-0.0074948095
C	5.2846145186	0.2346892557	0.9517110626
C	5.5521526750	1.6711215642	1.3587234998
O	4.4956838265	2.2159772388	2.1311432990
H	3.6743953340	2.2273825386	1.6047627697
H	6.4497729579	1.7088852330	1.9790190570
H	5.7288835595	2.2713999503	0.4559827071
H	4.9598424099	-0.3394956095	1.8328986055

C	6.4989163428	-0.4529152341	0.3220880165
O	7.5146086133	-0.5556498844	1.3076934898
H	8.2669155086	-1.0102362397	0.9089685770
H	6.8552288379	0.1593747573	-0.5197221551
C	6.1010262196	-1.8402194784	-0.1947086264
O	7.1857432961	-2.4636568411	-0.8627761459
H	7.2828909766	-2.0304466904	-1.7225045668
H	5.8686731949	-2.4705294347	0.6702615102
C	4.8623053127	-1.7781410940	-1.0979159402
O	5.1670986634	-1.1916140059	-2.3543418481
H	5.2585255675	-0.2351415803	-2.2484721686
H	4.5225038852	-2.7948022366	-1.3101167628
C	3.7624398515	-1.0227171226	-0.3583387064
H	3.4930860658	-1.5667374932	0.5602998266
O	2.6656768706	-0.8709256816	-1.1989364404
C	1.4622449624	-0.4257911193	-0.5673902644
C	0.4391899894	-1.5727808071	-0.6049103493
O	-0.8170221307	-1.1040875535	-0.1278361071
C	0.8115678487	-2.7710562081	0.2510573800
O	0.8543677142	-2.4562797528	1.6412997670
H	-0.0222038930	-2.6184792697	2.0088079060
H	0.0895820810	-3.5733181510	0.0710538026
H	1.8000522131	-3.1330740947	-0.0419767869
H	0.3390046948	-1.9163850627	-1.6459549764
H	1.6739464869	-0.1409513519	0.4649220733
C	0.9315614948	0.7758346899	-1.3473459112
H	1.0256502577	0.5516590818	-2.4142798959
C	-0.5100115162	1.1538046338	-0.9941253615
C	1.3631380287	2.7192954842	0.0103253429
C	0.9254556902	4.1103206831	-0.3580869939
H	0.2548030282	4.1065820795	-1.2178156829
H	0.4446109245	4.5903155920	0.4953831565
H	1.8237151304	4.6823625217	-0.6183708006
H	-0.8991664018	1.7684099227	-1.8237092384
C	-1.3937619290	-0.0875339281	-0.9439587112
H	-1.5306067758	-0.4918830347	-1.9587851499
O	-2.6021098683	0.2637914944	-0.3739333182
C	-3.7509112415	-0.5291358458	-0.6662829081
H	-3.5968837481	-1.0921557691	-1.5954134028
C	-4.0044648231	-1.4880008498	0.4993912756
O	-3.0784453166	-2.5641316343	0.5234480414
H	-2.1815790377	-2.2018687823	0.3923454997
H	-3.9327605830	-0.9067650264	1.4281347056
C	-5.3970708904	-2.1008944209	0.4304315725
O	-5.5272513200	-2.9731360986	-0.6832022444
H	-4.7441814235	-3.5390279156	-0.6963737612

H	-5.5911768796	-2.6497814560	1.3601337395
C	-6.4546732548	-1.0082642133	0.2718215829
O	-6.5293603400	-0.2055943745	1.4282129316
C	-7.4354858997	-0.6897297381	2.4139477414
H	-7.1228022869	-1.6647590728	2.8041234510
H	-7.4297862139	0.0376123968	3.2262813410
H	-8.4485921433	-0.7700350017	2.0018254304
H	-7.4295557718	-1.4619491411	0.0599069296
O	-6.1747084134	-0.1905960198	-0.8330989774
C	-4.9052901120	0.4686049066	-0.8289996265
H	-4.8580953344	1.1767687270	0.0088528817
C	-4.9006787893	1.2323925799	-2.1578515568
H	-5.1043192636	0.5184731474	-2.9618916188
H	-5.7247924608	1.9567095991	-2.1268580092
O	-3.6819027169	1.8701351810	-2.4851722394
O	1.6900876604	1.9613033573	-1.1232821941
O	1.9351214901	2.4806529549	1.0830213101
H	-3.3822457437	2.4503150669	-1.7557168448
O	-0.4702083121	1.8787039020	0.1893564235
H	-1.7378847481	3.0640431178	-0.2674439903
H	-1.8802732250	1.8793582437	1.4540275240
H	-0.0321404279	0.8353418787	1.7075092065
O	-2.4939732556	3.5982915823	-0.6102735274
O	-2.6067943040	1.9517129702	2.1048927309
O	0.1552488230	0.3368691621	2.5312165165
H	-2.0917302122	4.2674082745	-1.1752455134
H	-3.2788655485	1.3572457068	1.7519553593
H	0.4461750147	-0.5359026708	2.2260700890

D-Man-TS3b

-1 1

O	-4.3212825887	0.0280579238	0.0781428062
C	-5.5505316196	-0.2939411501	-0.5833581393
C	-6.4038666593	0.9663755017	-0.5651048188
O	-5.8365195118	2.0374672943	-1.2953023309
H	-5.1094669999	2.4263587682	-0.7634026103
H	-7.3698827163	0.7361076166	-1.0199603665
H	-6.5763405491	1.2579995852	0.4796544374
H	-5.3343308798	-0.5920857324	-1.6204385768
C	-6.2640063081	-1.4458491146	0.1307660278
O	-7.3815099617	-1.8171875264	-0.6622727014
H	-7.8123817478	-2.5640416811	-0.2282433675
H	-6.6092864513	-1.0944741114	1.1147940697
C	-5.3191436456	-2.6290434450	0.3295081492

O	-5.9556048555	-3.6685347652	1.0548778036
H	-6.0386191052	-3.3740710442	1.9725213974
H	-5.0731320551	-3.0461052555	-0.6528577617
C	-4.0212276499	-2.1871240189	1.0071104630
O	-4.2322459437	-1.8286775533	2.3643703923
H	-4.6676937326	-0.9673969280	2.4093437839
H	-3.3266896511	-3.0302038697	1.0248109607
C	-3.3967010983	-1.0535069727	0.1926489066
H	-3.1371554114	-1.4184852251	-0.8113736082
O	-2.2849498020	-0.5607329308	0.8648327651
C	-1.1133436392	-0.2096051630	0.1178582170
C	-0.0650571606	-1.3098484174	0.3291654202
O	1.1790443205	-0.8691903984	-0.2164711145
C	-0.3832211412	-2.6328806242	-0.3463400435
O	-0.5716042285	-2.5162512037	-1.7492193674
H	0.2561139831	-2.2133809811	-2.1418539968
H	0.4296313507	-3.3344511762	-0.1239036337
H	-1.3054485197	-3.0453425865	0.0647475969
H	0.0528832360	-1.4972621310	1.4068029495
H	-1.3486813651	-0.1368272858	-0.9467129350
C	-0.6433023907	1.1606256101	0.6375214715
H	-0.8027514659	1.1572864100	1.7260890270
C	0.8487275911	1.4476711439	0.3716564843
O	0.8926574721	1.9494891173	-0.9497100582
C	-0.0528631297	2.9621251381	-1.1747682093
C	0.2331755353	4.2727995850	-0.4764396046
H	0.6403294140	4.1541421294	0.5276156805
H	0.9619569292	4.8147172743	-1.0883148512
H	-0.6839644029	4.8620243967	-0.4227593057
H	1.2081231653	2.1984582400	1.0851222924
C	1.7301573722	0.2244867474	0.4996909076
H	1.8000097723	-0.0491111255	1.5635475147
O	2.9855839472	0.4894453903	-0.0286390072
C	4.0420932674	-0.3851938930	0.3800014185
H	3.7310956945	-0.9561135605	1.2636749714
C	4.4027162938	-1.3378569936	-0.7576336428
O	3.4111664088	-2.3305036551	-0.9754725118
H	2.5319264993	-1.9398231691	-0.8153861086
H	4.5469686551	-0.7386505925	-1.6658912395
C	5.7034320604	-2.0803442924	-0.4550123483
O	5.5308962974	-2.9928177367	0.6191291371
H	4.6906515220	-3.4470944068	0.4686788885
H	6.0190840699	-2.6193192994	-1.3568102082
C	6.8148443758	-1.1112280160	-0.0492962095
O	7.2173094187	-0.3225367166	-1.1461843048
C	8.2725053742	-0.8942459855	-1.9129620244

H	7.9737608018	-1.8511406179	-2.3557442727
H	8.4966758677	-0.1863186114	-2.7114113774
H	9.1643280473	-1.0423709435	-1.2925558598
H	7.6676336656	-1.6721645580	0.3492465516
O	6.4006414681	-0.2684813974	0.9941310631
C	5.2327831316	0.5133171910	0.7293106367
H	5.4248089461	1.1903840007	-0.1127803307
C	4.9670563034	1.3294508186	1.9812945765
H	4.0274877132	1.8722668239	1.8625521591
H	4.8751553988	0.6539865664	2.8415070563
O	5.9744430846	2.3040461980	2.2128693239
O	-0.5456886788	2.9762606780	-2.3230697607
H	6.8047772647	1.8438948546	2.3836848803
O	-1.3062561338	2.2289765058	0.0272021715
H	-2.1920099773	1.7468586653	-1.6904919162
H	-2.9984475018	2.7300969494	0.1886516536
H	-1.3996484552	3.3176424561	1.4974217439
O	-2.8872471480	1.3264464969	-2.2317678186
O	-3.8809745455	3.1518803880	0.2948939794
O	-1.4290141370	3.7918542454	2.3565598286
H	-3.4957891403	0.9785476629	-1.5640850182
H	-3.7334608229	4.0799460149	0.0804919283
H	-0.5036377911	3.8937244744	2.6044448679

D-Man-TS4a

-1 1

C	-6.5160437947	0.9565951814	-0.0123448003
C	-6.6877224652	-0.5540594709	-0.1745384563
C	-5.5701597678	-1.3424372224	0.5325593594
C	-4.2421432009	-0.8026082216	0.0121061088
O	-4.1598021562	0.5782529224	0.3193309904
C	-5.0950817430	1.3644024133	-0.4125420817
H	-6.6831379483	1.2290647213	1.0398131138
O	-7.4228570188	1.6639909762	-0.8439528003
H	-6.6275645173	-0.7846275725	-1.2470886932
O	-7.9734432188	-0.8911095984	0.3207118693
H	-5.6622166335	-2.4000974638	0.2711430733
O	-5.6483455934	-1.2728473257	1.9449783849
H	-4.1893061573	-0.9301233475	-1.0801585372
H	-4.9556370217	1.1879340604	-1.4899026555
C	-4.7700729581	2.8104988490	-0.0863300417
H	-8.3175956770	1.3960402496	-0.6011994698
H	-8.2068164634	-1.7710464658	0.0018802811
H	-5.3947881355	-0.3857496729	2.2325789164

H	-4.8891518524	2.9752738689	0.9923699046
H	-5.4555521464	3.4717550400	-0.6186234621
O	-3.4565442426	3.1581029088	-0.5041309262
H	-2.8294623554	2.5808709337	-0.0482475942
O	-3.1789168478	-1.4440904156	0.6412170932
O	2.1144104690	-1.5556376553	-0.7671836422
O	0.3526688854	0.6005162587	-0.4566766412
C	-1.9026577844	-1.1418085120	0.0799557067
C	-0.9705771541	-0.6370154746	1.1808324194
C	0.4117030832	-0.3779273550	0.5772763174
C	0.9093758464	-1.6826108023	-0.0558199584
O	0.0077539095	-2.1241236326	-1.0423259957
C	-1.2986809094	-2.4121758550	-0.5433032454
H	-2.0086812135	-0.3695179442	-0.6894873837
H	-0.8619283002	-1.4396048590	1.9228322442
O	-1.4923495976	0.4995406248	1.8413549342
H	1.0848827044	-0.0514216600	1.3720626259
H	-1.2287310381	-3.1906973215	0.2315740580
C	-2.1164973266	-2.9292070645	-1.7059214021
H	-2.1028443730	-2.1919398198	-2.5183464218
H	-3.1510151876	-3.0727772442	-1.3757194390
O	-1.5602394038	-4.1655599386	-2.1393208003
H	-2.0740477720	-4.4782352359	-2.8921672756
H	1.0020615315	-2.4400871056	0.7377659699
O	5.1046305210	-1.0134921413	2.2510572671
C	0.5851500409	1.9181231471	-0.1262408533
C	0.2513306968	2.7800335082	-1.3152427750
H	0.4766372104	3.8214555381	-1.0886041412
H	-0.8258952604	2.6870106383	-1.4916564009
H	0.7763613136	2.4693666195	-2.2199996118
C	3.2268962896	-1.0509729855	-0.0343728837
C	4.2149696904	-2.1679111049	0.2915973433
C	5.4411771084	-1.5776789994	0.9875146139
C	6.0326288037	-0.4438696317	0.1536035683
O	5.0589788806	0.5200012132	-0.1682294756
C	3.9211560059	0.0223337528	-0.8823309714
H	2.8890503751	-0.6140631748	0.9036624378
H	4.5389251824	-2.6480679435	-0.6396735066
O	3.6610316728	-3.1389135555	1.1698276230
H	6.1991406491	-2.3572500957	1.1183786814
O	6.6123663265	-1.0253466194	-0.9842452171
H	4.2580757747	-0.4436890567	-1.8198708759
C	3.0614876836	1.2184290444	-1.2703932089
H	4.6766380780	-1.6980543957	2.7806674946
H	2.2702572092	0.8331752938	-1.9286325619
H	3.7027837417	1.8609933478	-1.8957277442

C	7.2958693184	-0.0906054810	-1.8134566068
H	6.7868860213	0.1022538231	0.7323410257
H	8.0572282603	0.4500430777	-1.2381711657
H	6.5995468435	0.6268389325	-2.2594533334
H	7.7769230125	-0.6675320733	-2.6038083790
H	3.0225038947	-3.6701798624	0.6801900632
O	0.3656194922	2.3116484420	1.0469800588
H	-0.9057194102	1.2620582383	1.6503338017
O	2.5154090991	1.9507943617	-0.2064096559
H	2.9031410907	1.4182569729	1.6034828775
H	3.9268348004	2.9548890192	0.5961801833
H	2.7657431168	3.6251184116	-0.9216096511
O	3.1972168890	1.1993083583	2.5122503145
O	4.6016054419	3.4420538452	1.1105335002
O	3.0223736693	4.4691763352	-1.3561427370
H	3.8897911271	0.5269066171	2.3936544923
H	4.6445359341	2.9332013517	1.9300616262
H	2.5469159516	4.4637934795	-2.1937308637

D-Man-TS4b

-1 1

C	6.5403910381	0.2563016792	-0.4777402587
C	6.2539382109	-1.0481662160	0.2586141258
C	4.9680587908	-1.7126525409	-0.2532245868
C	3.8409179820	-0.6874299419	-0.1363822137
O	4.1611782484	0.4518776287	-0.9250701382
C	5.3052115475	1.1644232482	-0.4573760738
H	6.7972013023	0.0298834189	-1.5230659186
O	7.6066274978	0.9644004732	0.1363655519
H	6.1055476388	-0.8119087250	1.3212283098
O	7.3908508006	-1.8827809347	0.1028999757
H	4.7312816990	-2.5693135070	0.3839090821
O	5.0882316181	-2.2234418657	-1.5696267907
H	3.7287977614	-0.3822404245	0.9144543340
H	5.1233632582	1.5058271941	0.5727808483
C	5.4783608608	2.3591993368	-1.3796257942
H	8.3751608147	0.3808054012	0.1534795256
H	7.3515806168	-2.5852487702	0.7625324948
H	5.1446883691	-1.4898571657	-2.1956449374
H	5.6345821166	2.0002191447	-2.4051151421
H	6.3579252700	2.9271659499	-1.0722654362
O	4.3765181782	3.2529591652	-1.3275702347
H	3.6003969514	2.7973115430	-1.6760607316
O	2.6591527792	-1.2243874623	-0.6291616282

O	-2.6861671937	-1.0891920850	0.5189355627
C	1.4440324899	-0.8045563690	-0.0024028420
C	0.5749957527	-0.0320127603	-0.9826352636
C	-0.7996490202	0.2141941170	-0.3541371423
C	-1.3994463006	-1.1589595254	-0.0583454924
O	-0.6159941319	-1.8241023012	0.9045334872
C	0.7130148602	-2.0878556658	0.4522611353
H	1.6784734339	-0.1782640547	0.8627259720
H	0.4413410282	-0.6600735939	-1.8750487243
O	1.1772867829	1.1885717915	-1.3859807058
H	-1.4107974110	0.6708446155	-1.1474718440
H	0.6671355343	-2.7718175977	-0.4093246810
C	1.4432309049	-2.7585029601	1.5944852631
H	1.3510104245	-2.1366231341	2.4936409223
H	2.5037686838	-2.8509831141	1.3369931471
O	0.8710656173	-4.0444179205	1.8122268744
H	1.3244454375	-4.4497461337	2.5593214058
H	-1.4434172848	-1.7607105560	-0.9807482924
O	-5.2846557963	-0.8439694723	-2.8862477565
C	-1.5952895470	2.7245327690	0.1898166090
C	-1.4939231199	3.4071108868	1.5306870556
H	-2.0836199145	4.3292776911	1.4827215386
H	-0.4520912895	3.6719979655	1.7123341049
H	-1.8566588837	2.8035616765	2.3614458776
C	-3.6972647881	-0.6054096127	-0.3613135484
C	-4.6633854192	-1.7384903049	-0.7057104562
C	-5.7821149099	-1.2215988945	-1.6110978558
C	-6.4324850769	0.0236123188	-1.0089843383
O	-5.4707345831	0.9911484343	-0.6409697515
C	-4.4991137239	0.5244707808	0.3020304309
H	-3.2503869274	-0.2263457812	-1.2842817313
H	-5.1098404949	-2.1150654258	0.2233038844
O	-4.0242822926	-2.7995979272	-1.3995250227
H	-6.5475904086	-1.9982946131	-1.7200766318
O	-7.2061875277	-0.3999316415	0.0807361972
H	-5.0164822467	0.1203780013	1.1833386563
C	-3.7087154430	1.7101913819	0.8099882769
H	-4.7748782660	-1.5855345876	-3.2361614021
H	-3.1489763579	1.3806809518	1.6803723114
H	-4.4112658622	2.4911814224	1.1186378035
O	-2.8296412148	2.2360036817	-0.1847566655
C	-7.9394080309	0.6501969628	0.7035856046
H	-7.0585268851	0.5249771086	-1.7560286396
H	-8.5389506810	1.1939918890	-0.0363850916
H	-7.2715915821	1.3504783214	1.2156353380
H	-8.5989343386	0.1799115902	1.4334551639

H	-3.3875057782	-3.2153475119	-0.8059013987
O	-0.8952735446	3.1054225870	-0.7779824347
H	0.5444305114	1.9089685921	-1.2012426082
O	-0.7597500291	1.0384886872	0.7640034817
H	-1.5952460934	0.3371093059	2.2816868332
H	0.3907096382	0.8353590495	2.2574414658
H	1.2044581463	2.2504138292	0.9686219465
O	-1.9591044050	0.0187007425	3.1392895860
O	0.8078044916	0.7153683063	3.1357758830
O	2.1325249523	2.5279626181	1.0110328971
H	-2.5176601264	-0.7207572899	2.8728264177
H	0.1262814158	0.2359820859	3.6255165923
H	2.5210034741	2.0570080429	0.2600992319

References

(1) Bonora, B.; Boos, I.; Clausen, M. H. Convergent Strategy for the Synthesis of S-Linked Oligoxylans. *Carbohydr. Res.* **2017**, *443–444*, 53–57.

(2) Phanumartwiwath, A.; Hornsby, T. W.; Jamalis, J.; Bailey, C. D.; Willis, C. L. Silyl Migrations in D-Xylose Derivatives: Total Synthesis of a Marine Quinoline Alkaloid. *Org. Lett.* **2013**, *15*, 5734–5737.

(3) Zemplén, G.; Kunz, A. Studien Über Amygdalin, IV: Synthese Des Natürlichen l-Amygdalins. *Ber. Dtsch. Chem. Ges.* **1924**, *57*, 1357–1359.

(4) Petráková, E.; Kováč, P. Synthesis of New Methyl O-Acetyl-α- and -β-D-Xylopyranosides. *Carbohydr. Res.* **1982**, *101*, 141–147.

(5) Blom, P.; Ruttens, B.; van Hoof, S.; Hubrecht, I.; van der Eycken, J.; Sas, B.; van hemel, J.; Vandenkerckhove, J. A Convergent Ring-Closing Metathesis Approach to Carbohydrate-Based Macrolides with Potential Antibiotic Activity. *J. Org. Chem.* **2005**, *70*, 10109–10112.

(6) Das, A.; Jayaraman, N. Carbon Tetrachloride-Free Allylic Halogenation-Mediated Glycosylations of Allyl Glycosides. *Org. Biomol. Chem.* **2021**, *19*, 9318–9325.

(7) Garegg, P. J.; Hultberg, H. A Novel, Reductive Ring-Opening of Carbohydrate Benzylidene Acetals, with Unusual Regioselectivity. *Carbohydr. Res.* **1981**, *93*, C10–C11.

(8) ChemAdder/SpinAdder. *Spin Discoveries Ltd*. (http://www.chemadder.com).

(9) Frisch, M. J.; Trucks, G. W.; Schlegel, H. B.; Scuseria, G. E.; Robb, M. A.; Cheeseman, J. R.; Scalmani, G.; Barone, V.; Mennucci, B.; Petersson, G. A.; Nakatsuji, H.; Caricato, M.; Li, X.; Hratchian, H. P.; Izmaylov, A. F.; Bloino, J.; Zheng, G.; Sonnenb, D. J.; Gaussian, Inc. *Wallingford CT* **2009**.

(10) Lassfolk, R.; Pedrón, M.; Tejero, T.; Merino, P.; Wärnå, J.; Leino, R. Acyl Group Migration in Pyranosides as Studied by Experimental and Computational Methods. *Chem. Eur. J.* **2022**, *28*, e202200499.

(11) Chai, J.-D.; Head-Gordon, M. Long-Range Corrected Hybrid Density Functionals with Damped Atom–Atom Dispersion Corrections. *Phys. Chem. Chem. Phys.* **2008**, *10*, 6615–6620.

(12) Ditchfield, R.; Hehre, W. J.; Pople, J. A. Self-Consistent Molecular-Orbital Methods. IX. An Extended Gaussian-Type Basis for Molecular-Orbital Studies of Organic Molecules. *J. Chem. Phys.* **1971**, *54*, 724–728.

(13) Hehre, W. J.; Ditchfield, R.; Pople, J. A. Self-Consistent Molecular Orbital Methods. XII. Further Extensions of Gaussian-Type Basis Sets for Use in Molecular Orbital Studies of Organic Molecules. *J. Chem. Phys.* **1972**, *56*, 2257–2261.

(14) Marenich, A. v; Cramer, C. J.; Truhlar, D. G. Universal Solvation Model Based on Solute Electron Density and on a Continuum Model of the Solvent Defined by the Bulk Dielectric Constant and Atomic Surface Tensions. *J. Phys. Chem. B* **2009**, *113*, 6378–6396.

(15) Schlegel, H. B. Optimization of Equilibrium Geometries and Transition Structures. *J. Comput. Chem.* **1982**, *3*, 214–218.

(16) Fukui, K. Formulation of the Reaction Coordinate. *J. Phys. Chem.* **1970**, *74*, 4161–4163.

(17) Bootsma, A. N.; Wheeler, S. E. Popular Integration Grids Can Result in Large Errors in DFT-Computed Free Energies. *ChemRxiv.* **2019**.

(18) Garrett, B. C.; Truhlar, D. G.; Grev, R. S.; Magnuson, A. W. Improved Treatment of Threshold Contributions in Variational Transition-State Theory. *J. Phys. Chem.* **1980**, *84*, 1730–1748.

(19) Alhambra, C.; Corchado, J.; Sánchez, M. L.; Garcia-Viloca, M.; Gao, J.; Truhlar, D. G. Canonical Variational Theory for Enzyme Kinetics with the Protein Mean Force and Multidimensional Quantum Mechanical Tunneling Dynamics. Theory and Application to Liver Alcohol Dehydrogenase. *J. Phys. Chem. B* **2001**, *105*, 11326–11340.

(20) Case, D. A.; Aktulga, H. M.; Belfon, K.; Ben-Shalom, I. Y.; Brozell, S. R.; Cerutti, D. S.; Cheatham, T. E. I.; Cisneros, G. A.; Cruzeiro, V. W. D.; Darden, T. A.; Duke, R. E.; Giambasu, G.; Gilson, M. K.; Gohlke, H.; Goetz, A. W.; Harris, R.; Izadi, S.; Izmailov, S. A.; Jin, C.; Kasavajhala, K.; Kaymak, M. C.; King, E.; Kovalenko, A.; Kurtzman, T.; Lee, T. S.; LeGrand, S.; Li, P.; Lin, C.; Liu, J.; Luchko, T.; Luo, R.; Machado, M.; Man, V.; Manathunga, M.; Merz, K. M.; Miao, Y.; Mikhailovskii, O.; Monard, G.; Nguyen, H.; O'Hearn, K. A.; Onufriev, A.; Pan, F.; Pantano, S.; Qi, R.; Rahnamoun, A.; Roe, D. R.; Roitberg, A.; Sagui, C.; Schott-Verdugo, S.; Shen, J.; Simmerling, C. L.; Skrynnikov, N. R.; Smith, J.; Swails, J.; Walker, R. C.; Wang, J.; Wei, H.; Wolf, R. M.; Wu, X.; Xue, Y.; York, D. M.; Zhao, S.; Kollman, P. A. Amber 2020. *University of California, San Francisco.* 2020.

(21) Wang, J.; Wolf, R. M.; Caldwell, J. W.; Kollman, P. A.; Case, D. A. Development and Testing of a General Amber Force Field. *J. Comput. Chem.* **2004**, *25*, 1157–1174.

(22) Jorgensen, W. L.; Chandrasekhar, J.; Madura, J. D.; Impey, R. W.; Klein, M. L. Comparison of Simple Potential Functions for Simulating Liquid Water. *J. Chem. Phys.* **1983**, *79*, 926–935.

(23) Matubayasi, N.; Nakahara, M. Reversible Molecular Dynamics for Rigid Bodies and Hybrid Monte Carlo. *J. Chem. Phys.* **1999**, *110*, 3291–3301.

(24) Darden, T.; York, D.; Pedersen, L. Particle Mesh Ewald: An *N* ·log(*N*) Method for Ewald Sums in Large Systems. *J. Chem. Phys.* **1993**, *98*, 10089–10092.

(25) Schrödinger Release 2020-1: MacroModel, Schrödinger, LLC, New York, NY, 2021.

(26) Banks, J. L.; Beard, H. S.; Cao, Y.; Cho, A. E.; Damm, W.; Farid, R.; Felts, A. K.; Halgren, T. A.; Mainz, D. T.; Maple, J. R.; Murphy, R.; Philipp, D. M.; Repasky, M. P.; Zhang, L. Y.; Berne, B. J.; Friesner, R. A.; Gallicchio, E.; Levy, R. M. Integrated Modeling Program, Applied Chemical Theory (IMPACT). *J. Comput. Chem.* **2005**, *26* (16), 1752–1780.
